# Evidence-based guideline diagnosis, treatment, prevention and aftercare of oropharyngeal and hypopharyngeal carcinoma

**DOI:** 10.3205/000339

**Published:** 2025-06-24

**Authors:** Andreas Dietz, Kathy Taylor, Oliver Bayer, Susanne Singer, Markus Follmann, Monika Nothacker, Thomas Langer, Peter Klussmann, Stephan Lang, Thomas Hoffmann, Georg Maschmeyer, Susanne Wiegand, Michael Fuchs, Wilko Weichert, Jochen Heß, Orlando Guntinas-Lichius, Tim Waterboer, Michael Lell, Jens Büntzel, Panagiotis Balermpas, Kerstin Schmidt, Maria Steingräber, Gunther Klautke, Herbert Hellmund, Gunthard Kissinger, Peter Brossart, Imad Maatouk, Bernd Lethaus, Jan Raguse, Klaus Zöphel, Kristina Lippach, Fritz Sterr, Hans Christiansen, Christian Duncker, Annerose Keilmann, Havva Cici, Jutta Yzer, Alessandro Relic, Kerstin Paradies, Wilfried Budach

**Affiliations:** 1 Hals-Nasen-Ohren-Universitätsklinik Leipzig, Germany; 2 IMBEI, Institut für Medizinische Biometrie, Epidemiologie und Informatik, Universität Mainz, Germany; 3 Deutsche Krebsgesellschaft, Geschäftsstelle Berlin, Germany; 4 Arbeitsgemeinschaft Medizinischer Fachgesellschaften, AWMF, Berlin, Germany; 5 Hals-Nasen-Ohren-Universitätsklinik Köln, Germany; 6 Hals-Nasen-Ohren-Universitätsklinik Essen, Germany; 7 Hals-Nasen-Ohren-Universitätsklinik Ulm, Germany; 8 Charité, Medizinische Klinik Hämatologie, Onkologie und Tumorimmunologie, CBF, Berlin, Germany; 9 Hals-Nasen-Ohren-Universitätsklinik Kiel, Germany; 10 Hals-Nasen-Ohren-Universitätsklinik, Sektion Phoniatrie und Audiologie, Leipzig, Germany; 11 Institut für Allgemeine Pathologie und Pathologische Anatomie TU München, Munich, Germany; 12 Hals-Nasen-Ohren-Universitätsklinik und DKFZ, Heidelberg, Germany; 13 Hals-Nasen-Ohren-Universitätsklinik Jena, Germany; 14 Division Head, Infections and Cancer Epidemiology DKFZ, Heidelberg, Germany; 15 Institut für Radiologie und Nuklearmedizin, Klinikum Nürnberg, Nuremburg, Germany; 16 Hals-Nasen-Ohren-Klinik, Klinikum Nordhausen, Germany; 17 Klinik für Radio-Onkologie, Universitätsspital Zürich, Switzerland; 18 Sozialdienst Universitätsklinikum Heidelberg, Germany; 19 Praxis für Strahlentherapie Moabit, Berlin, Germany; 20 Klinik für Radioonkologie, Klinikum Chemnitz, Germany; 21 Bundesverband der Kehlkopfoperierten e.V., Bonn, Germany; 22 Kopf-Hals-M.U.N.D.-Krebs e.V., Bonn, Germany; 23 Med. Klinik III, Universitätsklinikum Bonn, Germany; 24 Med. Klinik II, Universitätsklinikum Würzburg, Germany; 25 Klinik für Mund-, Kiefer-, Gesichtschirurgie, Universitätsklinikum Tübingen, Germany; 26 Mund-Kiefer-Gesichtschirurgie, Fachklinik Hornheide, Germany; 27 Klinik für Nuklearmedizin, Klinikum Chemnitz, Germany; 28 Stabsstellen der Pflege, TU München, Munich, Germany; 29 Klinik für Strahlentherapie und Spezielle Onkologie, MHH Hannover, Germany; 30 Parksanatorium Aulendorf, Germany; 31 Stimmheilzentrum Bad Rappenau, Germany; 32 Sozialdienst, DIAKO Ev. Diakonie-Krankenhauses, Bremen, Germany; 33 Hals-Nasen-Ohren-Gemeinschaftspraxis, Bad Kreuznach, Germany; 34 Gynäkologische-onkologische Praxisklinik, Hamburg, Germany; 35 Klinik für Strahlentherapie und Radioonkologie, Universitätsklinikum Düsseldorf, Germany

**Keywords:** oropahyrnx carcinoma, hypopharynx carcinoma, head and neck cancer, p16, HPV16

## Abstract

The guideline is being drawn up as a joint guideline for oropharyngeal and hypopharyngeal carcinoma. Oropharyngeal carcinoma in particular has experienced the greatest increase in incidence among all head and neck carcinomas in the last 20 years and is now the sixth most common cancer in men in Germany. Together with hypopharyngeal carcinoma, these tumors are currently the most common cancer entity in the head and neck region. Due to the association with human papillomavirus type 16 (HPV16), we now distinguish two groups of oropharyngeal carcinomas in Germany: HPV16-positive (approx. 35%) and HPV-negative (approx. 65%). A HPV16 association with hypopharyngeal carcinoma has not been described. The therapy covers the entire spectrum of head and neck surgery, including diversified reconstructive procedures, transoral and external approaches, the options for primary and adjuvant radiotherapy (possibly in combination with chemotherapy) and the current recommendations for drug-based tumor therapy, which range from classic chemotherapy to immuno-oncology. In addition, measures for early detection and prevention are carried out, with particular consideration of the HPV16-associated genesis of oropharyngeal carcinoma, as well as adequate rehabilitation after the primary treatment of oropharyngeal and hypopharyngeal carcinomas. Finally, the treatment options for recurrences or distant metastases that cannot be cured in the further course of the disease are shown and classified.

## Abstract (de)

Die Leitlinie wurde als gemeinsame Leitlinie für das Oro- und Hypopharynxkarzinom erstellt. Insbesondere das Oropharynxkarzinom zeichnet sich durch die stärkste Inzidenzzunahme unter allen Kopf-Hals-Karzinomen in den letzten 20 Jahren aus und rangiert mittlerweile an sechster Stelle der häufigsten Krebserkrankungen bei Männern in Deutschland. Zusammen mit dem Hypopharynxkarzinom sind diese Tumore die aktuell am häufigsten vertretene Krebsentität im Kopf-Hals-Bereich. Durch die Assoziation mit dem humanen Papillomavirus Typ 16 (HPV16) unterscheiden wir heute zwei Gruppen der Oropharynxkarzinome in Deutschland: die HPV16 positiven (ca. 35%) und HPV-negativen (ca. 65%). Eine HPV16-Assoziation des Hypopharynxkarzinoms wird nicht beschrieben. Die Therapie umfasst das gesamte Spektrum der Kopf-Hals-Chirurgie mitsamt diversifizierten rekonstruktiven Verfahren, transoralen und äußeren Zugängen, den Möglichkeiten der primären und adjuvanten Strahlentherapie (ggf. in Kombination Chemotherapie) sowie die aktuellen Empfehlungen zur medikamentösen Tumortherapie, die von der klassischen Chemotherapie bis zur Immunonkologie reichen. Des Weiteren werden Maßnahmen zur Früherkennung und Prävention unter besonderer Berücksichtigung der HPV16-assoziierten Genese des Oropharynxkarzinoms, sowie der adäquaten Rehabilitation nach der primären Behandlung von Oro- und Hypopharynxkarzinomen ausgeführt. Schließlich werden die Möglichkeiten einer Behandlung bei nicht mehr kurativ therapiebaren Rezidiven bzw. Fernmetastasen im weiteren Verlauf der Erkrankung aufgezeigt und eingeordnet.

## 1 Information about this guideline

### 1.1 Special comment

The field of medicine is subject to a continuous process of further development, so that all details provided here, and in particular those on diagnostic and therapeutic procedures, can always only represent the state of knowledge at the time when the medical care guideline was printed. The greatest possible care has been taken with regard to the treatment recommendations given and to the choice and dosage of drugs. However, users are requested to check by referring to the patient package inserts and specialist information provided by the manufacturers, and in cases of doubt to consult a specialist. In the general interest of the guideline editors, readers are requested to draw attention to any questionable points or inconsistencies found.

Users themselves remain responsible for all diagnostic and therapeutic applications, medications, and dosages.

**Registered trademarks (protected proprietary names) are not specially identified in this guideline. The absence of an indication of this type can therefore not be taken to suggest that such names are unregistered product names.**

All parts of this guideline are protected by copyright. Any usage outside of the provisions of copyright law without written permission from the German Guideline Program in Oncology editors is therefore unlawful and liable to prosecution. No part of this work may be reproduced in any form without written permission from the German Guideline Program in Oncology editors. This applies in particular to reproduction, translation, microfilming and storage, usage and exploitation in electronic systems, intranets and the internet.

### 1.2 Objectives of the GGPO

The aim of the Association of the Scientific Medical Societies in Germany (AWMF), the German Cancer Society (DKG), and the German Cancer Aid Foundation (Stiftung Deutsche Krebshilfe) in implementing the German Guideline Program in Oncology (GGPO) is to jointly promote and support the development, updating, and use of scientifically based and practicable guidelines in oncology. The program is based on medical and scientific findings established by the specialist societies and the DKG, consensus among medical experts, users and patients, as well as the AMWF’s regulations for guideline development. The program receives specialist support and financing from the German Cancer Aid. In order to reflect the current state of medical knowledge and to take account of medical progress, guidelines have to be regularly checked and updated. The use of the AWMF regulations is intended to provide a basis for developing high-quality oncological guidelines in this framework. As guidelines represent an important instrument for quality assurance and quality management in oncology, they are intended to be used in a targeted and sustained way in everyday medical care. Active implementation measures as well as evaluation programs are therefore important components of the support provided by the German Guideline Program in Oncology. The aim of the program is to create professional preconditions, with secure medium-term financing, for the development and provision of high-quality guidelines in Germany. High-quality guidelines of this type not only support structured knowledge transfer but can also be used in the design of health-care structures. Relevant aspects of this include evidence-based guidelines as a basis for establishing and updating disease management programs, and the use of quality indicators derived from guidelines in the context of certification procedures for organ tumour centres.

### 1.3 Additional documents relating to this guideline

- Short version of the guideline
- Patient guideline
- Guideline report on the guideline development process
- Evidence report

This guideline and all additional documents can be accessed via the following web sites:

- German Guideline Program in Oncology (https://www.leitlinienprogramm-onkologie.de/leitlinien/oro-und-hypopharynxkarzinom)
- AWMF (https://register.awmf.org/de/leitlinien/detail/017-082OL)
- Guidelines International Network (https://www.g-i-n.net)

### 1.4 Composition of the guideline group

#### 1.4.1 Guideline coordination

**Guideline coordinators:**

Prof. Dr. Andreas Dietz (University of Leipzig Medical Center)

Prof. Dr. Wilfried Budach (Düsseldorf University Hospital)

#### 1.4.2 Involved professional societies and organisations

Participating professional associations and organizations (alphabetical), and their representative(s)

- Abteilung Experimentelle Krebsforschung in der DKG (AEK)_history: Prof. Sigrun Smola
- Arbeitsgemeinschaft Bildgebung in der Onkologie der DKG (ABO): Prof. Dr. Michael Lell
- Arbeitsgemeinschaft Hals-Nasen-Ohren-Heilkunde, Mund-Kiefer-Gesichtschirurgische Onkologie in der DKG (AHMO): Prof. Dr. Jens Peter Klußmann
- Arbeitsgemeinschaft Palliativmedizin in der DKG (APM): Prof. Dr. Jens Büntzel
- Arbeitsgemeinschaft Prävention und integrative Medizin in der Onkologie in der DKG (PRiO): Prof. Dr. Jens Büntzel
- Arbeitsgemeinschaft Radiologische Onkologie in der DKG (ARO): Prof. Dr. Panagiotis Balermpas
- Arbeitsgemeinschaft Soziale Arbeit in der Onkologie (ASO) in DKG: Kerstin Schmidt
- Arbeitsgemeinschaft Supportive Maßnahmen in der Onkologie in der DKG (AGSMO): Dr. Maria Steingräber
- Arbeitsgemeinschaft Tumorklassifikation in der Onkologie der DKG (ATO): Prof. Dr. Stefan Mönig
- Arbeitsgemeinschaft für Psychoonkologie in der DKG (PSO): Prof. Dr. Imad Maatouk, Prof. Dr. Susanne Singer
- Berufsverband Deutscher Strahlentherapeuten (BVDST): PD Dr. Gunther Klautke
- Berufsverband der Ärzte für Mund-, Kiefer- und Gesichtschirurgie (BVMKG): Prof. Dr. Dr. André Eckardt
- Bundesverband der Kehlkopfoperierten (Patientenvertretung): Herbert Hellmund
- Deutsche Gesellschaft für Hämatologie und Medizinische Onkologie (DGHO): Prof. Dr. Peter Brossart, Prof. Dr. Georg Maschmeyer
- Deutsche Gesellschaft für Medizinische Psychologie (DGMP): Prof. Dr. Imad Maatouk
- Deutsche Gesellschaft für Mund-, Kiefer- und Gesichtschirurgie (DGMKG): Prof. Dr. Bernd Lethaus, Prof. Dr. Dr. Jan Raguse
- Deutsche Gesellschaft für Nuklearmedizin (DGN): Prof. Dr. Klaus Zöphel
- Deutsche Gesellschaft für Palliativmedizin (DGP): Prof. Dr. Jens Büntzel
- Deutsche Gesellschaft für Pathologie (DGP): Prof. Dr. Wilko Weichert^†^
- Deutsche Gesellschaft für Pflegewissenschaft (DGP): Kristina Lippach, Fritz Sterr – Stellvertr.
- Deutsche Gesellschaft für Phoniatrie und Pädaudiologie (DGPP): Prof. Dr. Michael Fuchs
- Deutsche Gesellschaft für Radioonkologie (DEGRO): Prof. Dr. Wilfried Budach, Prof. Dr. Hans Christiansen
- Deutsche Gesellschaft für Rehabilitationswissenschaften (DGRW): Dr. Christian Duncker, Prof. Dr. Annerose Keilmann
- Deutsche Gesellschaft für Virologie (GfV): Dr. Tim Waterboer
- Deutsche Röntgengesellschaft (DRG): Prof. Dr. Michael Lell
- Deutsche Vereinigung für Soziale Arbeit im Gesundheitswesen (DVSG): Havva Cici, Jutta Yzer
- Deutscher Berufsverband der Hals-Nasen-Ohrenärzte: Dr. Alessandro Relic
- Deutsches Krebsforschungszentrum (DKFZ), Abteilung „Infektionen und Krebs-Epidemiologie (F020)“: Dr. Tim Waterboer
- Eingeladenen Fachexperten ohne Mandat: Prof. Dr. Orlando Guntinas-Lichius, Prof. Dr. Jochen Heß, Prof. Dr. Susanne Wiegand
- Interdisziplinäre Arbeitsgruppe Kopf-Hals-Tumoren (IAG-KHT): Prof. Dr. Wilfried Budach, Prof. Dr. Andreas Dietz
- Konferenz Onkologischer Kranken- und Kinderkrankenpflege in der DKG (KOK): Kerstin Paradies
- Neuroonkologische Arbeitsgemeinschaft in der DKG (NOA): Prof. Dr. Stephanie E. Combs
- Selbsthilfe-Netzwerk Kopf-Hals-M.U.N.D-Krebs: Gunthard Kissinger

^†^ Prof. Dr. Wilko Weichert, Director of the Institute of Pathology at the Technical University of Munich, passed away far too early on July 10, 2023, after a serious illness at the age of 52. All of the pathology contributions in this guideline were largely written by him and coordinated with him.

The German Federal Association for Speech Therapy was also invited to participate in the guideline, but decided not to do so.

Doctors from the Competence Center for Oncology of the Medical Services were involved in the development of this S3 guideline in an advisory capacity for individual aspects with socio-medical relevance. They did not participate in the voting on the individual recommendations and are not responsible for the content of this guideline.

#### 1.4.3 Workgroups

Composition of guideline workgroups

- Workgroup: core editorial team
- Composition of Workgroup: **Prof. Dr. Wilfried Budach**, **Prof. Dr. Andreas Dietz**, Oliver Bayer, Prof. Dr. Orlando Guntinas-Lichius, Prof. Dr. Jochen Heß, Prof. Dr. Thomas Hoffmann, Prof. Dr. Jens Peter Klußmann, Prof. Dr. Stephan Lang, Prof. Dr. Georg Maschmeyer, Prof. Dr. Susanne Singer, Katherine Taylor, Prof. Dr. Wilko Weichert, Prof. Dr. Susanne Wiegand

Workgroup managers are marked in bold.

#### 1.4.4 Patient involvement

The guideline was drawn up with the direct involvement of 2 patient representative organizations.

Mr. Herbert Helmund and Mr. Gunthard Kissinger were involved in the creation of the guideline from the outset as elected representatives and took part in the consensus conferences with their own voting rights.

#### 1.4.5 Methodological support

By the German Guideline Program in Oncology:

- Dr. Markus Follmann, MPH MSc (DKG, GGPO)
- Dipl.-Soz.Wiss. Thomas Langer (DKG, GGPO)
- Dr. rer. medic. Susanne Blödt, MScPH (AWMF-IMWi)
- Dr. Monika Nothacker, MPH (AWMF-IMWi)

By the scientific staff of the Department of Epidemiology and Health Services Research, Mainz University Medical Center:

- Prof. Dr. Susanne Singer
- Katherine J. Taylor, MSc
- Oliver Bayer, MSc

### 1.5 Abbreviations used

- 5-FU: 5-Fluorouracil
- AJCC: American Joint Committee on Cancer
- ASCO: American Society of Clinical Oncology
- AWMF: Association of the Scientific Medical Societies in Germany
- BGB: German Civil Code (German: Bürgerliches Gesetzbuch)
- CI: Confidence interval
- CIN: Cervical intraepithelial neoplasia
- CPS: Combined positive score
- CT: Computed tomography
- ddPCR: Droplet digital polymerase chain reaction
- DFS: Disease-free survival
- DNA: Deoxyribonucleic acid
- EC: Expert Consensus
- ECE: Extracapsular extension (lymph nodes)
- ECOG: Eastern Cooperative Oncology Group
- EGFR: Epidermal growth factor receptor
- EHNS: European Head and Neck Society
- EORTC: European Organisation for Research and Treatment of Cancer
- ESMO: European Society of Medical Oncology
- EXTREME: Study title: The Erbitux in First-Line Treatment of Recurrent or Metastatic Head and Neck Cancer
- FEES: Fiberoptic Endoscopic Evaluation of Swallowing
- FFPE: Formalin-fixed, paraffin-embedded sample(s)
- FUT: Follow-up treatment
- GGPO: German Guideline Program in Oncology
- GoR: Grade of recommendation
- HPV: Human papilloma virus
- HPV16: Human papilloma virus, subtype 16
- HR: Hazard ratio
- ICD: International Classification of Diseases
- IMRT: Intensity modulated radiotherapy
- IQWiG: Institute for Quality and Efficiency in Health Care (Institut für Qualität und Wirtschaftlichkeit im Gesundheitswesen)
- LoE: Level of evidence
- mRNA: Messenger RNA
- NCCN: National Comprehensive Cancer Network
- NCDB: National Cancer Database (USA)
- ND: Neck dissection
- NGS: Next generation sequencing
- OPSCC: Oropharyngeal head and neck squamous cell carcinoma
- PCR: Polymerase chain reaction
- PD: Progressive disease
- PD1: Programmed cell death protein 1
- PEG: Percutaneous endoscopic gastrostomy
- PET: Positron emission tomography
- PflBG: Nursing Professions Act, German law (German: Pflegeberufegesetz)
- PFS: Progression-free survival
- PICO: Population, Intervention, Comparison, Outcome
- PPV: Positive Predictive Value
- RKI: Robert-Koch-Institut
- RNA: Ribonucleic acid
- RND: Radical neck dissection
- RR: Risk ratio (relative risk)
- RTOG: Radiation Therapy Oncology Group
- SEER: Surveillance, Epidemiology, and End Results (USA)
- SIN: Squamous intraepithelial neoplasia
- SNB: Sentinel node biopsy
- STIKO: Standing Committee on Vaccination of the Robert Koch Institute
- TLM: Transoral laser microsurgery
- TNM: System of classification for the anatomical spread of malign tumours with the primary tumour (T), regionalry lymph nodes (N), and distant metastases (M)
- TORS: Transoral robotic surgery
- TOS: Transoral surgery
- TPS: Tumour proportion score
- UICC: Union Internationale Contre le Cancer (eng.: Union for International Cancer Control)
- UICC: Union for International Cancer Control
- WHO: World Health Organization

## 2 Introduction

### 2.1 Scope and purpose

#### 2.1.1 Objective and key questions

This guideline was prepared as a joint guideline for oropharyngeal and hypopharyngeal carcinoma on the recommendation of the Steering Committee of the Oncology Guideline Program of 01/11/2017 and thus closes the gap between the existing S3 guidelines on squamous cell carcinoma of the larynx and oral cavity.

Over the last 20 years in Germany, it has been possible to enormously improve overall survival as well as long-term functionality for oropharyngeal and hypopharyngeal carcinoma by standardizing the surgical aims to be achieved (e.g. R0 resection with at least 5 mm distance to the tumour margin, further development of large-lumen defect reconstructions), categorizing neck dissection, linking postoperative therapy to clear risk criteria (e.g. extracapsular extension (ECE) of the loco-regional lymph node metastases) [TK1]), and diversification of radiotherapy techniques and drug-based tumour therapy with standardization of first-line therapy.

Depending on the respective tumour location, spread and biology, we are increasingly seeing differences in the surgical radicality to be selected, in the diversified multimodal overall concept and in the need for early rehabilitation measures in order to optimize overall survival in the context of acceptable late functionality by selecting suitable alternatives. The existing international evidence-based guidelines already contain useful, up-to-date systematic reviews, which are taken into account in this S3 guideline.

This S3 guideline is the final step in completing the S3 LL programme for squamous cell carcinoma of the upper aerodigestive tract in order to achieve a uniform approach with regard to the quality of care and standardization, which is reflected in the quality indicators of the certified centres (head and neck tumour centre according to DKG/Onkozert).

The guideline generally does justice to the interdisciplinary nature of early detection, diagnosis, therapy, rehabilitation and aftercare. The guideline provides reliable support in achieving the therapeutic goals and contributes to reducing the frequency of avoidable complications and improving the prognosis of treated patients. The guideline is intended to provide patients and their relatives with understandable and comprehensible information about the relevant treatment concepts and their effects. The additional patient version of the guideline supports informed, participatory decision-making.

Quality indicators are derived from the guideline, which are particularly helpful for interdisciplinary decision-making in tumour boards, e.g. in head and neck tumour centres (Onkozert), and for mapping the quality of outcomes. Doctors in private practice and general practitioners thus have a recommendation for action in the follow-up care of patients. The guideline also provides valuable information for those involved in functional rehabilitation (e.g. speech therapists) and psychosocial rehabilitation (psychologists, social workers, medical psychotherapists). The guideline leads to an improvement in the communication channels between the specialist groups involved in rehabilitation and the physicians involved in curative treatment and to a better understanding of the underlying disease.

#### 2.1.2 Target audience

The recommendations in this S3 guideline are aimed at:

- ENT physicians, oral and maxillofacial surgeons, phoniatrists, radiation oncologists, oncologists, pathologists, radiologists, palliative care physicians, radiotherapists, nuclear medicine specialists, virologists, physicians in rehabilitation facilities
- Nursing staff
- Speech therapists
- Psychologists, social workers
- Patient counselling organizations
- Self-help groups
- Cost bearers
- Patients

The guideline is also intended to provide information for general practitioners.

#### 2.1.3 Validity and update process

The S3 guideline is valid until the next update. The validity period is set at 5 years from the date of publication (February 2029). Regular updates are planned; if urgent changes are required, these will be published separately. Comments and suggestions for the updating process are expressly welcome and can be addressed to the guideline secretariat: pharynxkarzinom@leitlinienprogramm-onkologie.de

### 2.2 Methodology

The methodological procedure for the preparation of the guideline is described in the guideline report and in detail in the evidence report. Both documents are freely available on the Internet on the pages of the Guideline Program Oncology (https://www.leitlinienprogramm-onkologie.de/leitlinien/oro-und-hypopharynxkarzinom) and the pages of the AWMF (https://register.awmf.org/de/leitlinien/detail/017-082OL).

#### 2.2.1 Levels of evidence (LoE)

The assessment of the individual endpoints was performed separately by two members of the working group. According to the GRADE approach, the following aspects were assessed, resulting in an increase or decrease in confidence in the evidence (see further information on the GRADE approach at https://www.gradeworkinggroup.org/).

**Risk of bias:** A high risk of bias, or even just concerns about the risk of bias in one or more of the included studies, may reduce confidence in the evidence.

**Inconsistency:** Heterogeneity between studies that cannot be explained by subgroup analysis may reduce confidence in the evidence.

**Indirectness:** Differences between the original PICO question and the included studies in terms of population, intervention, comparison group or outcomes may reduce confidence in the evidence. In particular, when surrogate outcomes are used, transferability needs to be critically assessed and confidence in the evidence may need to be downgraded.

**Large effect:** If the effect found is large (e.g. RR either >2.0 or <0.5 based on consistent data from at least two studies), this may lead to an increase in confidence in the evidence.

Our confidence in the evidence was then expressed as one of four GRADE quality levels.

##### Scheme of evidence grading

Symbol, quality level and interpretation

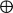

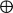

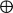

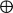
=high: The true effect is close to the estimated effect.
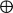

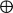

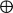

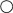
=moderate: The true effect is probably close to the estimated effect, but there is also the possibility that it is substantially different.
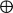

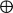

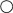

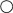
=minor: The true effect may differ significantly from the estimated effect.
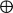

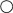

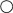

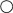
=very small: It is likely that the true effect differs significantly from the estimated effect.

We created a decision tree for the structured evaluation of the confidence level according to GRADE, which can be found in the evidence report in Section 2.6.

#### 2.2.2 Grades of recommendation (GoR)

The methodology of the oncology guideline programme provides for the assignment of recommendation grades by the guideline authors as part of a formal consensus process. Accordingly, a nominal group process or structured consensus conference moderated by the AWMF and DKG was conducted. As part of these processes, the recommendations were formally voted on by the mandate holders with voting rights. The results of the respective votes (consensus strength) are assigned to the recommendations according to the categories in the following list on consensus strength.

For all evidence-based statements (see Chapter 2.2.3) and recommendations, the evidence grading according to GRADE is shown in the guideline, as well as the strength of the recommendation (grade of recommendation) for recommendations. With regard to the strength of the recommendation, this guideline distinguishes between three grades of recommendation (see list below: Grading of recommendations), which are also reflected in the wording of the recommendations.

##### Recommendation grading scheme

Grade of recommendation – description – wording

- A – strong recommendation – shall
- B – recommendation – should
- 0 – open (optional) recommendation – can

##### Consensus strength

- Strong consensus: >95% of those eligible to vote
- Consensus: >75–95% of those eligible to vote
- Majority agreement: 50–75% of those eligible to vote
- No agreement: <50% of those eligible to vote

The decision criteria for determining the recommendation grades are explained in the guideline report for this guideline.

#### 2.2.3 Statements

Statements are declarations or explanations of specific facts or issues without a direct call for action. They are adopted as part of a formal consensus procedure in line with the procedure for recommendations and can be based either on study results or on expert opinions.

#### 2.2.4 Expert consensus (EC)

Statements/recommendations for which the guideline group decided to work on the basis of expert consensus are indicated as expert consensus. No systematic literature search was carried out for these recommendations (the studies cited in the background texts were selected by the experts involved). For recommendations based on expert consensus, no symbols or letters are used to indicate the strength of the recommendation and the quality of the evidence. The strength of the recommendation is determined solely by the wording used (should/should/can) according to the grading under “Recommendation grading scheme” in 2.2.2.

In this guideline, only 24% of the recommendations and statements were evidence-based. There are 43 evidence-based recommendations/statements compared to 136 consensus-based recommendations/statements. This low rate is due to the fact that the systematic evaluation of the evidence (PICO) was restricted to clinically important and potentially controversial issues due to limited resources. There is a high level of evidence for many of the recommendations agreed to by expert consensus, which is listed extensively in the background text, including the current state of the research. No additional de novo research of the known data situation was undertaken. These recommendations are uncontroversial and were agreed to in all cases with “strong consensus”. Where possible, evidence-based recommendations and statements from the two existing S3 head and neck guidelines [1], [2] were adapted/adopted.

#### 2.2.5 Independence and disclosure of possible conflicts of interest

All persons involved in the development of the guideline submitted a written declaration of any existing conflicts of interest via the AWMF online platform provided for this purpose at the beginning or at the latest during the guideline process. These were updated again before the first consensus conference.

Prof. Andreas Dietz and the DKG (Dr. med Markus Follmann, Gregor Wenzel) assessed the conflicts of interest of all those involved in guideline development. An overview of the conflicts of interest of the persons involved and the resulting consequences can be found in the guideline report.

Conflicts of interest (COIs) were dealt with in accordance with AWMF regulations: in order to ensure the greatest possible trustworthiness of the guideline recommendations, care was taken to ensure that the coordinators of the guideline project had only a few thematically relevant conflicts of interest. For this reason, two coordinators (Prof. Andreas Dietz and Prof. Wilfried Budach) were initially appointed, who were flanked by Prof. Georg Maschmeyer for drug-based tumour therapy expertise. The COIs of the two coordinators, which were present only for Chapters 9.2.1 and 9.2.2, were taken into account by their recorded abstention from voting.

Furthermore, it was ensured that members of the guideline group with minor conflicts of interest (e.g. receipt of third-party funding from industry for presentations or authorship) were not allowed to take on a leadership role, such as sole chairmanship of a working group or main responsibility for the preparation of evidence on a research question. A leadership role may be assumed if a second person is also involved in leading the working group without any conflict of interest.

Members of the guideline group with moderate conflicts of interest (advisory board or consultant activities and receipt of third-party funding from industry in a responsible position) may only participate in consensus building as advisory, non-voting experts.

Persons with high conflicts of interest (ownership interest) were not allowed to participate in the deliberations of the guideline group but could contribute their knowledge in the form of written comments if they wished.

The external, independent moderation of the formal consensus-building process as well as the interdisciplinary development of the guideline and its public/expert review in the consultation phase are further aspects that are intended to reduce undesirable influence from conflicts of interest and strengthen confidence in the recommendations made.

## 3 Anatomical classification of oropharynx and hypopharynx

**3.1 Consensus-based statement 2024**

*The anatomical classification of the oropharyngeal and hypopharyngeal regions is based on ICD-10-GM version 2022: The International Statistical Classification of Diseases and Related Health Problems, 10* *^th^* *Revision, German Modification (ICD-10-GM) is the official classification for the coding of diagnoses in outpatient and inpatient care in Germany. The 2022 version of the ICD-10-GM has been in use since January 1, 2022.*

- EC
- Strong consensus

In the clinical-epidemiological literature, it is more difficult to differentiate the hypopharynx and in particular the oropharynx from other parts of the head and neck region than many other cancers.

The oropharynx extends from the palatine tonsils, lingual tonsils, base of the tongue to the vallecula and the lingual epiglottis, soft palate (bordering the hard palate, which is considered part of the oral cavity), uvula and posterior pharyngeal wall. The plica pharyngoepiglottica is seen as the anatomical boundary between the oropharyngeal and hypopharyngeal side walls. The hypopharynx includes the lower part of the pharynx adjacent to the oropharynx, which extends from the hyoid bone to the cricoid cartilage. The hypopharynx is bounded inferiorly by the upper esophageal orifice. The anatomical classification is according to ICD-10-GM version 2022: The International Statistical Classification of Diseases and Related Health Problems, 10^th^ Revision, German Modification (ICD-10-GM) is the official classification for coding diagnoses in outpatient and inpatient care in Germany. The 2022 version of ICD-10-GM has been in use since January 1, 2022 (Table 1 (Tab. 1)).

### Oropharynx

In fact, oropharyngeal carcinomas mainly occur at the base of the tongue and in the palatine tonsils, i.e. the epithelia of Waldeyer’s pharyngeal ring. The oropharynx is bounded upwards by the transition line between the hard and soft palate. The soft palate together with the uvula is included. However, the posterior surface of the soft palate including the uvula is included in the anterior wall of the nasopharynx (C11.3). Laterally, the nasopharynx is separated from the oropharynx by an imaginary line at the upper edge of the palatine tonsil including the palatine arches. The posterior wall follows the same medial dividing line. The tongue is divided by the linea terminalis into the posterior third of the tongue base (oropharynx) and the anterior two thirds (oral cavity). The larynx begins on the upper edge of the epiglottis and includes the laryngeal part of the epiglottis (supraglottis). The oropharynx contains the lingual tonsil (tonsilla lingualis), the vallecula and the lingual epiglottis. The demarcation to the hypopharyngeal sides and posterior wall is made on an imaginary line at the level of the laryngeal entrance or the upper borders of the two piriform recesses.

When considering the classification of overlapping malignancies according to ICD-10, there is still some blurring in the demarcation to neighbouring regions. A very typical case of insufficient differentiation is “Tongue, unspecified” (C02.9), as the lower third of the tongue (the base of the tongue) belongs to the oropharynx, while the front two thirds belong to the oral cavity. The tonsil at the base of the tongue, as distinct from the base of the tongue, was still included in ICD-9, but has been subsumed in ICD.10 under the heading “Malignant neoplasm of other and unspecified parts of the tongue” under C02.4 (due to a lack of specific classification of the neoplasm). Kreimer et al. [3] undertook a systematic attempt to exclude unclear definitions on the basis of ICD codes and included a “mixed sites” category for this purpose: “oral cavity (C020-C023, C030-C050, C060-69), oropharynx (C019, C024, C051, C052, C090-C109), and larynx (C320-C329); The “mixed sites” category is proposed for overlapping lesions for better classification and comparability in scientific considerations (C028, C029, C058, C059, C140, C142, C148, C149)”. This concept has been incorporated into the BROADEN study, a multicentre study to investigate “HPV attributable fractions in multiple head and neck sites” [4].

### Hypopharynx

Three subsites are defined for the hypopharynx itself: [5]. The piriform sinus, which extends caudally on both sides of the aryepiglottic fold (plica aryepiglottica) to the esophageal orifice. The transition to the oesophagus is fluid and, due to the phylogenetic co-evolution of the hypopharynx and oesophagus, should be seen in a closer oncological context than the transition between the oropharynx and hypopharynx. Initial proteomic analyses have shown that molecular field carcinogenization often shows parallel development in the hypopharynx and oesophagus in the same individual [4]. The upper edge of the aryepiglottic fold forms the dividing line between the hypopharynx and the supraglottic part of the larynx. Approximately 60% of all hypopharyngeal carcinomas arise in the piriform sinus. The postcricoid region extends from the outer posterior wall of the larynx to the lower edge of the cricoid cartilage. Approximately 30% of hypopharyngeal carcinomas arise in this region. The posterior wall of the hypopharynx is the origin of around 10% of all hypopharyngeal carcinomas [5].

## 4 Epidemiology

Over the last 25 years, oropharyngeal carcinoma has emerged as the most rapidly increasing carcinoma in the head and neck region in Germany. In contrast, the incidence of hypopharyngeal carcinoma is stable to slightly declining. The chapter on epidemiology includes considerations of prevalence and incidence, as well as the risk factors that promote the disease.

### 4.1 Prevalence/incidence

**4.1 Consensus-based statement 2024**

*The estimated incidence of oropharyngeal carcinoma in Germany is 4–16/100,000 in men and 3–7/100,000 in women.*

*The average age is given as 61 years for men and 6* *6 ye* *ars for women (the proportion of women with the disease is approximately 20%).*

- EC
- Strong consensus

**4.2 Consensus-based statement 2024**

*The incidence of hypopharyngeal carcinoma in Germany is currently estimated at 2.3 (men) and 1.7 (women)/100,000 inhabitants. Overall, there has been a slight decline in the incidence in recent years.*

*The average age at diagnosis is 64 years for both sexes.*

- EC
- Strong consensus

The database of the Centre for Cancer Registry Data at the Robert Koch Institute (RKI) that can provide estimates of the incidence, prevalence and survival of cancer in Germany is based on the epidemiological state cancer registry data (mortality data are provided by the Federal Statistical Office). To date, cancer registries have only been set up at state levels nationwide after the implementation of the KFRG (Cancer Early Detection and Registry Act), and these do not yet report to the future national cancer registry (recently regulated by law: Amendment of the Federal Cancer Registry Data Act, BKRG, 2021: Act on the Consolidation of Cancer Registry Data from 2023) located at the RKI. Therefore a definitive statement on incidence and prevalence or mortality in Germany is currently not meaningfully possible. The prevalence data presented below should therefore be regarded as estimates.

#### Oropharyngeal carcinoma

In Germany, a total of around 9,450 men and 5,700 women are newly diagnosed with a tumour in the oral cavity or throat (C00–C14) every year. Among men, 3,340 cases are oropharyngeal carcinomas (tumours of the base of the tongue (C01), tonsils (C09) and oropharynx (C10)), which can be caused particularly frequently by a persistent HPV infection [6]. Due to the known causative connection with the causative HPV-16 infection, which was proven in 2010 at the latest [7], oropharyngeal carcinoma is now differentiated into two separate entities depending on HPV16 status [8]. Squamous cell carcinoma of the oropharynx is now the sixth most common form of cancer in men. In Germany, there are no reliable data on the incidence of oropharyngeal cancer and even less precise data on the separate consideration of HPV-16 or the HPV-associated surogatesurrogate parameter, the cell cycle component p16. The estimated incidence of oropharyngeal carcinoma in men is 4–16/100,000, in women 3–6/100,000 inhabitants; for tongue base and tonsil carcinomas, an increase in new cases is observed particularly in young adults [tonsil carcinomas in 2000, male: 2.4 new cases/100,000 inhabitants/year, 2019: 3.5, female: 0.6 to 1.4; tongue base male 1.3 to 2.0, female: 0.3 to 0.7; data from the Robert Koch Institute]. Overall, the incidence of cancer localizing into the base of the tongue/tonsil is increasing slightly according to rough estimates by the RKI Centre for Cancer Registry Data (available until 2019).

#### Hypopharyngeal carcinoma

An analysis of the incidence of hypopharyngeal carcinomas in Germany based on data from the Centre for Cancer Registry Data revealed 1,286 documented new hypopharyngeal carcinomas in Germany in 2015, with 1,045 cases assigned to diagnosis code C13 (hypopharyngeal carcinoma) and 241 to code C12 (carcinoma of the piriform recess). This corresponds to a total age-standardized incidence of 2.3 per 100,000 (piriform recessus/C13: 0.4/100,000, hypopharynx/C12: 1.9/100,000) [9]. The Rhineland-Palatinate Cancer Registry documented 59 (C12: 6; C13: 53) new cases in men and 11 (C12: 3; C13: 8) new cases in women in 2018, which corresponds to an overall incidence of hypopharyngeal carcinoma of 1.7/100,000. In the data published by the RKI, the tumours of the oral cavity and pharynx (C00-C14) are often combined, which makes detailed analyses of the incidence development of hypopharyngeal carcinomas difficult.

An analysis of the Thuringia Cancer Registry for the years 1996–2005 shows a significant increase in hypopharyngeal carcinomas from 2.4/100,000 to 4.4/100,000 [10], with the incidence in women increasing from 0.16/1,000,000 to 0.76/100,000. Data from the Munich Cancer Registry show an increase in the number of cases from 1998 (51 cases) to 2009 (113 cases) and then a continuous decline until 2020 (29 cases). This corresponds to an age-standardized incidence in men of 3.9/100,000 in 1998 and 0.9/100,000 in 2020, and an age-standardized incidence in women of 0.3/100,000 in 1998 and 0.1/100,000 in 2020 (Munich Cancer Registry).

Data from neighbouring countries show an increase in the age-standardized incidence in the Netherlands from 0.81/100,000 in 1989 to 0.95/100,000 in 2013, with the incidence in men falling continuously, while the incidence in women increased by 1.7% annually [11]. An analysis of the Danish Cancer Registry for the years 1980–2014 showed a significant increase in the age-adjusted incidence from 0.3 per 100,000 in 1980 to 1.1 per 100,000 in 2014, which corresponds to an increase of 4.1% per year [12]. The increase in incidence was similar for both sexes (4.0% for men, 4.3% for women).

### 4.2 Prevalence of HPV16 in oropharyngeal carcinoma

An increase in HPV-positive oropharyngeal cancer (OPSCC) has been shown worldwide [13]. The increase between 1995 and 2009 was between 1.3 and 3.3 cases per year per 100,000. Specific incidences or prevalences of HPV-positive tumours are not yet included in the German cancer registries, which is why the incidence of HPV-associated OPSCCs is often determined by the proportion of HPV/p16-positive carcinomas in relation to the total number of all OPSCCs. Initial systematic studies in Germany on the incidence of HPV-positive carcinomas in the 2000s show a rate of 40% for OPSCC and event 58% in tonsillar carcinoma [14]. In a large, multicentre international analysis of a total of 1,090 OPSCCs from the period 1990 to 2012, the rate of HPV-positive OPSCCs was between 19% and 25% depending on the detection method [15]. For Germany, the figures for HPV-positive tumours in the oropharynx were between 11.5% and 55% in the past decade, with an increase in the proportion of HPV-positive tumours already recorded from 2000 to 2010 [16]. Data from the Rhineland-Palatinate cancer registry showed a significant increase in OPSCCs in women from 2000 to 2009 [17]. In a monocentric study, the rate of HPV-positive OPSCCs was 28% between 2004 and 2006 and 59% between 2012 and 2013 [18]. Another study showed an increase from 21% to 53% of HPV-positive OPSCCs between 2000 and 2015 [19] Data from the Hessian Cancer Registry show an annual increase in the incidence of all OPSCCs of 0.8 cases/100,000 per year and an increase of 1/100,000 per year for HPV-associated OPSCCs. The increase mainly affects tumours of the tonsils and tongue base region. Using RKI data and data from the Hessian Cancer Registry in comparison with US data, a comparable significant increase in OPSCCs was shown. However, in Germany this affected both sexes, while in the USA it was mainly men [20]. In the USA, the incidence of HPV-positive OPSCC in 2017 was 12.5 cases per 100,000, with the highest increase in white men in the 65–69 year age group (4.24% annual increase, [21]). The proportion of HPV-associated carcinomas of all OPSCCs in the USA has been as high as 93% in recent years [22]. In Germany, the rate of HPV-positive OPSCCs is now around 45%. Compared to patients with HPV-negative OPSCC, patients with HPV-positive OPSCC in the USA have a lower median age (57 vs. 61 years). New findings from German studies, on the other hand, showed no difference in age and even a trend towards an advanced age at first diagnosis of HPV-positive tumours [22].

Overall, a clear increase in HPV-associated OPSCCs can therefore be observed in Germany. The trend of the increase is somewhat delayed compared to the USA, but equally pronounced. There are indications that the increase in women is higher than in the USA. Cancer registry data also show an increase, particularly in tonsil and tongue base carcinomas [23]. Based on RKI data, the incidence in Germany for oral cavity and pharynx (C00–C14) in 1999 was 2,560 new cases per year for women and 7,818 for men, whereas in 2018 it was 4,491 cases per year for women and 9,821 for men [6].

### 4.3 Risk factors

The main risk factors for the occurrence of oropharyngeal (HPV16/p16-negative) and hypopharyngeal carcinoma are chronic tobacco or alcohol abuse, and much less frequently other factors. Both tumour entities are therefore predominantly noxious-triggered.

#### 4.3.1 Epidemiological risk factors

**4.3 Consensus-based statement 2024**

*The main risk factors for the occurrence of oropharyngeal (HPV16/p16-negative) and hypopharyngeal carcinoma are chronic tobacco or alcohol abuse, much less frequently other factors. Both tumour entities are therefore predominantly noxious-triggered.*

- EC
- Strong consensus

The field of epidemiologically defined risk factors (except for HPV16-associated OPSCC) is still very limited for oropharyngeal and hypopharyngeal carcinoma. In addition to the above-mentioned factors of tobacco and alcohol, there are other factors, but these take a back seat [24], [25], [26]. Similarly, there are neither specific mutations nor molecular-histological subtypes, such as in breast cancer, that allow a prognostically differentiated classification. The risk profile for oropharyngeal and hypopharyngeal carcinomas identified in epidemiological studies is predominantly very similar to the risk factors for oral cavity carcinoma [1]. Chronic tobacco or alcohol abuse increases the risk of disease up to 6-fold, and a combination of both risk factors up to 30-fold. In addition to the consumption of tobacco or alcohol, an unbalanced diet, such as excessive consumption of meat or fried food, can also increase the risk of carcinoma developing in the oral cavity and throat region [27], [28]. Conversely, it has been shown that a balanced Mediterranean diet more than halves the risk of developing carcinoma in the throat, adjusted for nicotine consumption and BMI. The key protective elements of the Mediterranean diet are citrus fruits, vegetables (especially fresh tomatoes), olive oil and fish oils [29], [30].

A few reports make a connection with individual sectors or occupational groups. Tobacco and alcohol-adjusted case-control studies and cohort studies have consistently described an association between employment in the construction industry, among painters and varnishers and in metalworking occupations and the occurrence of throat cancer. The relative risks or standardized mortality rates range between 1.5 and 3. In individual studies, a correlation was also found for employees in the paper and rubber industry. The studies on the textile industry and the woodworking trades showed inconsistent results [31], [32], [33], [34]. It is therefore necessary to take a detailed occupational, nicotine and alcohol history in patients with throat carcinomas in order to determine the significance of occupational and non-occupational causation. Careful consideration of the various risk factors in individual cases will make it possible to identify patients with pharyngeal carcinomas in whom occupational exposure is likely to be an equally important partial cause of the disease. This requires cooperation between the attending physician and an occupational physician. A definitive compensable occupational disease for throat cancer (compared to larynx and paranasal sinuses) has not yet been defined in the Occupational Diseases Ordinance (BKV).

#### 4.3.2 Histological precursor versions

Malignant tumours of the oropharynx and hypopharynx are 95% squamous cell carcinomas. Most squamous cell carcinomas have different degrees of differentiation. Grading is carried out according to the worst differentiated part of the tumour. However, the grading anchored in the WHO only has very limited prognostic value [35]. The extent of keratinization is also considered to have little prognostic value [36]. Newer concepts of tumour grading of laryngeal carcinomas show a higher predictive value (see below).

The updated WHO classification has been available since 2017, in which a number of innovations and concepts have been included with regard to squamous cell carcinomas and their precursors. The etiologically defined classification is the main change in the new concept. HPV-associated and toxin-triggered squamous cell carcinomas are clearly differentiated as independent tumour entities, whereby HPV-associated oropharyngeal carcinomas are no longer graded according to the conventional scheme as before and have been given their own ICD number in the WHO classification (ICD-O 8085/3). A two-stage system (low-grade vs. high-grade dysplasia) has been proposed for the precursors of squamous cell carcinoma [37], [38].

##### Subtypes of squamous cell carcinoma

Squamous cell carcinomas (PECA) are divided into subtypes according to the WHO classification. In addition to PECAs with classic morphology, there are special forms such as verrucous PECA (also known as Ackermann’s tumour). This is highly differentiated (G1) and exhibits a so-called pushing border phenomenon (“displacing growth”). In addition, basaloid squamous cell carcinomas are explicitly differentiated (by definition: G3, high-grade). These have a worse prognosis than conventional squamous cell carcinomas and are usually advanced at the time of diagnosis. The histological hallmark is basaloid differentiation with tumour cells arranged in a pallisade-like pattern at the edge of the tumour nests. These tumours should not be confused with HPV-associated PECAs, which can also be associated with a basaloid and non-keratinizing morphology. Papillary and verrucous squamous cell carcinomas are prognostically more favourable due to their superficial growth, but are very rare tumours overall (1–4%). Verrucous squamous cell carcinoma can have focal infiltration foci and then behaves like a conventional squamous cell carcinoma. Spindle cell carcinoma (formerly: sarcomatoid carcinoma, carcinosarcoma) can arise de novo or after radiation from a conventional squamous cell carcinoma [35].

Grading is based on nuclear pleomorphism and architecture. HPV-associated carcinomas are not (or are no longer) graded, because the conventional morphology is often G3. In the future, further classifiers (formation of tumour buds, so-called budding or formation of tumour cell separation) could be used to assess the grading. Good prognostic accuracy has been reported [35], [37], [38].

##### Precancerous precursor lesions

Non-invasive precursor lesions of squamous cell carcinoma are referred to as epithelial dysplasia and, according to the 2005 WHO classification, synonymously as intraepithelial neoplasia (squamous intraepithelial neoplasia: SIN). They are classified as low-, moderate- and high-grade (SIN 1–3); an important criterion here is, among other things, the disruption of the epithelial architecture in the lower, middle or upper third. In the new nomenclature of intraepithelial neoplasia, no distinction is made between carcinoma in situ and high-grade intraepithelial neoplasia: high-grade intraepithelial neoplasia (SIN 3)=carcinoma in situ. The malignancy risk of low- and moderate-grade intraepithelial neoplasia (SIN 1 and SIN 2) is 11%, while that of high-grade intraepithelial neoplasia (SIN 3=carcinoma in situ) is 90%. It is not necessary for a lesion to pass through all stages of intraepithelial neoplasia to become a squamous cell carcinoma. Squamous cell carcinoma can arise from all grades of intraepithelial neoplasia and even in morphologically inconspicuous mucosa [36].

The nomenclature of intraepithelial lesions is subject to constant change (see other organs such as the cervix or intestine), with three or two-stage systems essentially being used: SIN I–III vs. low and high grade dysplasia [36]. It may be advisable to specify both terms (neoplasia and dysplasia) in the text of the findings.

In both previously published S3 guidelines on laryngeal carcinoma and oral cavity carcinoma, overview texts were prepared on histological precursor lesions as risk factors, which apply equally to oropharyngeal and hypopharyngeal carcinoma [1], [2].

#### 4.3.3 HPV16 in oropharyngeal carcinoma

**4.4 Consensus-based statement 2024**

*HPV-associated oropharyngeal carcinoma is a genetically diverse tumour entity that is distinct from HPV16-negative oropharyngeal carcinoma.*

- EC
- Strong consensus

**4.5 Consensus-based recommendation 2024**

*For a TNM-relevant assessment of the HPV-16 association of an HPV infection, p16 immunohistology should be performed.*

- EC
- Strong consensus

**4.6 Consensus-based statement 2024**

*5%–23% of p16-positive oropharyngeal carcinomas are HPV16-negative after verification by polymerase chain reaction (PCR) and in situ hybridization.*

- EC
- Strong consensus

**4.7 Consensus-based statement 2024**

*The HPV-16 virus plays an almost exclusive role in the genesis of HPV-associated oropharyngeal carcinomas.*

*The infection is mainly transmitted through sexual intercourse (genital, anal, oral).*

- EC
- Strong consensus

For oropharyngeal carcinoma (OPSCC), it is noticeable that the “classic” risk factors of tobacco/alcohol consumption have been overshadowed by the now prominent and reliably substantiated causal role of infection with human papillomavirus (predominantly high-risk subtype HPV16) (particularly for tonsil and tongue base carcinomas, which are the most rapidly increasing head and neck subsites). It is now assumed that HPV-associated OPSCC is a genetically diverse tumour subgroup distinct from HPV-negative oropharyngeal carcinomas [39], [40], [41], [42]. In particular, the early expressed (“early”, E) proteins E5, E6 and E7, which are encoded by the viral genome, contribute to this. The oncoprotein E7 binds and destabilizes the retinoblastoma tumour suppressor protein (pRb), releasing factors that are necessary for transcription, proliferation and cell cycle progression. As a by-product of this interaction, the protein p16^INK4A^ (hereafter p16) is highly expressed [43], [44]. In infected cells, E6 leads, among other things, to inactivation of p53 and thus to the prevention of cell cycle control, thereby increasing genetic instability. Tumours that are purely HPV-associated often show a histological phenotype reminiscent of a basaloid squamous cell carcinoma, but without belonging to the subgroup of basaloid squamous cell carcinomas in the narrower sense. HPV proteins also lead to “immune escape”, which makes chronic infection and thus possible malignant degeneration more likely [45], [46], although a single infection with HPV does not necessarily lead to malignant degeneration. E5 supports the expression of growth factors and epidermal growth factor receptor (EGFR) and thus increases cell proliferation. However, EGFR expression is usually reduced in HPV-positive tumours [47]. In HPV-driven tumours, the p53 wild type is usually found and no TP53 mutations, which are associated with tumour development by classical noxious agents.

Since the corresponding morphology is subject to a certain range of variation, histological classification alone is unreliable. The detection of HPV16 mRNA E6*I, a sequence coding for the neoplastic transformation-causing proteins E6 and E7, is currently regarded as the most reliable detection method for definitive HPV16 association; however, it is often difficult to implement in routine diagnostics [23], [48].

##### Importance of p16 as an HPV16 surrogate parameter

The most common method currently used to “detect” an HPV infection is p16 immunohistology (see Chapter 7 for specific diagnostics). If squamous cell carcinomas strongly express p16, this is indicative of an HPV association of OPSCC. However, up to 23% of p16-positive OPSCCs that are examined using polymerase chain reaction (PCR) and in situ hybridization are ultimately HPV-negative [49], [50], [51]. This applies in particular to sites (e.g. larynx) where HPV cancers are rather rare and for suboptimal material [52] as well as in the context of a non-HPV16 HPV association. The frequency of p16-positive and HPV-DNA-negative tumours is lower in regions with a high incidence of HPV-associated tumours. Despite this uncertainty, p16 is currently the simplest and cheapest method for indirect HPV16 detection and is therefore unanimously recommended by the AJCC and UICC TNM Committee. In routine clinical practice, p16 immunohistology followed by HPV DNA detection (PCR or in situ hybridization) has proven effective in identifying oropharyngeal carcinomas that are truly HPV-associated.

##### HPV16 transmission, “high risk sexual behavior”, geographical differences

HPV transmission occurs predominantly through skin and mucous membrane contact. It is currently assumed that HPV is primarily transmitted through sexual intercourse (genital, anal, oral), but for oncogenic and non-oncogenic HPV types, contact with public wet surfaces (toilets, door handles, public pools, etc.) is also likely. Sexual behaviour in particular is viewed differently as an infection risk factor in various epidemiological studies. In 2007, Maura Gillison’s group published (New England Journal of Medicine) the until recently undisputed observation that the HPV-16-associated risk of developing oropharyngeal carcinoma is particularly associated with high-risk sexual behaviour. From a total of >26 “self-reported” vaginal sex partners (high-risk sexual behaviour; HR-SB) over the entire lifespan, a highly significant association with the occurrence of oropharyngeal cancer was observed (odds ratio 3.1; CI 1.5–6.5), which correlated with the increasing number of partners. A comparable odds ratio was also calculated for the number of >6 “self-reported” oral sex partners [53]. Brenner et al. [54] essentially describe risky sexual behaviour (i.e. a higher number of sexual partners, same-sex sex, younger age at sexual debut) as risk factors for the occurrence of early HPV antibodies. In addition to the factors mentioned above, men between the ages of 51 and 60 were also described as a high-risk group in the USA [55]. In a recent publication from the Leipzig LIFE cohort (propensity score matching 112 oropharyngeal carcinoma patients with 303 controls from the normal population), the association with HR-SB could not be confirmed, at least in Germany (greater Leipzig area). No differences were seen in the self-reported number of lifetime vaginal and oral sex partners between the Leipzig propensity score-matched sample of oropharyngeal cancer patients and controls. A comparison of the Leipzig results with the above-mentioned study by D’Souza shows a significantly lower prevalence of HR-SB in control subjects and an even lower prevalence of HR-SB in oropharyngeal carcinoma cases. The consistent absence of HR-SB in the overwhelming majority of HPV-related oropharyngeal cancers is also presented in light of a lower frequency of HPV-related oropharyngeal cancers in the Leipzig cohort compared to observations in the US (35.1% versus 64%, corresponding to seropositivity for HPV16 E6 and/or E7 antibodies).

In fact, there is increasing evidence that does not allow a generalized transfer of epidemiological data from the USA to Europe, especially Germany, without a more differentiated view. We see these differences in the evaluation of HR-SB as a risk factor and in the consideration of the discordance of the value of p16 as a surrogate parameter for a genuine HPV16 involvement in the development of oropharyngeal carcinoma (for more details, see Chapter 6.2 [49]). The HPV type spectrum is also different: the spectrum in Europe is narrower and focussed on HPV16 compared to a broader spectrum in the USA, which also includes other types, such as HPV18/45, which we generally do not see in Europe.

The HPV-16 virus plays an almost exclusive role in the genesis of oropharyngeal carcinomas. Infection with the HPV viruses occurs in the basal cells of the squamous epithelium, and the lymphatic tissue in the tonsils (crypt epithelium) enables the viruses to gain access to the basal cells even without injury. In the other parts of the mucosa, infection is only possible in the case of erosion or minor trauma. Infections can heal or lead to a latent infection [23], [48], [56], [57].

*Critically, numerous authors in Germany point out that purely HPV16-associated oropharyngeal carcinoma, which is not triggered by noxious agents, is very rare in Germany. The vast majority of patients (not exactly quantifiable) have a mixture of p16 positivity and existing noxious agent exposure. In this respect, the boundaries between the two oropharyngeal carcinoma entities, which are supposedly different, are blurred.*

It is possible that the smoking habits of patients are a major factor responsible for the geographical impact on HPV prevalence rates since then. It appears to be well established that patients with HPV-positive OPSCC are predominantly non-smokers, particularly in the USA and Canada. Therefore, in countries with a comparatively small proportion of smokers, such as those regularly described for US cohorts, HPV16 prevalence is significantly higher than in regions with a higher proportion of smokers, such as Germany. For example, HPV16 prevalence in Sweden (the lowest proportion of smokers (7%) in Europe) is estimated at 70%. In Germany, where the proportion of smokers is 24%, HPV16 prevalence is estimated at only 40%. The described interaction between smoking habit and HPV status is not fully understood. However, smoking appears to have a protective effect against the cancer-causing HPV16 infection. Based on the results of the Kiel group around Hoffmann M and Quabius ES on more than 1,000 patients and supported by two US-American studies, the following hypothesis is currently being discussed: smoking leads to significantly increased “secretory leukocyte protease inhibitor” (SLPI, an antileukoproteinase) and AnxA2 (annexin A2) expression in mucosal tissue. SLPI, which is excessively elevated in smokers, binds to AnxA2, which in this combination prevents the binding of HPV, if present. The binding of HPV to AnxA2 is crucial for a successful HPV infection of the mucosal cells. Conversely, in non-smokers with significantly higher AnxA2 levels, HPV can bind more easily to unoccupied – non-SLPI-bound – AnxA2, making successful infection of the cells more likely [58]. The promoting influence of cannabis (marijuana) on the development of HPV16-positive OPSCC has also been proven (through p38 MAPK pathway activation) [59], [60].

## 5 Early detection, prevention

**5.1 Consensus-based recommendation 2024**

*Screening of the entire population for oropharyngeal or hypopharyngeal carcinoma should not be offered.*

- EC
- Strong consensus

**5.2 Consensus-based recommendation 2024**

*According to STIKO recommendations, boys and girls between the ages of 9 and 14 should be vaccinated against HPV. A booster vaccination is recommended up to the age of 17.*

- EC
- Strong consensus

**5.3 Consensus-based recommendation 2024**

*Prophylactic HPV vaccination with the aim of therapeutic benefit as part of the treatment of an existing HPV-associated oropharyngeal carcinoma should not be offered.*

- EC
- Strong consensus

The aspects of early detection and prevention that have been extensively described to date apply to both oropharyngeal and hypopharyngeal carcinoma. In both S3 guidelines published to date on laryngeal carcinoma and oral cavity carcinoma, comprehensive overview texts have been prepared on this topic, which apply equally to oropharyngeal and hypopharyngeal carcinoma [1], [2]. HPV-associated oropharyngeal carcinoma is a special case, which will be discussed in more detail below. The focus is on education and raising awareness of risk factors. In the case of HPV-associated oropharyngeal carcinoma, vaccination is an option.

### 5.1 General view

In general, it is not advisable to screen the entire population for oropharyngeal and hypopharyngeal cancer due to the rarity of the disease. Risk groups can be defined: men and women who regularly smoke heavily (more than 20 cigarettes/day for more than 20 years) and regularly consume large amounts of alcohol (12 grams of pure alcohol equivalent to 1/8 liter of wine for women and 24 grams of pure alcohol equivalent to ¼ liter of wine or ½ liter of beer for men) have an increased risk of developing oropharyngeal and hypopharyngeal cancer. The risk of cancer is super-additive in the presence of both risk factors [25]. Screening for pharyngeal cancer cannot be recommended at present, even in high-risk groups, as there is currently no evidence of effectiveness, i.e. a reduction in incidence and mortality (literature and further information on early detection techniques: [2]).

### 5.2 HPV vaccination

The Standing Committee on Vaccination (STIKO) recommends vaccination against HPV for boys aged 9 to 14 years. The recommendation was published together with the scientific justification for this decision in Epidemiological Bulletin 26/2018. The STIKO has recommended HPV vaccination for girls since 2007. The aim of HPV vaccination for girls and boys is to reduce the burden of disease caused by HPV-associated tumours. The results of the systematic review on the efficacy and safety of HPV vaccination in boys and men are presented in tabular form in the appendix (detailed scientific justification: [61], [62]).

According to the fact sheets of the STIKO and the current vaccination recommendations from the RKI, the following applies: HPV infects both women and men, often during the first sexual contact. HPV-related cervical cancer mainly affects younger women between the ages of 35 and 59. In men, HPV mainly causes tumours in the throat, genital and anal areas. Complete vaccination protection can only be achieved if there has been no persistent infection with the HPV types contained in the vaccine prior to vaccination. For this reason, the vaccination should ideally be carried out before the first sexual contact. In Germany, 6% of girls and 3% of boys stated that they were 14 years old or younger at the time of their first sexual intercourse, while 82% of 18-year-old girls and 69% of 18-year-old boys are sexually active. Even after their first sexual experience or first intercourse, unvaccinated girls or boys can and should still be vaccinated against HPV. Even if a possible persistent HPV infection has already occurred, the vaccination can still provide protection against the other HPV types contained in the vaccine. The earlier the vaccination is given, the better. Various studies from a number of countries have shown that the HPV vaccination has no influence on the sexual behaviour of vaccinated people. Compared to unvaccinated people, vaccinated girls or women in these studies did not have sexual intercourse with a greater number of partners earlier as a result of knowing about their HPV vaccination, nor did they consciously refrain from using condoms.

Since the vaccination was approved, more than 270 million doses have been administered worldwide. Both before and after approval, the safety of the HPV vaccination was investigated in various extensive studies. No serious side effects, i.e. side effects that have a lasting negative impact on health, were found in causal connection with the HPV vaccination. In particular, the studies showed no connection with autoimmune diseases or neurological complications. Side effects such as headaches, dizziness or fatigue are common and can also occur in a severe form. However, these are temporary and completely reversible. As with other vaccinations, anaphylaxis can occur in very rare cases (approximately 1.7 cases per 1 million vaccinations). The Paul Ehrlich Institute, which is responsible for the safety of vaccines in Germany, has published further information on its website (https://www.pei.de).

Based on the current vaccination rate (44.6%), model calculations show that HPV vaccination of girls could reduce the incidence of cervical cancer in Germany by more than half over the next 100 years (163,000 fewer cases). If a comparable vaccination level is achieved for boys, more than 76,000 additional cases of HPV-related cancer in women and men can be prevented. By vaccinating both sexes against HPV, women and men can also protect their respective partners against HPV-related cancers.

Two inactivated HPV vaccines are currently licensed in Germany: the bivalent HPV vaccine Cervarix^®^ and the nine-valent vaccine Gardasil^®^9. Gardasil^®^9 offers additional protection against HPV types that are responsible for around 90% of genital warts. Both vaccines are recommended for vaccination against HPV.

**Vaccination schedule**

- 9 to 14 years: 2 doses at least 5 months apart (3 doses are required if the interval is shorter)
- 15 years and older: Cervarix^®^: 0 – 1 – 6 months, Gardasil^®^9: 0 – 2 – 6 months

According to the current information for healthcare professionals, there are contraindications: Cervarix^®^ and Gardasil^®^9 should not be used in case of hypersensitivity to the active substances contained in the respective vaccine or other vaccine components mentioned in the information for healthcare professionals. In addition, persons with hypersensitivity should not receive another dose of Gardasil^®^9 after previous administration of Gardasil^®^9 or Gardasil^®^ (quadrivalent). In the case of an existing pregnancy, vaccination against HPV should be postponed.

No booster vaccination is currently recommended (RKI, HPV vaccination). In a systematic review conducted by the RKI in collaboration with the STIKO HPV working group in 2014 on the evidence for the duration of the protective effect of the HPV vaccination against types 16 and 18 in girls and women, there was no evidence of a decrease in vaccination protection over time. The data in the systematic review referred to 1 or 2 RCTs with a follow-up period of ≥5 years after basic immunization and the investigated outcomes including the incidence of HPV infections, persistent HPV infections and CIN II+ lesions. According to the GRADE methodology, the quality of the evidence was assessed as “very low”. Furthermore, one study showed that HPV 16 and HPV 18 antibody responses are higher after vaccination with the two- or four-valent vaccine over several years than after an immune response following natural infection. In addition, the HPV 16 and HPV 18 antibody responses increase again significantly if a booster vaccination is administered several years after completion of the basic immunization. It can be assumed that HPV vaccination in boys and men has a duration of protection comparable to that in girls and women [62].

In general, the HPV vaccination rate in Germany is too low. In Germany, the nationwide rate for a complete HPV vaccination series with two vaccine doses among 15-year-old girls at the end of 2019 was 47.2% and among 15-year-old boys 5.1% (RKI 2022). Intensive and effective vaccination programs are necessary.

#### Therapeutic benefit of vaccination after the occurrence of HPV-associated oropharyngeal cancer

Countrywide, some physicians vaccinate with inactivated vaccine after the occurrence of HPV-associated oropharyngeal carcinoma with reference to the individualized recommendations of this vaccination in cervical carcinoma. However, this vaccination for cervical carcinoma is also controversial and is sometimes only recommended to prevent recurrences of precursor lesions.

The S3-LL “Vaccination prevention of HPV-associated neoplasia” explicitly does not recommend this vaccination. Recommendation 09-10: Consensus-based recommendations state: “HPV vaccination with the aim of therapeutic benefit in the treatment of existing HPV-associated lesions should not be performed”, and “In HPV-vaccine-naïve women with cervical intraepithelial neoplasia (CIN), HPV vaccination may be considered before or after treatment of CIN with the aim of reducing the recurrence rate” [63], [64]. As there is no evidence to date for the possible effectiveness of vaccination with the same intention in manifest oropharyngeal carcinoma, **no vaccination recommendation** is made in analogy to the recommendation for cervical carcinoma [63].

### 5.3 HPV screening offers

Any biomarker used for the early detection of OPSCC, especially HPV16-driven OPSCCs in the normal population, would therefore have to have a specificity of approximately 100% in order not to generate many more false-positive than true-positive test results, the ratio of which can be expressed as the positive predictive value (PPV). Even with a sensitivity of 100% (all patients are identified as such), a test with 99% specificity generates one (n=1) false-positive test result in 100 individuals tested. If 100,000 tests were carried out, this would result in 1,000 positive test results, of which, however, a maximum of 10 can be true-positive due to the rarity of the disease (approximately 10/100,000), i.e. there would be approximately 100 times more false-positive than true-positive test results, or a PPV of 1%. Such a test is unacceptable due to the associated psychological stress for those tested false-positive, and not cost-effective due to the diagnostic follow-up costs of all those tested positive. These correlations and fundamental implications for the screening of OPSCC were presented and discussed in detail at [65].

In contrast to cervical carcinoma, the location of the primary HPV infection in the head and neck area is unknown and therefore cannot be specifically sampled. There are no described precursor lesions for HPV-OPSCC that would be clinically detectable and thus represent an endpoint for a screening procedure. The early detection of small tumours in asymptomatic patients with the aid of various HPV biomarkers therefore appears feasible at best, with the aim of improving treatment morbidity and thus post-therapeutic quality of life.

Brush cytologies of the palatine tonsils and the base of the tongue are poorly tolerated in awake individuals [66] and are not very sensitive due to poor access to the tonsillar crypts where the tumours often arise [67]. Mouth and throat rinse samples have been used in various studies for the detection of oral HPV infections [53], [55], [68], but even in incident HPV-positive OPSCC patients they only show a sensitivity of approximately 50% [69] or have proven to be insufficiently sensitive for use outside of studies [70]. In addition, there is little data to suggest that the development of HPV-positive OPSCC can be predicted by measuring oral HPV DNA [53].

Antibodies against early HPV16 proteins, in particular oncoprotein E6 (and to a lesser extent E2), on the other hand, are very sensitive (approximately 90%) and specific (approximately 99%) markers for HPV16-positive OPC at the time of diagnosis [71], and can be measured years to decades before diagnosis [3], [72], [73]. They are being actively investigated in various studies as early detection markers [74], [75]. It is important to differentiate between antibodies against viral proteins that are expressed early (“early”, E) or late (“late”, L) during the viral replication cycle, as the latter, in particular antibodies against the main capsid protein L1, also occur in natural HPV infection and HPV vaccination, and are therefore completely unsuitable for HPV-OPSCC prediction [76].

A “liquid biopsy”, i.e. the detection of cell-free or circulating HPV DNA (cfDNA, ctDNA) from peripheral blood or plasma using droplet digital PCR (ddPCR) or next generation sequencing (NGS), is very sensitive [77], but the prognostic value cannot yet be estimated well due to the currently insufficient data available [78].

Post-therapeutic follow-up represents a completely different area of application for HPV biomarkers. The prediction of treatment success or failure with the help of early HPV antibodies does not appear promising, as the kinetics of the antibody response are greatly delayed, i.e. even successful tumour treatment does not lead to complete seroreversion [79]. In contrast, HPV DNA detection in liquid biopsies appears to be very suitable as a tumour marker in tumour aftercare [80], [81]. However, the detection of HPV DNA in mouthwashes proved to be unsuitable for routine use due to its low sensitivity in a German feasibility study [70].

The prediction of HPV-positive OPSCC with the aid of antibodies against early HPV proteins therefore appears promising, although the feasibility has not been conclusively clarified due to the difficulties associated with screening for rare diseases described above. Robbins et al. [74] estimate that an HPV16 E6 seropositive 50-year-old man has a risk of 7.3% and 17.4% of developing HPV-positive OPSCC in the next 5 and 10 years respectively (age 60: 14.4% and 27.1%). The maximum risk for women is 5.5% for a 60-year-old woman over the next 10 years. However, these calculations are based on figures from the USA, where both the OPSCC incidence rate and the proportion of HPV-driven OPSCCs among all OPSCCs are significantly higher than in Germany. Outside of international clinical studies [82], HPV serology for the detection of antibodies against “early” viral proteins is not routinely available. Effective screening would also require a defined risk profile to narrow down risk groups. Brenner et al., D’Souza et al. and Giuliano et al. [53], [54], [55] describe risk factors for the occurrence of early HPV antibodies as essentially risky sexual behaviour in the USA, although this was not observed in cohorts from Germany (as described in more detail above) [83], [84]. The use of this information, which probably applies to the USA and Canada but may not be readily transferable to Germany, for an organized screening procedure therefore appears to be feasible/useful only to a limited extent, also in view of the rarity of the disease, so that general screening of both the population and special risk groups is not currently recommended.

## 6 Prognosis, predictors

**6.1 Consensus-based statement 2024**

*HPV16-associated oropharyngeal carcinomas have a better prognosis than HPV-negative ones.*

- EC
- Strong consensus

**6.2 Consensus-based statement 2024**

*The prognosis of oropharyngeal and hypopharyngeal carcinoma essentially depends on the localization, TNM classification (including status of extranodal extension of the cervical lymph node metastases) and R status. Furthermore, p16 positivity (only for oropharynx), differentiation and the presence of lymph vessel invasion (lymphangiosis carcinomatosa) are prognostically rele* *v* *a* *nt.*

- EC
- Strong consensus

### 6.1 Prognostic factors of oropharyngeal carcinoma depending on HPV16

For oropharyngeal carcinoma (OPSCC), it is noticeable that the “classic” risk factors of tobacco/alcohol consumption have been overshadowed by the now prominent and sufficiently substantiated causal role of infection with the human papillomavirus (predominantly high-risk subtype HPV-16), particularly for tonsil and tongue base carcinomas, which are the most rapidly increasing head and neck subsites). It is now assumed that HPV-associated OPSCC is a genetically diverse subgroup distinct from HPV-negative oropharyngeal carcinomas with a significantly better prognosis [39], [40], [41], [42], [85].

Retrospective analyses suggest that HPV-associated OPSCC responds significantly better to previous treatment concepts. This tumour group showed significantly better survival after primary surgical as well as radio- or radiochemotherapy or anti-EGFR treatment. HPV is not a predictor for a specific treatment regimen and the prognosis is comparable after primary surgery plus adjuvant treatment and primary radiochemotherapy in retrospective cohorts [86]. In the majority of studies, overall survival after 5 years averaged up to 80% for HPV-positive (or p16-positive) OPSCC and 30–35% for HPV-negative OPSCC [23].

The retrospective analyses of an American study published in 2010 (RTOG 0129), in which accelerated fractionated radiotherapy in combination with cisplatin was compared with standard fractionated radiotherapy with cisplatin, provided the key impetus for HPV16-dependent therapeutic considerations. Kian Ang (a highly respected radiotherapist from the M.D. Anderson Cancer Center USA, who was instrumental in establishing the clinical consideration of HPV16 and unfortunately died unexpectedly in 2013) [7] was able to develop the following trend-setting 3-tier score (*the good, the bad, the ugly*) depending on tobacco consumption, HPV-16 status, N-status and tumour size:

1. Low-risk tumours: HPV-16-positive, ≤10 pack years or >10 pack years + N0-N2a
2. Intermediate-risk tumours: HPV-16-positive + >10 Pack Years + N2b-N3 or HPV-16-negative + ≤10 Pack Years + T2-T3
3. High-risk tumours: all other HPV-negative

(UICC 7^th^ edition)

The HPV-16-positive never smokers differed from the HPV-16-negative heavy smokers in terms of 5-year survival by almost 50% [7] (note: the 7^th^ edition of the TNM classification was used here). However, the stratification proposed by Ang does not differentiate in a comparable way in all retrospective cohorts. In the case of HPV-positive tumours, low tobacco consumption was not relevant to prognosis in various studies [87].

In contrast, oropharyngeal (HPV-16/p16 negative) and hypopharyngeal carcinomas are squamous cell carcinomas of the upper aerodigestive tract, which have almost identical risk factors and often premalignant precursor lesions (for an overview, see Section 4.3.3). In analogy to laryngeal carcinoma, it can be postulated for oropharyngeal and hypopharyngeal carcinoma that the prognosis essentially depends on the localization, TNM classification and R status. Furthermore, differentiation and the presence of lymph vessel invasion (lymphangiosis carcinomatosa) are relevant to prognosis. Oropharyngeal carcinomas have better prognoses than hypopharyngeal carcinomas.

In both previously published S3 guidelines on laryngeal carcinoma and oral cavity carcinoma, overview texts were prepared on premalignant lesions, risk factors and prognostic factors, which apply equally to oropharyngeal and hypopharyngeal carcinoma (for an overview, see the S3 Guideline for Oral Cavity Carcinoma 007-100OL, 2021, Ch.7.4; for an overview, see S3 Guideline for Laryngeal Carcinoma 017-076OL; Ch.3.2.1; 4).

### 6.2 Importance of p16 as HPV16-Surogat parameter

The p16 protein is encoded by the CDKN2A gene, is a CDK (cyclin-dependent kinase) inhibitor and plays a central role in cell cycle regulation and cellular senescence [88], [89]. In noxious-associated head and neck tumours, p16 is often inhibited by methylation, mutations or deletions (Network CGA). In contrast, in HPV-associated OPSCC, tumour cells are usually characterized by strong p16 overexpression, which is caused by the activity of viral HPV oncoproteins, especially E7, and can be used as a surrogate parameter for this entity [42], [43], [44], [90], [91].

Therefore, p16 detection via immunohistochemical staining (p16-IHC) with FFPE tumour sections is recommended as a suitable surrogate parameter for HPV status in OPSCC in routine diagnostics according to the current edition of the TNM classification (AJCC 8^th^ edition) and the CAP (College of American Pathologists) and ASCO (American Society of Clinical Oncology) guidelines [91], [92], [93]. For positive detection, at least 70% of the tumour cells should show heterogeneous, nuclear and cytoplasmic staining with moderate to strong intensity. Comparative studies of IHC staining with commercial anti-p16 antibodies and FFPE tumour sections showed moderate differences in specificity, intensity and variability as assessed by independent observers [94], [95].

Patients with a p16+/HPV16- OPSCC have a significantly worse prognosis compared to p16+/HPV+ tumours [49], [96], [97], [98], [99], [100] and show a slightly better risk profile than HPV-/p16– tumours [49], [101]. In a recent pooled analysis of 13 studies across Europe (7,895 patients), Mehanna et al. showed that the 5-year overall survival for patients with p16+/HPV16+ status was 81.1% (95% CI 79.5–82.7), for p16–/HPV16–40.4% (38.6–42.4), for p16-/HPV16+ 53.2% (46.6–60.8) and for p16+/HPV16- 54.7% (49.2–60.9). Patients with discordant OPSCC (p16–/HPV+ or p16+/HPV–) had a significantly worse prognosis than patients with p16+/HPV+ OPSCC and a significantly better prognosis than patients with p16-/HPV16- OPSCC. In the collectives contributed from Germany (1,035 patients from Cologne, Giessen and Kiel), the proportion of p16–/HPV16+ was 1.4–13.1%, p16+/HPV– 2.7–9.4% [49]. Thus, additional HPV-specific detection via viral DNA or RNA in combination with p16-IHC appears to be urgently required for the inclusion of patients in clinical trials [92], [93]. When reporting the test result, it should be stated whether the detection was carried out using p16-IHC as a surrogate parameter (p16-positive) or an HPV-specific method (HPV-positive) [92].

Since the corresponding morphology is subject to a certain range of variation, histological classification alone is unreliable for reliable HPV detection in the narrower sense. The detection of HPV16 mRNA E6*I, a sequence coding for the neoplastic transformation-causing proteins E6 and E7, is currently regarded as the most reliable detection method for definitive HPV16 association; however, it is often not possible in routine practice (fresh material) [23]. In fact, relevant working groups report 15–29.7% of p16-positive OPSCCs that were ultimately HPV16-negative in the polymerase chain reaction (PCR) and in situ hybridization [49], [50], [51]. The discordance p16+/HPV16– was highest in oropharyngeal sublocations outside the tonsils and tongue base [49].

The detection of HPV-16 mRNA E6*I is therefore currently regarded as the most reliable detection method for the definitive HPV-16 association in oropharyngeal carcinomas.

The sensitivity and specificity of p16-IHC as a surrogate parameter for the presence of an actual HPV association has been investigated in numerous studies. A meta-analysis of 24 studies showed a sensitivity of 94% (95% CI: 91–97%) and a specificity of 83% (95% CI: 78–88%) for p16-IHC alone compared to the detection of viral E6/E7 transcripts as a reference [102]. A high sensitivity but moderate specificity in the diagnosis of HPV-positive OPSCC has been confirmed in more recent studies, where **the proportion of p16+/HPV16– OPSCC is between 4–29%** [92], [93], [96], [97], [98].

Holzinger et al. [103] were able to show that sensitivity and specificity can be significantly increased by combining p16 and pRb ICH, with increased p16 expression in combination with underexpression of pRb (78% sensitivity, 93% specificity, 78% PPV, 93% NPV). The vast majority of retrospective studies therefore emphasize p16 and establish the association with HPV-16 as equivalent despite the above-described uncertainty. Not least for this reason, therapeutic conclusions based on retrospective analyses should be weighed very carefully. In the 8^th^ edition of the TNM classification, however, only p16 is (currently still) required as a discriminator.

In addition to p16-IHC as a surrogate parameter, there are several established methods for the detection of HPV-positive OPSCC with tumour tissue. These are based on the detection of viral DNA or RNA via in situ hybridization (ISH) or PCR-based technologies using fresh tumour samples or tissue sections [98]. The detection of transcripts of the viral oncoproteins E6 and/or E7 is often used as a reference or gold standard for the detection of HPV-positive OPSCC with tumour tissue [104]. However, the detection of viral transcripts is technically more demanding and more expensive, which makes it difficult to implement in current routine diagnostics [93], [104]. Modern ddPCR (droplet digital PCR) and NGS (next generation sequencing) based technologies enable the non-invasive detection of circulating HPV DNA in blood or saliva [98]. Several prospective studies have shown a high concordance between HPV detection in blood and tumour tissue and demonstrate the potential of circulating HPV DNA in assessing treatment success and monitoring the risk of recurrence in primary HPV-positive OPSCC as well as recurrences and metastases [105], [106], [107], [108], [109], [110]. In a direct comparison with blood and saliva samples from 66 patients with HPV16-positive OPSCC, NGS technology showed the highest sensitivity in the detection of HPV DNA compared to ddPCR or quantitative RT-PCR. However, these data as well as the practical implementation in clinical routine need to be confirmed and verified in larger, standardized clinical studies [98], [106], [110].

The revision of the TNM classification for oropharyngeal carcinomas has been fundamentally revised, particularly on the basis of the ICON-S study [92], [111], [112], [113]. The 8^th^ edition of the TNM classification (UICC, AJCC) is currently available, in which a different, p16-associated approach with a reclassification of tumour stages and N-status has been introduced. The anatomical assignment and definition of the regions on which the TNM classification is based was explained in detail in Chapter 3.

Therefore, if an OPSCC is p16-positive, not only pT1N0M0 tumours are subsumed under stage I, but now also pT1.2 pN0.1 tumours. Stage II even allows pT1.2 pN2 and pT3 pN0.1 tumours, compared to only pT2N0 previously; stage III includes pT3 pN2 and pT4 pN0-2 (8^th^ edition TNM 2017). The staging of p16-positive OPSCC is differentiated clinically and pathologically. cN2 is stage II and pN2 is stage III. It should also be noted that the N category is categorized according to the number of positive lymph nodes (pN1: 1–4; pN2: ≥5) and thus differs from all other squamous cell carcinomas and a tumour-perforated lymph node capsule (extracapsular extension, ECS) [114] is no longer taken into account. Several authors complain about the resulting purely classificatory “down-staging”, which was justified and enforced in the 7^th^ edition of the TNM classification on the basis of the poorer stage discrimination of p16-positive oropharyngeal carcinomas that had previously only been observed retrospectively. Furthermore, clinically and pathologically different TN stages were introduced. The changes were therefore made with a view to purely prognostic significance. When considering tumour stages, great care must therefore be taken not to fall into the danger of “under treatment”, especially in the case of parallel tobacco, alcohol and HPV associations [115], [116], [117]. The TNM classification of hypopharyngeal carcinoma has not changed since the 7^th^ edition.

### 6.3 Prognostic factors of oropharyngeal and hypopharyngeal carcinoma (HPV16-negative)

Reference: In both previously published S3 guidelines on laryngeal carcinoma and oral cavity carcinoma, overview texts were prepared on premalignant lesions, risk factors and prognostic factors, which apply to the same extent to oropharyngeal and hypopharyngeal carcinoma (for an overview, see the S3 Guideline for Oral Cavity Carcinoma 007-100OL, 2021, Ch.7.4; for an overview, see the S3 Guideline for Laryngeal Carcinoma 017-076OL; Ch.3.2.1; 4).

### 6.4 Definition of anatomical region, TNM

#### 6.4.1 TNM oropharyngeal carcinoma, discrimination according to p16

See Table 2 (Tab. 2), Table 3 (Tab. 3) and Table 4 (Tab. 4).

**6.3 Consensus-based recommendation 2024**

*The tumour stages according to the TNM classification and the R status are the most important prognostic factors in oropharyngeal and hypopharyngeal carcinoma and should be indicated in all cases with reference to the current 8*th* edition.*

*Additional parameters on the primary tumour should be recorded: Lymphatic vessel, venous and perineural sheath invasion and degree of differentiation.*

- EC
- Strong consensus

UICC stage for **p16-positive** carcinomas based on the clinical and pathological T-/N-stage (TNM 8^th^ edition; [112])

**T-status primary tumour** (clinically and pathologically identical)

TX: Primary tumour cannot be determined

Tis: Carcinoma in situ

T1: Tumour ≤2 cm in greatest extent

T2: Tumour >2 cm and ≤4 cm

T3: Tumour >4 cm, or tumour extension to the lingual surface of the epiglottis

T4: Moderately or very advanced

T4a: Moderately advanced disease with infiltration: larynx (further than lingual epiglottis surface), outer tongue muscles, medial pterygoid muscles, hard palate, mandible

T4b: Far advanced disease with envelopment of the carotid (communis or interna) or infiltration of: lateral pterygoid muscles, processus pterygoideus, lateral nasopharynx, skull base

**Clinical cervical lymph node status (cN)**

Clinical criteria apply to non-surgically treated patients without cervical lymph node dissection (neck dissection). Clinical assessment summarizes information from sources such as physical examination, imaging and fine needle aspiration.

NX: Nodal status cannot be determined

N0: No regionally enlarged or metastasized lymph nodes

N1: Metastasis in single ipsilateral node, ≤3 cm, and no extranodal spread, ECS(–)

N2

N2a: Metastasis in single ipsilateral node, >3 cm and ≤6 cm, and ECS(–)

N2b: Metastasis in multiple ipsilateral nodes, all ≤6 cm, and ECS(–)

N2c: Metastases in bilateral or contralateral nodes, all ≤6 cm, and ECS(–)

N3

N3a: Metastases in one node, >6 cm, and ECS(–)

N3b: Metastases in a node with clinically obvious ECS(+) (ECSc)

**Pathological cervical lymph node status (pN)**

Pathologic criteria apply to surgically treated patients with cervical lymph node dissection where multiple whole lymph nodes are available for microscopic evaluation.

NX: Nodal status cannot be determined

N0: No metastases in the examined lymph nodes

N1: Metastasis in single ipsilateral node, ≤3 cm, and no extranodal spread, ECS(–)

N2

N2a: Metastasis in single ipsilateral node, >3 cm and ≤6 cm, and ECS(–); or metastasis in single ipsilateral node, ≤3 cm, and ECS(+)

N2b: Metastasis in multiple ipsilateral nodes, all ≤6 cm, and ECS(–)

N2c: Metastases in bilateral or contralateral nodes, all ≤6 cm, and ECS(–)

N3

N3a: Metastases in one node, >6 cm, and ECS(–)

N3b: Metastasis in single ipsilateral node, >3 cm, and ECS(+); or multiple ipsilateral, contralateral, or bilateral nodes with ECS(+); or single contralateral node of any size and ECS(+)

**Distant metastases (M)**

The terms pM0 and MX are not valid TNM categories. The following categories can be used:

cM0: No evidence of distant metastases

cM1: High probability of distant metastases present (high evidence in imaging)

pM1: Existing distant metastases, histologically confirmed

(TNM 8^th^ edition [112])

#### 6.4.2 TNM Hypopharyngeal carcinoma

See Table 5 (Tab. 5).

**T-status primary tumour**

TX: Primary tumour cannot be determined

Tis: Carcinoma in situ

T1: Tumour limited to a subunit of the hypopharynx (left or right pyriform sinus, posterior wall of the hypopharynx or postcricoid region) and/or tumour ≤2 cm in greatest extension

T2: Tumour extends into the adjacent subunits of the hypopharynx or the adjacent neighbouring structures (larynx, oropharynx) and/or tumour >2 cm and ≤4 cm without fixation of the hemilarynx

T3: Tumour >4 cm, or clinical fixation of the hemilarynx, or extension into the oesophageal mucosa

T4: Moderately or very advanced disease

T4a: Moderately advanced disease with infiltration: thyroid cartilage, cricoid cartilage of the larynx, hyoid bone, thyroid gland, oesophageal muscles, prelaryngeal soft tissue (muscles, subcutis)

T4b: Far advanced disease with envelopment of the carotid artery (com. or interna) or infiltration of mediastinum, prevertebral fascia

**cN, pN, M analogous to oropharynx (see above)**

(TNM 8^th^ edition, no change compared to 7^th^ edition TNM classification)

**R classification (generally applies to squamous cell carcinomas in the head and neck region, i.e. equally for oropharyngeal and hypopharyngeal carcinomas)**

The categories of the R classification are clearly defined. R does not belong to the obligatory TNM classification, but describes the presence of residual tumour after therapy, usually after surgical therapy. As the use of the R classification provides important information on any further treatment that may be necessary and on the patient’s prognosis, and as this is called for in the S3 guidelines, some principles are outlined below. Carcinomas that have been resected in sano are defined as R0; the exact distance between the tumour margins and the resection margin is irrelevant as long as the immediate margin is tumour-free. The minimum distance of the tumour bandages to the resection margin should be specified exactly for all relevant resection margins (in mm/cm, see below). An R1 situation is present if the tumour microscopically reaches one of the preparation margins directly, and an R2 situation is present if the tumour remains in the patient macroscopically (this also applies, for example, in the case of a primary tumour operated on in sano with clinically known non-surgically treated metastasis). An RX situation exists if the resection margin cannot be reliably assessed histomorphologically (e.g. in the case of highly fragmented material) (for an overview, see the S3 Guideline on Laryngeal Carcinoma 017-076OL; Section 4.3).

### 6.5 Histopathology report

**6.4 Consensus-based recommendation 2024**

*The following parameters should be indicated in the histopathology report:*

*– Tumour location and size*

*– pTN status*

*– Histological tumour type according to the current WHO classification*

*– Local tumour extent, infiltrated structures*

*– Number of LKs examined*

*– Number of affected LKs*

*– Largest diameter of the lymph node metastases*

*– Tumour growth beyond the capsule (ECS, ENE)*

*– Lymph vessel/venous invasion and perineural invasion*

*– Presence of an in situ component (with size)*

*– Differentiation of the tumour according to the established grading scheme*

*– Distance to the lateral and basal resectate margins for all relevant resection margins as well as for the invasive and in situ components in mm.*

*– R classification*

*Oropharynx only: Indication of p16 expression status (positive, negative). For positive detection, at least 70% of the tumour cells should show heterogeneous, nuclear and cytoplasmic staining with moderate to strong intensity.*

- EC
- Strong consensus

**6.5 Consensus-based recommendation 2024**

*Tumour resection should be performed as an en bloc resection of the primary tumour. If a safe en-bloc resection is not possible (“piecemeal” technique in the context of transoral surgery, TLM or TORS), it is suitable to send separate marginal sections, which should also be sent to the pathologists as frozen sections.*

*In this case, the tumour fragments should be marked and arranged in relation to each other according to the resection. The transtumoural section margins should be marked separately from the outer areas relevant as margin sections. The subsequent frozen sections should be clearly assigned to the resection margins.*

- EC
- Strong consensus

The histopathological report has already been described in detail in the S3 guideline on laryngeal carcinoma and is only supplemented by the p16 findings in this guideline [2].

The pTNM classification as the basis for the pathological assessment of resectates has already been explained above. The treatment decision is also influenced by the following parameters, some of which are also associated with or depicted in pTNM:

- Tumour location and size,
- Histological tumour type according to the current WHO classification [37], [38], [118],
- Local tumour extent, infiltrated structures,
- Lymph node metastases separated according to level (see neck levels according to Robbins [119]) and side:
  - Number of lymph nodes examined,
  - Number of affected lymph nodes,
  - Largest diameter of the lymph node metastases,
  - Tumour growth beyond the capsule,
  - Lymph vessel/venous invasion and perineural invasion,
- Presence of an in situ component (with size),
- Differentiation of the tumour according to the established grading scheme [120],
- Distance to the lateral and basal resectate margins for all relevant resection margins and for the invasive and in situ components in mm.
- R classification
- Indication of the p16 expression status (positive, negative). For positive detection, at least 70% of the tumour cells should show heterogeneous, nuclear and cytoplasmic staining with moderate to strong intensity [91], [92], [93].

For biopsies, samples should be taken from the periphery of the tumour and, if possible, centrally from the base of the tumour. Resectates for the pathologist should be provided with topographically clear information regarding the anatomical orientation and exact localization.

The corresponding parameters mentioned above must be precisely stated in the histopathological description of the findings (where applicable). For biopsies, the number of parameters is naturally reduced.

**Biopsies:**

Clinically, biopsies of a manifest macroscopically clear neoplasm should be taken from the periphery of the tumour and, if possible, centrally from the base of the tumour (see also below). The examination order to the pathologist must contain all clinically relevant information. If the findings are unclear, the biopsy should be repeated after consultation with the pathologist.

**Resectates:**

In the case of resectates, the tumour specimen should be sent to the pathologist by the surgeon with a clear description of the anatomical topography (suture or color marking, needle marking, clock dial diagram) (BDP2017). In case of doubt, a personal consultation should take place. In the case of a neck dissection, the levels must be marked separately or sent in portions as individual specimens for pathohistological examination. The cut edges on the resectate can be examined by means of a frozen section according to clinical relevance, so that resection can be carried out in the same session in the event of tumour involvement. It is preferable to send the resectate “en bloc” and have the surgeon or pathologist take a frozen section of the resectate margins. Areas with specific clinical questions should be marked separately. This procedure preserves the integrity of the specimen in the best possible way and allows the most reliable statement on the R status, as small sections do not have to be virtually adapted to each other retrospectively.

*Transoral robotic surgery (TORS) or laser microsurgery (TLM) resections of primary tumours of the oropharynx and hypopharynx are increasingly used approaches for cancer resection in selected patients with accessible tumours. Oncologic principles are similar to open proce* *d* *u* *res. Therefore, if safe en bloc resection is not possible (“piecemeal” technique in the context of transoral surgery, TLM or TORS), it is appropriate to send separate margin sections, which should ideally also be sent to the pathologists as frozen sections. In this case, the tumour fragments should be marked on cork and arranged in relation to each other according to the resection. The transtumoural section margins should be marked sep* *a* *r* *at* *ely from the outer areas relevant as margin sections. The subsequent frozen sections should be clearly assigned to the resection margins. For this purpose, it is advisable to invite the pathologist to the operating theatre and agree on a documentation concept together. This makes it possible to determine a final R status even after piecemeal surgery. The previous refusal in principle of some pathologists to give an R status after piecemeal surgery, who then classified RX, should be overcome in the context of good interdisciplinarity.*

**Cervical lymph nodes:**

Locoregional metastasis of the primary tumour to the cervical lymph nodes is a reliable negative predictor of prognosis, with the more lymph nodes involved, the less favourable the course of the disease. Furthermore, involvement of the caudal levels (IV and V) and growth beyond the capsule have a negative impact on the prognosis [121], [122], [123], [124], [125], [126], [127], [128]. It is preferable for the surgeon to portion the levels, as exact ex-situ allocation in pathology is only possible to a very limited extent, even when markings are applied. The dissection of lymph nodes (especially those with tumour involvement) should be avoided. The lymph nodes should be assigned to the level at which the largest lymph node diameter is visualized. Structures of particular interest should be marked separately.

The histopathological findings of the neck dissection specimens should include the side of the neck, the cleared levels, the total number of lymph nodes with the number of affected lymph nodes per level, the diameter of the largest lymph node metastasis, additionally removed structures and – if present – information on growth that exceeds the lymph node capsule (extra nodal extension, ENE). *As the current discussion on the different extensions of ENE has not yet resulted in any clinical implications, this aspect is currently not addressed further in this guideline.* The detection of isolated tumour cells in lymph nodes or of micrometastases, which can only be detected using immunohistology, is currently still of unclear clinical relevance [129].

For further explanations regarding the histopathological assessment of oropharyngeal and hypopharyngeal carcinomas, please refer to the current S1 guideline on the pathological anatomical diagnosis of squamous cell carcinomas of the head and neck, 1^st^ edition (BDP2017).

### 6.6 Surgical safety distance

**6.6 Consensus-based statement 2024**

*A clear margin is defined as the distance from the inva* *s* *i* *ve tumour front that is 5 mm or more from the re* *se* *c* *t* *ed margin.*

- EC
- Strong consensus

**6.7 Consensus-based recommendation 2024**

*In the case of R1 resection, a two-stage resection should be* *performed if possible in order to achieve a final R0 resection.*

- EC
- Strong consensus

An overriding goal of oncological surgery is complete tumour resection with histological evidence of tumour-free margins. The margins can be assessed in real time intraoperatively by frozen section or by assessing formalin-fixed tissue. Tumour-free margins are a basic principle of surgical strategy to reduce the risk of local tumour recurrence. Conversely, positive margins increase the risk of local recurrence and are an indication for postoperative adjuvant therapy [130]. Classic clinical-pathological studies with unchanged current relevance have shown the significance of narrow or positive margins and their correlation with local tumour recurrence [131]. Other authors only observe a negative influence on freedom from local recurrence with R1, but not with any safety margins in mm from the tumour border in the case of an R0 resection [130]. The removal of frozen sections is generally recommended. In a recent multicentre Italian study (450 patients, 10 centres), it was even shown that in patients in whom R0 resection was verified intraoperatively by frozen sections, the local control rate was twice as high as in those without frozen section verification. This is a surprising, unexpected and not easily explained result. The authors suggest that frozen section assessment is a quality indicator of surgical and pathological procedures and their effectiveness [130]. If there is an initial incision through an invasive tumour at the surgical margin, additional harvested adjacent margins from the patient (i.e. not from the excised tumour) may mean a higher risk of local recurrence and should therefore be described in the surgical report. When obtaining additional margins from the patient, there may be confusion as to whether the tissue removed from the surgical bed corresponds to the actual location of the positive margins [132]. If positive surgical margins are reported, a repeat resection should be considered if possible or, if not reasonably possible, adjuvant therapy should be considered. The patient’s prognosis is not worsened by an initial R1, or close R0 situation, which leads to a final stable R0 situation through resection at the correct site [130], [133], [134].

Assessment of frozen section margins is always at the surgeon’s discretion and should be considered if it facilitates complete removal of the tumour. Achieving sufficiently wide margins may require resection of an adjacent structure in the oral cavity or laryngopharynx, such as the base and/or anterior tongue, mandible, larynx or part of the cervical oesophagus.

Adequate resection is defined by current international consensus as clean resection margins with at least sufficient distance from the gross tumour to obtain a clear frozen section and permanently free margins (can often mean 1.0–1.5 cm of visible and palpable normal mucosa). In general, a frozen section examination of the margins is usually performed intraoperatively and when a resection line is not clear due to indistinct tumour margins or when residual disease is suspected (i.e., soft tissue, cartilage, carotid artery or mucosal irregularities). For transoral endoscopic and robotic approaches for oropharyngeal and hypopharyngeal cancers, margins of 1.5–2.0 mm may be acceptable, but data are based on retrospective studies, so caution is advised [135]. Such margins would in principle be considered “narrow or closed” and are considered too narrow for certain sites, such as the base of the tongue.

- **A clear resection margin is defined as the distance from the invasive tumour front that is 5 mm or more from the resected margin.** There is international agreement on this, which is reflected in the current NCCN guidelines [136]. For hypopharyngeal carcinomas with diffuse, partially submucosal spread, particularly towards the oesophagus, the 5 mm safety margin should be regarded as the absolute minimum and can be generously extended to 1 cm for safe resection according to international consensus.
- A narrow margin is defined as the distance from the invasive tumour front to the resected margin that is less than 2–5 mm depending on the anatomical site affected.
- A positive margin is defined as carcinoma in situ or invasive carcinoma at the resected margin. If carcinoma in situ is present and if additional clean margins can be achieved by resection, this is the preferred approach. Carcinoma in situ should not be considered an indication for concomitant postoperative systemic therapy/RT.

If the surgeon takes additional resection margins from the patient, the new margins should relate to the geometric orientation of the resected tumour specimen with a statement from the pathologist that this will be the final resection margin and its histologic status in the final tender (NCCN Guideline Version 2.2022, SURG-A 4 of 8).

The unfavourable prognostic impact of pretreatment (especially surgical pretreatment) for local control regardless of R0 resection in the context of salvage surgery has been pointed out [130]. Tumour grading (often criticized as inconsistent and subjective) also appears to have a greater prognostic role than previously assumed. In the aforementioned multicentre Italian study, the group of well-differentiated squamous cell carcinomas showed a statistically significant and clinically relevant difference in 5-year tumour relapse-free survival and OS compared to the group of poorly differentiated squamous cell carcinomas, depending on localization and independent of R0 resection [130].

## 7 Clinical diagnostics

**7.1 Consensus-based recommendation 2024**

*Immediate referral to an appropriate specialist should be made for any of the following findings if they persist for more than four weeks:*

*– Blood in the saliva*

*– Hoarseness*

*– Difficulty speaking and breathing*

*– Persistent foreign body sensation, especially on one side*

*– Pain radiating into the ear*

*– Dysphagia and/or pain when swallowing*

*– Unclear coughing up blood*

*– Swelling of the throat*

*– Foetor ex ore*

- EC
- Strong consensus

This chapter is carried out with minor specifications analogous to the S3 guideline on laryngeal carcinoma 017-076OL, section 6.1; S3 guideline on oral cavity carcinoma 007-100OL, version 3.4, section 5.1 [1], [2].

Any change in swallowing with a foreign body sensation is suspicious for the presence of squamous cell carcinoma. All areas of the oropharynx and hypopharynx can be affected and lead to different symptoms. As the hypopharyngeal and parts of the oropharyngeal mucosa are not accessible to direct inspection, visible tissue changes that are prominent in other localizations, such as the anterior oral cavity, cannot be detected directly by the patient or the doctor. Appropriate endoscopic/mirror examinations must be performed. The symptoms vary greatly depending on the location of the carcinoma. Hypopharyngeal and tongue base carcinomas are characterized by difficulty swallowing, a lumpy sensation and swallowing pain (radiating into the ear). Changes in voice and hoarseness may also occur, and in the later stages, breathing may also be impaired. If the above-mentioned symptoms persist for more than four weeks, a specialist should be consulted.

Initially, there may be swelling of the lymph nodes in the neck, which can be confused with inflammatory diseases such as pharyngo-laryngitis or lymphadenitis. Progressive tumour growth leads to increasing functional impairments such as considerable voice and breathing disorders as well as difficulties in swallowing and nutritional disorders with weight loss. Severe pain develops, radiating to the entire head and neck region. In up to 40% of patients, the cervical lymph nodes may already be affected at the time of initial diagnosis despite a clinically unremarkable neck. Immediate referral to a specialist should be made for the following findings if they persist for more than three weeks:

- Blood in the saliva
- Hoarseness
- Difficulty speaking and breathing
- Persistent foreign body sensation, especially on one side
- Pain radiating into the ear
- Dysphagia and/or pain when swallowing
- Unclear coughing up blood
- Swelling of the throat
- Foetor ex ore

More intensive education of the population and accelerated referral of patients with unclear findings and special risk constellations to specialists are necessary in order to shorten the time interval from the first symptom to the start of tumour-specific treatment (Make Sense Campaign of the IAG-KHT, EHNS).

### 7.1 Clinical examination

The clinical examination for suspected oropharyngeal or hypopharyngeal carcinoma includes specific ENT examinations following a general medical history, full body examination and recording of relevant comorbidities: this involves endoscopy of the oral cavity, oro- and hypopharynx and larynx in the phonation and respiratory position. A 90° magnifying laryngoscope should be used for this purpose, whereby surface anaesthesia of the pharynx usually improves the examination options. Many patients with malignancies of the head and neck have a considerable gag reflex, which makes indirect examination of the larynx, deep oropharynx and hypopharyngeal entrance via the oral cavity difficult or even impossible. In such cases, only a flexible endoscopic examination with a 4 mm flexible rhino-pharyngo-laryngoscope is possible, which is advanced via the lower nasal passage and the pharynx to the larynx after local surface anaesthesia. In addition to the assessment of pathological mucosal changes, particular attention must be paid to vocal fold mobility. A phoniatric examination to assess the vibration behaviour of the vocal folds, particularly in hypopharyngeal carcinomas with incipient vocal fold fixation with the aid of a stroboscope, is recommended.

Systemic tumour seeding, especially in the lungs, is also possible. If several regions of the pharynx and larynx are affected at the same time, this is referred to as multilocular tumour growth. For this reason and due to the possible presence of synchronous secondary carcinomas in the pharynx or larynx, endoscopy of the neighbouring mucosal areas, e.g. with a 90° loupe laryngoscope or with a flexible rhino-pharyngo-laryngoscope, is part of the primary clinical diagnosis of oropharyngeal and hypopharyngeal carcinoma. During panendoscopy under anaesthesia, the tracheo-bronchial system and the oesophagus can also be examined in order to rule out further secondary carcinomas and to determine the exact extent of the tumour by palpation and endoscopy while the patient is relaxed.

Suspect lesions distant from the primary tumour should be biopsied separately. In these situations, a mapping biopsy in inconspicuous areas of the mucosa may also be useful. The clinical examination also includes palpation of the neck to detect lymph node metastases, which is supplemented by ultrasound and CT/MRI if neoplasia is suspected. Newer endoscopic procedures such as coherence tomography, autofluorescence and electronic chromoendoscopy (narrow band imaging) can also be used.

In the context of the hitherto largely unstructured recording of the individual surgical risk or the individual stress limit with regard to therapeutic procedures to be performed, the systematic recording of comorbidities and so-called “frailty” (a state of reduced physical resistance after stress) has recently come to the fore. This is also related to the diversification of treatment options for head and neck cancer, which should of course be adapted to the individual conditions of a patient as part of a geriatric assessment with regard to undesirable treatment-related complications (adverse events). Patients older than 65 years make up the majority of cancer patients, which is why the group of older patients is particularly relevant and important for treatment in all oncological disciplines [137]. Therefore, assessment of frailty in elderly patients is recommended prior to surgical [138] or oncologic [139] therapy. On the other hand, increased symptom burden is an important risk factor for poor clinical outcomes [140] and the presence of mental health problems [141] in cancer patients as well. Established instruments such as the G8 questionnaire [142] or the ^MIDOS2^ [143] are currently available. Initial validations of these instruments in collectives of patients with head and neck tumours show a significant correlation between frailty and increased symptom burden and thus indicate coherence in HNSCC patients, as these two risk factors can predict the presence of the other [144], [145], [146]. A deeper understanding of the coherence between these two risk factors could potentially facilitate the achievement of a better quality of life by reducing treatment-related complication rates.

### 7.2 cT classification

There are no significant additions for oropharyngeal and hypopharyngeal carcinoma compared to the explanations for laryngeal carcinoma (S3 Guideline on Laryngeal Carcinoma 017-076OL, Section 6.2). More detailed descriptions of the cT classification can also be found in Section 6.4.1.

### 7.3 Imaging

**7.2 Consensus-based recommendation 2024**

*CT (contrast-enhanced) or MRI should be performed to determine the local extent of an oropharyngeal or hypopharyngeal carcinoma.*

- EC
- Strong consensus

**7.3 Consensus-based recommendation 2024**

*In order to avoid distortions of the contrast agent behaviour on the primary tumour, the tumour biopsy should only be performed after the slice imaging has been performed.*

- EC
- Strong consensus

**7.4 Consensus-based recommendation 2024**

*If artifacts are expected in the oropharynx due to metal in the oral cavity, MRI should be preferred to CT for the assessment of the primary tumour.*

- EC
- Strong consensus

**7.5 Consensus-based recommendation 2024**

*In locoregionally advanced tumours, an FDG-PET/CT should be performed to exclude distant metastases before function-restricting therapeutic measures.*

- EC
- Strong consensus

**7.6 Evidence-based statement 2024**

*PET-CT has no value in the primary diagnosis of the local extension of a known oropharyngeal or hypopharyngeal carcinoma.*

- LoE: 2+
- [1], [147], [148], [149], [150], [151], [152], [153]
- 2+: S3 guideline adaptation – Oral Cavity Carcinoma, Version 3.0 2021 (6.8)
- Strong consensus

**7.7 Evidence-based recommendation 2024**

*To determine the N category, the entire region from the base of the skull to the upper thoracic aperture should be examined with CT or MRI.*

- GoR: A
- LoE: 2+
- [1], [154], [155], [156], [157], [158], [159], [160], [161], [162]
- 2+: S3 guideline adaptation – Oral Cavity Carcinoma, Version 3.0 2021 (6.10)
- Strong consensus

**7.8 Evidence-based statement 2024**

*The diagnostic specificity of lymph node staging in the neck can be improved by ultrasound-guided fine-needle biopsy.*

- LoE: 2++
- [1], [163], [164], [165]
- 2++: S3 guideline adaptation – Oral Cavity Carcinoma, Version 3.0 2021 (6.11)
- Strong consensus

**7.9 Consensus-based statement 2024**

*The diagnostic specificity and sensitivity of lymph node staging in the neck is improved by FDG-PET-CT/MRI.*

- EC
- Strong consensus

**7.10 Evidence-based recommendation 2024**

*In patients with confirmed oropharyngeal and hypopharyngeal carcinoma, a chest CT should be performed to rule out pulmonary tumour involvement (filia, second carcinoma).*

- GoR: A
- LoE: 3
- [1], [166], [167], [168], [169]
- 3: S3 guideline adaptation – Oral Cavity Carcinoma, Version 3.0 2021 (6.13)
- Strong consensus

**7.11 Consensus-based recommendation 2024**

*As part of the primary diagnosis, imaging should be performed to rule out liver metastases.*

- EC
- Strong consensus

**7.12 Evidence-based recommendation 2024**

*In patients with suspected recurrence in the head and neck region, PET-CT can be performed if this cannot be confirmed or ruled out with CT and/or MRI.*

- GoR: 0
- LoE: 3
- [1], [170], [171], [172]
- 3: S3 guideline adaptation – Oral Cavity Carcinoma, Version 3.0 2021 (6.15)
- Strong consensus

**7.13 Consensus-based recommendation 2024**

*In patients with suspected recurrence in the head and neck region, a sonography of the head and neck region may be indicated to justify the indication for further measures.*

- EC
- Strong consensus

In addition to the clinical examination, the diagnosis of oropharyngeal and hypopharyngeal carcinoma also includes imaging measures such as ultrasound diagnostics, CT or MRI, chest X-ray or chest CT; PET-CT can also be used for special indications. As basic dental diagnostics, a panoramic tomography should be available to assess the dental status, also with regard to possible radiotherapy.

The information in the literature regarding the superiority of CT or MRI for diagnosing the primary tumour in the oropharynx or hypopharynx is inconsistent. A number of authors consider MRI to be the method of choice due to its higher sensitivity; in other publications, CT is classified as better or at least equivalent [173], [174]. CT examinations are generally better tolerated by patients than MRI examinations due to the short examination time [162]. The better soft tissue contrast with higher detail recognition of soft tissues and superficial structures and, above all, the lower artifacts caused by metallic fillings or implants speak in favour of MRI [175]. While CT is occasionally considered advantageous for the assessment of cortical erosion, MRI provides a better depiction of perineural, intramuscular [162], or perivascular tumour extension as well as a more precise diagnosis of any involvement of the skull base, orbit or cervical spine [175]. It has been shown that CT is perceived as more comfortable than MRI due to the faster examination technique [162].

In a meta-analysis that included 2 studies focusing on T-stage, 1 study focusing on T- and N-stage and 3 studies focusing on N-stage, the pooled sensitivity/specificity was 89.3%/89.5% (PET/CT) and 71.6%/8.0% for conventional cross-sectional imaging (CT and MRI) [176].

CT and MRI are of similar accuracy for the diagnosis of cervical lymph node metastases; they are clearly superior to clinical examination [177]. CT appears to be slightly more reliable than MRI for the visualization of infrahyoid lymph node metastases, while the latter appears to visualize the nodes along the vascular nerve sheath better. MRI is therefore recommended for routine diagnostics to determine soft tissue infiltration and lymph node status [155]. In a direct comparative study, MRI performed better than CT for the determination of cervical lymph node metastases in terms of sensitivity, specificity and accuracy [160]. In combination with FDG-PET, the diagnostic accuracy of MRI can be increased [159], without, however, allowing a reliable statement to be made about the dignity of the detected lymph nodes [172], [178]. In principle, however, PET is much less informative as a stand-alone method than in combination with CT or MRI [159], [179], [180], [181] and should therefore always be performed as a hybrid procedure (PET-CT or PET-MRI).

The accuracy of CT, MRI and ultrasound in the assessment of lymph node metastases is comparable, although the data on this is sparse. In the case of borderline large lymph nodes (short diameter >5 mm) on CT or MRI without signs of central necrosis, a targeted ultrasound-guided fine needle biopsy or FDG-PET can increase diagnostic accuracy [164], [165]. However, the value of PET-CT for the diagnosis of cervical lymph nodes is controversial due to the high number of false positive findings [153], [157], [182], [183]. This method is considered particularly unsuitable for lymph nodes less than 10 mm in size [182], [183].

In the 8^th^ edition of the TNM classification, the determination of extranodal tumour growth (ENE, extranodal extension) was excluded in pretherapeutic staging for HPV16/p16-positive oropharyngeal carcinomas, but it remains highly valid for postoperative adjuvant therapy decisions. In a meta-analysis that included 2,478 patients from 22 studies, the pooled sensitivity/specificity was 0.73/0.83 (CT) and 0.60/0.96 (MRI), with a lower pooled specificity for HPV-positive than for HPV-negative oropharyngeal cancers (0.74 vs. 0.87) [183].

A standard method for assessing the cervical lymph nodes is ultrasonography, for which a higher sensitivity and specificity have been reported in individual studies than for CT [184] or MRI [185]. It is an inexpensive method that can often be repeated during follow-up, but its accuracy and informative value depend heavily on the experience of the examiner. However, other studies indicate that the reliability of ultrasound staging of the neck is limited due to its low specificity [186].

Few studies have looked at the sensitivity of ultrasound-guided fine-needle biopsy (FNB) to determine pathologic lyphatic node dignity. While the sensitivity of this method is low for small tumours with a clinical N0 neck [187], [188], it can be helpful for preoperative confirmation of dignity in palpable LKs. Ultrasound-guided fine-needle aspiration has a higher specificity than CT for palpable lumps [177], but no higher overall diagnostic reliability [163].

A combination of the examination modalities CT, MRI and PET-CT does not lead to any significant improvement [156]. Despite its high sensitivity, PET-CT has no improved diagnostic value for primary tumours and therefore cannot replace the established CT or MRI procedures [150], [151], [189]. The meta-analysis published in 2008 by Kyzas et al. [157] included 32 studies on the diagnostic value of FDG-PET/CT in patients with a head and neck tumour. For cN0 patients, the sensitivity of FDG-PET alone was 50% (95% CI=37–63%) and the specificity was 87% (95% CI=76–93%). In studies with FDG-PET and anatomical imaging, the respective sensitivities and specificities were 80%/86% and 75%/79%, but not specified for cN0 patients.

In a further meta-analysis on the detection of distant metastases in advanced pharyngeal carcinoma, which summarized 1,166 patients from 9 studies, the following pooled sensitivity/specificity was found: 0.92/0.93 (PET/CT+MRI), 0.80/0.91 (PET/MRI), 0.79/0.88 (PET/CT). Metastases were found in 9.2% and synchronous tumours in 11.8% of the cohort [190].

The higher the T category, the higher the probability of the presence of a secondary tumour or metastases in the lung, although secondary tumours or distant metastases may also be present in smaller tumours (especially in the hypopharynx) [191]. For this reason, a chest CT is generally recommended as part of the diagnosis of the primary tumour (chest X-ray overview is obsolete for staging) [166]. Both LK metastases and a second pulmonary carcinoma can be detected by CT with high sensitivity and specificity [167]. In patients with suspected recurrence in the head and neck region, sonography of the neck regions may also be indicated to justify the indication for further measures [192]. In comparison with bone scintigraphy and abdominal ultrasound, CT proved to be the safest screening method for detecting distant metastases [166]. This also applies with high significance to the comparison of thoracic CT with conventional X-ray of the lungs [167], whereby CT detected either metastasis or a synchronous second carcinoma in approximately 11% of cases and was recommended as a screening method for patients with advanced primary tumours [168]. Due to its high sensitivity and the preferred location of secondary tumours/distant metastases in the lung, chest CT is recommended as an important imaging modality for primary staging in all patients with head and neck tumours [169]. Despite the very low overall incidence of liver metastases, this guideline nevertheless recommends liver imaging as an obligatory part of pre-therapeutic staging (recommendation 7.11: 100% consensus of mandate holders). At this point, it should not go unmentioned that some authors would only recommend abdominal imaging to exclude liver metastases to a limited extent, for example only in N2/3 situations [193], [194].

If a metastasis is detected in a single lymph node of category N2b or N2c, or if an involved lymph node measures more than 3 cm in maximum diameter (N2a or N3), there is a higher risk of distant metastases, a whole-body FDG-PET-CT is recommended as an alternative to CT thorax/abdomen to rule out or objectify distant metastases. for the objectification of distant metastases (sensitivity for the detection of distant metastases 96.8%, specificity 95.4%, positive predictive value 69.8%, and negative predictive value 99.6%) [195]. In a study by the Institute for Quality and Efficiency in Health Care, a benefit assessment of positron emission tomography (PET and PET/CT) for head and neck tumours was carried out. The primary objective was to examine the benefit of the method in metastatic cervical lymphadenopathy for the detection of the unknown primary tumour (cancer of unknown primary tumour, CUP syndrome). In addition, the extent to which PET or PET/CT is superior to standard diagnostic procedures without PET was investigated. A systematic database analysis was carried out for this purpose, whereby only one single usable comparative study could be identified with regard to recurrence-free 2-year survival, which neither proved nor disproved a patient-relevant benefit of PET [189]. For the question of staging of the primary tumour, CT and SPECT showed a higher specificity compared to PET, particularly for the detection of bone invasion [147]. PET also performed no better than CT or MRI for the diagnosis of cervical lymph node metastases, while PET tended to have a higher sensitivity than CT for the detection of distant metastases [157].

For recurrence detection, the few usable studies identified by IQWiG comparing PET vs. a combination of CT and/or MRI showed that PET had a significantly higher pooled sensitivity than the combination of CT and/or MRI. Here, the specificity is reduced by false positive findings due to accumulation in inflammatory lesions. However, FDG-PET showed a higher reliability with a sensitivity of 100% and a specificity of 61–71% than CT and/or MRI [172]. A good pooled sensitivity of 84% for PET/CT was also shown for the detection of an unknown primary tumour, which is why the assumption was made that both the combination with CT and PET alone is able to diagnose additional primary tumours after the primary diagnosis with CT and/or MRI has been completed. This is also confirmed by other studies, according to which FDG-PET not only diagnoses distant metastases more reliably, but also detects 24–26% more primary tumours than CT or MRI alone [172], [196], [197].

**7.14 Evidence-based recommendation 2024**

*After combined radio-chemotherapy, surveillance using FDG-PET-CT should precede a planned salvage neck dissection in node-positive patients with oropharyngeal or hypopharyngeal carcinoma.*

- GoR: B
- LoE: 1b
- [2], [195], [198], [199], [200], [201]
- 1b: S3 guideline adaptation – Laryngeal Carcinoma, Version 1.1 2019 (6.6)
- Strong consensus

**7.15 Evidence-based recommendation 2024**

*If the FDG-PET-CT is negative, the salvage neck dissection can be omitted.*

- GoR: B
- LoE: 1b
- [201], [202]
- Strong consensus

**7.16 Consensus-based recommendation 2024**

*Surveillance using FDG-PET-CT should take place 1* *2 we* *eks after the end of treatment.*

- EC
- Strong consensus

FDG-PET-CT plays a role in the follow-up of node-positive patients who have undergone primary combined radio-chemotherapy.

In an important, worldwide “practice changing” prospective randomized study, Mehanna et al. [201] found that survival rates were comparable between patients who underwent PET-CT surveillance and those who underwent elective neck dissection. After a median follow-up of 36 months, the 2-year overall survival of the 564 patients included was 84.9% in the image-guided follow-up group (N=54 lymph node dissections) and 81.5% in the lymph node dissection group (N=221). The hazard ratio (for death) clearly supported PET-CT-guided surveillance and indicated non-inferiority to the elective neck dissection group (upper limit of 95% CI for hazard ratio <1.50; p=0.004). Since the study was grouped according to the 7^th^ edition of the TNM classification, the recommendation of PET-CT surveillance after primary radiochemotherapy is currently given for all initially node-positive (N+) tumours in view of the possible underestimation in N1-categorized p16-positive oropharyngeal carcinomas in the 8^th^ edition.

Breik et al. [203] favours PET/CT for the follow-up of patients with oral cavity, oropharyngeal and hypopharyngeal carcinomas in the 3–6 month period after the end of therapy. In 140 patients, recurrences or metastases were found in 25%, 60% of them within the first 6 months. Sensitivity/specificity within 3–6 months after the end of therapy was 0.95/0.83 (PET/CT) and 0.60/0.86 (MRI), after 3 months 1.0/0.7 (PET/CT) and 0.50/0.86 (MRI), and after 6 months 0.93/0.87 (PET/CT) and 1.0/0.83 (MRI).

### 7.4 Panendoscopy

**7.17 Consensus-based recommendation 2024**

*Panendoscopy should be performed as part of the primary diagnosis of oropharyngeal and hypopharyngeal carcinomas.*

*It is a central component of primary diagnostics for more precise expansion of the primary tumour and for the detection of secondary carcinomas.*

- EC
- Strong consensus

Panendoscopy under anaesthesia includes oesophagoscopy, tracheobronchoscopy, pharyngoscopy (inspection of the nasopharynx, oropharynx and hypopharynx), microlaryngoscopy and inspection of the oral cavity. In analogy to the S3 Guideline on Laryngeal Carcinoma 017-076OL, Chapter 6.4, panendoscopy should be performed as a mandatory part of the primary diagnosis of oropharyngeal and hypopharyngeal carcinomas. It is a central part of primary diagnostics for a more precise diagnosis of the extent of the primary tumour and for the detection of secondary carcinomas.

#### Background

The frequency of clinically occult secondary carcinomas varies considerably in the literature from 1% to over 10%. There are indications that the frequency of secondary carcinomas depends on the stage of the primary tumour. In patients with oropharyngeal and hypopharyngeal carcinoma who smoke and regularly drink large amounts of alcohol, there is a probability that these noxious substances have also caused carcinomas or precursor lesions in other regions of the upper airways and oropharynx. The extent of this probability is stated very differently in the literature [204], [205], [206], [207], [208], [209], [210].

The literature predominantly describes a connection between primary tumours in the head and neck region, particularly the oropharynx and hypopharynx, and the occurrence of syn-/metachronous secondary carcinomas. Stöckli et al. [210] dealt intensively with this topic and described 16.2% secondary carcinomas in 358 patients examined. Here, 40% of synchronous secondary carcinomas were found in the primary tumour of the oral cavity and pharynx, of which 20% were detected exclusively by panendoscopy (i.e. remained undetected in imaging). Haughey et al. [206] found 14% of secondary carcinomas with the highest prevalence in oral cavity, oropharyngeal and hypopharyngeal carcinomas in the largest systematically evaluated series to date, including 40,287 patients. Di Martino et al. [205] evaluated the tumour registry of the ENT clinic at RWTH Aachen and found 7.7% metachronous carcinomas in 843 patients with head and neck carcinomas, 15.3% of which were in the lungs. With regard to the explicit occurrence of second tumours in the oesophagus in oral cavity carcinoma, figures of 0.8% are given, with significantly higher rates in pharyngeal carcinoma [211]. Koo et al. [212] observed second carcinomas for small oral cavity and pharyngeal carcinoma (tongue T1-2) patients with existing tobacco and alcohol consumption (not quantified) in 14.5–16.3% of patients. In the few never smokers/drinkers, the second carcinoma rate was 0%. Similar observations were published by Rodriguez-Bruno et al. [208]. Hung et al. [213] in a large cohort of 2,965 patients from the “Taiwanese Longitudinal Health Insurance Database 2000” (high-ranking publication in PloS One) found an odds ratio of 55.3 for synchronous oesophageal cancer in patients with oral cavity/pharyngeal cancer compared to the normal population group (prevalence 2.19% versus 0.04%) and strongly recommended performing panendoscopy in routine primary diagnostics. Sharma et al. [214] saw 5.56% second cancers (predominantly in the hypopharynx) in 234 patients in Germany, with these most commonly observed in primary oral cavity and oropharyngeal cancers. Less convincingly, Davidson et al. [204] concluded on the basis of a 154-patient series that routine panendoscopy was unnecessary for 2.6% of secondary cancers.

In England, the “Tumour Assessment and Staging: UK National Multidisciplinary Guidelines” [202] recommends panendoscopy under general anaesthesia as part of the primary diagnosis. It is important that the endoscopy is performed by a “senior surgeon”. The authors critically concede that although there is a documented proportion of secondary cancers for panendoscopy in the literature, according to a review by McGarey et al. [215] the proportion of secondary cancers has decreased in the last three decades and therefore rigid oesophagoscopy in particular should only be performed in patients with primary tumours in whom an increased incidence of secondary cancers has been described (oropharynx and hypopharynx are addressed). In the guidelines and the larger studies mentioned here, the anaesthesia- and surgery-associated risk of panendoscopy is considered to be subordinate or negligible in relation to the more precise initial diagnosis and thus undisputed added value for the patient.

The NCCN Guidelines, Version 2.2022, Cancer of the Oro- and Hypopharynx, explicitly recommend performing panendoscopy under general anaesthesia (examination under anaesthesia (EUA) with endoscopy) as part of the pre-therapeutic staging examination

Clinical experience has shown that the imaging of small and early, superficially located squamous cell carcinomas of the mucous membranes of the upper aerodigestive tract is often inadequate, as even the new high-resolution cross-sectional imaging and PET hybrid procedures cannot detect these lesions. For this reason, panendoscopy is considered more important than imaging when it comes to determining the exact extent of the tumour and visualizing metachronous secondary carcinomas (especially in the oesophagus). A distinction must be made between this and the targeted biopsy endoscopy performed for histological verification in the case of apparent abnormalities in the mirror examination or imaging. The latter does not replace the panendoscopy required in the context of staging to rule out secondary carcinomas and visualize the extent of the primary tumour.

The proposal under discussion that panendoscopy should only be performed in cases where there is a history of increased risk [208] is not expedient in the light of the literature. It is known from numerous epidemiological studies that many head and neck tumour patients dissimulate in their medical history as part of the primary diagnosis and to a large extent provide unreliable information on tobacco and alcohol consumption. Even with the use of very extensive epidemiological data collection instruments, it was not possible to overcome the existing inherent retrospective imprecision. In the case of the association with HPV16, this trigger only applies to the oropharynx. Nevertheless, panendocopy should remain an obligatory diagnostic component even for p16-positive oropharyngeal carcinomas, as combined risk factor constellations are predominant, particularly in Germany. Second cancers can occur synchronously or metachronously. Synchronous secondary carcinomas may already be clinically apparent at the time of the manifestation of oropharyngeal and hypopharyngeal carcinoma during the ear, nose and throat examination or one may become aware of them due to the specific symptoms. Panendoscopy is suitable for the exclusion or detection of clinically occult secondary carcinomas and their precursors and cannot be replaced by imaging. According to publications and the prevailing expert opinion, it is therefore one of the mandatory diagnostic measures to be performed prior to a therapeutic decision on histologically proven oropharyngeal or hypopharyngeal carcinoma.

#### Performance of panendoscopy

Panendoscopy is usually performed together with endoscopy of the expected tumour region, which also includes a tissue sample (biopsy). Palpation of the oral cavity, in particular the tongue, the base of the tongue and the accessible pharyngeal region under anaesthesia during the initial phase of maximum muscle relaxation is considered an important step during panendoscopy. This gives the surgeon a good three-dimensional impression of the extent of the tumour. Panendoscopy addresses the entire upper airway and alimentary canal, i.e. the entire oral cavity, the entire naso-, oro- and hypopharynx and the larynx are inspected. In addition, an endoscopy of the tracheobronchial system and the oesophagus is performed (secondary carcinomas are often found in the distal oesophagus). The oral cavity and oropharynx can be inspected directly without an endoscope, although the mucosa, base of the tongue, hypopharynx and larynx should be assessed with an endoscope. Laryngoscopes, such as the “Kleinsasser tube”, are usually used for this purpose. Endoscopy of the tracheobronchial system can be performed using rigid endoscopes with different angles. Oesophagoscopy can also be performed with a rigid, preferably pneumatic oesophagoscope. Due to the possible complications, such as perforation of the oesophageal wall, particularly at the transition from the piriform sinus into the oesophagus, but also distally, some authors recommend performing oesophagoscopy with a flexible endoscope. The disadvantage of a flexible endoscope, particularly in the hypopharynx, is that this region cannot be stretched. Rigid endoscopy is therefore advantageous for inspecting the postcricoid area. Tracheobronchoscopy can also be performed optically with flexibility.

In the presence of hypopharyngeal carcinoma with laryngeal involvement, microlaryngoscopy performed as part of panendoscopy is of particular importance in order to define the extent of the primary tumour. In particular, superficial mucosal changes that cannot be detected by imaging can be easily recognized. The piriform sinus is often compressed in sectional imaging, so that the exact extent of the tumour can often not be precisely determined if there is surrounding oedema. The endoscopic “stretching” of this region is of great additional diagnostic benefit. In addition, microlaryngoscopy performed prior to treatment can determine whether the entire larynx can be adjusted endoscopically in order to possibly perform a transoral laser surgical resection during definitive treatment. In these situations, a mapping biopsy may also be useful.

The findings concerning the primary tumour extension obtained during panendoscopy including palpation are entered or drawn into a prefabricated pictogram. It is advisable to check the resectability and enter this on the document (midline crossing of the base of the tongue, etc.). In the event of resectability, the exact surgical procedure should be determined (transoral adjustability, necessity of reconstruction, type of reconstruction, etc.).

### 7.5 Biopsy

**7.18 Consensus-based recommendation 2024**

*In the case of biopsies, the sample should be taken from the edge of the tumour and, if possible, centrally from the base of the tumour.*

- EC
- Strong consensus

The removal of a tissue sample and its subsequent histopathological examination to detect/exclude of a malignant tumour or a precursor lesion is an essential prerequisite for the initiation of tumour-specific treatment (S1-BDP). In analogy to the S3 Guideline on Laryngeal Carcinoma 017-076OL, Chapter 6.5, a similar procedure should be followed for oropharyngeal and hypopharyngeal carcinoma, although excisional biopsies are much less common here. The biopsy is usually performed as part of panendoscopy. It is generally recommended that imaging relevant to further treatment is carried out before a tissue sample is taken, as the biopsy can lead to tissue reactions that make assessment during imaging more difficult or falsify it.

In the case of macroscopically clearly identifiable advanced tumours, the tissue sample should preferably be taken from the peripheral area of the tumour, i.e. the progression zone, and under no circumstances from the necrotic centre only. However, it is also desirable to collect tumour bases from non-necrotic areas where feasible. The usual form of biopsy for macroscopically clear, advanced tumours is an incisional biopsy with microshears or sharp forceps (Blakesley). An excisional biopsy should only be performed for circumscribed lesions without deep infiltration. This procedure is particularly suitable for older patients in order to avoid a second procedure under anaesthesia in the case of in sano resection (R0). Areas of mucosa that are suspicious for the presence of a precursor lesion should also be biopsied (see above). A so-called brush biopsy is not recommended. Photo documentation of the tumour during panendoscopy under anaesthesia and prior to sampling is recommended and is of great benefit in the subsequent treatment decision. If the histology findings are unexpectedly negative, the biopsy should be repeated at least once. The histomorphological parameters to be indicated are described above; the description of the R status is only useful for the excisional biopsy.

#### 7.5.1 Detection of HPV16, p16

P16 immunohistology can be used in many scenarios as a surrogate marker of HPV infection in squamous cell carcinoma of the head and neck [216] (see detailed explanations in Chapters 4.3.3 and 6.2). It has been suggested that a case should be considered positive if more than 70% of the tumour cells express p16 at least moderately in nuclear and cytoplasmic levels, although slightly different cut-offs have also been proposed [93], [94]. Different p16 antibodies show slightly different positivity rates, but result in comparable expression patterns overall. The inter-observer variability in the evaluation appears to be rather low [94].

HPV detection using molecular methods (extract-based using PCR/array or in situ using hybridization to detect HPV DNA/RNA) can be used confirmatively in cases with unclear p16 staining or other limitations in the predictivity (see also Section 4.3.3) of a p16 immunohistology for an HPV association. The variety of molecular methods available is large, and no single one can be recommended here as being particularly sensitive. The method used should be checked regularly in inter-laboratory tests, it seems desirable to include positive controls.

#### 7.5.2 Detection of PD-L1

PD-L1 positivity (programmed cell death ligand 1) plays a role in the stratification of squamous cell carcinomas of the head and neck region for treatment with immune checkpoint inhibitors. Determination as part of the primary diagnosis is useful, but according to the current approval situation, it can only be recommended as mandatory in the diagnosis of recurrence or distant metastasis. As distant metastases can often not be confirmed with biopsy, the PD-L1 status obtained during primary diagnosis is a valuable marker for the initiation of first or second-line therapy.

Of four established different PD-L1 scoring algorithms, two are currently relevant for approval for squamous cell carcinomas of the head and neck region: the CPS (combined positive score) and the TPS (tumour positive score).

CPS describes the proportion of PD-L1-positive tumour and immune cells (lymphocytes and macrophages) in relation to all tumour cells, multiplied by 100. The value is given as a pure number, i.e. not as a percentage.

PD-L1-positive cells

(tumour cells, lymphocytes, macrophages)

CPS = ____________________________________ x 100

Total number of vital tumour cells

TPS is the proportion of tumour cells that are PD-L1-positive. The value is given in %.

PD-L1-positive tumour cells

TPS (%) = __________________________ x 100

Total number of vital tumour cells

These variables cannot be converted into each other mathematically and should therefore both be requested diagnostically when asking about the PD-L1 status.

The IC score (immune cell score: IC indicates the percentage of the tumour area that is occupied by PD-L1-positive tumour-infiltrating immune cells, such as lymphocytes, macrophages, granulocytes, dendritic cells) and the TAP (tumour area positive) score can also be considered, albeit they are currently less relevant, as the study situation regarding immune checkpoint blockade is changing rapidly and future approvals are difficult to predict. The evaluation according to IC or TAP score currently plays no role in treatment decisions for oropharyngeal and hypopharyngeal carcinoma in the first or second line.

The interchangeability of different assay systems for PD-L1 measurement has long been discussed, but ultimately appears to be broadly possible in different entities. The evaluator variability is also acceptable according to current studies [217]. Participation in quality assurance programs seems advisable in this respect.

### 7.6 Sentinel lymph nodes

**7.19 Consensus-based recommendation 2024**

*No recommendation can be made for SLN biopsy as a method of avoiding elective neck lymph node evacuation in oropharyngeal and hypopharyngeal carcinoma.*

- EC
- Strong consensus

In analogy to the S3 Guideline on Laryngeal Carcinoma 017-076OL, Chapter 6.6 [2], no recommendation can be made for the suitability of SLN biopsy as a method for avoiding elective neck lymph node removal in oropharyngeal and hypopharyngeal carcinoma. Sentinel lymph nodes are those lymph nodes that are closest to the tumour in the lymph drainage area of a malignant tumour. If tumour cells have already been carried into these lymph nodes with the lymph flow and have led to a metastasis, further metastases are likely to be found in downstream lymph nodes. If, on the other hand, no metastases are found in the removed sentinel lymph node, there is a low probability that further lymph node metastases are present. In this respect, this procedure is of particular importance in cN0 neck, as it is hoped that it will eliminate the need for an elective neck dissection. The sentinel lymph node technique is a clinically established technique for breast carcinoma, prostate carcinoma and melanoma.

In the head and neck area, the oral cavity is considered to be an important primary tumour site that can be considered for meaningful SNB. An initial consensus guideline is available [218], which describes the high potential of SNB but does not yet make any recommendations for routine clinical practice. The S3 Guideline on Oral Cavity Carcinoma 007-100OL, Version 3.4, Chapter 8.3, also does not recommend the routine performance of SNB: there is no robust evidence from clinically controlled studies for the suitability of SLN biopsy as a method to avoid elective neck lymph node evacuation. In the most comprehensive and most recent meta-analysis by Liu et al. [219], which is also assessed as having only a low risk of bias, 66 studies with 3,566 tongue cancer patients (i.e. also some with oropharyngeal carcinoma) are analysed and the sensitivity of SLN biopsy with regard to the detection of lymph node metastasis is stated as 0.87 [0.85–0.89] (with inclusion of all studies including the validation studies with immediately subsequent neck dissection). Considering only the studies in which patients with negative SLN were followed up and the false negatives were detected over the course, the sensitivity is 0.85 [0.82–0.88]. In the results to date, however, SLN biopsy is equivalent in terms of survival after conventional neck dissections for small squamous cell carcinomas (T1/T2) [220], [221]. In terms of postoperative quality of life and functionality, there are advantages for SLN biopsy [222], as well as in terms of lower complication rates [221]. According to the S3 Guideline on Oral Cavity Carcinoma, SLN biopsy can be offered for early, transorally resectable oral cavity carcinomas that do not require a transcervical approach in the same procedure. If the sentinel lymph node is positive and detection is uncertain, a complete neck dissection should be performed.

In more recent studies that primarily focus on the oropharynx and hypopharynx, a meta-analysis by van den Bosch et al. [223] stands out. In a pooled analysis of 19 studies and 377 patients, the sensitivity and negative predictive value were estimated at 0.93 (95% CI: 0.86–0.96) and 0.97 (95% CI: 0.94–0.98). According to the authors, the accuracy of the SNB justifies its place in the diagnosis of patients with oropharyngeal and hypopharyngeal carcinomas. However, randomized studies for further verification are lacking. Further meta-analyses underline the high sensitivity and specificity in oropharyngeal and hypopharyngeal carcinoma [224], but this has not yet led to further recommendations in guidelines. This is probably also due to the fact that many authors do not consider elective selective neck dissection with very low morbidity to be a disadvantage. Werner et al. [225] were also able to show that SNB is safest when several detected lymph nodes (up to three) are removed, so that the assumed advantage over selective neck dissection has diminished.

### 7.7 Patient information/education

**7.20 Consensus-based statement 2024**

*Information about their illness and the resulting therapeutic options, including the alternatives, is a basic prerequisite for patients to make an informed decision about treatment.*

- EC
- Strong consensus

**7.21 Consensus-based recommendation 2024**

*The patient should be informed in detail and repeatedly about their illness, treatment options and subsequent disorders in accordance with their individual needs.*

*In addition to being informed about therapeutic mea* *s* *u* *res, the patient should also be informed about the necessary rehabilitation, including social and professional integration.*

- EC
- Strong consensus

This chapter will be implemented with minor specifications and updates in analogy to the S3 Guideline on Laryngeal Carcinoma 017-076OL, Chapter 6.7.

In view of a person’s right to self-determination, it is solely up to the of-age, consenting patient to decide whether or not a particular medical treatment should be carried out by a doctor. Treatment by the doctor is therefore only justified and thus exempt from punishment if the patient has previously consented to it. Informing the patient about what is to be done to them, by what means and with what risks and consequences is a prerequisite for the patient’s informed consent to the treatment [226], [227]. It is a mandatory legal requirement (BGB, MBO, ...) and is the sole responsibility of the doctor. The information must be provided in good time so that the patient can make a well-considered decision about consent (Section 630e (2) no. 2 BGB). In Germany, the Act to Improve Patients’ Rights (Patients’ Rights Act; [228]) has been in force since 2013. In this law, the standards previously scattered in various legal texts were combined into one law. In particular, the treatment contract is regulated more clearly under the new paragraph § 630a BGB. It also stipulates that the patient must be comprehensively informed about all circumstances relevant to the treatment, such as diagnosis, prospects of success, risks and therapy as well as various treatment alternatives. The patient also has a duty to cooperate, particularly with regard to the medical history [227], [228]. The law also stipulates the obligation of the treating party to provide information about their own errors, as well as the errors of other treating parties, based on certain circumstances, e.g. a health risk that has arisen. Even if the reversal of the burden of proof to the detriment of the treating party still only comes into effect in connection with gross malpractice, the law regulates numerous simplifications of the burden of proof for the patient. For example, the treating party must prove that they did not make a mistake if, for example, there was a controllable risk, errors were made in the information provided or its documentation or the treating party simply lacked the suitability for the measure carried out, e.g. in the course of a novice operation [226].

A patient who is confronted with the diagnosis of oropharyngeal or hypopharyngeal carcinoma must come to terms with this situation. The informative discussion with the attending physician is of great importance for this. The patient needs sufficient time to come to grips with the diagnosis of “carcinoma”. For this reason, information is not usually provided in a single consultation, but as a process that accompanies the course of the disease. In addition to the patient, relatives also play a major role, who should of course only be fully informed about the disease with the patient’s consent, as they will support the patient in their home environment. Once the diagnosis has been communicated, the patient is often not fully receptive. Much of what is explained to them in the first phase is often not properly understood, especially the consequences of treatment, be it surgery, radiotherapy or chemotherapy. For this reason, it is also important to inform relatives about the consequences of treatment. Informing patients and relatives together makes communication within the partnership and family easier. This should be communicated to the patient.

As several functions such as voice production, swallowing and breathing can be impaired in the case of oropharyngeal and hypopharyngeal carcinoma, the patient must be given a detailed explanation of the possible disorders that may arise after a specific therapy. This initial informative discussion with the patient and their relatives is important so that they can decide on the right therapy for them. However, the patient will often follow the doctor’s recommendation, so it is all the more important to explain the consequences of the treatment. As most patients have no idea of the anatomy of the upper aerodigestive tract, the explanations must be clearly explained to the patient with the aid of diagrams. Many patients also need to be made aware of the seriousness of the disease; this applies in particular to smaller carcinomas, the treatment of which would only lead to minor functional impairment. In order to present the patient with a broad picture of the treatment options following the decision of the interdisciplinary tumour board, it is generally advisable to consult an ENT surgeon as well as a radiotherapist (and an internal oncologist if the treatment option is appropriate), who can accompany the consultation.

The patient must always be informed about the prospects of success. If information is provided on the probability of recovery, this should be given as absolute (and not relative) frequencies where possible [229], [230]. The probable risks and consequences of non-therapy should also be explained.

The presence of the patient is also possible (but not obligatory) at tumour board meetings, so that the advantages and disadvantages of various treatment options, including the prospects of success, can be discussed in the patient’s presence. A representative of the self-help groups should also be included in the patient’s explanation. It is very helpful if contact can be established with a patient who has undergone similar treatment. This gives the patient a clearer picture of the situation after the therapy and gives them a better idea of the functional impairments as well as the rehabilitative options.

If the patient has decided on a therapy or sensible alternative recommended by the interdisciplinary tumour board, this must of course be explained in detail. The information should be provided by the surgeon himself or by a doctor who has sufficient experience in the field of the proposed therapy. It is particularly important for the patient to be informed about the prognosis with regard to voice production, breathing and swallowing as well as visible – and therefore possibly stigmatizing – changes to the face and neck after the therapeutic procedure. It is also important to know whether a permanent tracheostomy or a temporary tracheostomy is being performed and whether a tracheostomy tube needs to be worn. In the case of operations in the oropharyngeal and hypopharyngeal region, aspiration is not an insignificant problem and must be explained to the patient, in addition to the possible impairment of swallowing function and voice production. Appropriately illustrated, standardized information sheets should be used to support the oral information provided to the patient, which is mandatory in all cases. Handwritten entries of important complications in the information sheets are essential for documenting the oral information provided to the patient. According to the Patients’ Rights Act, the patient must be given a copy of the information sheet (Act on the Improvement of Patients’ Rights). The contents of the information sheet depend on the type of treatment; the radio-oncologist is responsible for providing information about primary radiotherapy, while information on surgical treatment is provided by the ear, nose and throat specialist.

In the case of primary tumour treatment, an appropriate differentiation must be made, including both transoral laser surgery and transcervical surgery. The exact location of the tumour must also be taken into account. In addition to the treatment of the primary tumour, the patient must also be informed about the treatment of the lymphatic drainage channels. The necessity of an elective neck dissection as well as that of a therapeutic neck dissection must be fully explained to the patient. The patient must be informed that changes to the treatment recommendations are possible after the primary tumour has been treated, taking into account the histological findings available at that time. These must then be explained again. Furthermore, in the case of a partial resection of the larynx, the temporary tracheotomy that may be necessary in some cases should be discussed in detail with the patient. As adjuvant therapy in the form of radiochemotherapy is necessary in addition to surgical treatment in many cases of extensive tumours, especially if lymph node metastasis has been detected, the patient must also be informed about this. In particular, it must be explained to the patient why adjuvant therapy in the form of radiotherapy and chemotherapy is necessary despite the surgical extirpation of the tumour. Chemotherapy accompanying radiotherapy has become established in recent years, and patients must be informed in detail about the benefits of this additional treatment and the corresponding side effects.

The informing physician should also consider how the patient can mentally process the information provided. Irrational “subjective theories of illness” should be counteracted.

Despite truthful information, hope can be conveyed even if the prognosis is unfavourable, if necessary by providing information about palliative treatment options.

Staff with experience in psycho-oncology can be helpful for patients with psychological stress and/or their relatives.

In the case of clinical trials, the patient must be informed in detail in advance about the content of the trial and must of course be free to decide whether he or she is available for a corresponding trial. The patient must also be informed in detail if tissue asservation is planned as part of tumour biobanks. The attending physician must explain to the patient the advantages of such a biobank for future research projects and how it can enable later research. Since the majority of oropharyngeal and hypopharyngeal carcinomas are caused by noxious substances such as smoking and alcohol abuse, this connection must be clearly explained to the patient, particularly with regard to their behaviour after treatment. The patient should also be informed about the possibilities of functional rehabilitation, e.g. through speech therapy measures and any PEG placement that may be considered appropriate.

On discharge from hospital, the doctors providing further treatment should be informed of the reasons for the choice of therapy. This concerns not only information about the type and extent of the tumour treated, but also information about decision-making by the tumour board and the patient.

## 8 Treatment recommendations in the primary therapy of oropharyngeal and hypopharyngeal carcinoma, taking into account effectiveness, functionality and quality of life

The following chapter looks at the current evidence for the various approaches to primary treatment of oropharyngeal and hypopharyngeal cancer. The generation of the best possible current evidence on treatment recommendations was the focus of the de novo research work. In addition to overall survival, progression-free survival and treatment response, the focus was equally on functionality and quality of life. Chapter 8 is therefore largely based on the evidence tables compiled as part of the guideline work, which can be viewed in the appendix. In addition to the few randomized prospective studies, the numerous larger, non-randomized retrospective studies/case series and the registry studies were also included in the tables. It has been shown that prospective controlled studies with defined standard and test arms are difficult to implement, particularly for the primary surgical treatment of oropharyngeal and hypopharyngeal carcinoma (in contrast to definitive radiochemotherapy or first and second-line treatment in the recurrence or metastatic stage), due to the large heterogeneity in terms of stage, localization and surgical technique with large numbers of cases. Randomized studies on the primary surgical approach compared to definitive radio- or radiochemotherapy for oropharyngeal carcinoma have only been conducted for patients with localized disease in early stages (cT1-2 cN0-1) with very small numbers of cases [231], [232] and are therefore of limited significance.

In hypopharyngeal carcinoma, there is only one randomized study of medium size (n=194) from the 1990s, in which a laryngectomy followed by adjuvant radiotherapy was compared with induction chemotherapy followed by radiotherapy if there was a response to chemotherapy or a laryngectomy followed by adjuvant radiotherapy if there was no response to chemotherapy in locally advanced disease [233]. In this respect, registry studies with very large case numbers (e.g. Yoshida et al.: 4,473 patients from the National Cancer Data Base, NCDB [234]) and retrospective case series must be taken into account for this entity in particular.

**8.1 Consensus-based recommendation 2024**

*The treatment of oropharyngeal and hypopharyngeal carcinoma should be carried out on an interdisciplinary basis after coordination of each individual case within tumour boards involving the specialist disciplines of otorhinolaryngology, oral and maxillofacial surgery, radiation oncology, oncology, pathology and radiology.*

- EC
- Strong consensus

**8.2 Consensus-based recommendation 2024**

*The patient should be informed in detail and repeatedly about their illness, treatment options and subsequent disorders.*

- EC
- Strong consensus

**8.3 Consensus-based recommendation 2024**

Patients with oropharyngeal and hypopharyngeal carcinoma should be examined by an experienced dentist to determine their dental status before starting treatment.

- EC
- Strong consensus

**8.4 Consensus-based recommendation 2024**

*Therapy breaks beyond the planned weekend breaks should be avoided during radiotherapy.*

- EC
- Strong consensus

**8.5 Consensus-based recommendation 2024**

*If possible, unplanned breaks in therapy should be compensated for by twice daily irradiation on 1–2 days of the week with an unchanged single dose.*

*An interval of at least 6 hours should be maintained between 2 fractions.*

- EC
- Strong consensus

The treatment recommendations for oropharyngeal and hypopharyngeal carcinoma are compiled on the basis of the 8^th^ edition of the TNM classification (Section 6.4, list in Section 1.4.3, list in Section 1.5, Table 1 (Tab. 1), Table 2 (Tab. 2)). Due to the distinction between p16-positive and p16-negative in oropharyngeal carcinoma, different T and N categories are sometimes hidden behind the same stages, which are formulated in the respective headings.

The general treatment strategies for oropharyngeal carcinoma do not differ depending on the p16 status, despite the significantly better prognosis for HPV/p16-positive tumours. However, registry studies (e.g. [235]) show that in HPV/p16-positive oropharyngeal carcinomas, the results of radiochemotherapy are still positive for relatively high tumour burden up to cT3 tumours (stage I, II, not stage III) with bilateral lymph node involvement (N2) are equivalent to the primary surgical approach (± radiotherapy or radiochemotherapy) with regard to all important oncological and functional endpoints, which is only well documented for HPV/p16-negative tumours with a smaller tumour burden (cT1-2 cN0) (see evidence tables PICO-2). The differences in the TNM classification mean that both cT3cN2 (p16+) and cT2cN0 (p16–) correspond to stage II and the treatment recommendations do not differ between HPV/p16+ and HPV/p16–. The different classification of HPV/p16-positive tumours can therefore lead to confusion, which is illustrated once again in the following example: a 1.5 cm p16-positive tonsillar carcinoma with 3 ipsilateral 2–4 cm cervical lymph node metastases would be classified as cT1 cN1 cM0 and therefore stage I according to the 8^th^ edition of the TNM classification. In the case of p16 negativity, the identical tumour extension is classified as cT1 cN2b cM0, stage IVa. However, since p16 reduces the tumour stage from IVa to I, the maximum therapy would still be recommended in both cases according to current evidence, i.e. either primary surgical therapy with adjuvant radiochemotherapy with cisplatin or primary radiochemotherapy with cisplatin. Oropharyngeal carcinomas “down-staged” by p16 positivity therefore run the risk of being underestimated purely in terms of classification (for detailed explanations, see Chapter 6).

The recommendations for primary treatment of hypopharyngeal carcinoma are shown separately.

### 8.1 UICC stage I and II oropharyngeal carcinomas (p16-positive: T1-T3N2; p16-neg.: T1-T2N0)

P16 categorizes differently in the T and N distribution according to TNM stages. T3 tumours are also classified within stage II in the case of p16 positivity, whereas only T2 tumours are stage II in the case of p16 negativity. pN2 is still assigned to stage II in the case of p16 positivity for T2 tumours, otherwise already stage IV. pN0 is required for stage II in the case of p16 negativity. The ECS (+) is only included in the TNM consideration in the case of p16 negativity, but is of great importance for the treatment decision irrespective of p16.

**8.6 Evidence-based statement 2024**

*There is evidence that the results of primary surgical therapy (± adjuvant radio/radiochemotherapy) and primary radio/radiochemotherapy do not differ signifi* *c* *a* *ntly in terms of overall survival, relapse-free survival, locoregional relapse rate and distant metastasis-free survival in patients with HPV/p16-positive oropharyngeal carcinoma in stages I–II (UICC 8* *^th^* *edition).*

- LoE: 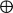

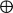

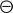

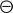
- [231], [232], [235], [236], [237], [238], [239]
- Strong consensus

**8.7 Evidence-based recommendation 2024**

*Patients with HPV/p16* ***positive*** *oropharyngeal carcinoma in stages I–II (UICC 8* *^th^* *edition) should receive either primary surgical therapy (± adjuvant radiotherapy or radiochemotherapy) or primary radiotherapy or radiochemotherapy.*

- GoR: B
- LoE: 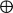

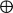

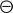

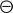
- [231], [232], [235], [236], [237], [238], [239]
- Strong consensus

**8.8 Evidence-based recommendation 2024**

*Patients with HPV/p16 negative oropharyngeal carcinoma in stages I, II (cT1N0, cT2N0; UICC 8* *^th^* *edition) should receive either primary surgical therapy (± adjuvant radio- or radiochemotherapy) or primary radio- or radiochemotherapy.*

- GoR: B
- LoE: 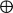

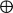

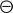

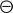
- [235]
- Strong consensus

#### 8.1.1 Surgical therapy

**8.9 Evidence-based recommendation 2024**

*In the case of a primary surgical approach, a transoral procedure for primary tumour resection (transoral laser microsurgery, TLM; transoral robotic surgery, TORS) should be preferred to a procedure with a transcervical approach for T1 and T2 tumours of the oropharynx, HPV/p16* ***positive and negative*** *.*

- GoR: B
- LoE: 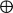

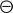

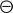

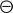
- [219], [240], [241], [242], [243], [244]
- Strong consensus

**8.10 Consensus-based recommendation 2024**

*If a transoral surgical technique (TLM, TORS) is chosen for a laterally located tumour with an increased risk of postoperative bleeding and is combined with neck dissection, consideration should be given to clipping/bandaging the arterial vessels supplying the tumour side transcervically in order to reduce the intraoperative and postoperative risk of bleeding.*

- EC
- Strong consensus

The indication for primary surgical treatment of oropharyngeal carcinoma is viewed heterogeneously worldwide. In German-speaking countries, there is a traditional preference for a primary surgical approach given reasonable R0 resectability (see also Section 6.6 Surgical safety margin). Internationally, primary surgical therapies are considered comparable to primary radiotherapy or radiochemotherapy in terms of effectiveness, functionality and late toxicity. Especially in very early stages (cT1N0, p16-independent), surgery with radiotherapy alone is on the same level of recommendation, although large prospective randomized studies on this issue are still pending.

Some authors prefer monomodal therapies (i.e. surgery or radiotherapy alone) to a multimodal approach for reasons of principle. This means that if adjuvant radiotherapy is considered necessary after surgery, radiotherapy alone should be preferred (1 vs. 2 modalities) in order to reduce the accumulation of the various modality-dependent toxicities/comorbidities. Similarly, it is argued that in the case of a necessary postoperative risk constellation with necessary adjuvant radiochemotherapy, it is not better to give preference to primary radiochemotherapy (2 vs. 3 modalities). Cheraghlou et al. [245] conducted a registry study with 4,443 patients with HPV-positive oropharyngeal carcinoma of all tumour stages based on the National Cancer Data Base, classified according to the current 8^th^ TNM edition (Evidence Table PICO-2, registry-based studies). In patients with stage I disease, treatment with definitive radiotherapy alone was associated with significantly reduced survival compared with radiochemotherapy (hazard ratio [HR], 1.798; P=0.029) or surgery with adjuvant radiotherapy (HR 2.563; P=0.002) or surgery with adjuvant radiochemotherapy (HR, 2.427; p=0.001). There was no significant difference after primary radiochemotherapy compared to surgery with adjuvant radiotherapy. In patients with stage II disease, significantly poorer survival was observed in patients treated with a single modality (either surgery [HR, 2.539; P=0.009] or radiotherapy [HR, 2.200; P=0.030]) compared to treatment with radiochemotherapy. Primary radiochemotherapy and surgery followed by adjuvant radiochemotherapy were equivalent. In patients with stage III disease, triple-modality therapy (surgery + adjuvant radiochemotherapy) was associated with significantly improved survival (HR 0.518; P=0.024) compared to treatment with definitive radiochemotherapy alone. Similar results in favour of the trimodal approach were also observed for advanced p16-negative oropharyngeal carcinoma (retrospective cohort with 131 patients; [246]). The data to date therefore show that the “mono-meets multimodal” approach is not easily transferable, at least for oropharyngeal carcinoma.

In general, a trend towards transoral resection (as opposed to classic open approaches) + neck dissection + adjuvant radio- (chemo-) therapy has been observed over the last 20 years. Transoral surgery (TOS) techniques, including transoral laser microsurgery (TLM) and transoral robotic surgery (TORS), have been promoted in retrospective comparisons with conventional surgery or primary radiochemotherapy as gentle, minimally invasive procedures with good late functional outcomes [247], [248], [249], [250], [251], [252], [253], [254].

In order to determine the current best evidence in the comparative consideration of transoral (TORS, TLM, TOS) versus open surgical procedures, PICO question 4 was asked (evidence table PICO-4: Primary transoral surgical treatment procedures vs. open classical surgery). Only retrospective, predominantly non-randomized comparative studies and systematic reviews/meta-analyses for different head and neck localizations (larynx, oro- and hypopharynx) of different tumour sizes (predominantly T1-2) with low or very low evidence were found. The endpoints were different (mortality [241], [243], [244], 2-year DFS [241], 3-year DFS [243], [255], recurrences [243], [244], [255], tracheostomy postoperatively [243], swallowing function 1 week and 1 year [244], intraoperative blood loss [241], [244], [256], complications [257], length of hospital stay [241], [243], PEG after 1 month and 1 year [256]). The meta-analyses indicate that TORS may have better disease-free survival (DFS) and a reduced risk of free flap reconstruction compared to open surgery. TORS was associated with fewer tumour-positive resection margins (R1), a lower number of recurrences, fewer intraoperative tracheostomies, a shorter hospital stay and a shorter duration of postoperative nasal tube feeding compared to open surgery [240], [258]. Compared to the more invasive techniques of conventional surgery, TORS appears to be less time-consuming and associated with less access morbidity [253], [259].

The only prospective randomized trials to date that have directly compared TOS (transoral techniques such as TORS, TLM, etc.) with radiochemotherapy are the ORATOR 1 (phase II; T1-2 N0-2 tumours; 34 patients per arm, 88% p16-positive) and ORATOR 2 trials (ORATOR 1; Nicholas et al. [231], [236]; ORATOR 2, Palma et al. [232]) (ORATOR: Oropharyngeal Radiotherapy versus Transoral Robotic Surgery, Evidence Table PICO-3). The primary endpoint of the ORATOR 1 study addressed quality of life related to swallowing function (MD Anderson Dysphagia Inventory; MDADI score) after one year, with a clinically meaningful difference defined as a difference of more than 10 points. In the ORATOR 2 phase 2 trial, TOS + neck dissection + reduced adjuvant radiation dose (de-escalation arm) was compared with definitive radiochemotherapy for T1-2 N0-2 oropharyngeal carcinoma (100% p16 positive) with the endpoint “overall survival”. A 1:1 randomization and stratification according to smoking status (<10 pack years, ≥10 pack years) was performed. Patients in the radiotherapy arm were irradiated with a reduced radiation dose of 60 Gy, based on the study protocol of the Phase II NRG-HN002 trial [260] ORATOR 2 had to be terminated prematurely due to unacceptably high grade V toxicities in the TOS group [232].

The median follow-up time of the ORATOR 1 study in the most recent publication from 2022 [231] was 45 months. In the combined MDADI score (dysphagia), the functional outcome after radiotherapy was significantly better compared to TORS + ND, especially in the 1^st^ year after therapy. With longer follow-up (maximum 5 years), the difference decreased significantly, but was still statistically significant in favour of radiotherapy in 3 out of 5 sub-assessments and in the overall score. Clinically, however, the differences are to be classified as less significant, so that in view of the small number of cases, a superiority of radiotherapy cannot be classified as certain. The results show that primary transoral surgery or the primary non-surgical approach are relevant treatment options for stage I–II oropharyngeal carcinomas.

The study is viewed critically from a surgical perspective: in particular, the surgical technique is held causally responsible for the high complication rate of the ORATOR studies. Zech et al. have provided a valuable interpretation of this from a German ENT specialist perspective with a long-standing background in transoral laser microsurgery (TLM), which is quoted in more detail below [261]. The high complication rate of the ORATOR2 study (as well as the previous study ORATOR) [232], [236], [262] could be the result of the unusual operative technical approach of the surgeons from Canada and Australia, if one compares the surgical procedure with other studies. In particular, the safety margin of at least of at least 10 mm required in the study protocol does not correspond to the clinical standard and has already been criticised in several comments on the study [263], [264], [265]. Experiments on body donor specimens have shown that for radical tonsillectomies a safety margin of more than 2 mm is not possible due to the limited thickness of the upper pharyngeal constrictor muscle (Musculus constrictor pharyngis superior), which separates the tonsil bed from the carotid sheath [266]. MRI measurements of the muscle confirm a mean thickness of 2.4 mm (standard deviation=0.8 mm) [267]. In the currently recruiting “best-of” study EORTC-1420 (NCT02984410), this is taken into account, and for the deep tonsillar margin it is not the safety margin, but rather the integrity of the superior constrictor pharyngis muscle that is defined as a quality feature of an oncologically clean radical en bloc tonsillectomy in patients with low-stage oropharyngeal carcinoma [268]. The authors justify this with equivalent local control after resection with a safety margin of 3 mm compared to 5 mm in this localisation [269]. In the current NCCN (National Comprehensive Cancer Network) guidelines [136] oropharyngeal safety margins of even 1.5–2 mm (irrespective of HPV status) are sometimes considered acceptable, although the NCCN and this guideline generally recommend 5 mm as the current international consensus (see Chapter 6). In clinical practice, some ENT surgeons in the USA already opt for a wait-and-see approach [270], [271] after resection of small HPV-associated oropharyngeal carcinomas and a narrow resection margin just as frequently as for adjuvant/post-resection, as primarily recommended in the NCCN guidelines. However, prospective data on this type of de-escalation is still lacking, so that this discussion is only presented here, but cannot (yet) lead to a deviating recommendation in this guideline.

The safety margin of more than 1 cm required in the ORATOR2 study protocol requires resection with narrow resection margins, and the additional circumferential margin incisions therefore represent an exceptionally radical surgical procedure in comparison to standard clinical practice and in no way a common (consented) treatment concept. This impression is reinforced by the high rate of tracheotomies in the surgical arm (65%). In the analysis of transoral resections at the University Hospital of Cologne for T1-T4 HPV-positive and -negative oropharyngeal carcinomas, for example, a tracheostomy was only necessary in 12% of cases [272]. The combination of the supposedly carotid-approaching excisions and a ban on the use of regional and free flap plasty for defect coverage in the ORATOR studies therefore appears to be a very plausible explanation for the two surgical-related deaths (fulminant haemorrhage and cervical osteomyelitis) [273]. The German Society for Otorhinolaryngology, Head and Neck Surgery and the Working Group for Oncology therefore warn against uncritically transferring the results of the ORATOR2 study to clinical practice [261].

The unusually high rate of (post-)operative complications of transoral surgery in both ORATOR trials is verified by the results of the large Phase III trials “PATHOS” and EORTC-1420 (NCT02215265, NCT02984410), which are currently still recruiting and in which many German centres are also participating on the urgent recommendation of the IAG-KHT. In comparison to the ORATOR trials, operational quality standards and quality control measures were implemented in the study protocol of these trials [274]. In view of the planned number of over 1,000 patients, more reliable data on the morbidity and mortality of transoral surgery can be expected here.

The efforts of the ORATOR authors to minimise surgical risks and in particular the probability of bleeding by recommending ligation of the external carotid artery or its branches in laterally located tumours are to be acknowledged, as they can reduce post-operative bleeding rates, but are not generally required (although recommended in this guideline if there is good access to the relevant vessels during neck dissection). Laccourreye et al. reported 3.6% post-operative bleeding in 514 transorally operated patients with laterally located oropharyngeal tumours, of which 1.5% occurred at home after discharge from the hospital. The bleeding occurred predominantly in the first week, but also up to the third week and no longer after the fourth week. Prophylactic ligation of the external carotid artery or its branches as part of the primary procedure was not performed and was not recommended by the authors [275]. Salassa and Hinni [276] came to similar conclusions after a comprehensive retrospective study of 701 patients after transoral tumour surgery in the years 1996–2006, which showed a total of 1.4% postoperative bleeding between the 0^th^ and 17^th^ day. Three patients (0.4%) in this group had a catastrophic life-threatening haemorrhage, two of whom died. The main cause of haemorrhage was identified as the lingual artery in four cases, the superior laryngeal artery in two cases and the facial artery in two cases. In order to prevent potential bleeding after transoral tumour surgery, the authors recommend that in the event of bleeding occurring transorally, electrocaustic or vascular clips should be used first for arterial diameters over 2 mm. Prophylactic ligation of the tonsillar inflow from the external carotid artery or the external carotid artery itself is not recommended here either. A German study from 2004 by Esriti and Mann from the ENT University Clinic in Mainz investigated bleeding in the context of laser-surgical transoral tumour removal in the ENT area [277]. In the collective of 223 patients, a total of 97 oropharyngeal carcinomas were treated. Postoperative haemorrhage was observed in a total of 6% of these patients, with oropharyngeal carcinomas accounting for 10%. Severe secondary haemorrhage occurred in a total of five patients, of whom two required ligation of the external carotid artery and one who required ligation of the lingual artery. The Mainz colleagues also did not call for prophylactic ligation of the external carotid artery. Pollei et al. conducted a comprehensive analysis of a total of 906 patients who underwent transoral surgery for oropharyngeal carcinoma [278]. A postoperative haemorrhage rate of 5.4% was described here. 76.3% of these postoperative haemorrhages required surgical revision. Serious bleeding episodes were rare and occurred in 1.1% of patients. Prophylactic ligation of the external carotid artery was performed in 15.6% of patients. This was considered particularly necessary for advanced tumour stages. The rebleeding rate correlated with increasing tumour stage. The authors found that prophylactic ligation of the external carotid artery tributaries showed no advantage over patients who were not prophylactically ligated, although the rate of post-operative haemorrhage did not differ. Even in the group of prophylactically ligated patients, there was one case of life-threatening secondary haemorrhage. The authors conclude that due to the high risk of secondary haemorrhage, particularly in advanced tumour stages with intraoperatively exposed vessels in the case of a simultaneous neck dissection performed at the same time, the external carotid artery could be ligated. Although prophylactic ligation of the supplying vessels prior to transoral resection is not currently regarded as an absolute “must”, it is common practice in many hospitals in order to reduce the intraoperative and postoperative risk of haemorrhage.

Non-transoral conventional surgery with classic external approaches is currently being used less and less frequently for stage I–II tumours. Transmandibular approaches in particular are only very rarely required for the resection of oropharyngeal carcinoma due to the higher access morbidity in the context of the alternative access spectrum (transcervical lateral, suprahyoid). With regard to the individual surgical techniques of the primary tumour, reference is made to the existing surgical doctrines due to the high complexity of resection and reconstruction techniques [279], [280], [281], [282].

The question of “surgical therapy – de-escalation” for HPV-associated oropharyngeal carcinoma is currently unanswered.

#### 8.1.2 Radiotherapy

**8.11 Evidence-based recommendation 2024**

*The total dose of radiotherapy should be de-escalated for primary and adjuvant radiotherapy or radiochemotherapy for HPV/p16* ***positive*** *oropharyngeal carcinoma within clinical trials.*

- GoR: B
- LoE: 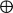

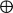

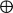

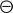
- [262], [263]
- Strong consensus

Radiotherapy has undergone significant technical development in the last 20 years. Intensity-modulated radiotherapy (IMRT) is available throughout Germany. Most institutes use rotation procedures (Volumetric Arc Therapy: VMAT) for IMRT, which are even better than IMRT with fixed irradiation angles at limiting the high-dose irradiated area to the actual target volume. The benefits of this new technology for patients with head and neck tumours have been well documented in a randomized study [264] and case series [265], [266], [267]. Compared to older 3D-conformal radiotherapy, IMRT reduces both acute (mucositis, xerostomia) and late side effects (fibrosis, xerostomia, swallowing function). The dose-response relationships for radiotherapy and their volume dependencies in relation to the long-term functional outcome, in particular swallowing function, are now well understood [268], [269], [270], so that further optimization of radiotherapy in terms of radiotherapy optimized for swallowing function (Swallowing Sparing IMRT) is becoming increasingly popular, now that a randomized study has also demonstrated the benefit compared to standard IMRT [271]. To implement this technique, it is important to contour the organs at risk [272], [273] and the target volumes [274], [275], [276] in accordance with current international standards. The recommended doses in the target volumes should not be undercut in order to better protect the organs at risk. IMRT techniques should be regarded as standard for both primary radio- or radiochemotherapy and adjuvant radio- or radiochemotherapy. High-quality cross-sectional imaging (CT or MRI) before the start of primary therapy is a prerequisite for high-quality radiation planning.

Interruptions of radiotherapy during primary or adjuvant radiotherapy beyond the planned breaks at weekends lead to a reduced effectiveness of radiotherapy due to repopulation of remaining tumour stem cells and should therefore be avoided [277], [278]. If there are nevertheless interruptions to radiotherapy, it is possible to make up for lost time by radiotherapy twice a day at intervals of >6 hours with an unchanged single dose. In contrast to increasing the total dose, this procedure does not lead to a higher probability of late side effects. Tumour stem cells are also repopulated in the interval between surgical resection and the start of radio- or radiochemotherapy. If the interval between tumour resection and the start of radiotherapy exceeds 6 weeks, a meta-analysis of the available data [279] shows a significant increase in relapse rates (odds ratio: 2.89; 95% VB 1.60–5.21). Suitable organizational measures must therefore be taken to ensure that this interval is not exceeded if possible.

#### 8.1.3 Primary radiotherapy ± combination with drug-based tumour therapy

**8.12 Evidence-based recommendation 2024**

*The primary non-surgical treatment of patients with HPV/p16-positive and -negative oropharyngeal carcinoma in stage T1 cN0 (UICC 8* *^th^* *edition) should be radiotherapy alone.*

- GoR: B
- LoE: 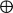

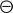

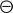

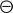
- [234]
- Strong consensus

**8.13 Evidence-based recommendation 2024**

*The primary non-surgical treatment for patients with oropharyngeal carcinoma in stage I cN1 (>1 LK *≤*3 cm) with HPV/p16+ or stage II (UICC 8^th^ edition) should be radiochemotherapy.*

- GoR: B
- LoE: 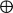

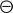

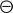

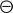
- [234], [235]
- Strong consensus

**8.14 Consensus-based recommendation 2024**

*In HPV/p16-positive and -negative oropharyngeal carcinoma, simultaneous chemotherapy should be cisplatin-based in the case of primary radiochemotherapy.*

*In patients who cannot receive cisplatin, e.g. due to impaired renal function, carboplatin + 5-FU, mitomycin C + 5-FU, a taxane or cetuximab (HPV/p16+) can be used as simultaneous systemic therapy.*

- EC
- Strong consensus

**8.15 Evidence-based recommendation 2024**

*Cetuximab should not be used in patients with* ***HPV/p16-positive*** *oropharyngeal carcinoma who do not have a contraindication to cisplatin-based chemotherapy due to its proven inferiority in terms of survival.*

- LoE: 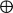

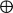

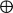

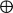
- [280], [281], [282]
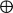

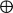

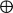

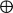
: Subgroup p16 positive
- Strong consensus

**8.16 Consensus-based recommendation 2024**

*Cetuximab should not be used in patients with* ***HPV/p16-negative*** *oropharyngeal carcinoma who do not have a contraindication to cisplatin-based chemotherapy due to its proven inferiority in terms of survival.*

- EC
- Strong consensus

**8.17 Consensus-based recommendation 2024**

*For primary radiotherapy or radiochemotherapy of HPV/p16-positive and HPV/p16-negative oropharyngeal carcinomas in stages I–III (UICC 8* *^th^* *edition), radiotherapy should be carried out with 5x2 Gy per week up to a target volume dose of 70 Gy in the area of the affected lymph nodes and the primary tumour or another established regimen with a biologically equivalent total dose.*

- EC
- Strong consensus

**8.18 Consensus-based recommendation 2024**

*In primary radiotherapy or radiochemotherapy of HPV/p16-positive and HPV/p16-negative oropharyngeal carcinomas, unaffected lymph node levels should be i* *r* *ra* *diated with 45–54 Gy with single doses of* *1.5 Gy–1.8 Gy* *.*

*The elective lymph node levels to be irradiated should be based on the current international consensus.*

- EC
- Strong consensus

**8.19 Consensus-based recommendation 2024**

*Primary radio- or radiochemotherapy of HPV/p16-positive* ***and HPV/p16-negative*** *oropharyngeal carcinomas should be carried out using the IMRT technique with the best possible protection of the salivary glands, the unaffected swallowing tract and the oral cavity, without falling below the recommended doses in the target volumes.*

- EC
- Strong consensus

##### Primary radiotherapy or radiochemotherapy for HPV/p16+ oropharyngeal carcinoma

After primary radiochemotherapy in patients with HPV/p16+ oropharyngeal carcinoma, randomized studies [234], [235], [280], [281], [282] and the large registry databases show 5-year survival rates of around 90% in stage I, around 80% in stage II and around 70% in stage III (8^th^ UICC classification). The vast majority of patients received conventionally fractionated or slightly accelerated radiotherapy with 70–72 Gy within 6–7 weeks in combination with cisplatin-containing simultaneous chemotherapy.

The good results, particularly in stages I–II, have raised the question of whether it is possible to de-escalate the therapy without compromising survival in order to reduce the toxicity of the therapy. Various strategies have been tested: in 3 larger randomized studies [280], [281], [282] it was tested whether a weekly administration of cetuximab is as effective as 3 applications of 100 mg/m² cisplatin (2 studies) or 6–7 weekly applications of 40 mg/m² cisplatin for radiotherapy. The studies showed a significant and clinically relevant inferiority of cetuximab in survival and locoregional relapse rate. In addition, treatment with cetuximab was no less toxic overall than cisplatin. Thus, the simultaneous administration of cisplatin and radiotherapy remains the standard therapy for HPV/p16+ oropharyngeal carcinoma.

Yom et al. [260] compared in a randomized phase II study in HPV/p16+ oropharyngeal carcinomas in study I-II (8^th^ UICC classification) a single slightly accelerated intensity-modulated radiotherapy (60 Gy in 5 weeks) with a conventional fractionated intensity-modulated radiotherapy (60 Gy in 6 weeks) in combination with weekly 40 mg/m² cisplatin. A significantly higher locoregional relapse rate was found in the arm with radiotherapy alone. There were no differences in all other oncological endpoints, including toxicity. The registry data [234], [235] showed that in HPV/p16+ oropharyngeal carcinomas in stages I–II, survival was also significantly worse if concurrent chemotherapy was not administered during radiotherapy. Only in patients without lymph node involvement was radiotherapy alone as effective as radiochemotherapy in stages I–II [234], [239]. This opens up a certain scope for saving simultaneous chemotherapy in stages I cN0, although data from randomized studies are still pending.

In two small phase II studies, induction chemotherapy was initially applied to HPV/p16+ oropharyngeal carcinomas in stages II and III. If there was a good response to chemotherapy, the total doses of subsequent radio- or radiochemotherapy were reduced by 16–25 Gy. The PFS in patients treated with reduced doses was still above 80% after 3 years in both studies. However, due to the small case numbers of n=20 [262] and n=62 [283], no treatment recommendations can be derived from this.

In summary, de-escalation of the total dose of radiotherapy or simultaneous therapy with cisplatin in stages I–III of HPV/p16+ oropharyngeal carcinoma remains a study question. According to the 3 larger randomized studies (1,321 patients in total), which exclusively included HPV/p16+ patients with oropharyngeal carcinoma in stages I–III (8^th^ UICC classification), conventionally fractionated radiotherapy with 5x2 Gy per week up to a total dose of 70 Gy in IMRT technique in combination with 3x100 mg/m² cisplatin or 40 mg/m² weekly cisplatin parallel to radiotherapy is the best investigated treatment concept. In other studies, slightly accelerated fractionations of radiotherapy with 70–72 Gy within 6 weeks were also used with good results [7]. However, the benefit of accelerated radiotherapy in HPV/p16+ patients with oropharyngeal carcinoma is uncertain.

For patients with oropharyngeal carcinoma who are not suitable for cisplatin, e.g. due to impaired renal function, there are results from a number of randomized studies that tested carboplatin in combination with 5-FU [284], [285], [286], [287], [288], mitomycin C in combination with 5-FU [289], [290] or weekly docetaxel [291] simultaneously with radiotherapy compared to radiotherapy alone. In some of these studies, some patients with tumours of the oral cavity, larynx and hypopharynx were also included. For both combination chemotherapies applied simultaneously with radiotherapy, a significant advantage was shown in terms of survival and locoregional tumour control. Only studies with ≤80 patients in the radiochemotherapy arm are available for monochemotherapy with carboplatin [286], [292], [293], [294], and there were no significant advantages for monotherapy with mitomycin C [295], [296], [297] in parallel with radiotherapy. The HPV/p16 status of the oropharyngeal carcinomas was not determined in any of these studies, so it is unknown whether the benefit shown also depends on the HPV/p16 status of the tumours. Weekly applications of carboplatin and paclitaxel, concurrently with radiotherapy, have been investigated in a number of non-randomized phase II trials and some retrospective cohort studies [298], [299], [300], [301], [302], [303], [304]. Oropharyngeal carcinomas represented the main group of treated squamous cell carcinomas of the head and neck, with no differentiation made according to HPV/p16 status. The non-randomized retrospective studies indicate a similar efficacy of carboplatin + paclitaxel compared to weekly therapy with 40 mg/m² cisplatin in parallel with radiotherapy. In contrast, there is very little data available for the administration of paclitaxel alone [305]. Cetuximab in combination with radiotherapy alone was tested in a randomized study [306] with radiotherapy alone for squamous cell carcinoma of the oropharynx (63%), larynx and hypopharynx. A significant advantage in overall survival and in the locoregional recurrence rate was demonstrated. The p16 status was retrospectively determined in 182 oropharyngeal carcinomas (66%). This showed that the benefit of cetuximab was largely limited to the p16 positive tumours [307].

##### Primary radio- or radiochemotherapy for HPV/p16– oropharyngeal carcinoma

Results of randomized studies on the effect of chemotherapy applied in addition to radiotherapy for patients with exclusively HPV/p16– oropharyngeal carcinomas are not yet available, as simultaneous radiochemotherapy was already used as standard therapy before the different biology of HPV/p16+ oropharyngeal carcinomas was known. An evaluation of these studies in oropharyngeal carcinomas was therefore not carried out. Radiotherapy was compared with the combination of radiotherapy and chemotherapy in a total of 107 randomized studies (19,805 patients). The latest update of the MACH-NC meta-analysis [308] from 2021 shows that only simultaneous radiochemotherapy leads to an improvement in overall survival and PFS, whereas neither induction chemotherapy nor adjuvant chemotherapy lead to a benefit in this regard. In 58 randomized trials, radiotherapy was compared with simultaneous radiochemotherapy in 14,401 patients. The vast majority of these patients had received simultaneous chemotherapy containing cisplatin. There was an absolute survival benefit of 6.5% (95% CL 4.6–8.4%) after 5 years and 3.6% (95% CL 1.8–5.4%) after 10 years. 34.7% of these patients had oropharyngeal carcinomas. The benefit of simultaneous chemotherapy applied in addition to radiotherapy was the same for oropharyngeal carcinoma (HR 0.82) as for squamous cell carcinoma of the larynx (HR 0.81), hypopharynx (HR 0.88) and oral cavity (HR 0.82) [308]. It is therefore assumed that the benefit of simultaneous chemotherapy is the same for HPV/p16– and HPV16+. The available registry data confirm this assessment [309]. This applies in particular to HPV/p16– oropharyngeal carcinomas in stages III–IVb, whereas the benefit of additional simultaneous chemotherapy in stages I–II is not well documented [309]. It should be noted that only 9.1% of patients in the MACH-NH meta-analysis were older than 70 years, only 5.4% had an ECOG ≥2 and only 5.4% were treated in stages I–II (UICC 7^th^ edition). While in the MACH-NH meta-analysis patients over 70 years of age did not benefit from simultaneous chemotherapy, the NCDB and SEER registry databases also documented survival benefits in patients over 70 years of age [310], [311]. Excluding this patient group in the absence of contraindications for simultaneous chemotherapy therefore does not appear justified.

For patients with a contraindication to cisplatin, randomized studies have also demonstrated a survival benefit for the simultaneous application of carboplatin and 5-FU [284], [285], [286], [287], [288] or mitomycin and 5-FU [289], [290] or weekly docetaxel [291] simultaneously with radiotherapy compared to radiotherapy alone. Only studies with ≤80 patients in the radiochemotherapy arm are available for monotherapy with carboplatin [292], [293] or mitomycin C [286], [294], [295], [296], and there were no significant advantages for monotherapy with mitomycin C [295], [296], [297] in parallel with radiotherapy. Weekly applications of carboplatin and paclitaxel concurrently with radiotherapy have been investigated in a number of non-randomized phase II trials and some retrospective cohort studies [298], [299], [300], [301], [302], [303], [304]. Oropharyngeal carcinomas represented the main group of treated squamous cell carcinomas of the head and neck, with no differentiation made according to HPV/p16 status. The non-randomized, retrospective comparative studies indicate a similar efficacy of carboplatin + paclitaxel compared to weekly therapy with 40 mg/m² cisplatin in parallel with radiotherapy. In contrast, there is very little data available for the administration of paclitaxel alone [305]. A benefit of cetuximab in combination with radiotherapy for HPV/p16 oropharyngeal carcinoma cannot be derived from the available data [306], [307], [312].

As with HPV/p16 negative oropharyngeal carcinomas, radiotherapy should be carried out using the IMRT technique with the best possible protection of the salivary glands, the swallowing tract and the oral cavity, without reducing the dose in the target volumes. The standard fractionation for simultaneous chemotherapy is conventional fractionation with 5x2 Gy per week up to a target volume dose of 70 Gy. Accelerated radiotherapy with a reduction of the total treatment time to 6 weeks or less has not shown any benefit in combination with simultaneous chemotherapy according to the results of 2 randomized studies [285], [313]. Hyperfractionated radiotherapy with 2 fractions per day 5 times per week with single doses of 1.2–1.25 Gy up to total doses of 74.4–80.4 in combination with simultaneous chemotherapy were compared with hyperfractionated radiotherapy alone in several randomized trials [314], [315], [316]. The additional chemotherapy also improved survival and locoregional tumour control in combination with hyperfractionated radiotherapy. In the randomized studies on radiotherapy alone, which compared different fractionations of radiotherapy, only hyperfractionated radiotherapy proved to be significantly superior in terms of overall survival, as described in the above-mentioned studies, whereas accelerated radiotherapy regimens only improved locoregional tumour control but did not result in a survival benefit [317]. To date, hyperfractionated radiotherapy in combination with simultaneous chemotherapy has not been compared with conventionally fractionated radiotherapy with simultaneous chemotherapy. Thus, it remains unclear whether hyperfractionated radiotherapy with simultaneous chemotherapy may be even more effective than conventionally fractionated radiotherapy in combination with simultaneous chemotherapy, as predicted by a network meta-analysis [318]. In the case of radiotherapy alone with contraindications to concurrent chemotherapy, hyperfractionated radiotherapy is a good option, although it is not certain whether patients with poor general health or very old patients who are not suitable for concurrent chemotherapy will benefit from hyperfractionated radiotherapy. It should be noted in all the studies on fractionated radiotherapy that only about one third of the patients had oropharyngeal carcinomas and their HPV/p16 status was unknown. However, the studies showed no evidence of different effects of fractionation between squamous cell carcinomas of the oropharynx, hypopharynx, larynx and oral cavity.

#### 8.1.4 Adjuvant radiotherapy ± combination with drug-based tumour therapy

**8.20 Consensus-based recommendation 2024**

*Post-operative radiotherapy should be started as soon as possible after the wound has healed and within* *6 weeks* *of the operation.*

- EC
- Strong consensus

**8.21 Consensus-based recommendation 2024**

*Patients with HPV/p16 positive oropharyngeal carcinoma in stages I, II (UICC 8* *^th^* *edition) who have undergone primary surgical treatment should receive adjuvant radio/radiochemotherapy if*

*– R1 or <5 mm resection has been performed in healthy tissue or*

*– solitary lymph node >3 cm or*

*– more than one tumour-involved lymph node or*

*– ≥1 lymph node with ECS has been histologically proven.*

- EC
- Strong consensus

**8.22 Consensus-based recommendation 2024**

*In patients with* ***HPV/p16-negative*** *oropharyngeal carcinoma in stages pT1, pT2 N0 (UICC 8* *^th^* *edition) who have undergone primary surgical treatment, adjuvant radio/radiochemotherapy should be given if resection R1<5 mm has been performed in healthy tissue.*

- EC
- Strong consensus

**8.23 Consensus-based recommendation 2024**

*In pT1-pT2 pN0 (M0) HPV/p16-positive* ***and -negative*** *oropharyngeal carcinoma, adjuvant radiotherapy should be dispensed with if the resection >5 mm has been performed in healthy tissue.*

- EC
- Strong consensus

**8.24 Consensus-based recommendation 2024**

*In pT1-pT2 (M0) HPV/p16-positive and HPV/p16-negative oropharyngeal carcinoma with only one affected lymph node <3 cm (without ECS), adjuvant radiotherapy can be dispensed with if all of the following criteria are met:*

*– G1-G2 (HPV/p16 neg.)*

*– L0*

*– V0*

*– Pn0*

*– R0>5 mm*

- EC
- Strong consensus

**8.25 Consensus-based recommendation 2024**

*Adjuvant irradiation of the region with affected lymph node levels without capsular rupture in HPV/p16-positive and HPV/p16-negative oropharyngeal carcinomas should be carried out with 54–60 Gy in single doses of 1.8–2.0 Gy*

*and*

*adjuvant irradiation of affected lymph node levels with capsule breakthrough should be carried out with 66 Gy in single doses of 2.0–2.2 Gy.*

- EC
- Strong consensus

**8.26 Consensus-based recommendation 2024**

*Adjuvant irradiation of unaffected lymph node levels in HPV/p16-* ***positive*** *oropharyngeal carcinomas should* *be carried out* *with 45–54 Gy with single doses of* *1.5–1.8 Gy* *.*

*The elective lymph node levels to be irradiated should be based on the current international consensus.*

- EC
- Strong consensus

**8.27 Consensus-based recommendation 2024**

*If adjuvant radiochemotherapy is indicated for HPV/p16-positive or HPV/p16-negative oropharyngeal carcinoma, cisplatin-based chemotherapy should be administered simultaneously with radiotherapy.*

*In patients who cannot receive cisplatin, e.g. due to impaired renal function, mitomycin C + 5-FU, carboplatin + 5-FU or docetaxel can be used as simultaneous systemic therapy.*

- EC
- Strong consensus

##### Postoperative radiotherapy

The effect of postoperative radiotherapy compared to no adjuvant radiotherapy has not been investigated in randomized studies. However, the results of cohort studies and prospective registry data showed a very clear superiority of adjuvant radiotherapy in patients with more than one tumour-involved lymph node, extracapsular tumour growth at the lymph nodes (ECE) or with tumours only barely resected in healthy tissue (<5 mm), so that randomized studies were not conducted [319], [320], [321], [322]. These studies included squamous cell carcinomas of the larynx, oropharynx, hypopharynx and oral cavity. The HPV/p16 status was not known in any of the collectives examined. The collectives studied were mainly treated in Western countries between 1970 and 1998 and in Asia in the period 1990–2008. Based on the available data on the low incidence of HPV/p16+ oropharyngeal carcinomas in these countries during the study period, it can be assumed that the oropharyngeal carcinomas in these studies were predominantly HPV/p16– [323], so that these data essentially apply to HPV/p16– oropharyngeal carcinomas. Soliman et al. [324] analysed the data of 15,036 patients with HPV/p16+ oropharyngeal carcinoma who were documented in the National Cancer Database (USA) between 2010 and 2017 and who had received adjuvant radiotherapy or no adjuvant radiotherapy after primary surgical treatment, predominantly in stages I and II. Patients who had received adjuvant radiochemotherapy were excluded. In the “Propensity Score Matched” analysis, adjuvant radiotherapy led to a significant survival benefit of approximately 8% in absolute terms after 5 years in the overall collective and 10% for patients with risk factors (R1 resection, ECE, LVI, blood vessel invasion). Overall survival with postoperative radiotherapy was 90% and 87% after 5 years in the overall collective and in patients with risk factors, respectively, which is significantly higher than the values reported in the old data for patients with oropharyngeal carcinoma with unknown HPV/p16 status [319], [320], [321], [322]. The available data suggest that adjuvant radiotherapy reduces the locoregional recurrence rate and improves survival in oropharyngeal carcinoma regardless of HPV/p16 status, provided that more than one regional lymph node is affected or the primary tumour was only barely resected in healthy tissue (<5 mm). For patients with pT3/pT4 tumours, lymph node involvement with ECE or R1 resection, there are no reliable data on treatment outcomes without adjuvant radiotherapy for HPV/p16+ oropharyngeal carcinomas, as there is an international consensus that these patients, just like those with HPV/p16 tumours, require adjuvant radiotherapy [325].

##### Postoperative radiochemotherapy

The value of additional cisplatin-containing chemotherapy administered simultaneously with radiotherapy was investigated in 3 larger randomized studies, which included patients with squamous cell carcinoma of the oropharynx, hypopharynx, larynx and oral cavity [326], [327], [328]. HPV/p16 status was not determined in these studies. However, for the reasons mentioned above, it can also be assumed that the majority of oropharyngeal carcinomas in these studies were HPV/p16–. The results of the studies consistently showed that patients with evidence of ECE in the affected lymph nodes or R1 resection (defined in these studies as resection <5 mm in healthy tissue) had a survival benefit from adjuvant radiochemotherapy compared to adjuvant radiotherapy alone. In an analysis based on the NCDB registry [329], the results of adjuvant radiochemotherapy were compared with those of adjuvant radiotherapy alone in 1,127 patients with HPV/p16+ and 424 patients with HPV/p16– oropharyngeal carcinoma who had intermediate risk factors for recurrence (at least one of the following factors: pT3-T4, ≥2 tumour-involved lymph nodes, involved lymph nodes in level IV or V, lymph vessel invasion). In the “Propensity Score Matched” cohorts of HPV/p16+ and HPV/p16– tumours, no benefit in terms of survival was shown for the additional platinum-containing chemotherapy. Other endpoints were not investigated. Thus, in the presence of these intermediate risk factors, adjuvant radiotherapy alone is preferable.

Although lymph node involvement with ECE in HPV/p16+ oropharyngeal carcinomas is not taken into account in the N classification and staging, the results from various registry studies show that the detection of ECE in the lymph nodes of oropharyngeal carcinomas is associated with a significantly higher recurrence rate and poorer survival regardless of HV/p16 status [330], [331], [332], [333]. However, if adjuvant radio- or radiochemotherapy has been applied to HPV/p16+ oropharyngeal carcinomas, the recurrence rate in the presence of ECE is only minimally increased and only detectable in larger collectives. The available data from the registry studies consistently show no benefit of adjuvant radiochemotherapy compared to adjuvant radiotherapy alone [330], [331], [332]. The results of randomized studies are not available. Data on the relapse pattern after adjuvant radiotherapy or radiochemotherapy for HPV/p16+ oropharyngeal carcinoma indicate that the poorer prognosis in patients with ECE to the lymph nodes is almost exclusively due to a higher rate of distant metastasis [334]. Apparently, the intensity of the platin-containing chemotherapy applied simultaneously with radiotherapy is not sufficient to reduce distant metastasis. The available registry data therefore indicate that adjuvant radiotherapy alone may be sufficient for HPV/p16+ oropharyngeal carcinoma, even in cases of lymph node involvement with evidence of ECE, and that additional chemotherapy may not be necessary.

In contrast, the available registry data show a clear advantage for platinum-containing chemotherapy applied simultaneously with adjuvant radiotherapy for HPV/p16– oropharyngeal carcinomas with evidence of ECE in the lymph nodes [330], [331], [332].

Less well documented is the influence of positive (<1 mm) or narrow resection margins (<5 mm) on the recurrence rate and survival of oropharyngeal carcinomas depending on HPV/p16 status. The available data from registry studies show a greater negative impact on the recurrence rate and survival for HPV/p16 oropharyngeal carcinomas than for HPV/p16+ tumours [333]. Regardless of HPV/p16 status, the recurrence rates do not differ significantly in the case of positive (<1 mm) compared to narrow (<5 mm) resection margins, even though a trend in favour of larger resection margins can be seen [335], [336]. Only with tumour-free resection margins of ≥5 mm do the differences become larger and in some cases statistically significant, provided that larger collectives were examined [321], [335]. Regardless of whether no adjuvant therapy or adjuvant radio- or radiochemotherapy has been carried out, the treatment results for HPV/p16+ oropharyngeal carcinomas are significantly better in terms of the recurrence rate and survival [333], [335]. In a large collective of NCDB [337] patients with HPV/p16+ oropharyngeal carcinoma with a proven positive resection margin as well as when ECE was detected in the affected lymph nodes or when both risk factors were present, survival did not differ whether the patients had received adjuvant radiotherapy alone or radiochemotherapy. The results of randomized studies are not available. In summary, the benefit of adjuvant radiochemotherapy compared to adjuvant radiotherapy alone for positive or close resection margins in HPV/p16+ oropharyngeal carcinomas in contrast to HPV/p16– oropharyngeal carcinomas cannot be considered certain. However, patients with HPV/p16+ oropharyngeal carcinomas with locally advanced disease (pT3-4 or pN2-3) who also had ECE resected at the affected lymph nodes or only just in healthy tissue were significantly underrepresented in the previously described collectives, so that a relevant benefit of adjuvant radiochemotherapy cannot be ruled out in these cases. If postoperative radiochemotherapy is indicated, it should be noted that results of randomized studies are only available for cisplatin or cisplatin +5-FU, which have shown a significant reduction in the relapse rate or an improvement in survival [326], [327], [328]. In a randomized Japanese study (n=261), 3x100 mg/m² cisplatin at 3-week intervals was compared with weekly 40 mg/m² cisplatin simultaneously with postoperative radiotherapy [338]. The non-inferiority of the weekly administration of cisplatin was demonstrated. However, the proportion of patients with oropharyngeal carcinoma (11%) was small.

For patients who cannot receive cisplatin, e.g. due to impaired renal function, there is little data available for postoperative radiochemotherapy. For mitomycin C, data from two non-randomized studies are available, which only showed a trend towards a survival benefit [297], [339]. The weekly administration of docetaxel simultaneously with radiotherapy was compared with radiotherapy alone in a randomized study [291]. In a small subgroup of this study, the therapy was also given in the postoperative situation. There was a trend towards improved survival for the combined therapy. For carboplatin + 5-FU and carboplatin + paclitaxel, no results of randomized studies in the postoperative situation are available. However, it seems plausible to use these combinations in the postoperative situation by analogy with the efficacy of these combinations in primary radiochemotherapy (see 8.3.1). There are no data suggesting a benefit for the use of cetuximab in the postoperative situation.

For patients with an intermediate risk of recurrence (no ECE and resection ≥5 mm in healthy tissue), 60 Gy (5x2 Gy per week) was applied in the region of the former primary tumour and the affected lymph nodes in the vast majority of patients in the clinical trials and the registry data, regardless of HPV/p16 status. In the neighbouring unaffected lymph node regions, 45–50 Gy with 5x1.8–2.0 Gy per week were administered electively. There is an international consensus for the selection of the lymph node levels to be electively irradiated, which can be regarded as a standard guideline [276]. For HPV/p16+ oropharyngeal carcinomas, lower doses of 50 Gy in conventional fractionation [263] or only 30 Gy with 2x1.5 Gy per day were also investigated in smaller phase II studies [340], provided there was an R0 resection and no ECE at the lymph nodes. In the phase II study mentioned [340] 208 patients were randomized between 60 Gy and 50 Gy. The PFS after 3 years was 90% with 50 Gy as well as with 60 Gy. In the single-arm study with only 30 Gy, the PFS was also just under 90%. However, the number of cases in the studies mentioned is still too small to be able to generally recommend a dose reduction. In patients with a high risk of relapse (ECE or resection <5 mm in healthy or R1), 36 Gy with 1.8 Gy twice daily in combination with docetaxel was applied in 22 patients in the study by Moore et al. [340], without a noticeably increased relapse rate being reported. In contrast, in the study by Ferris et al. [263], 66 Gy was given in conventional fractionation in combination with simultaneous cisplatin with a high risk of relapse. De-escalation of the radiation dose below the recommended dose of 66 Gy (5x2 Gy per week) remains the recommended standard in the high-risk situation regardless of HPV/p16 status. For elective adjuvant irradiation of neighbouring, unaffected lymph node sites, 45–50 Gy (5x1.8–2.0 Gy per week) is also recommended in the high-risk situation. IMRT techniques are also considered standard for postoperative radiotherapy.

#### 8.1.5 Neoadjuvant and adjuvant drug-based tumour therapy

Neither induction concepts nor adjuvant, purely drug-based therapy concepts are generally recommended for the primary treatment of oropharyngeal carcinoma. In large meta-analyses, no advantages could be generated by adding these concepts. In the future, depending on the development of the promising study situation, it is conceivable that neoadjuvant and adjuvant concepts will find their way into the clinical practice of primary therapy through the use of checkpoint inhibitors [341], [342], [343]. 

**8.28 Consensus-based recommendation 2024**

*Neoadjuvant or adjuvant systemic therapy should only be used for primary surgical therapy or definitive radio- or radiochemotherapy for HPV/p16-positive* ***and HPV/p16-negative*** *oropharyngeal carcinomas within clinical trials.*

- EC
- Strong consensus

### 8.2 Oropharyngeal carcinomas in the UICC stages III p16-positive: T1N3-T4; stage III and IV-A, -B p16-neg.: T3-TxN3, M0)

**8.29 Evidence-based statement 2024**

*There is evidence that patients with HPV/p16-positive stage III oropharyngeal carcinoma (UICC 8* *^th^* *edition) have better overall survival and relapse-free survival after primary surgical therapy followed by adjuvant radiotherapy or radiochemotherapy compared to primary radiochemotherapy.*

- LoE: 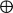

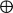

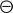

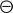
- [235], [344]
- Strong consensus

**8.30 Evidence-based recommendation 2024**

*Patients with HPV/p16-positive stage III or HPV/p16-negative stage III–IVb oropharyngeal carcinoma (UICC* *8* *^th^* *e* *dition) should be treated with primary surgical therapy followed by adjuvant radiotherapy or radiochemotherapy if a good functional outcome and R0 resection are likely to be achievable.*

*Otherwise, these patients should receive primary r* *ad* *io* *ch* *emother* *apy.*

- GoR: B
- LoE: 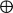

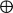

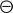

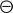
- [245], [345]
- Strong consensus

**8.31 Evidence-based recommendation 2024**

*In patients with HPV/p16-positive* ***and HPV/p16-negative*** *oropharyngeal carcinomas who are not treated with surgery, primary radiochemotherapy should be preferred to radiotherapy alone, especially in the age group up to 7* *0 y* *ears.*

- GoR: A
- LoE: 1a
- [2], [346], [347]
- 1a: S3 guideline adaptation – Laryngeal Carcinoma, Version 1.1 2019 (7.13)
- Strong consensus

#### 8.2.1 Surgical therapy

Surgical treatment was described in more detail in Chapter 8.1.1. With a particular focus on advanced oropharyngeal carcinomas (p16-pos. stage III, p16-neg. stage III and IVa), there is a complete lack of randomized studies that directly compare primary surgical treatment with radiochemotherapy. In this respect, registry studies are helpful for classification, although a lower level of evidence must be assumed. Cheraghlou et al. ([235]; Evidence Table PICO 2, registry-based studies) conducted a registry study with 4,443 patients with HPV-positive oropharyngeal carcinoma of all tumour stages based on the National Cancer Data Base (NCDB, data from 2009–2013), classified according to the current 8^th^ TNM edition. It should be noted that this study did not include data on smoking status and the surgical procedures used, but the stage-dependent modality comparison was one of the best results. In patients with stage III disease (n=450), triple-modality therapy (surgery + adj. radiochemotherapy) was associated with significantly improved survival (HR 0.52; P=0.024) compared to treatment with definitive radiochemotherapy alone. The 3-year overall survival of stage III patients after radiochemotherapy was 71.9% and after surgery + adjuvant radiochemotherapy 84.9%. Similar results in favour of the trimodal approach were also observed for advanced p16-negative oropharyngeal carcinoma (retrospective cohort with 131 patients; [246]).

Amini et al. also conducted a registry study from the NCDB (2009–2011) with 3,952 patients (2,454 p16-pos.) and a somewhat less conclusive focus [348]. The T3-T4 proportion was given as 20.8% and the reference group was surgical therapy alone (rarely relevant). All bi- and trimodal approaches were better than surgery alone, which was only comparable to radiotherapy alone. It was concluded that bimodal therapies appeared to be beneficial in HPV-positive oropharyngeal cancer. In HPV-negative patients, postoperative chemotherapy with radiotherapy was associated with an improvement in overall survival, while no significant benefit was observed in HPV-positive patients ([348]; conclusion PICO 3 evaluation). Kamran et al. ([344]; Evidence Table PICO-3) conclude in a registry study (NCDB 2004–2013; 22,676 patients; subset 6,872 with HPV status; 73.3% HPVpos.; 31.7% T3-T4) of a propensity-matched cohort for HPV-positive patients that patients treated primarily surgically have a better 3-year survival, especially in the subgroup of (HPV)-negative patients (p=0.06), than after primary radiochemotherapy. For HPV-positive status, this difference was not significant (p=0.38). Other registry studies presented in the PICO-3 Table ([238], [239], [349], [350]) came to a similar conclusion.

In summary, based on the registry studies for stage III and IVa, p16-negative oropharyngeal carcinomas, the majority of authors favour primary surgery followed by adjuvant radiotherapy or radiochemotherapy as the treatment of first choice. For p16-positive. The registry studies do not provide a uniform picture for p16pos. patients, although the largest registry study of 450 HPV-positive stage III patients [235] shows a significant superiority of primary surgery + adjuvant radiochemotherapy. Since all registry studies did not adjust for smoking status, the current data situation should be evaluated with the necessary caution. Overall, the primary surgical approach is currently to be favoured in the patient group addressed, provided that R0 resection is possible and a meaningful functional outcome can be expected. Information on the respective surgical techniques has already been provided in Section 8.1.1.

#### 8.2.2 Radiotherapy

With regard to the accompanying text on radiotherapy in general, please refer to the explanations in Section 8.1.2.

**8.32 Evidence-based recommendation 2024**

*De-escalation of the total dose of radiotherapy should be carried out for primary and adjuvant radiotherapy or radiochemotherapy for* ***HPV/p16-positive*** *oropharyngeal carcinoma within clinical trials.*

- GoR: B
- LoE: 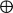

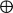

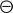

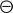
- [262], [351]
- Strong consensus

#### 8.2.3 Primary radiotherapy ± combination with drug-based tumour therapy

For information on primary radiotherapy and radiochemotherapy, please refer to the accompanying text, Section 8.1.3.

**8.33 Evidence-based recommendation 2024**

*The primary conservative therapy for patients with HPV/p16-positive stage III and HPV/p16-negative stage III–IVb oropharyngeal carcinoma (UICC 8* *^th^* *edition) should be radiochemotherapy.*

- GoR: A
- LoE: 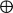

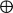

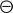

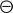
, 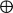

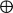

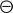

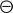
- [234], [235], [309]
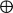

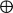

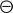

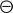
: In the HPV/p16 negative subgroup
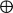

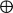

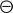

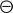
: In the HPV/p16 positive subgroup
- Strong consensus

**8.34 Evidence-based recommendation 2024**

*In patients with HPV/p16-positive* ***stage III and HPV/p16-negative stage III–IVb*** *oropharyngeal carcinoma (UICC* *8* *^th^* *e* *dition), locoregional tumour control and overall survival are statistically significantly better after primary radiochemotherapy than after radiotherapy alone.*

- GoR: A
- LoE: 1a, 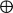

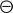

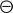

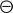
- [2], [235], [346], [347], [352], [353]
- 1a: In the subgroup p16 negative (S3 guideline adaptation – Laryngeal Carcinoma, Version 1.1 2019 (7.9))
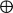

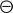

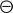

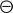
: In the HPV/p16 positive subgroup
- Strong consensus

**8.35 Evidence-based recommendation 2024**

*In* ***HPV/p16-positive*** *oropharyngeal carcinoma, simulta* *n* *e* *ous chemotherapy should be cisplatin-based in the case of primary radiochemotherapy.*

- GoR: A
- LoE: 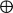

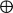

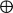

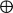
- [280], [281], [282]
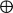

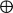

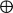

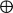
: In the HPV/p16 positive subgroup
- Strong consensus

**8.36 Consensus-based recommendation 2024**

*In* ***HPV/p16-negative*** *oropharyngeal carcinoma, simulta* *n* *e* *ous chemotherapy should be cisplatin-based in the case of primary radiochemotherapy.*

- EC
- Strong consensus

**8.37 Consensus-based recommendation 2024**

*In patients who cannot receive cisplatin due to impaired renal function, for example, carboplatin + 5-FU, mitomycin C + 5-FU, a taxane or cetuximab (HPV/p16+) can be used as simultaneous systemic therapy.*

- EC
- Strong consensus

**8.38 Evidence-based recommendation 2024**

*Cetuximab should not be used for HPV/p16-positive* ***and HPV/p16-negative*** *oropharyngeal carcinomas that have no contraindication to cisplatin-based chemotherapy due to its proven inferiority in terms of survival.*

- GoR: A
- LoE: 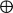

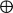

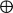

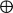
, 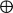

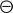

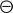

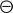
- [280], [281], [282], [307], [354]
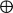

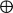

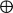

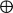
: In the HPV/p16 positive subgroup
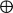

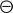

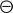

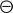
: In the HPV/p16 negative subgroup
- Strong consensus

**8.39 Consensus-based recommendation 2024**

*For primary radiotherapy or radiochemotherapy of HPV/p16-positive and HPV/p16-negative oropharyngeal carcinomas in stages I–III (UICC 8* *^th^* *edition), radiotherapy should be carried out with 5x2 Gy per week up to a target volume dose of 70 Gy in the area of the affected lymph nodes and the primary tumour or another established regimen with a biologically equivalent total dose should be used.*

- EC
- Strong consensus

**8.40 Consensus-based recommendation 2024**

*In primary radiotherapy or radiochemotherapy of HPV/p16-positive* ***and -negative*** *oropharyngeal carcinomas, irradiation of non-affected lymph node levels should be carried out with 45–54 Gy with single doses of 1.5–* *2 Gy* *.*

*The elective lymph node levels to be irradiated should be based on the current international consensus *[276]*.*

- EC
- Strong consensus

**8.41 Consensus-based recommendation 2024**

*For* ***HPV/p16-negative*** *oropharyngeal carcinomas, the dose should be between 50 Gy and 60 Gy with individual doses of 1.5 to 2.0 Gy, depending on the risk, in the lymph node levels to be electively irradiated.*

- EC
- Strong consensus

**8.42 Consensus-based recommendation 2024**

*Primary radio- or radiochemotherapy of HPV/p16-positive* ***and HPV/p16-negative*** *oropharyngeal carcinomas in stages III (HPV/p16 positive) or III–IVb (HPV/p16 nega* *t* *i* *ve) should be carried out using the IMRT technique with the best possible protection of the salivary glands, the unaffected swallowing tract and the oral cavity, without falling below the recommended doses in the target volumes.*

- EC
- Strong consensus

#### 8.2.4 Adjuvant radiotherapy ± combination with drug-based tumour therapy

With regard to adjuvant therapy, please refer to the accompanying text, Section 8.1.4.

**8.43 Consensus-based recommendation 2024**

*Post-operative radiotherapy should be started as soon as possible after the wound has healed and within* *6 weeks* *of the operation.*

- EC
- Strong consensus

**8.44 Evidence-based recommendation 2024**

*Patients with HPV/p16-positive* ***stage III and HPV/p16-negative stage III–IVb*** *oropharyngeal carcinoma (UICC* *8* *^th^* *e* *dition) who have undergone primary surgical treatment should receive adjuvant radiotherapy or radiochemotherapy.*

- GoR: A
- LoE: 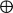

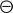

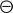

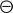
- [319], [348]
- Strong consensus

**8.45 Evidence-based recommendation 2024**

*Adjuvant radiochemotherapy after primary surgical treatment of HPV/p16-positive* ***and HPV/p16-negative*** *oropharyngeal carcinomas in stage III or III–IVb should be given if the resection <5 mm has been performed in healthy tissue or if extracapsular tumour growth in one or more lymph nodes has been histologically proven.*

- GoR: B
- LoE: 1b, 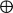

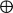

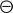

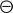
- [2], [326], [327], [328], [330], [331], [332], [355]
- 1b: S3 guideline adaptation – Laryngeal Carcinoma, Version 1.1 2019 (7.39)
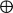

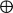

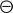

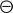
: In subgroup HPV/p16 positive
- Strong consensus

**8.46 Consensus-based recommendation 2024**

*Adjuvant irradiation of unaffected lymph node levels in HPV/p16-positive* ***and HPV/p16-negative*** *oropharyngeal carcinomas should be carried out with 45–54 Gy with single doses of 1.5–1.8 Gy.*

*The elective lymph node levels to be irradiated should be based on the current international consensus.*

- EC
- Strong consensus

**8.47 Consensus-based recommendation 2024**

*The adjuvant irradiation of HPV/p16-positive* ***and HPV/p16-negative*** *oropharyngeal carcinomas should be carried out using the IMRT technique with the best possible protection of the salivary glands, the swallowing tract and the oral cavity, without falling below the recommended doses in the target volumes.*

- EC
- Strong consensus

**8.48 Consensus-based recommendation 2024**

*If adjuvant radiochemotherapy is indicated for HPV/p16-positive or HPV/p16-negative oropharyngeal carcinoma, cisplatin-based chemotherapy should be administered simultaneously with radiotherapy.*

*In patients who cannot receive cisplatin, e.g. due to impaired renal function, mitomycin C + 5-FU, carboplatin + 5-FU or docetaxel can be used as simultaneous systemic therapy.*

- EC
- Strong consensus

#### 8.2.5 Neoadjuvant and adjuvant drug-based tumour therapy

**8.49 Evidence-based recommendation 2024**

*Neoadjuvant chemotherapy prior to planned definitive radio- or radiochemotherapy should not be carried out for* ***HPV/p16-negative*** *oropharyngeal carcinomas.*

- GoR: A
- LoE: 1++
- [1], [356], [357], [358]
- 1++: S3 guideline adaptation – Oral Cavity Carcinoma, Version 3.0 2021 (8.29)
- Strong consensus

**8.50 Consensus-based recommendation 2024**

*Neoadjuvant or adjuvant systemic therapy should only be used for primary surgical therapy or definitive radio- or radiochemotherapy for HPV/p16-positive* ***and HPV/p16-negative*** *oropharyngeal carcinomas within clinical trials.*

- EC
- Strong consensus

Neither induction concepts nor adjuvant, purely drug-based therapy concepts are generally recommended for the primary treatment of oropharyngeal carcinoma. In large meta-analyses, no advantages could be generated by adding these concepts. In the future, depending on the development of the promising study situation, it is conceivable that neoadjuvant and adjuvant concepts will find their way into the clinical practice of primary therapy through the use of checkpoint inhibitors [341], [342], [343]. 

### 8.3 Oropharyngeal carcinomas with distant metastases: UICC stages IV p16-positive:M1; stage IV-C p16-neg.

In the case of a tumour that has already metastasized at a distance at the time of initial diagnosis, the limited curative treatment options and the very limited prognosis must be strictly weighed in the interdisciplinary tumour board. The treatment options are subsumed under the generic term of palliative medical treatment (see also Chapter 9). As the principles in these tumour standards apply equally to squamous cell carcinoma of the oral cavity, oropharynx (p16-pos. and -neg.), hypopharynx and larynx, reference is made to the S3 Guideline on Oral Cavity Carcinoma, Version 3.0, Chapter 8.9 for further treatment recommendations, in which the topic and recommendations are described in detail and agreed upon [1].

### 8.4 Hypopharyngeal carcinomas in UICC stages I and II

#### General preliminary remarks

In the treatment of hypopharyngeal carcinoma, important surgical-anatomical features must be taken into account in addition to the purely descriptive anatomy (Chapter 3). These relate to the neighbouring relationships to the larynx, oesophageal opening, cervical vascular sheath and prevertebral fascia. As the hypopharyngeal mucosa is well vascularized or permeated by a dense network of lymphatic vessels and there is no natural barrier to the oesophagus, carcinomas can spread considerably faster than in the glottis, for example, which is naturally encircled by the laryngeal cartilage skeleton, regardless of the tumour biology. In addition, it is assumed that the hypopharynx and oesophagus have phylogenetic similarities and that there are therefore also similarities in oncology. This is related to the common embryonic precursor, the foregut [359]. In this context, a further important observation on the field carcinogenization of hypopharyngeal carcinomas should be described, which was observed after very good survival rates (60%; outside this study <10%) of T4b hypopharyngeal carcinomas with infiltration of the upper oesophagus after radical laryngohypopharyngoesophagectomy [360], i.e. resection of the hypopharynx with the entire oesophagus with consecutive gastric pull-up as oesophageal replacement and adjuvant radiotherapy with 60 Gy in conventional fractionation. In a comprehensive molecular characterization (SELDI-TOF proteomics), a group of patients with hypopharyngeal carcinoma was compared with a group of non-cancerous patients. In both groups, several samples were examined in the hypopharynx and oesophagus. The analysis revealed a group of 45 aberrantly expressed proteins that were only detectable in the cancer patients. In some of the cancer patients, comparable expression patterns of this signature were also found in the supposedly healthy oesophageal mucosa along the oesophagus. The 5-year survival rates of these patients showed that the signature proved to be predictive of the presence of field cancerization in the oesophagus. All patients with hypopharyngeal carcinoma and the named signature in the oesophagus had died and 80% of patients without this signature in the oesophagus survived [361], [362].

Thus, squamous cell carcinomas of the hypopharynx behave in a similar aggressive manner to those of the oesophagus. There is a close phylogenetic link between the hypopharynx and oesophagus, which appears to be strictly distinct from the oropharynx and larynx. In particular, the observations on field carcinogenesis in hypopharyngeal carcinoma described above underline the importance of oesophagoscopy in the context of diagnostic panendoscopy (see also Chapter 7) and raise awareness of the therapeutic consideration of hypopharyngeal carcinoma as a separate entity. In addition, locoregional metastases occur very early in hypopharyngeal carcinoma and the stage distribution at initial diagnosis tends to be predominantly advanced compared to other head and neck entities (>80% stages III, IV at initial diagnosis [363]). The comparatively poor prognosis decreases further with increasing patient age [364]. In a more recent registry study (SEER 1,780 patients), the following were shown to have an unfavourable prognosis: older patients with a higher T category, advanced N category, hypopharyngeal posterior wall involvement, multiple distant metastases and no reasonably possible surgical treatment with the aim of R0 resection (inoperability) of the primary tumour [363].

Also noteworthy is the reference to a subgroup of 21% of patients with HPV/p16-positive hypopharyngeal carcinomas in a registry study (NCDB, 2004–2016; 9,314 patients), who showed significantly better therapy-independent overall survival compared to non-HPV-associated patients (HR to death was 0.60, p≤0.0001) [365] HPV/p16 status has not yet been taken into account in the treatment of hypopharyngeal carcinomas due to a lack of further data.

According to William Wei, three growth types of hypopharyngeal carcinoma are described:

1. “clear margins” (approximately 70% of cases
2. “submucosal spread” (approximately 30% of cases)
3. “submucosal spread and skip lesions” (submucosal spread and drip metastases below the tumour in the upper oesophagus, rather rare)

For this reason, some authors demand different resection margin safety distances for hypopharyngeal carcinoma, which far exceed the obligatory 5 mm for other head and neck localizations. Wei, for example, requires 2 cm tumour-free resection margins upwards, 3 cm downwards and 2 cm laterally for hypopharyngeal carcinoma (overview [5]). However, this view has not been accepted internationally, so that the 5 mm safety margin from the tumour front is also regarded as a “clean” resection margin for hypopharyngeal carcinoma (Section 6.6).

Primary surgical treatment ± adjuvant radio- or radiochemotherapy and primary radio- or radiochemotherapy have been established as primary treatments for localized hypopharyngeal carcinomas. Direct randomized comparisons of the two principal treatment methods have never been carried out. The available registry data show a worse prognosis than for oropharyngeal carcinoma in all locoregionally limited tumour stages, regardless of the treatment method. For T1N0-T2N0 squamous cell carcinomas of the hypopharynx, there are no relevant differences between primary surgical and primary non-surgical treatment in terms of overall survival and the locoregional recurrence rate [11], [365].

#### 8.4.1 Surgical therapy

**8.51 Evidence-based statement 2024**

*There is no evidence that transoral treatment procedures (TLM, TORS) are inferior to open transcervical surgical procedures for T1 and T2 hypopharyngeal carcinoma in terms of tumour recurrence rate and survival.*

- LoE: 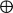

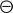

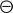

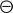
- [11], [241], [365]
- Strong consensus

**8.52 Evidence-based recommendation 2024**

*Hypopharyngeal carcinomas in stages cT1-cT2 cN0 cM0 should be treated either with primary resection ± adjuvant radio- or radiochemotherapy or with primary radio- or radiochemotherapy.*

- GoR: A
- LoE: 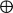

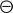

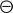

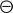
- [365]
- Strong consensus

In contrast to laryngeal carcinoma, organ-preserving surgery is only rarely considered for carcinomas of the hypopharynx due to the advanced stage of the tumour at the time of diagnosis [366], [367]. In addition, patients with hypopharyngeal carcinomas are often in an unfavourable general clinical condition due to extensive comorbidities [368]. Frequently, there is a long-standing, severe nicotine and alcohol abuse, chronic bronchitis, COPD, malnutrition, liver dysfunction and a consecutive impairment of blood coagulation. The prognosis of hypopharyngeal carcinoma across all stages is the least favourable of all squamous cell carcinomas of the head and neck in Europe (5-year survival <20%, [369]). A careful preoperative risk assessment is therefore necessary before therapeutic considerations, especially primary surgical measures, and intensive postoperative monitoring is therefore often planned.

Advances in open laryngeal surgery and TLM have now enabled oncologically safe R0 tumour resections with acceptable functional restrictions in some patients with hypopharyngeal carcinomas. One of the basic principles of oncological surgery is that a complete, histologically verifiable R0 resection (>5 mm) must be guaranteed. If this is not reliably possible as part of an intended surgical procedure (such as a TLM or TORS), alternative treatment options (open extended partial resection and reconstruction, laryngectomy or non-surgical treatment options) must be chosen as a matter of priority. In the case of hypopharyngeal carcinomas, whether a complete tumour resection will be possible can only be assessed by reviewing the endoscopic findings (as part of diagnostic panendoscopy by an experienced tumour surgeon) and meaningful imaging (usually MRI, CT) and preoperative swallowing diagnostics by FEES (see also Chapter 7). In addition to TLM [367], [370], [371], [372], [373], [374], [375], [376], [377], [378], TORS [240], [241] has also found its way into the treatment of hypopharyngeal carcinoma worldwide.

Patients with smaller tumours of the medial wall of the piriform sinus and the postcricoid region, which are still movable on the cartilaginous base, as well as smaller and superficially growing tumours of the lateral wall of the piriform sinus and the posterior wall of the hypopharynx are particularly suitable for TLM. For tumours in the area of the apex of the piriform sinus and the oesophageal orifice, organ-preserving surgery is generally not feasible. In 228 consecutive patients with hypopharyngeal carcinomas treated at a TLM clinic with experienced surgeons, surgical treatment was only possible in 136 patients (60% of all patients). Of these 136 patients, 90 underwent total laryngectomy. Of the 46 patients who underwent organ-preserving surgery, 23 underwent open partial pharyngectomy and 23 underwent transoral laser surgery. This means that 34% of all patients operated on and 20% of all 228 patients in the entire series underwent organ-preserving surgery, half of which, i.e. only 10% of all patients, underwent transoral laser surgery [367]. Even though these data are older, the authors believe that the surgical approach to TLM in hypopharyngeal carcinoma has not changed to date.

Overall, it can therefore be assumed that only around 20% of all patients with hypopharyngeal carcinoma can undergo larynx-preserving surgery. Of these, around half can be operated on by laser surgery and the other half by open partial pharyngectomy.

If the tumour reaches the resection margins, a subsequent resection should be attempted in order to achieve an R0 result; as already explained in Section 6.6, a subsequent resection has no negative prognostic influence, particularly in the TLM of laryngeal/hypopharyngeal carcinoma [134].

There is extensive literature on the high validity of TLM. The indications for organ-preserving TLM in the hypopharynx range from T1 to T4a tumours with good results: Steiner et al. published local control rates over five years of 84% pT1; 70% pT2; 75% pT3; 57% pT4a; recurrence-free 5-year survival 73% for stages I and II, 59% for stage III, 47% for stage IVa [377]. Eckel et al. showed an (uncorrected) survival of 59.3% and a local control rate of 73.3% in large collectives after 5 years, with 76.5% of all patients showing a functioning larynx after this time [367]. Rudert et al. showed an uncorrected survival of 48% and a disease-specific survival of 58% (71% in stages I and II) [379]. In carefully selected patients with early hypopharyngeal carcinoma, transoral laser surgery (in combination with unilateral or bilateral neck dissection and, if necessary, postoperative radiotherapy) leads to very good oncological and functional results.

As part of the PICO 4 analysis (Evidence Table PICO 4), we became aware of the only comparative study worldwide to date on TORS versus open surgery, in which only hypopharyngeal carcinomas were treated [241]. In n=56 patients, the tumour stages were distributed as follows: T1–T2 80%, T3 16%, T4 14% (male proportion 98%, mean age 64.7 years). The evidence is very low, as this is a retrospective study without matching and increased T1 tumours in the intervention group (TORS) and thus there is an imbalance between the intervention and control groups. The 2-year DSF showed no difference between the groups (relative risk: 1.08; CI 95% 0.83–1.41), with lower intraoperative blood loss and shorter hospital stay in the TORS group.

Transoral resection (preferably TLM) of hypopharyngeal carcinomas should be avoided and the legitimacy of converting the operation to laryngectomy should always be discussed and obtained. It is therefore advisable to plan and determine the therapeutic procedure in detail for each individual patient in advance as part of the interdisciplinary tumour board, whereby the individual situation and special wishes of the patient concerned must of course be taken into account. Subsequent radiotherapy cannot be considered an appropriate completion of an incomplete tumour resection.

With regard to functional impairment, the extent of the expected postoperative swallowing impairment in particular must be assessed in advance. In particular, the following questions must be clarified:

- in view of the planned extent of resection, whether temporary or prolonged aspiration must be expected,
- whether the patient’s overall clinical situation suggests that temporary aspiration can be tolerated, and
- whether a temporary tracheostomy or PEG may be required.

On the other hand, more serious vocal impairments are generally not to be expected with transoral resection of hypopharyngeal carcinomas, and serious airway obstructions are also generally not to be expected with atraumatic surgical technique and perioperative shielding with antibiotics and steroids.

Some unfavourably located T2 hypopharyngeal carcinomas are reserved for the classic transcervical, i.e. open, techniques. Please refer to Section 8.5.1.

#### 8.4.2 Radiotherapy

The principles of radio- and radiochemotherapy for hypopharyngeal carcinoma do not differ from those for HPV/p16-negative oropharyngeal carcinoma with regard to technical implementation, contouring of target volumes and organs at risk (see 8.1.2).

#### 8.4.3 Primary radiotherapy ± combination with drug-based tumour therapy

**8.53 Consensus-based recommendation 2024**

I*n the case of primary non-surgical therapy, patients with hypopharyngeal carcinomas in stages I–II (UICC 8^th^ edition) should receive radiotherapy alone.*

*Radiochemotherapy may be offered for larger T2 tumours.*

- EC
- Strong consensus

**8.54 Consensus-based recommendation 2024**

*If radiochemotherapy is indicated for stage II hypopharyngeal carcinoma, cisplatin-based chemotherapy should be administered simultaneously with radiotherapy.*

- EC
- Strong consensus

**8.55 Consensus-based statement 2024**

*In patients who cannot receive cisplatin, e.g. due to impaired renal function, mitomycin C + 5-FU, carboplatin + 5-FU or a taxane can be used as simultaneous systemic therapy.*

- EC
- Strong consensus

**8.56 Consensus-based recommendation 2024**

*For primary radiotherapy or radiochemotherapy of hypopharyngeal carcinoma in stages I–II (UICC 8* *^th^* *edition), radiotherapy should be carried out with 5x2 Gy per week up to a target volume dose of 70 Gy in the area of the affected lymph nodes and the primary tumour or another established regimen with a biologically equivalent total dose should be used.*

*Irradiation of unaffected lymph node levels should be carried out with 45–54 Gy with individual doses of 1.5–1.8 Gy.*

*The elective lymph node levels to be irradiated should be based on the current international consensus.*

- EC
- Strong consensus

**8.57 Consensus-based recommendation 2024**

*Primary radio- or radiochemotherapy of hypopharyngeal carcinomas in stages I+II should be carried out using the IMRT technique with the best possible protection of the salivary glands, the unaffected parts of the swallowing tract and the oral cavity, without falling below the recommended doses in the target volumes.*

- EC
- Strong consensus

The registry data from the USA [365], Canada [380] and the Netherlands [11] unanimously show that the treatment results in terms of overall and progression-free survival for squamous cell carcinoma of the hypopharynx in stages T1-2 N0 do not differ significantly depending on whether primary surgical treatment, primary radiotherapy or primary radiochemotherapy was carried out. Accordingly, the current recommendations of the ESMO and NCCN guidelines also list all of these treatment options without preference [381]. Particularly in the case of larger T2 tumours (e.g. postcricoid), where a primary laryngectomy would be necessary surgically, organ preservation by means of primary radiochemotherapy or induction chemotherapy followed by radiotherapy in the event of a good response to induction therapy or laryngectomy in the event of a poor response should be discussed on an interdisciplinary basis, as described in more detail under 8.5.2.

The same principles apply to the dosage and fractionation of radiotherapy as for HPV/p16-positive oropharyngeal carcinomas (see 8.1.3), i.e. radiotherapy was carried out in most studies on radio- or radiochemotherapy with 5x2 Gy per week up to target volume doses of 70 Gy in the area of the primary tumour and the affected lymph nodes. Slightly accelerated and also hyperfractionated radiation regimens with biologically equivalent doses give approximately equivalent results (described in detail in 8.1.3).

An advantage in terms of relapse-free survival or overall survival with simultaneous radiochemotherapy compared to radiochemotherapy alone has not been established for squamous cell carcinoma of the pharynx, larynx and oral cavity in stages I+II. In the patient population of the MACH-NC meta-analysis, only 5.5% of patients were in stages T1N0 and T2N0 and only 15.5% of patients had hypopharyngeal carcinoma [308]. In an earlier evaluation of the MACH-NC meta-analysis, stages I–II were evaluated separately for all tumour locations. There was no benefit of additional chemotherapy compared to radiotherapy alone for these early stages [346]. For locally advanced squamous cell carcinomas, however, a survival benefit from chemotherapy applied simultaneously with radiotherapy is well documented [308], [346]. Whether patients with larger T2N0 hypopharyngeal carcinomas benefit from radiochemotherapy cannot be ruled out. If radiochemotherapy is indicated for these cases after interdisciplinary discussion, the same criteria apply with regard to the selection of chemotherapy as for locally advanced hypopharyngeal carcinomas (see 8.5.3).

#### 8.4.4 Adjuvant radiotherapy ± combination with drug-based tumour therapy

**8.58 Consensus-based recommendation 2024**

*Patients with stage I+II hypopharyngeal carcinoma (UICC 8* *^th^* *edition) who have undergone primary surgical treatment should receive adjuvant radiotherapy or radiochemotherapy if resection R1 or <5 mm has been performed in healthy tissue. Adjuvant radiotherapy can also be offered for G3, L1, V1 or Pn1.*

- EC
- Strong consensus

**8.59 Consensus-based recommendation 2024**

*If adjuvant radiotherapy is indicated for hypopharyngeal carcinoma in stages I+II, radiotherapy should be given with 60–66 Gy (1.8–2.2 Gy single dose) in the area of the narrow resection margins and with 45–54 Gy with single doses of 1.5–1.8 Gy in the area of unaffected lymph node levels.*

- EC
- Strong consensus

**8.60 Consensus-based recommendation 2024**

*Postoperative radiotherapy or radiochemotherapy of hypopharyngeal carcinomas in stages I–II should be started as soon as possible after wound healing has been completed within a period of 6 weeks after surgery and should be carried out using the IMRT technique with the best possible protection of the salivary glands, the swallowing tract and the oral cavity, without falling below the recommended doses in the target volumes.*

- EC
- Strong consensus

Results of randomized studies on the value of radio- or radiochemotherapy compared to no adjuvant therapy are not available for squamous cell carcinomas of the hypopharynx or for oropharyngeal and laryngeal carcinomas or tumours of the oral cavity. This applies both to stages T1-2N0 and to locally advanced tumours. However, the results of systematic reviews and registry studies [319], [320], [321] indicate an improvement in relapse-free and overall survival if risk factors are present, although the subgroups of hypopharyngeal carcinomas in stages T1-2N0 were very small. In the registry studies, the narrow resection margin (<5 mm, N+ and ECE) was identified as a risk factor, whereas other factors such as G3, L1 and V1 were not investigated. For T1-2N0 carcinomas resected ≤5 mm in healthy tissue, the available data suggest that postoperative radio- or radiochemotherapy is likely to be beneficial. For patients with other risk factors (G3, L1, V1), a benefit can neither be excluded nor confirmed.

For locally advanced squamous cell carcinoma of the pharynx, larynx and oral cavity, several randomized studies have shown an improvement in relapse-free survival [326], [327], [328]; in a meta-analysis [382] improved overall survival for patients in whom resection ≤5 mm was performed in healthy tissue was also shown. However, no patients with T1-T2 N0 tumours were treated in these studies, so that the benefit of additional simultaneous chemotherapy to postoperative radiotherapy is unknown.

#### 8.4.5 Neoadjuvant and adjuvant drug-based tumour therapy

Neoadjuvant treatment concepts are not used for T1N0 and T2N0 hypopharyngeal carcinomas. One exception is “large” T2 carcinomas, which can no longer be meaningfully resected with laryngectomy and can therefore be treated with a laryngeal organ preservation protocol as an alternative to primary laryngopharyngectomy (Section 8.5.3). 

### 8.5 Hypopharyngeal carcinomas in UICC stages III and IV

**8.61 Evidence-based statement 2024**

*The treatment outcomes for squamous cell carcinoma of the hypopharynx in stages cT3 cN0–cN3 cM0 after primary resection ± adjuvant radiotherapy or radiochemotherapy do not differ significantly in terms of survival in the available registry data compared to primary radiotherapy or radiochemotherapy.*

- LoE: 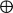

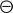

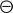

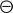
- [383], [384]
- Strong consensus

**8.62 Evidence-based recommendation 2024**

*Hypopharyngeal carcinomas in stages cT3 cN0–cN3 cN0 should be treated either with primary resection ± adjuvant radio- or radiochemotherapy or with primary radio- or radiochemotherapy.*

- GoR: B
- LoE: 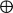

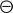

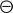

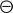
- [383], [384]
- Strong consensus

**8.63 Evidence-based recommendation 2024**

*If a laryngectomy is required surgically, neoadjuvant chemotherapy followed by radio- or radiochemotherapy can be carried out in addition to the aforementioned treatment methods if there is a good response to neoadjuvant therapy (at least partial regression) or subsequent* *resection if there is a poor response to neoadjuvant therapy.*

- GoR: B
- LoE: 1a
- [2], [346], [347], [352], [353]
- 1a: S3 guideline adaptation – laryngeal carcinoma, version 1.1 2019 (7.28)
- Strong consensus

**8.64 Consensus-based statement 2024**

*In patients with cT4a cN0-cN3 cM0 hypopharyngeal carcinoma, there is evidence from cancer registry databases for better overall survival after a primary surgical approach.*

- EC
- Strong consensus

**8.65 Consensus-based recommendation 2024**

*Patients with hypopharyngeal carcinomas of stage cT4a cN0–cN3, in whom an R0 resection appears surgically possible, should undergo primary surgical treatment.*

*Alternatively, radiochemotherapy or neoadjuvant chemo* *therapy can be carried out, accepting a higher local recurrence rate.*

- EC
- Strong consensus

**8.66 Evidence-based recommendation 2024**

*If an R0 resection of a hypopharyngeal carcinoma in stage cT4a cN0–cN3 cM0 is probably not achievable, primary radiochemotherapy should be given.*

- GoR: A
- LoE: 1a
- [2], [346], [347]
- 1a: S3 guideline adaptation – Laryngeal Carcinoma, Version 1.1 2019 (7.34)
- Strong consensus

Primary surgical therapy ± adjuvant radio- or radiochemotherapy and primary radio- or radiochemotherapy have been established as primary therapies for localized hypopharyngeal carcinomas. Direct randomized comparisons of the two principal treatment methods have never been carried out. The available registry data show a worse prognosis in all locoregionally limited tumour stages than in oropharyngeal carcinomas, regardless of the treatment method. For T1N0-T2N0 squamous cell carcinomas of the hypopharynx, there are no relevant differences between primary surgical and primary non-surgical treatment in terms of overall survival and the locoregional recurrence rate [11], [365]. For T4 (N0/N+) tumours; however, the registry data show a significant survival advantage for primary surgical treatment followed by adjuvant radio- or radiochemotherapy compared to primary radiochemotherapy. For hypopharyngeal carcinoma in stage T4a, the international guidelines (EHNS-ESMO-ESTRO ) and this guideline therefore give preference to primary surgical treatment followed by adjuvant radiotherapy or radiochemotherapy [381].

Cartilage infiltration of the cartilago cricoidea and thyroidea was identified as a key criterion for the greater effectiveness of primary surgical treatment methods, in addition to the advanced extent of the tumour. The complex anatomy and special expertise in the interpretation of imaging of hypopharyngeal carcinoma was pointed out [385], [386], [387]. Alternatively, non-surgical modalities for laryngeal organ preservation and the downstream option of salvage surgery can be considered as a second choice in this situation, particularly if primary laryngectomy is declined [388]. Primary ablative surgery (laryngopharyngectomy) and non-surgical treatment procedures are only considered equivalent options for T3N0-3 hypopharyngeal carcinoma (i.e. without cartilage infiltration), as is the case for the larynx. In T4b situations, the therapeutic options are generally limited to non-surgical treatment methods. Overall, the poor prognosis in advanced stages, the high level of existing comorbidities and the usually late initial diagnosis in already advanced stages must be taken into account when choosing the appropriate treatment for hypopharyngeal carcinoma patients.

#### 8.5.1 Surgical therapy

**8.67 Consensus-based statement 2024**

*A tracheostomy performed before a laryngectomy has a negative effect on the prognosis because stoma recurrences can occur more frequently.*

- EC
- Strong consensus

**8.68 Consensus-based recommendation 2024**

*A tracheotomy should be avoided before a planned total laryngopharyngectomy. In the event of dyspnoea, transoral tumour debulking can be performed as part of the initial diagnosis to avoid a tracheotomy.*

- EC
- Strong consensus

In principle, some T3 hypopharyngeal carcinomas can also be safely resected using transoral procedures, preferably TLM. Please refer to Section 8.4.1. The vast majority of stage III and IV hypopharyngeal carcinomas will be reserved for open transcervical techniques in the event of a surgical procedure:

##### Transcervical laryngeal and partial hypopharyngeal resection

These resections are exceptional situations with strict indications because they can result in a lengthy rehabilitation phase of months to years for the patients concerned. In this case, a hemilaryngectomy is combined with a partial resection of the hypopharynx (medial and/or lateral piriform sinus wall) for laryngeal tumours growing transglottically on one side. Depending on the extent of the resection, the laryngeal skeleton can be built up with a rib cartilage graft and the hypopharynx reconstructed with a forearm graft. The creation of a wide piriform sinus with a thin forearm graft maintains the flexibility of the reconstructed larynx and thus enables elevation during the act of swallowing. This forward and upward movement of the larynx with simultaneous opening of the oesophageal inlet is the prerequisite for aspiration-free food intake ([5], [389], [390], [391], [392], [393]; S3-Larynx chap. 7.5 [2]). The laryngotracheal flap (LTF) as a local tissue transfer should also be mentioned here [394]. By using a laryngotracheal approach for hypopharyngeal tumour excision, the contralateral LTF can be used to reconstruct the hypopharyngeal defect. Although the contralateral uninvolved laryngotracheal tissue remains intact, this does not appear to increase the tumour recurrence rate. This may also reduce the use of complicated regional or free flaps. According to Chu et al. from Teipei, 75% of hypopharyngeal defects could be reconstructed with this flap without other flaps. Postoperative complications are rare, with only 2% of patients having a pharyngoesophageal stenosis and 5% a pharyngocutaneous fistula [395].

##### Pharyngolaryngectomy

As primary therapy, pharyngolaryngectomy is still an important part of the therapeutic options for advanced-stage carcinomas. In principle, direct closure of the pharynx is attempted if there is still sufficient residual tissue in the hypopahrynx. The use of a voice prosthesis is also recommended for voice rehabilitation using the primary technique (detailed description in S3-LL Laryngeal carcinoma [2]). The ideal method for hypopharyngeal reconstruction should preferably have the following characteristics: single-stage procedure, high success rate of tissue transfer, low removal morbidity, low fistula and stenosis rates, restoration of the ability to speak and swallow, successful reconstruction achievable in a heavily irradiated area and tolerance of postoperative radiotherapy (overview [395]).

Complete, i.e. circular hypopharyngeal resection generally requires reconstruction of the alimentary canal. The forearm graft is the method of choice for this. It is important to plan the graft sufficiently wide in order to be able to model the widest possible “tube”. To prevent postoperative stenosis, the lower anastomosis with the oesophageal inlet should be dissolved by triangular exchange [390].

Another variant of stenosis prophylaxis with simultaneous formation of a very wide neohypopharynx was described by Bootz et al. [396]. In this case, the forearm graft is sutured to the prevertebral fascia with the longitudinal edges in a U-shape. This reduces shrinkage and stenosis to a minimum. Alternatively, the reconstruction can also be performed with a pedicled flap. This is suitable for poor vascularization after extensive resection and in the context of salvage resections after primary radiochemotherapy. Voice rehabilitation is performed with voice prostheses that are placed in the anterior esophageal wall below the caudal anastomosis.

Alternatively, jejunum transfer is recommended as a replacement after circular laryngopharyngectomy. Due to the segmental blood supply of the jejunum, up to 20 cm of jejunum can be harvested on the basis of a single vascular arcade. The transfer of the vascular mesentery with the jejunum is a further advantage of this flap, as it allows all dead spaces to be filled and all important vascular structures to be covered. In patients with limited oesophageal dilatation (“small” T4b), free jejunal flaps have an overall success rate of 90–100%. In addition, fistula and stricture rates are acceptably low [397], [398]. Clinical experience with the jejunal flap has shown a high rate of successful recovery of swallowing function, with earlier rehabilitation and restoration of swallowing compared to other reconstructive methods. However, some patients may suffer from intermittent dysphagia due to uncoordinated peristalsis during swallowing. Voice rehabilitation is also a major problem with this visceral transfer [399]. A tracheoesophageal puncture with voice prosthesis insertion results on average in a less satisfactory voice than is achieved with skin flaps. Excessive mucus production tends to clog the prosthesis and results in a typically wet voice that lacks volume [399]. The free jejunal flap also requires a laparotomy to harvest the graft. The need for intra-abdominal surgery exposes the patient to additional abdominal morbidity and even mortality [399], [400]. Adhesions, intestinal bleeding, intestinal obstruction and anastomotic or abdominal wound dehiscence are among the possible complications that have led to a reluctance to perform jejunum transfer in recent years.

A very rarely used alternative for voice rehabilitation is the construction of a speech siphon with simultaneous reconstruction of the alimentary canal from overlong jejunal segments and was also described by Remmert et al. [401], [402]. The procedure is primarily suitable for younger patients due to the need for abdominal surgery and has the advantage of a very short rehabilitation period of a few weeks to regain swallowing and vocal function ([2], Chapter 7.5).

##### Pre-therapeutic tracheostomy 

When a hypopharyngeal carcinoma with relevant laryngeal involvement is first diagnosed, there may already be considerable dyspnoea, which often necessitates a pretherapeutic tracheostomy (analogy Ch. 7.1 [2]. Patients with extensive laryngeal carcinomas are often admitted to hospital for emergency treatment. A primary tracheostomy is often performed as an emergency measure. It is known from retrospective observations that tracheotomy, which is necessary before a laryngectomy or partial laryngectomy, can have a negative effect on the prognosis [403], [404], [405], [406]. In particular, recurrences in the tracheostoma area are described more frequently in such cases, which are difficult to treat with both surgery and radiation. If a recurrence occurs in the area of the tracheostoma, the prognosis with regard to survival deteriorates significantly [407], [408]. If a laryngectomy is considered as the primary treatment option, the procedure should be performed promptly in the event of dyspnoea in order to prevent a pre-therapeutic tracheostomy, or the patient should remain intubated until the laryngectomy (exceptional cases). Furthermore, attempts can be made to avoid tracheotomy, e.g. by endoscopic tumour debulking during the first examination under anaesthesia. If the decision is made to undergo primary radiotherapy, a pre-therapeutic tracheostomy is unavoidable, whereby this should be included in the radiation field to prevent recurrence in the tracheostoma area [409].

##### Pharyngolaryngectomy + oesophageal resection + gastric pull-up

The very radical and extensive surgery for T4b hypopharyngeal carcinomas with oesophageal infiltration mentioned at the beginning is only possible and sensible in very few cases. Surgery-related mortality is particularly high in this patient group due to the high level of comorbidities. The literature reports a mortality rate of between 5% and 25% together with an overall incidence of complications of between 26% and 55% [410], [411], [412], [413]. In this respect, gastric pull-up should therefore be reserved for exceptional situations (alternative: colonic interposition). In any case, a surgical partner team that is highly experienced in oesophageal surgery is essential for the success of the operation [360].

#### 8.5.2 Radiotherapy

The basic considerations of the current scientific classification of hypopharyngeal carcinoma have already been outlined (Section 8.1.2). The same principles apply to the dosage and fractionation of radiotherapy for locally advanced hypopharyngeal carcinomas as for HPV/p16-negative oropharyngeal carcinomas (see 8.1.3), i.e. radiotherapy was carried out in most studies on radio- or radiochemotherapy with 5x2 Gy per week up to target volume doses of 70 Gy in the area of the primary tumour and the affected lymph nodes. Slightly accelerated and also hyperfractionated radiation regimens with biologically equivalent doses produce approximately equivalent results (described in detail in Section 8.1.3).

#### 8.5.3 Primary radiotherapy ± combination with drug-based tumour therapy

**8.69 Consensus-based recommendation 2024**

*The primary non-surgical therapy for patients with* *stage III–IVb* *hypopharyngeal carcinoma (UICC 8* *^th^* *edition) should be radiochemotherapy.*

- EC
- Strong consensus

**8.70 Evidence-based statement 2024**

*In patients with stage III–IVb hypopharyngeal carcinoma (UICC 8* *^th^* *edition), locoregional tumour control and overall survival are statistically significantly better after primary radiochemotherapy than after radiotherapy alone.*

- LoE: 1a
- [2], [308], [346], [347], [352], [353]
- 1a: S3 guideline adaptation – Laryngeal Carcinoma, Version 1.1 2019 (7.9)

**8.71 Evidence-based recommendation 2024**

*In stage III–IVb hypopharyngeal carcinoma, simultaneous chemotherapy should be cisplatin-based in the case of primary radiochemotherapy.*

- GoR: A
- LoE: 1a
- [2], [308], [346], [347]
- 1a: S3 guideline adaptation – Laryngeal Carcinoma, Version 1.1 2019 (7.14)
- Strong consensus

**8.72 Consensus-based recommendation 2024**

*In patients who cannot receive cisplatin, for example due to impaired renal function, carboplatin + 5-FU, mitomycin C + 5-FU or a taxane can be used as simultaneous systemic therapy.*

- EC
- Consensus

**8.73 Consensus-based recommendation 2024**

*For primary radiotherapy or radiochemotherapy of hypopharyngeal carcinoma in stages III–IVb (UICC 8* *^th^* *edition), radiotherapy should be carried out with 5x2 Gy per week up to a target volume dose of 70 Gy in the area of the affected lymph nodes and the primary tumour or another established regimen with a biologically equivalent total dose.*

- EC
- Strong consensus

**8.74 Consensus-based recommendation 2024**

*In primary radiotherapy or radiochemotherapy of hypopharyngeal carcinoma, unaffected lymph node levels should be irradiated with 45–54 Gy with single doses of 1.5–2 Gy.*

*The elective lymph node levels to be irradiated should be based on the current international consensus.*

- EC
- Strong consensus

**8.75 Consensus-based recommendation 2024**

*Primary radio- or radiochemotherapy of hypopharyngeal carcinomas in stages III to IVb should be carried out using the IMRT technique with the best possible protection of the salivary glands, the unaffected swallowing tract and the oral cavity, without falling below the recommended doses in the target volumes.*

- EC
- Strong consensus

##### Primary radiochemotherapy

For locally advanced squamous cell carcinoma of the pharynx, larynx and oral cavity, the MACH-NC meta-analysis [308], based on 34 randomized studies with 6,788 patients, showed an absolute survival benefit of 6.5% after 5 years for simultaneous radiochemotherapy compared to radiotherapy alone (p<0.001). The tumour location was known in 4,650 patients. 756 patients in this group had locally advanced hypopharyngeal carcinoma in stages III–IVb (M0). In this subgroup, the benefit of simultaneous radiochemotherapy in terms of survival compared to radiotherapy alone was slightly lower (hazard ratio 0.88, 95% confidence interval 0.75–1.04) than for oropharyngeal carcinoma (hazard ratio 0.81, 95% confidence interval 0.71–0.95) and not formally statistically significant. However, independent evaluations of registry data [11], [365], [380] show a significant survival advantage of 10–15% in favour of simultaneous radiochemotherapy for locally advanced hypopharyngeal carcinomas. The advantage was highest in patients under 60 years of age and hardly detectable in patients over 70 years of age. In contrast, there was no advantage for induction chemotherapy or adjuvant chemotherapy in the MACH-NC meta-analysis [308]. Irrespective of this, induction chemotherapy can be used if a primary laryngectomy would be necessary surgically in order to achieve laryngeal organ preservation depending on the response to induction chemotherapy, as described in more detail under 8.5.2.

The survival benefit for simultaneous radiochemotherapy compared to radiotherapy alone is best confirmed for the simultaneous application of cisplatin-containing chemotherapy and radiotherapy [308]. Most experience exists for 3x100 mg/m² cisplatin and weekly cisplatin (40 mg/m²) as well as for cisplatin in combination with 5-FU.

For patients in whom cisplatin is not an option, e.g. due to poor renal function, randomized studies in which patients with hypopharyngeal carcinoma were also treated demonstrated a survival advantage for the simultaneous application of carboplatin in combination with 5-FU [284], [285], [287], [288], mitomycin C in combination with 5-FU [289], [290] or weekly docetaxel [291] simultaneously with radiotherapy compared to radiotherapy alone. Only studies with ≤80 patients in the radiochemotherapy arm are available for monochemotherapy with carboplatin [286], [292], [293], [294] and there were no significant advantages for monotherapy with mitomycin C [295], [296], [297] in parallel with radiotherapy. Weekly applications of carboplatin and paclitaxel concurrently with radiotherapy have been investigated in a number of non-randomized phase II trials and some retrospective cohort studies [298], [299], [300], [301], [302], [303], [304]. Hypopharyngeal carcinomas represented only a small proportion of the treated squamous cell carcinomas of the head and neck. The non-randomized, retrospective comparisons indicate a similar efficacy of carboplatin + paclitaxel compared to weekly therapy with 40 mg/m² cisplatin in parallel with radiotherapy. In contrast, there is very little data available for the administration of paclitaxel alone [305]. A benefit of cetuximab in combination with radiotherapy for hypopharyngeal carcinoma cannot be derived from the available data [306], [307], [312].

In comparison to radiotherapy alone, simultaneous radiochemotherapy with cisplatin and other chemotherapies not only increases hematotoxicity, which is usually well manageable, but also the severity of acute mucositis with the associated swallowing difficulties. Other typical acute side effects of radiotherapy, such as skin reaction, xerostomia and loss of taste, are less severely increased by simultaneous chemotherapy, but the individual effects add up so that radiochemotherapy is significantly more stressful for patients overall [414]. An increase in the rate of late effects of radiotherapy due to additional chemotherapy is less well documented and has mostly been described as non-significant or borderline significant in individual studies [315], [415], [416], [417]. Due to the different ways in which late effects are documented, a valid meta-analysis of these data cannot be meaningfully carried out. Nevertheless, there is little doubt that the late side effects are also increased by the additional chemotherapy [414]. The working group led by J.A. Langendijk [418] was able to prove in prospective, systematic analyses [419], taking into account the individual dose distribution [420] of radiotherapy, that the rate of long-term swallowing disorders after combined radiochemotherapy is significantly higher than after radiotherapy alone [421], [422]. However, the same working group was also able to show that these negative effects can be at least partially reduced by using modern radiotherapy techniques such as swallowing sparing IMRT [268]. However, the effects described above are poorly documented for the subgroup of hypopharyngeal carcinomas, although swallowing disorders are to be expected particularly frequently due to the location of the tumours.

**8.76 Evidence-based recommendation 2024**

*If a laryngopharyngectomy is required surgically, neoadjuvant chemotherapy followed by radio- or radiochemotherapy can be carried out in addition to the aforementioned treatment methods if there is a good response to neoadjuvant therapy (at least partial regression) or subsequent resection if there is a poor response to neoadjuvant therapy.*

- GoR: A
- LoE: 1a
- [2], [346], [347], [352], [353]
- 1a: S3 guideline adaptation – Laryngeal Carcinoma, Version 1.1 2019 (7.28)
- Strong consensus

**8.77 Consensus-based recommendation 2024**

*All patients in the stages where a laryngectomy would be necessary should be discussed in the interdisciplinary tumour board and a joint therapy recommendation should be made by the ear, nose and throat specialist and the radiation oncologist. This treatment recommendation and the alternatives should be communicated to the patient by both disciplines.*

- EC
- Strong consensus

##### Laryngeal organ preservation

The topic of laryngeal organ preservation programs was initiated in the 1990s by the first large randomized studies in the USA and Europe with the introduction of definitive non-surgical protocols instead of laryngectomy, which in the event of failure resulted in salvage laryngectomy. For the history and the main treatment options established for advanced laryngeal carcinoma, please refer to the S3 Guideline on Laryngeal Carcinoma [2].

There are only a few randomized studies (Phase III: EORTC 1996 [233]; EORTC 2009 [423]), (Phase II: TREMPLIN [424]; DELOS II: [425]) for organ preservation of advanced hypopharyngeal carcinoma as an alternative to pharyngolaryngectomy. The first large randomized study and the only one that directly compared a non-surgical with a surgical treatment procedure was initiated by the EORTC and recruited 202 patients with untreated, operable hypopharyngeal (78%) and laryngeal (22%) carcinomas [233]. Patients were randomized into an experimental arm with up to three cycles of induction chemotherapy with cisplatin/5-FU followed by standard fractionated radiotherapy up to a total dose of 70 Gy and a standard arm with partial pharyngolaryngectomy and postoperative radiotherapy. Patients in the experimental arm who developed a partial remission after the first cycle of induction chemotherapy received a second and third cycle. Patients who developed a complete remission at any point during induction chemotherapy were randomized to radiotherapy. Patients who developed less than a partial remission after the first cycle and less than a complete remission after the third cycle were assigned to pharyngolaryngectomy without prior radiotherapy. After induction chemotherapy, 54% of patients developed a complete remission in the area of the primary tumour and 43% in the area of the primary tumour and the locoregional lymphatic drainage pathways. After five years, there was no significant difference in survival between the two study arms (30% versus 35% in the surgical arm). The 3- and 5-year survival rates with functional larynx were 42% and 35%, respectively. In patients who achieved complete remission after induction chemotherapy and subsequently received only radiotherapy, a functional larynx was still present in 58% of patients after 5 years. It should be noted that only 6% of the included patients had a T4 tumour (T3: 75%, T2: 19%), so that no valid statement can be made from this study as to whether the induction concept can also be offered to patients with T4 tumours without a survival disadvantage compared to primary surgical therapy ± radiotherapy or radiochemotherapy.

The risk of distant metastasis was significantly lower in the induction chemotherapy arm (25% versus 36%). In the second EORTC study mentioned, an alternating chemotherapy protocol was compared with a sequential chemotherapy protocol with a standard arm of the protocol described above. In 450 patients, there was no difference between the two arms [423]. A study directly comparing primary simultaneous radiochemotherapy and primary laryngectomy has not yet been conducted.

If the aim is to achieve functional laryngeal preservation using induction chemotherapy, a taxane-containing chemotherapy protocol has proven to be advantageous (taxane, platinum, 5FU: TPF) [426], [427]. With high response rates and good selection options, only a few salvage operations are usually necessary. The additional application of docetaxel during induction therapy also increased the laryngeal preservation rate 3 years after therapy from 57.5% to 70.3% (p=0.03) in a randomized study [426].

This effect was also confirmed in the DeLOS II study, albeit without the additive value of cetuximab for induction of organ preservation and overall survival. Early laryngectomy prior to radiotherapy was performed in 30% of patients after lack of response to short induction (1 cycle TPF/TP and subsequent endoscopic response assessment), which led to comparatively low toxicities/complications with very good 2-year survival [425]. In the DeLOS-II study, 50% of 170 patients had hypopharyngeal carcinomas with 69% stage IV tumours. The 2-year survival was 70% with 49% laryngectomy free survival.

Furthermore, induction therapies are being modified in the direction of less toxic substances, and 5-FU is being partially replaced. The randomized phase II TREMPLIN trial showed that cetuximab can achieve equally good results as simultaneous administration of cisplatin with a different toxicity profile (greater cutaneous toxicity, less haematotoxicity and nephrotoxicity), which may be advantageous for some patients [424].

Critics of organ preservation programmes cite the high function-limiting late toxicity, the higher non-tumour-related mortality and the comparatively high complication rates if salvage surgery is necessary. The supporters, on the other hand, cite the high rate of organ preservation with adequate patient selection, the possibility of salvage surgery as a curative option and the lack of a survival disadvantage. Careful individual counselling of the patient is therefore of particular importance in this situation. In the S3 guideline on laryngeal cancer, the option of primary non-surgical laryngeal organ preservation was included as an alternative to laryngectomy and should not be withheld from patients as a realistic treatment option in the consultation. This recommendation is adopted for hypopharyngeal carcinoma in the same way as for laryngeal carcinoma.

#### 8.5.4 Adjuvant radiotherapy ± combination with drug-based tumour therapy

**8.78 Consensus-based recommendation 2024**

*Postoperative radio- or radiochemotherapy should be used for hypopharyngeal carcinomas*

*– for pT3 carcinomas and pT4 carcinomas*

*– pN2-pN3*

*– for carcinomas with narrow or positive resection margins (R0<5 mm; R1), perineural invasion, vascular invasion (lymph vessel invasion and/or venous invasion)*

*– in the case of an affected lymph node with extracapsular tumour growth.*

- EC
- Strong consensus

**8.79 Consensus-based recommendation 2024**

*Postoperative radiochemotherapy should*

*– for R1 or resection margin <5 mm in the area of the mucosa in the parts of the tumour not surrounded by cartilage or*

*– extracapsular tumour growth at the lymph nodes.*

- EC
- Strong consensus

**8.80 Evidence-based recommendation 2024**

*If adjuvant radiochemotherapy is indicated, chemotherapy should be carried out with a simultaneous cisplatin-containing regimen.*

- GoR: A
- LoE: 1b
- [2], [327], [355], [428]
- 1b: S3 guideline adoption – Laryngeal Carcinoma, Version 1.1 2019 (7.39)
- Strong consensus

**8.81 Consensus-based recommendation 2024**

*In patients who cannot receive cisplatin, e.g. due to impaired renal function, mitomycin C + 5-FU, carboplatin + 5-FU or docetaxel can be used as simultaneous systemic therapy.*

- EC
- Strong consensus

**8.82 Consensus-based recommendation 2024**

*For stage III–IVb hypopharyngeal carcinomas, adjuvant irradiation should be given for*

*– affected lymph nodes with capsular perforation (ECS) with 60–66 Gy with single doses of 2.0–2.2 Gy,*

*– affected lymph node levels without capsular perforation with 54–60 Gy with single doses of 1.8–2.0 Gy, and*

*– unaffected lymph node levels with 45–54 Gy with single doses of 1.5–1.8 Gy.*

*The elective lymph node levels to be irradiated should be based on the current international consensus.*

- EC
- Strong consensus

**8.83 Consensus-based recommendation 2024**

*Adjuvant radio- or radiochemotherapy of hypopharyngeal carcinomas in stage III–IVb should be carried out using the IMRT technique with the best possible protection of the salivary glands, the unaffected swallowing tract and the oral cavity, without falling below the recommended doses in the target volumes.*

- EC
- Strong consensus

**8.84 Consensus-based recommendation 2024**

*Postoperative radiotherapy or radiochemotherapy should be started as soon as possible after the wound has healed and within 6 weeks of the operation.*

- EC
- Strong consensus

**8.85 Consensus-based statement 2024**

*The voice and swallowing function should be examined and documented pre-therapeutically during larynx-preserving therapy.*

- EC
- Strong consensus

##### Postoperative radiotherapy

The indication and implementation of adjuvant radiotherapy and radiochemotherapy do not differ in principle from those of HPV/p16-negative oropharyngeal carcinoma (see 8.1.4).

The effect of postoperative radiotherapy compared to no adjuvant radiotherapy has not been investigated in randomized studies. Results of cohort studies and prospective registry data indicate that in patients with more than one tumour-involved lymph node, with extracapsular tumour growth at the lymph nodes (ECE) or with tumours only barely resected in healthy tissue (<5 mm), adjuvant radiotherapy is clearly superior [319], [320], [321], [322]. These studies included squamous cell carcinomas of the larynx, oropharynx, hypopharynx and oral cavity. The proportion of hypopharyngeal carcinomas was between 12% and 43%. The available data suggest that adjuvant radiotherapy reduces the locoregional recurrence rate and improves survival in hypopharyngeal carcinomas if more than one regional lymph node is affected or if the primary tumour was resected just inside the healthy area (<5 mm). For patients with pT3/pT4 tumours, lymph node involvement with ECE or R1 resection, there are no reliable data on treatment outcomes without adjuvant radiotherapy for hypopharyngeal carcinoma, as there is an international consensus that these patients require adjuvant radiotherapy [325].

For patients with an intermediate risk of recurrence (no ECE and resection ≥5 mm in healthy tissue), 60 Gy (5x2 Gy per week) was applied independently in the region of the former primary tumour and the affected lymph nodes in the vast majority of patients in the clinical trials and the registry data. In the adjacent, unaffected lymph node regions, 45–50 Gy with 5x(1.8–2.0) Gy per week were administered electively. There is an international consensus for the selection of the lymph node levels to be electively irradiated, which can be regarded as a standard guideline [276]. IMRT techniques are also considered standard for postoperative radiotherapy.

##### Postoperative radiochemotherapy

The value of additional cisplatin-containing chemotherapy administered simultaneously with radiotherapy has been investigated in 3 large randomized trials for locally advanced squamous cell carcinoma of the oropharynx, hypopharynx, larynx and oral cavity [326], [327], [328]. The proportion of hypopharyngeal carcinoma in these studies was between 10% and 20%. Subgroup analyses by tumour location are not available from these studies. In the registry studies on hypopharyngeal carcinoma, the effect of postoperative radiochemotherapy was not compared with the effect of postoperative radiotherapy alone. Nevertheless, it seems plausible to assume that the results of the 3 randomized studies also apply to hypopharyngeal carcinomas, even if it cannot be ruled out that the effect sizes may differ slightly from those of the other tumour localizations. The results of the studies consistently showed that patients with evidence of ECE in the affected lymph nodes or an R1 resection (defined in these studies as resection <5 mm in healthy tissue) have a survival advantage after adjuvant radiochemotherapy compared to adjuvant radiotherapy alone, so that postoperative radiochemotherapy is considered standard for these patients.

If postoperative radiochemotherapy is indicated, it should be noted that results of randomized studies are only available for cisplatin or cisplatin + 5-FU, which have shown a significant reduction in the relapse rate or an improvement in survival [326], [327], [328]. In a randomized Japanese study (n=261), 3 x100 mg/m² cisplatin at intervals of 3 weeks were compared with weekly 40 mg/m² cisplatin simultaneously with postoperative radiotherapy [338]. The non-inferiority of the weekly administration of cisplatin was demonstrated. The proportion of patients with hypopharyngeal carcinoma in this study was 34%.

For patients who cannot receive cisplatin, e.g. due to impaired renal function, there are little data available for postoperative radiochemotherapy. For mitomycin C, data are available from two small randomized studies that only showed a trend towards a survival benefit [297], [339]. The weekly administration of docetaxel simultaneously with radiotherapy was compared with radiotherapy alone in a randomized study [291]. In a small subgroup of this study, the therapy was also given in the postoperative situation. There was a trend towards improved survival for the combined therapy. No results of randomized studies are available for carboplatin + 5-FU and carboplatin + paclitaxel in the postoperative situation. However, it seems plausible to use these combinations in the postoperative situation by analogy with the efficacy of these combinations in primary radiochemotherapy (see 8.5.3). There are no data suggesting a benefit for the use of cetuximab in the postoperative situation.

In postoperative radiochemotherapy in the studies described, the regions with the highest risk of recurrence (lymph nodes with ECE and areas of scarce resection) were irradiated with 5x2 Gy per week up to 66 Gy. The adjacent regions and electively irradiated lymph node sites also received between 54 and 60 Gy in conventional fractionation [326], [327]. The frequency of grade III/IV late side effects in the US study [326] after radiochemotherapy was between 1–6% higher than after radiotherapy alone in various categories, although no statistical significance was achieved. To reduce side effects, postoperative radiotherapy or radiochemotherapy is now routinely carried out using IMRT techniques.

#### 8.5.5 Neoadjuvant and adjuvant drug-based tumour therapy 

Induction chemotherapy prior to radiotherapy has a firm place in the organ-preserving treatment of hypopharyngeal carcinoma, which can only be treated curatively by laryngopharyngectomy. The first large randomized study and the only one to directly compare non-surgical with surgical treatment was initiated by the EORTC and recruited 202 patients with untreated, operable hypopharyngeal (78%) and laryngeal (22%) carcinoma [233]. We therefore speak of level IA evidence for an induction protocol in advanced hypopharyngeal carcinoma. In contrast, the primary radiochemotherapy recommended for laryngeal carcinoma for laryngeal organ preservation [353] is also relevant for hypopharyngeal carcinoma by analogy (RTOG 91-11 only recruited laryngeal carcinomas), but only level IVA evidence due to the lack of a direct comparative study [381] (more details in Section 8.5.3 and [2]).

#### 8.5.6 Hypopharyngeal carcinoma stage IVC

In the case of a tumour that is already distantly metastasized at the time of initial diagnosis, the limited curative treatment options and the very limited prognosis must be strictly weighed in the interdisciplinary tumour board. The treatment options are subsumed under the generic term of palliative medical treatment. As the principles in these tumour standards apply equally to squamous cell carcinomas of the oral cavity, oro- (p16-positive and p16-negative), hypopharynx and larynx, reference is made to the S3 Guideline on Oral Cavity Carcinoma, Version 3.0, Chapter 8.9 for further recommendations, in which the topic and recommendations are described in detail and agreed upon [1].

### 8.6 Neck dissection

**8.86 Consensus-based recommendation 2024**

*Both elective and therapeutic neck dissection should take functional aspects into account and preserve structures such as the accessory nerve, the sternocleidomastoid muscle and the internal jugular vein in addition to other non-lymphatic structures.*

- EC
- Strong consensus

**8.87 Consensus-based statement 2024**

*The preservation of the accessorius nerve during neck dissection leads to an improvement in quality of life.*

- EC
- Strong consensus

Neck dissection is an integral part of the primary surgical treatment of oropharyngeal and hypopharyngeal carcinoma. Sentinel node biopsy is not recommended as an alternative to elective neck dissection in the N0 situation for oropharyngeal and hypopharyngeal carcinomas (see also Section 7.6). Neck dissection is based on the Robbins classification, taking into account the neck level ([119]; see also Chapter 6.5).

- Radical (comprehensive) neck dissection (RND): resection level I–V incl. resection of the internal jugular vein (VJI) of the sternocleidomastoid muscle (SCCM) and the accessorius nerve
- Modified radical neck dissection (mRND): resection of levels I–V; preservation of one or more non-lymphatic structures of the RND (mRND type 1–3)
- Selective neck dissection (SND): retention of one or more LK levels of the RND; retention of the VJI, M.SCM and N. accessorius (new: SND I–III; obsolete: supraomohyoidal ND)
- Extended neck dissection

Obsolete terms such as “functional” or “supraomohyoid” neck dissection should no longer be used.

According to the current ASCO consensus, an adequate dissection should include at least 18 lymph nodes [429]. In principle, a distinction is made between elective neck dissection and curative (definitive) neck dissection. Elective neck dissection is considered in a cN0 situation. The indication for an elective neck dissection should be based on the risk of occult metastasis in the corresponding neck level. Curative neck dissection is used in the N+ situation. According to Robbins, a distinction is made between radical (comprehensive) and selective neck dissection, whereby the individual neck levels dissected should be specified for the latter. The type of neck dissection (radical or selective) is defined according to the preoperative clinical staging (for oropharynx independent of p16 and the associated different N classification) and according to the recommendation of the NCCN Guidelines (NCCN Guideline Version 2.2022) is based on the following formula:

- cN0: Selective neck dissection
  - Oropharynx Level II-IV
  - Hypopharynx Level II-IV and Level VI, if necessary
- cN1-cN2a-c: Selective or (modified) radical neck dissection
- cN3: (modified) Radical neck dissection (R0-resectable where appropriate)

In patients with lateralized oropharyngeal or hypopharyngeal carcinoma who undergo neck dissection at the same time or prior to transoral endoscopic head and neck surgery, ligation of endangered supply blood vessels should be performed in order to reduce the severity and incidence of postoperative bleeding [430], [431].

#### 8.6.1 Elective neck dissection

**8.88 Consensus-based recommendation 2024**

*In patients with T1 oropharyngeal carcinoma of the tonsil and lateral pharyngeal wall clearly located lateral to the midline and cN0 status, elective ipsilateral selective neck dissection (level IIa, III and IV) should be performed as part of a primary surgical procedure regardless of p16 status.*

- EC
- Strong consensus

**8.89 Consensus-based recommendation 2024**

*In patients with cT2 cN0 oropharyngeal carcinoma, at least one unilateral selective neck dissection (level IIa, III, IV) should be performed as part of a primary surgical procedure for strictly lateral localization regardless of p16 status.*

*In patients with tumours close to the midline and independent of the midline, all soft palate and tongue base carcinomas, a bilateral elective selective neck dissection (level IIa, III, IV) should already be performed in stage T1 as part of a primary surgical procedure, regardless of the p16 status.*

- EC
- Strong consensus

**8.90 Consensus-based recommendation 2024**

*In patients with cT1 cN0 hypopharyngeal carcinoma clearly located lateral to the midline and cN0 status, at least one elective ipsilateral selective neck dissection (level IIa, III and IV) should be performed as part of a primary surgical procedure.*

- EC
- Strong consensus

**8.91 Consensus-based recommendation 2024**

*In the case of cN0 hypopharyngeal carcinomas with tumour localization close to the midline or indications of deep tumour infiltration or from category T2, a bilateral elective selective neck dissection (level IIa, III, IV) should be performed in the case of a primary surgical approach.*

- EC
- Strong consensus

**8.92 Consensus-based recommendation 2024**

*In patients with lateralized oro-, hypopharyngeal carcinoma undergoing neck dissection simulta* *ne* *ousl* *y or prior to transoral endoscopic head and neck surgery, ligation/clipping of compromised supplying blood vessels may be considered to reduce the severity and incidence of postoperative bleeding.*

- EC
- Strong consensus

The indication for elective neck dissection in patients with oropharyngeal and hypopharyngeal carcinomas in tumour stages T1 and T2 with a clinically negative lymph node status depends largely on the expected occult cervical metastasis rate. Cervical lymph node sonography, computed tomography and/or magnetic resonance imaging are currently standardly used to determine the clinical N-status. The detection accuracy can be increased by advanced procedures such as positron emission tomography (comprehensive description of imaging in Section 7.3) and/or sentinel lymph node biopsy [432], [433]. Studies on sentinel lymph node biopsy indicate that it could be equivalent to elective neck dissection in terms of patient survival in patients with early-stage head and neck tumours with clinically negative neck lymph node status ([224], [434]; see also Section 7.6). Elective neck dissection is currently the gold standard for determining nodal status.

Currently, elective neck dissection is indicated for an expected occult metastatic probability of 15% to 20% [435], [436], [437], [438], [439], [440], [441]. If the expected occult metastasis rate is lower, watchful waiting can also be performed; if a neck dissection is nevertheless performed, the potential patient benefit and the expected morbidity must be carefully weighed against each other. Relevant complications that must be taken into consideration are, in addition to bleeding events, primarily sensory or motor nerve damage (especially of the accessorius nerve), chronic lymphedema, chyle fistulas and wound healing disorders [222], [430], [431], [442], [443], [444], [445], [446], [447], [448].

##### T1/2 cN0 oropharyngeal carcinoma

For oropharyngeal carcinomas in the early stages T1 and T2N0M0, the rates of occult lymph node metastasis described in the literature range from approximately 12–39% [437], [446], [449], [450], [451], [452], [453], [454], [455], [456], [457], [458], [459], [460]. The difference between patients with tumour size T1 compared to T2 is remarkable: for T1 tumours, the risk of occult lymph node metastases is less than 15%, while rates of around 30% are described for T2 and above. The latter corresponds to the occult metastasis rates in patients with primary tumours in the advanced tumour sizes T3 and T4. In the case of primary tumours clearly located laterally to the midline, there is also a difference in the lateral distribution of metastases. While ipsilateral occult metastases are found in 17–33% of patients with T1 and T2 oropharyngeal carcinomas across all stages, the rate for contralateral occult metastases is 0–29% [429], [439], [450], [456], [461], [462], [463], [464], [465], [466], [467], [468], [469], [470], [471].

Occult contralateral metastases are found in 0–20% of cases of T1 tumours and in 5–35% of cases of T2 tumours [439], [451], [464], [471], [472]. Anatomical differences within the oropharynx must also be taken into account: when the soft palate and the base of the tongue are affected, the rates of contralateral occult metastasis are higher than for tumours in the tonsil area [429], [464], [473], [474], [475], [476], [477], [478] due to the intersecting lymphatic drainage pathways. Occult bilateral metastasis rates of approximately 14% have been described for soft palate carcinomas as early as stage T1, and up to 20% for tongue base carcinomas. From stage T2, the rates are approximately 32% for soft palate carcinomas and approximately 35% for tongue base carcinomas [429], [439], [475], [477].

There are also clear differences with regard to the involvement of the lymph node levels: with cN0 status, occult lymph node metastases predominantly manifest themselves in the ipsilateral levels II and III. The distribution according to lymph node regions is as follows ipsilaterally: Level Ib: 0–9%, Level IIa: 26–75%, Level IIb: 5–14.9%, Level III: 0%–41%, Level IV: 0%–9%, Level V: 0–12% [437], [460], [464], [471], [479], [480], [481], [482]. A higher T-stage, extracapsular growth, clinically ipsilateral lymph node involvement, lymphangiosis carcinomatosa or oropharyngeal carcinoma of the tonsil increase the probability of ipsilateral level IIb involvement [437].

Overall, HPV-associated oropharyngeal carcinomas in tumour stages T1 and T2 metastasize lymphogenously more frequently than non-HPV-associated tumours [42], [483]. With regard to the influence of HPV association on the presence of occult metastases, no clear data are currently available: for example, Shoustare and Kato et al. describe a higher probability of occult contralateral metastases compared to non-HPV-associated tumours; in contrast, Shah, Amsbbaugh and Tritter et al. describe lower metastasis rates [460], [476], [477], [479], [484].

There are currently no randomized controlled phase III studies on the issue under discussion. Taking into account the above-mentioned study results, elective ipsilateral selective neck dissection is always indicated in patients with oropharyngeal carcinomas of the tonsil and the lateral pharyngeal wall that are clearly lateral to the midline, i.e. already at tumour size T1. In patients with stage T2, bilateral elective selective neck dissection should be performed. In patients with tumours close to the midline, soft palate and tongue base carcinomas, bilateral selective neck dissection is already recommended at stage T1. This applies to patients with both HPV-positive and HPV-negative oropharyngeal carcinomas. Both ipsilateral and contralateral elective selective neck dissection can be limited to levels II, III and IV [429], [473], [485].

##### T1/2 cN0 hypopharyngeal carcinomas

Hypopharyngeal carcinomas have a high occult ipsilateral metastasis rate of 24–41% regardless of the extent of the primary tumour in stage cN0 [447], [449], [486]. However, a cN0 stage is rarely found in affected patients at initial diagnosis; cervical lymph node metastases are usually already clinically manifest. With a primary tumour size of T1, the occult contralateral metastasis rate is 8% [487]. The location of the primary tumour significantly influences the contralateral metastasis rate: in T1 tumours of the anterior wall of the piriform sinus, the lateral hypopharyngeal wall or the posterior hypopharyngeal wall, the rate is around 5% [487], [488], while occult contralateral metastases are already present in around 13% of T1 cases with infiltration of the medial wall of the piriform sinus. Tumour involvement of the postcricoid region, the medial wall of the piriform sinus, tumour growth close to the midline in the area of the posterior pharyngeal wall or tumour infiltration of more than 1 mm in depth increase the risk of contralateral lymph node metastasis [487], [489], [490], [491], [492].

For primary tumours in stage T2, the rate of occult contralateral metastasis is up to 21% [493], [494]. This already corresponds to the rate for advanced tumour stages T3 and T4 [487]. The distribution of occult lymph node metastases according to metastasis level is as follows: Level Ib: 3.8%, Level IIa: 53.8%, Level IIb: 7.7%, Level III: 50%, Level IV: 19.2%, Level V: 7.7% [495].

There are currently no phase III studies available on the issue in question. Taking into account the above-mentioned study results, an ipsilateral elective neck dissection on the side affected by the tumour can be performed in patients with strictly unilateral tumour localization in the area of the hypopharyngeal side wall or posterior wall in stage T1. In the case of tumour localization close to the midline or indications of deep tumour infiltration or from category T2, bilateral elective neck dissection is recommended. Selective neck dissection of levels II, III and IV should be performed both ipsilaterally and contralaterally. Level VI neck dissections are performed in the hypopharynx as needed to resect the primary tumour and all clinically suspicious neck nodes. Elective dissection depends on the extent and location of the primary tumour.

#### 8.6.2 Curative neck dissection

**8.93 Consensus-based recommendation 2024**

*In nodal-positive oropharyngeal and hypopharyngeal carcinoma, at least ipsilateral curative neck dissection should be performed as part of a primary surgical proce* *d* *u* *re, regardless of the stage.*

- EC
- Strong consensus

**8.94 Consensus-based recommendation 2024**

*The extent of a curative neck dissection as part of a primary surgical procedure for nodalpositive oropharyngeal and hypopharyngeal carcinomas should be made dependent on the extent of lymph node involvement. The minimum extent includes selective neck dissection of level IIa, III, IV and can be increased up to radical neck dissection.*

- EC
- Strong consensus

Based on the basic locoregional metastatic behaviour of oropharyngeal and hypopharyngeal carcinomas in Section 8.6.1 and the current ASCO recommendations [429], curative neck dissection is indicated as an essential component of definitive surgery in the N+ situation. In the case of unilateral nodal-positive laterally located oropharyngeal and hypopharyngeal carcinoma, at least one ipsilateral curative neck dissection should be performed as part of a primary surgical procedure, regardless of the stage. If the tumour location is close to or even exceeds the midline, a bilateral neck dissection should be performed even in a unilateral cN+ situation. The extent of a curative neck dissection as part of a primary surgical procedure for nodal-positive oropharyngeal or hypopharyngeal carcinomas should depend on the extent of lymph node involvement on the respective side of the neck. The minimum extent includes selective neck dissection of level IIa, III, IV and can be increased to radical neck dissection [429].

In patients with lateralized oropharyngeal and hypopharyngeal carcinoma undergoing neck dissection simultaneously or prior to transoral endoscopic head and neck surgery, ligation of vulnerable supplying blood vessels should be performed to reduce the severity and incidence of postoperative bleeding. Patients with cN+ disease who have either clear extranodal extension into the surrounding soft tissue or involvement of the carotid artery or cranial nerve should be offered a non-surgical approach. Patients with biopsy-proven distant metastases should not undergo routine surgical resection of metastatic cervical lymph nodes [429].

For advanced hypopharyngeal carcinoma treated primarily by surgery, level VI dissection (including pretracheal lymph nodes, delphic lymph nodes and unilateral or bilateral paratracheal lymph nodes) and hemithyroidectomy up to total thyroidectomy may be indicated.

#### 8.6.3 Salvage neck dissection

The term “salvage surgery” is classified internationally as follows: *“Salvage surgery is no longer limited to patients who failed radiotherapy or radiochemotherapy, but also includes patients who previously underwent surgical treatment for tumours located from the base of the skull to the lower neck areas (including thyroid cancer)*” [496]. This means that even after sole surgical treatment of the initial carcinoma in the oropharynx or hypopharynx in the event of a new recurrence/second tumour or locoregional metastases in the former resection area, repeat surgery is referred to as salvage surgery. Salvage neck dissection is described in more detail in Section 9.1.1 as part of the more detailed considerations on salvage surgery.

Planned neck dissection after definitive radiochemotherapy for node-positive oropharyngeal and hypopharyngeal carcinomas is a special situation. Here, salvage neck dissection should only be considered immediately after primary therapy if the FDG-PET-CT proves positive. The background and recommendations were explained in detail in Chapter 7.3 (see recommendations 7.14–7.16, [201], [429]).

### 8.7 Special aspects of nursing care for oropharyngeal and hypopharyngeal carcinoma patients

While patients with oropharyngeal or hypopharyngeal carcinoma present with a complex medical picture, a wide range of nursing care needs can also be derived (see Table 6 (Tab. 6)). In the entire nursing process, which is reserved for nurses (*when we refer to nurses in these explanations, we always mean professionally or university-qualified nursing staff as defined by the Nursing Professions Act (PflBG)*) (§ 4 PflBG), activities come together that are oriented towards the needs of those affected and can be preventive, maintaining, promoting, healing, restoring or alleviating for the patients (§§ 5, 37 PflBG).

In addition to this broad range of tasks along the entire treatment and care process, the concept of health literacy also includes the need for caregivers to support those affected in their search for and understanding, evaluation and application of health information [497] and to help them achieve independence.

Based on this, the following explanations are dedicated less to the basic nursing activities (wound care, personal hygiene, mobilization, etc.) and more to the special nursing tasks in the presence of oropharyngeal or hypopharyngeal cancer. A total of four key areas were identified in the literature.

#### Information and advice

First and foremost is the topic of information and advice (see also Chapter 5). Several studies have identified a broad lack of knowledge about oropharyngeal and hypopharyngeal cancer among various population groups. Both healthy subjects and those affected (regardless of form and severity) had little or no knowledge about the aetiology, prevention and treatment of oral cavity or oropharyngeal tumours [498], [499], [500], [501], [502], [503], [504], [505], [506].

As this lack of knowledge is also cited as the primary reason for delayed diagnosis [507], this is an important area of action for nurses, as providing information and advice is one of the original nursing tasks and is a central component of patient education geared towards health literacy. Knowledge about cancer in general and about one’s own illness in particular can be significantly improved through individual adaptation and the use of digital media, among other things [504], [508]. Providing information and knowledge about the disease can also reduce emotional problems, anxiety and depression during the hospital stay for primary therapy [509].

##### Psychosocial support

At this point, another central aspect of nursing care should be mentioned: psychosocial support. Patients with head and neck tumours report increased anxiety and show increased depressive tendencies or depression, and their quality of life is also (significantly) lower. The diagnosis of depression also has an influence on the survival of a tumour disease in the head or neck area [510], [511], [512]. Furthermore, facial features indirectly altered by the carcinoma can put a strain on the individual psyche [512], [513]. Accordingly, targeted care process planning is required which, in addition to the selection of measures, their implementation and evaluation, also includes assessments that can provide targeted psychosocial support by taking individual needs and quality of life into account [514], [515], [516].

The social integration of patients is also very important in order to ensure a stable psychological situation [512]. This is because their role experience can change as a result of the illness [517], and their quality of life correlates significantly with problems in their social contacts [518]. Likewise, returning to active employment can sustainably improve the quality of life of those affected [519], which is why the controlled and systematic discharge of those affected and further care planning are of central importance [520].

Psychosocial support also includes the aspect of sexuality. The sexual activity and satisfaction of those affected can be restricted by physical changes, specific or non-specific anxiety or psychological stress [521], [522], [523]. These sexual needs must also be considered as part of comprehensive nursing care.

##### Nutritional situation

The quality of life of those affected also deteriorates if malnutrition is present [518], [524]. This leads to a third, central field of action for caregivers: the nutritional situation.

Patients with oropharyngeal or hypopharyngeal carcinoma have a high risk of malnutrition due to impaired chewing and swallowing function [523], [525]. If this is the case, those affected lose weight, they cope much less well with surgery, chemotherapy or radiotherapy and their treatment-related morbidity and mortality increase [526], [527], [528]. The more advanced the carcinoma, the greater the chewing and swallowing deficits, which in many cases leads to gastric tube or PEG insertion [529].

Accordingly, early recognition of nutritional needs (through screenings and regular assessments; Blumenberg et al. 2017) as well as continuous monitoring and nutritional support are essential for those affected [517], [525], [526], [527], [530], [531]. This support must also be guaranteed beyond inpatient treatment in the outpatient sector, which once again emphasizes the importance of targeted discharge management [520].

In addition to active support with food intake, regular training to maintain or restore chewing and swallowing function (e.g. through speech exercises, singing, tongue movements, pharyngeal stimulation) is one of the central tasks of caregivers [523], [532]. The application of nutrition via nasogastric tube or PEG is also used in various places [533] (see Chapter 10.4 for a detailed discussion of nutritional issues).

##### Dependence on addictive substances

Regular consumption of alcohol or cigarettes is often cited as a cause of oropharyngeal and hypopharyngeal carcinoma (for more details, see Chapter 4) [534], [535], [536]. The addictive behaviour often associated with this must be addressed by caregivers in a professional and targeted manner [537]. This includes, on the one hand, support in the controlled intake and supply of the respective addictive substance, and on the other hand, support in the (gradual) reduction as well as an individually adapted and agreed to complete withdrawal [534], [538]. The reduction or withdrawal from both addictive substances leads to better therapy results, better wound healing, lower mortality and better nutritional status [539], [540]. At this point, there is again a link to the initial need for information and advice, as reducing or quitting alcohol and/or cigarettes as a preventative measure can already significantly reduce the risk of disease [540].

## 9 Treatment recommendations for residual tumour, recurrence, second carcinoma and recurrent metastasis

When treating local or locoregional recurrences, second carcinomas or residual squamous cell carcinomas of the oropharynx after primary treatment, different initial situations arise. The majority of patients with locoregional recurrences have undergone pretreatment consisting of surgery and/or postoperative radio- or radiochemotherapy or primary radio- or radiochemotherapy. Unless it is a rare second carcinoma in a different (new) location (defined according to the criteria of Warren and Gates, 1932, which are still valid today: *1: each of the tumours is malignant, 2: each must be at least 3 cm locally distant from the first carcinoma and 3: the probability that one is a metastasis of the other must be excluded.* Field carcinogenesis, which has now been characterized by molecular biology, must also be taken into account. Overview: [541], we are generally talking about **new tumour growth in a previously treated area**, which must be taken into account in a special way when making treatment decisions. According to the recently presented Odense-Birmingham definition, the following criteria are proposed for a recurrent tumour: 1*: same anatomical subregion or adjacent subregion within 3 cm of the primary lesion, 2: time interval of occurrence not more than 3 years (from completed treatment of the primary lesion) and 3: same p16 status for oropharyngeal carcinoma *[542]*.* A secondary carcinoma can occupy an intermediate position, as it is often a manifestation with sufficient local distance from the primary carcinoma after more than 3 years, but can still occur within the radiation field of the primary lesion or the more extensive surgical field in primary therapy.

The biological background of tissue changes after pre-treatment is diverse. Locoregional recurrences most frequently grow in areas that have been pre-irradiated with high doses, but less frequently in areas that have only been pre-operated on and not irradiated, and generally represent a particular challenge for further treatment. The literature provides ample evidence of the special situation after radiotherapy and less evidence of the situation after primary surgical treatment alone. For example, it has been shown that the in vitro radiosensitivity of recurrent squamous cell carcinoma cell lines established from a pre-irradiated area is significantly lower than the radiosensitivity of primary squamous cell carcinoma cell lines [543]. In animal models, tumour cells introduced by injection into a tissue pre-irradiated at a higher dose (e.g. 20–30 Gy single-time dose) grow significantly slower into a tumour of the same size than in a non-pre-irradiated area [544], [545], which is referred to as the tumour bed effect. The reason for this is the reduced ability of the pre-irradiated tissue to provide sufficient neoangiogenesis for faster tumour growth. As a result, the tumours also tend to develop central tumour necrosis [546], i.e. tumour hypoxia, more quickly than in tissue that has not been pre-irradiated. This effect can also contribute to a reduced effect of radiotherapy or radiochemotherapy. In animal models, a significantly increased rate of the formation of distant metastases was also observed [544], [546]. However, these tumour bed effects in the mouse model weaken with increasing distance from the pre-irradiation and are only slightly pronounced after 200 days [544], [545]. In addition, there is the problem that the radiation tolerance of normal tissue is reduced when tumours in pre-irradiated tissue are re-irradiated. In an animal model, it has been shown that the tolerance of the cervical myelon in rhesus monkeys to re-radiation therapy increases again with a longer interval and is at least 61% of the original tolerance after 12 months [547]. For re-irradiation ± chemotherapy of recurrences in the pre-irradiated area, the initial conditions are probably worse than in the context of primary therapy due to the lower radiation sensitivity of the tumours with simultaneous reduced tolerance of the normal tissue in analogy to the preclinical observations described above. In the case of persistent tumours in the high-dose irradiated area of radio- or radiochemotherapy, re-irradiation is hardly an option in the first 3–6 months, not only because of the low re-tolerance of the normal tissue, but also because of the obvious resistance of the irradiated tumours. With a longer interval (at least 6 months, preferably >1 year) to pre-irradiation and in the case of independent second tumours, the data from the animal models suggest somewhat more favourable starting conditions for re-irradiation ± chemotherapy.

In general, it should be noted that the treatment of recurrent tumours and second carcinomas without distant metastases (any T, any N, not M1) after completion of primary therapy follows the same principles for both oro- (HPV-independent) and hypopharyngeal carcinomas – with special consideration of the changed initial situation in the pre-treated area – as primary therapy (Chapter 8). The previous therapy (surgery, primary or adjuvant radiotherapy or radiochemotherapy) and the particularities of renewed tumour growth in the previously treated area (scar recurrence; Section 9.1.1) must be taken into account. Unfortunately, recurrences after primary treatment in the oropharynx and hypopharynx are significantly less favourable prognostically than in the oral cavity and larynx [548], [549], [550], [551]. It is therefore always necessary to check whether surgical treatment (R0 resection; salvage surgery) and, if necessary, adjuvant radio- or radiochemotherapy or definitive radio- or radiochemotherapy is still possible. If the locoregional recurrence is in an area that has been pre-irradiated with a higher dose, a new course of radiotherapy or radiochemotherapy can only be given with the understanding that the risk of late side effects is increased and, in some cases, that the total dose is reduced. If a meaningful resection is no longer possible due to the extent of the tumour, the option of re-radiation ± chemotherapy is a potentially curative option [552], [553], [554], [555], [556], [557]. If neither resection ± radio- or radiochemotherapy, nor radio- or radiochemotherapy are feasible, there is the option of systemic therapy (drug-based tumour therapy). In drug-based tumour therapy, a distinction is made between first-line, second-line and possibly third-line therapy. First-line therapy is now internationally standardized (Section 9.2.1). In the case of distant metastases (M1), resection and targeted radiotherapy can be considered in the presence of individual metastases (oligometastasis). If this is also no longer possible, drug-based tumour therapy is also used in this situation. In the case of distant metastases, this can also be combined with targeted radiotherapy of metastases and, if necessary, resection.

**Observation of the tissue situation in the pre-treated area: after surgery alone without radiotherapy:**

There is little data on the growth behaviour of locoregional recurrences in non-pre-irradiated tissue after surgery for oropharyngeal or hypopharyngeal carcinomas, and the available data are mainly for laryngeal carcinoma. In a case series of 29 recurrences of laryngeal carcinomas treated only surgically, the recurrent tumours predominantly showed diffuse, multicentric, and more frequent submucosal growth in different regions of the larynx, and in about half of the cases their extent was underestimated in imaging [558]. It is consistent with clinical experience and seems plausible that this altered growth pattern of recurrent tumours is also present in the majority of cases of oropharyngeal and hypopharyngeal carcinomas and makes it very difficult to determine the exact extent of the tumour. Whether the radiosensitivity of recurrent tumours that grow in non-irradiated tissue that has been scarred/altered by surgery increases has been little studied to date. An intrinsically reduced radiosensitivity at the cellular level does not appear plausible. However, scarring could lead to impaired blood flow, which would increase the likelihood of tumour hypoxia and the associated reduced radiosensitivity. Studies on patients have shown that keloids are usually hypoxic and hyperplastic (red) scars often have hypoxic areas. In contrast, no hypoxic areas were found in the area of white scars [559], [560], [561]. The data suggest that hypoxic areas are not regularly present in healed scar tissue. The initial conditions for a good efficacy of radio- or radiochemotherapy are therefore better than after pre-irradiation, especially as there is also an unrestricted radiation tolerance of the normal tissue. However, the diffuse, often multicentric and submucosal tumour growth increases the risk of underestimating the extent of the tumour, which worsens the conditions for both salvage surgery and radio- or radiochemotherapy compared to primary therapy in an untreated area.

The considerations described above should not be underestimated in individual cases in view of the widespread lack of randomized studies and larger prospective registry data in the treatment of locoregionally limited recurrences of squamous cell carcinoma of the oropharynx and hypopharynx.

### Definition of salvage surgery

In this context, the term “salvage surgery” should be explained in more detail, as it is commonly used to describe rescue surgery following failed radiotherapy. The term salvage surgery has become very prominent in the context of laryngeal organ preservation programs, in which laryngectomy was performed as salvage after tumour persistence or short-term recurrence following successful radiochemotherapy. However, current scientific consensus defines the term more broadly: “*Salvage surgery is no longer limited to patients who failed radiotherapy or radiochemotherapy, but also includes patients who previously underwent surgical treatment for tumours located from the base of the skull to the lower neck areas (including thyroid cancer)*” [496]. This means that even after sole surgical treatment of the initial carcinoma in the head and neck area in the event of a new recurrence/second tumour or locoregional metastases in the former resection area (scar), repeat surgery is also referred to as “salvage surgery”.

### 9.1 Treatment recommendation if surgery or radiation is still an option

**9.1 Consensus-based statement 2024**

*From a radiotherapeutic and surgical perspective, the biological characteristics of tumour recurrences or second carcinomas in previously treated areas cannot be equated with therapy-naïve, i.e. non-pretreated tissue situations in primary therapy.*

- EC
- Strong consensus

In the special consideration of recurrence or second tumour therapy, some basic considerations/approaches have currently prevailed [562], which are listed below.

- From a radiotherapeutic and surgical point of view, the biological characteristics of tumour recurrences or second carcinomas in previously treated areas cannot be equated with therapy-naïve, i.e. non-pretreated tissue situations in primary therapy.
- In general, it should be noted that the treatment of recurrent tumours, secondary carcinomas without distant metastases (any T, any N, not M1) after completion of primary treatment of oropharyngeal and hypopharyngeal carcinoma follows the same principles as primary treatment with regard to testing R0 operability and radiation or radiochemotherapy capability – taking particular account of the changed initial situation in the pre-treated area.
- In the case of locoregional oropharyngeal and hypopharyngeal carcinoma recurrences or second carcinomas, the possibility of surgical treatment in patients without distant metastases should always be reviewed and, if R0 resection is likely to be achievable, favoured in the multidisciplinary tumour board.
- The term “salvage surgery” is no longer limited to indications in which radiotherapy or radiochemotherapy has failed, but also includes the treatment of patients who have previously undergone surgical treatment of tumours alone.
- Salvage surgery for oropharyngeal and hypopharyngeal carcinomas, especially in pre-irradiated areas, is subject to significantly higher complications, such as wound healing disorders, wound infections, secondary healing, persistent fistula formation, etc., than primary surgery in non-pretreated tissue and should therefore include reconstructions with transfer of non-pretreated tissue.
- The initial conditions for good efficacy of salvage surgery, radio- or radiochemotherapy in oropharyngeal and hypopharyngeal carcinoma recurrences or secondary carcinomas are better in solely preoperated areas than after additional pre-irradiation or primary radiochemotherapy, but worse overall than in therapy-naïve tissue.
- The diffuse, often multicentric and submucosal tumour growth in pre-treated areas increases the risk of underestimating the tumour extent and the actual degree of hypoxia, which worsens the conditions for salvage surgery as well as radio- or radiochemotherapy compared to primary therapy.
- Due to scarring in the previously treated area (“scar recurrence”), it is often difficult or impossible to show the exact extent of the tumour on imaging. Biopsy confirmation of the recurrence can be difficult due to the diffuse extension in the pre-treated area, as the tumour often eludes direct visualization during panendoscopy.
- If an urgent oropharyngeal or hypopharyngeal carcinoma recurrence/secondary tumour is suspected in pre-treated tissue and a targeted biopsy is not expedient due to diffuse tumour spread that is not visible externally/endoscopically, exploratory salvage surgery with limited resection of suspicious masses can be performed both in the pre-treated primary tumour and in the neck area for histological confirmation and visualization of the tumour spread.
- Recurrences after primary treatment in the oropharynx and hypopharynx are significantly less favourable prognostically than in the oral cavity and larynx.

#### 9.1.1 Possibility of salvage surgery including neck dissection, adjuvant and primary radio- and radiochemotherapy

**9.2 Consensus-based recommendation 2024**

*In principle, a biopsy or, depending on the scar, a limited excisional biopsy should be performed to histologically confirm a recurrent tumour/secondary carcinoma in a previously treated area.*

*If a targeted biopsy is not expedient as part of the clarification of an urgent suspected recurrent/secondary tumour in pre-treated tissue due to diffuse tumour spread that is not visible externally/endoscopically, exploratory salvage surgery with limited resection of suspicious masses in the oropharynx/hypopharynx as well as in the neck area can be performed for histological confirmation and visualization of the tumour spread.*

- EC
- Strong consensus

**9.3 Consensus-based recommendation 2024**

*In the case of locoregional recurrence in the pre-treated area, it should be checked whether a functionally meaningful R0 salvage resection is possible.*

- EC
- Strong consensus

**9.4 Consensus-based recommendation 2024**

*In the case of locoregional recurrences or double carcinomas in the oropharynx or hypopharynx in the pre-operated but not pre-irradiated area, resection ± adjuvant radio- or radiochemotherapy should be performed if R0 resection is reasonably possible, or primary radio- or radiochemotherapy should be performed.*

- EC
- Strong consensus

**9.5 Consensus-based recommendation 2024**

*In the case of locoregional recurrences or double carcinomas in the oropharynx or hypopharynx in the pre-irradiated area, salvage resection should be preferred to non-surgical treatment if R0 resectability is functionally feasible.*

*Postoperative re-irradiation can be offered in particular after salvage resection if the following criteria are met:*

*– A long interval to the 1* *^st^* *radiation series (at least* *6 mo* *nths)*

*– No grade IV late effects of the 1* *^st^* *radiation series in the area to be re-irradiated*

*– No major wound healing disorders after salvage resection*

*– If risk factors are present (e.g. resection <5 mm i.G., >1 affected LK or ECE)*

- EC
- Strong consensus

**9.6 Consensus-based recommendation 2024**

*If post-operative re-irradiation (possibly with combined chemotherapy) is performed after salvage resection, a total dose of at least 50 Gy (R0)–60 Gy (R1/2) in 2 Gy equivalent dose for an alpha/beta value of 10 Gy should be aimed for.*

*In this situation, the CTV should be kept as small as reasonably possible (e.g. former tumour region* *5–10 mm* *) in order to minimize the volume of normal tissue that cumulatively receives >100 Gy.*

*Simultaneous chemotherapy can be selected analogous to primary radiochemotherapy. With a short interval (6–12 months) to the previous chemotherapy, an alter* *n* *a* *tive chemotherapy regimen should be preferred.*

- EC
- Strong consensus

**9.7 Consensus-based statement 2024**

*In the case of a locoregional recurrence of the cervical lymph nodes of an original oropharyngeal (regardless of HPV status) or hypopharyngeal carcinoma in a pre-irradiated/pre-operated area, a salvage neck dissection should be performed if functionally feasible.*

- EC
- Strong consensus

The recommendation for the practical procedure in the case of locoregional recurrence (tumour and cervical lymph nodes) is based on the limited data presented above and clinical experience compiled in textbooks. Unfortunately, most of the groups are mixed, i.e. primary surgery ± adjuvant radiotherapy or radiochemotherapy, or pre-treated with radiotherapy, so that the current data situation does not allow a clear standardized recommendation for the best treatment of locoregional recurrence in a pre-treated area. Randomized studies directly comparing salvage surgery ± postoperative radio- or radiochemotherapy with radio- or radiochemotherapy are not available. Data from larger prospective registry studies are also not available. Therefore, the assessment of treatment effects is limited to case collections with mostly small case numbers (30–250) of mixed patient collectives [551], [563], [564], [565] that most frequently received resection with postoperative radio- or radiochemotherapy or primary radio- or radiochemotherapy as part of primary therapy (i.e. not recurrence therapy) as well as a few patients who were pretreated with surgery alone. Locoregional recurrences classified as resectable were predominantly treated surgically, whereas recurrences classified as non-resectable were treated with radio- or radiochemotherapy including brachytherapy or with chemotherapy alone or only with “best supportive care”. This means that there is a clear bias in these case series to the disadvantage of non-surgical treatment methods, which should be taken into account in the already sparse data situation. However, if the results of different treatment methods are compared on this basis, meta-analyses show a clear survival advantage (10–30%) for salvage surgery compared to non-surgical treatment methods [548], [566], [567], [568], [569]. It should be noted that the majority of patients in these case series received postoperative radio- or radiochemotherapy despite pre-radiation. If R0 resection is successful, overall survival 5 years after salvage surgery is between 20–40% in the meta-analyses [548], [566], [568] and even 50–60% for HPV16/p16-positive oropharyngeal carcinomas [568], thus demonstrating the high curative potential of the salvage surgery procedure. In the case of R1 resection [548], or if lymph node metastases with extracapsular growth are detected, the prognosis is significantly worse [565]. In contrast, a long interval between initial therapy and recurrence therapy is a favourable prognostic factor [568]. Second tumours have a better prognosis than recurrent tumours [570].

Despite all the previously described limitations of the data on the treatment of locoregional recurrences of oropharyngeal and hypopharyngeal carcinomas, the possibility of surgical treatment in patients without distant metastases should be reviewed and favoured if R0 resection is likely achievable. In the case of locoregional recurrences or second tumours in non-pre-irradiated tissues, radio- or radiochemotherapy alone is a sensible alternative, both in the case of resectability limitations and given resectability with a tumour extent that is clearly defined on imaging and can be easily confirmed via biopsy.

##### Recurrence or second tumour in previously treated scarred tissue

When surgically treating tumour recurrences or second carcinomas in previously treated tissue (scar carcinomas), the aspects discussed above must be taken into account from a radiotherapeutic and surgical perspective, which can be disregarded in the primary treatment of non-pretreated tissue. This must be taken into account in particular for larger resection areas (neck dissection, T2-4 primary tumour resection with reconstruction). Altered lymphatic drainage of the pre-treated tissue, poorer blood circulation in scar tissue and the often observed poorer oxygen supply (hypoxia) are often sufficient reasons to assume limited radiation effectiveness (see introductory text 9.1). The exact extent of the tumour in scar recurrences cannot be reliably depicted by imaging. Biopsy confirmation of the recurrence can be difficult due to the diffuse extent of the scar, as the tumour often eludes direct visualization during panendoscopy. For this reason, uncritical direct application of the principles of primary therapy in a non-pretreated area is not recommended, particularly in the case of scarring. Therefore, exploratory salvage surgery with resection of suspicious masses both in the HNSCC primary tumour area and in the locoregional neck area after/instead of an unsuccessful biopsy attempt is justified for the above-mentioned reasons and is explicitly recommended by leading head and neck oncologists (the mandate holders of this guideline also agreed 100% with this recommendation). Often, the exact extent of the tumour can only be determined by such a procedure, in which reasonable salvage resections can be performed without prior frustrating biopsy attempts (e.g. limited neck dissection, resection of a scarred tonsil bed after previous surgery, etc.). In this particular situation, exploratory surgery affects diagnostic and therapeutic criteria in equal measure. In experienced hands, this procedure also leads to relevant time savings in an otherwise very lengthy and inappropriate staging procedure characterized by biopsies in the wrong place. In addition, the subsequent histopathological description of the extent of the tumour provides a more precise planning basis for a possible subsequent resection and targeted adjuvant radio/radiochemotherapy or re-radiation.

##### Special aspects of salvage laryngopharyngectomy

Salvage laryngopharyngectomy for recurrences in the hypopharynx after primary radio(chemo)therapy is often the only curative treatment option for patients with advanced hypopharyngeal carcinomas and is an integral part of multimodal therapy for advanced hypopharyngeal carcinomas. Salvage laryngopharyngectomy is characterized by a more difficult intraoperative preparation, as the radiotherapy in the tissue leads to a scarred transformation and reduced blood supply to the tissue (analogous to chapter 7.7. S3 larynx guideline [2]). Salvage surgery, especially in pre-irradiated areas, is subject to a significantly higher degree of complications, such as wound healing disorders, wound infections, secondary healing, persistent fistula formation, etc., than surgery in non-pretreated tissue. In general, free and pedicled tissue transfer with non-pretreated, well-vascularized tissue and competent wound management are recommended [571], [572], [573], [574], [575], [576], [577], [578]. There is no consensus in the literature as to which tissue transfer is optimal, particularly in the case of salvage laryngopharyngectomy for hypopharyngeal carcinoma recurrence/residual tumour after primary laryngeal organ-preserving multimodal therapy (reference to Section 7.7 of the S3 Guideline on Laryngeal Carcinoma [2]). Almost exclusively retrospective cohort studies are available. Most studies are available on the pectoralis major flap [579], [580]. A few studies compare two different flap plasty techniques [580], [581]. In a retrospective cohort analysis of 359 patients regarding pharyngeal fistulas after salvage laryngectomy, it was shown that the fistula rate after reconstruction using a pectoralis major flap (15%) was significantly lower than after reconstruction using a free flap (25%) or primary wound closure (34%). In patients who developed a fistula, the persistence of the fistula was significantly longer with primary closure (14 weeks) compared to pectoralis major flap reconstruction (9 weeks) or free flap reconstruction (6.5 weeks) [580]. A more recent study by Piazza et al. [582] described impressively low fistula rates after salvage surgery with free tissue transfer: 55 patients (mean age 66 years; male-to-female ratio 8:1) were included in the study. Prior treatments were radiotherapy in 22 (40%) patients, radiochemotherapy in 21 (38.2%) and partial laryngectomy followed by adjuvant (C)RT in 12 (21.8%). Reconstruction was achieved by radial forearm and anterolateral thigh flap (ALT) plasty in 16 (29.1%) and 39 (70.9%) patients, respectively. The success rate of flap plasty was 98.2% with only three pharyngocutaneous fistulas (5.4%) and one pharyngoesophageal stenosis (1.8%) [582].

It can therefore be concluded that in the context of salvage laryngopharyngectomy, the introduction of tissue from the non-irradiated area can significantly reduce the risk of fistula, although there is no evidence on the type of tissue transfer [580], [583], [584], [585], [586]. We now know from numerous recent reports that a hypopharyngeal carcinoma that is in principle resectable after primary radiochemotherapy does not necessarily have to be salvage-operable. According to recent reports, less than half of the previously resectable tumours proved to be no longer resectable/operable after radiochemotherapy [587]. Further information on the current techniques of reconstructive head and neck surgery was provided in Chapters 8.1.1 and 8.2.1.

For the recurrence detection of oropharyngeal and hypopharyngeal carcinomas in general, the few usable studies identified by IQWiG showed that PET had a significantly higher pooled sensitivity than the combination of CT and/or MRI in the technology comparison PET vs. combination of CT and/or MRI. Here, the specificity is reduced by false positive findings due to accumulation in inflammatory lesions. However, FDG-PET showed a higher reliability with a sensitivity of 100% and a specificity of 61–71% than CT and/or MRI [172] (Section 7.3).

Planned neck dissection after definitive radiochemotherapy for node-positive oropharyngeal and hypopharyngeal carcinomas is a special situation. Here, salvage neck dissection should only be considered immediately after primary therapy if the FDG-PET-CT proves positive. The background and recommendations were explained in detail in chapter 7.3 (see recommendations 7.14–7.16, [201]).

##### Postoperative radiochemotherapy after salvage surgery

The value of postoperative radiochemotherapy after salvage surgery was investigated in one small randomized study (n=130) [588]. A statistically significant and clinically relevant advantage in locoregional tumour control and relapse-free survival was demonstrated by adjuvant radiochemotherapy, but a statistically significant survival benefit could not be demonstrated in the small number of cases. Radiochemotherapy was administered using the Chicago regimen, which is rarely used in Europe, with 60 Gy in 30 fractions over 11 weeks in combination with simultaneous 5-fluorouracil and hydroxyurea. In a multi-institutional evaluation of several US centres (216 patients), postoperative radiotherapy ± chemotherapy (70%, predominantly cisplatin-based) with total doses >60 Gy did not show any benefit over total doses of 40 Gy–60 Gy in terms of survival and locoregional relapse rate [589]. The rate of ≥ grade 3 late side effects was 24% for total doses <60 Gy compared to 32% for total doses above 60 Gy. The majority of patients in this case series were irradiated with 5x1.8–2.0 Gy per week, a smaller proportion with 2x1.5 Gy per day 5x per week. With regard to the locoregional relapse rate, there was a trend (13% lower) in favour of hyperfractionated radiotherapy. Elective irradiation of unaffected adjacent lymph node levels did not improve the results.

##### Radio- or radiochemotherapy for functionally impractical R0 resectability

If R0 resection does not appear to be feasible for locoregional recurrences and second carcinomas, the possibility of radio- or radiochemotherapy should be examined for patients without distant metastases. For previously irradiated patients who were treated with re-radiation (in some cases also with brachytherapy) ± chemotherapy without prior salvage surgery, overall survival after 2 years of 30% [563], [590] to 41% [591] and after 5 years of 16% [566] were reported in meta-analyses. In the majority of case series, total doses of 45 Gy–70 Gy (median 60 Gy) were achieved in conventional fractionation (1.8–2 Gy single dose) or, in some series, hyperfractionated radiotherapy with 2x1.5 Gy per day. Several case series have shown better survival and a lower locoregional recurrence rate with total doses of >50 Gy [592] or >60 Gy [556]. No advantage was seen for hyperfractionated radiotherapy with 2x1.5 Gy per day. In a larger case series (n=216), the effect of elective irradiation of adjacent, unaffected lymph node regions was investigated [556] and no benefit was found. Smaller case series on the use of hypofractionated stereotactic radiotherapy with total doses of 10–50 Gy in 1–8 fractions reported survival rates comparable to those of conventional fractionation [590]. Overall, however, there is significantly more experience with conventionally fractionated reirradiation. Re-irradiation with protons has theoretical advantages over photon therapy in terms of dose distribution. The first major published case series (n=242) showed results that are in the upper range of the data published with photon therapy [593]. Simultaneously with re-irradiation, systemic therapy with cisplatin, carboplatin, cetuximab, docetaxel, paclitaxel, 5-FU, capecitabine, hydroxyurea or a combination of two of these substances was used in the majority of patients in the aforementioned case series. It is not known which substances are most effective in combination with reirradiation and whether there is any benefit at all. However, due to the limitation of the total dose and volume of reirradiation, additional systemic therapy is considered a sensible option and was therefore frequently used in the case series cited. In particular, if no cisplatin was used during primary treatment, it makes sense to apply cisplatin-containing chemotherapy in combination with reirradiation. To date, there are no reliable data on the use of induction therapies prior to reirradiation ± simultaneous chemotherapy and the use of immune checkpoint inhibitors in combination with reirradiation.

The acute toxicity of re-irradiation ± chemotherapy does not differ significantly from the toxicity of therapy in patients who have not been previously irradiated. However, an increased risk of late toxicities is to be expected. The published rates of ≥3 late effects are between approximately 10% and 40% in most studies [570], [590], [591], [594]. The more frequent grade 3–4 late effects include trismus, fibrosis, pharyngeal stenosis and fistulas as well as osteoradionecrosis (up to 17%) [588]. Treatment-related deaths have been observed with these therapies in 0.1–8.4% of patients [590], [594]. Mass bleeding from the carotid artery (carotid blowout syndrome) accounts for only a small proportion of these deaths, unless the carotid artery is largely overgrown by the recurrent tumour [595]. The grade 3–4 late side effects are often associated with a reduced quality of life due to predominantly poorer swallowing function. Total doses of re-irradiation of more than 60 Gy in conventional fractionation are associated with an approximately 10% increased risk of grade 3–4 late effects in both postoperative irradiation and definitive irradiation [556]. In the collectives that received postoperative re-radiation ± chemotherapy, grade 3–4 late effects are on average approximately 5–20% higher than with re-radiation ± chemotherapy alone [556], [588]. When deciding which patients are suitable for re-irradiation ± chemotherapy, it should be noted that in all of the studies and case series reported here, patients with grade 4 late effects of pre-irradiation were not treated with re-irradiation. In addition, the interval from the first radiotherapy in almost all patients was >6 months.

There are indications from some case series that grade 4 side effects occur more frequently in tissues that have been exposed to a cumulative dose of more than 120 Gy (2 Gy equivalent dose with the alpha/beta value of the respective normal tissue) [596]. This also applies to the “carotid blowout syndrome”. Volumes receiving cumulative total doses of >120 Gy should therefore be kept as small as possible. Total cumulative doses of >130 Gy to a maximum of 137 Gy have only been reported in exceptional cases in the available case series [591]. The greater the distance from the previous irradiation and the fewer late effects from the previous therapies, the lower the risk of severe late effects can be estimated. The American Radium Society has published additional information on this topic [554]. The influence of additional simultaneous chemotherapy on the higher-grade late effects of re-radiation is insufficiently documented in the case series to make a conclusive assessment in this regard.

##### Radio- or radiochemotherapy in non-irradiated patients with recurrences after surgical therapy alone

Very little data is available on the effect of primary radiotherapy or radiochemotherapy in patients with recurrences after surgery alone who have not received prior radiotherapy. In a collection of cases (n=75) of locoregional recurrences of hypopharyngeal and laryngeal carcinomas [597], a 5-year survival proportion of 76% was observed after approximately 70 Gy in 2 Gy single dose ± cisplatin-containing chemotherapy. This result is only slightly below the results expected in patients who were not previously treated. In this situation, radio- or radio-chemotherapy should be given according to the same principles and recommendations as for primary therapy (see Chapter 8), taking into account the possible altered lymphatic drainage due to the surgical procedure and the scar situation (as explained in detail above). In this particular situation, preference should therefore also be given to salvage surgery ± adjuvant radiotherapy or radiochemotherapy, where this appears feasible.

#### 9.1.2 Radiation reserve, re-irradiation

**9.8 Consensus-based recommendation 2024**

*In the event of re-radiation, patients should be informed about the significantly increased risk of grade IV late effects of radiotherapy.*

- EC
- Strong consensus

**9.9 Consensus-based recommendation 2024**

*In the case of locoregional recurrences or second cancers in the oropharynx or hypopharynx in the previously irradiated area, where R0 resection is not feasible, re-irradiation can be carried out, if possible in combination with simultaneous chemotherapy, if the following criteria are met:*

*– a long interval to the 1* *^st^* *radiation series (at least* *6 months* *)*

*– no grade IV late effects of the 1* *^st^* *radiation series in the area to be re-irradiated*

*Simultaneous chemotherapy can be selected in the same way as primary radiochemotherapy. With a short interval (6–12 months) to the previous chemotherapy, an al* *ter* *n* *a* *tive chemotherapy regimen should be preferred.*

- EC
- Strong consensus

**9.10 Consensus-based recommendation 2024**

*If re-irradiation is carried out with curative intent for a locoregional recurrence/secondary carcinoma, a total dose of approximately 60 Gy (2 Gy equivalent dose for an alpha/beta value of 10 Gy) should be aimed for.*

*In this situation, the CTV should be kept as small as reasonably possible (e.g. GTV + 5 mm) in order to mi* *ni* *m* *i* *ze the volume of normal tissue that cumulatively receives >100 Gy.*

*In the event of re-irradiation, patients should be informed of the significantly increased risk of grade IV late effects of radiotherapy.*

- EC
- Strong consensus

### 9.2 Treatment recommendation in case of non-operability or radiation option

Patients with a good general and performance status should be given palliative, usually cisplatin-based, systemic therapy if the options of salvage surgery and/or radio- or radiochemotherapy have been exhausted or are no longer possible. In patients with advanced, relapsed or metastatic oropharyngeal or hypopharyngeal carcinoma, response rates of 10–43% can be achieved with palliative chemotherapy, possibly in combination with immuno-checkpoint inhibitors [598], [599]. The selection of the active substances to be used currently depends on the expression of PD-L1 in the tumour tissue (CPS, TPS relevant for the immuno-checkpoint inhibitor pembrolizumab; see Section 7.5.2), the remission pressure at the time of therapy indication and the question of whether cisplatin-based radiochemotherapy has already taken place within the last 6 months. If more than 6 months have passed since the previous platinum-containing first-line combination, renewed platinum therapy (possibly also as a combination) can also be considered [600].

**Special consideration of systemic drug therapy in recurrent or metastatic oropharyngeal carcinoma: influence of HPV status:**

In patients with metastatic/recurrent oropharyngeal carcinoma, chemotherapy with docetaxel + cisplatin + cetuximab (TPEx) shows a survival benefit for patients with p16-positive compared to patients with p16-negative tumours (HR 0.61) [601]. For the combination FU + cisplatin + cetuximab (EXTREME), a difference in survival between patients with p16-positive versus p16-negative tumours has not been proven due to insufficient case numbers [602]. As the studies were not powered for this question, the significance is limited [603]. In patients with metastatic/relapsed oropharyngeal carcinoma, immunotherapy with PD1 or PD-L1 inhibitors (nivolumab, pembrolizumab, durvalumab) has shown no difference between patients with p16-positive and p16-negative tumours with regard to the endpoint “overall survival”. However, the response to immunotherapy is generally better in p16-positive tumours than in p16-negative tumours [604], [605], [606].

#### 9.2.1 “First line” drug-based tumour therapy

**9.11 Consensus-based recommendation 2024**

*The antibody pembrolizumab, which is directed against the PD-1 receptor, is to be used in patients with metastatic or recurrent oropharyngeal and hypopharyngeal carcinoma with PD-L1-expressing tumour and immune cells (CPS=1) that cannot be treated locally as first-line monotherapy (especially in CPS*≥*20 and with low disease burden) or in combination with platinum and 5-fluorouracil.*

- EC
- Strong consensus

**9.12 Consensus-based recommendation 2024**

*After 4–6 cycles of such combination therapy (recommendation 9.11), maintenance therapy should be given until progression or intolerance with pembrolizumab in PD-L1 positive patients or with cetuximab according to the EXTREME/TPEx protocol in negative PD-L1 status (CPS<1) if the disease is not progressive.*

- EC
- Strong consensus

**9.13 Consensus-based recommendation 2024**

*In patients with metastatic or non-locally treatable recurrent oropharyngeal and hypopharyngeal carcinoma in good general condition (ECOG PS≤1) who have no immunohistochemical evidence of PD-L1-expressing tumour or immune cells (CPS<1) cetuximab should be used as first-line therapy in combination with platinum (preferably cisplatin) and 5-fluorouracil (EXTREME protocol) or with docetaxel instead of 5-FU (TPEx protocol).*

- EC
- Strong consensus

**9.14 Consensus-based recommendation 2024**

*After platinum-based prior therapy, nivolumab can also be considered as first-line monotherapy (regardless of PD-L1 expression) instead of monotherapy with pembrolizumab, particularly in patients who are not suitable for combined chemo(immune) therapy or in whom there is low remission pressure.*

- EC
- Strong consensus

**9.15 Consensus-based recommendation 2024**

*In patients with metastatic or recurrent oropharyngeal and hypopharyngeal carcinoma with CPS<1 that cannot be treated locally and are unsuitable for combination therapy with cisplatin or 5-FU, combination therapy with docetaxel (or paclitaxel) and cetuximab, or nivolumab monotherapy (after prior platinum-based radio-chemotherapy or platinum-based chemotherapy alone) can be used.*

- EC
- Strong consensus

**9.16 Consensus-based recommendation 2024**

*In patients with metastatic or recurrent oropharyngeal and hypopharyngeal carcinoma that cannot be treated locally in a reduced general condition (ECOG PS≥2) and given contraindications to immunotherapy, monotherapy, for example with docetaxel or cetuximab (off label), should primarily be considered and weighed against symptom-oriented supportive therapy alone.*

- EC
- Strong consensus

Patients with early relapse after cisplatin-based radiochemotherapy can choose between immunotherapy with nivolumab [604] or pembrolizumab or monochemotherapy, for example with docetaxel or paclitaxel. Nivolumab can be administered here regardless of PD-L1 expression (approval in 2017). In patients with recurrence after (more than) 6 months or patients with a first diagnosis of R/M HNSCC without a curative option and CPS≥1 (more detailed explanation of CPS in Section 7.5.2), pembrolizumab is administered as monotherapy or in combination with chemotherapy (platinum and 5-FU). Pembrolizumab monotherapy can be considered for patients with PD-L1 expression of CPS≥1 (further explanation of CPS in Section 7.5.2) if there is no high time-to-remission pressure.

In the Keynote-048 study, the EXTREME protocol with subsequent cetuximab maintenance therapy was randomized against the PD1 inhibitor pembrolizumab alone and against the combination of pembrolizumab, cisplatin and 5-FU with subsequent pembrolizumab maintenance therapy [599]. The subgroup of patients with oropharyngeal and hypopharyngeal carcinomas amounted to approximately 48%. Pembrolizumab alone showed an improved overall survival of 12.3 versus 10.4 months compared to EXTREME in a population with a CPS≥1. Pembrolizumab in combination with chemotherapy improved overall survival by 3 months (13.6 versus 10.6 months) in the population with CPS≥1. Pembrolizumab as monotherapy in patients with high PD-L1 expression (CPS≥20) led to an extension of overall survival from 10.7 to 14.9 months, with a significantly better side effect profile. Since more patients under pembrolizumab monotherapy than under concurrent or sole chemotherapy were primarily progressive, a combination of pembrolizumab with cisplatin and 5-FU is typically administered in cases of high time-to-remission pressure. This protocol corresponds to the current first-line standard and has replaced the previous standard (EXTREME protocol) at the highest level of evidence (evidence level A1) for the group of patients with CPS≥1 [599].

The combination of pembrolizumab, cisplatin and 5-FU achieves a significant extension of overall survival with a comparable remission rate compared to the EXTREME protocol in patients with PD-L1 expression CPS≥1. However, the rate of side effects is comparable to the EXTREME protocol and significantly higher than with pembrolizumab alone. The rate of side effects of the combination of pembrolizumab with platinum/5-FU is comparable to the side effect rate in the EXTREME arm and significantly higher than under monotherapy with pembrolizumab. Fatal treatment-related adverse events with pembrolizumab were reported in 1% (monotherapy) and 4% (in combination with chemotherapy) and in 3% with cetuximab in combination with chemotherapy [599]. Combined chemo-immunotherapy according to the protocol of the KN048 study has also prevailed over the previous EXTREME standard and replaced it at the highest level of evidence (evidence level A1) [607].

With regard to cisplatin-based combined systemic therapy, the combination of platinum with the monoclonal EGFR antibody cetuximab and 5-fluorouracil (with subsequent cetuximab maintenance therapy) had been considered the standard in this situation since 2008 based on data from the randomized phase III EXTREME trial [602], before the above-mentioned pembrolizumab monotherapy or chemotherapy combination was approved in 2019 on the basis of the Keynote 048 trial. The EXTREME triple combination was the first ever to show a significantly increased response rate, progression-free and overall survival (10.1 vs. 7.4 months with an HR of 0.8) compared to platinum in combination with 5-FU and was therefore positive in all effectiveness parameters. Patients’ quality of life improved over the course of treatment, with at most an insignificant increase in toxicity, and tumour-related symptoms were reduced [608]. Maintenance therapy with cetuximab was well tolerated in this phase III study. A predictive biomarker for the selection of patients for this therapy has not yet been identified [603], [609]. Other EGFR antibodies such as panitumumab or the VEGF antibody bevacizumab were unable to achieve the results of the EXTREME trial, meaning that cetuximab is currently the only approved EGFR antibody in combination with platinum-containing chemotherapy in the palliative first-line treatment of recurrent or metastatic squamous cell carcinoma of the head and neck region.

In the randomized phase II study TPExtreme [601], the long-standing standard EXTREME protocol was compared with a combination of cisplatin, docetaxel and cetuximab (TPEx). In 541 randomized patients, it was shown that replacing 5-FU with docetaxel significantly improved tolerability and treatment feasibility without reducing efficacy. With TPEx, chemotherapy was shortened from 6 cycles to 4 cycles and the total cisplatin dose was reduced by 50% compared to the EXTREME protocol. Since the publication of these study results, TPEx has been considered a valid alternative to the EXTREME protocol. The prophylactic administration of G-CSF is recommended as standard. Both protocols are currently still considered the first-line standard for patients with a PD-L1 expression of CPS<1.

Monotherapy should be considered for patients with a reduced general condition. It has been shown that chemotherapy with cisplatin alone leads to a longer survival time compared to treatment with methotrexate, but has a higher toxicity [610]. Tyrosine kinase inhibitors such as gefitinib, erlotinib and afatinib have not been shown to provide significant benefit in the palliative systemic treatment of recurrent or metastatic head and neck squamous cell carcinoma, either as monotherapy or in combination with chemotherapy.

Patients with recurrent or metastatic squamous cell carcinoma of the head and neck should undergo regular cross-sectional imaging every 6–12 weeks during ongoing therapy in order to be able to switch to second-line therapy in good time.

#### 9.2.2 “Second line” drug-based tumour therapy

**9.17 Consensus-based recommendation 2024**

*After failure of primary platinum-containing combination therapy with cetuximab, second-line therapy with a checkpoint inhibitor (pembrolizumab [TPS*≥*50%] or nivolumab as monotherapy) should be carried out.*

- EC
- Strong consensus

**9.18 Consensus-based recommendation 2024**

*After failure of primary platinum-containing combination therapy with pembrolizumab, second-line therapy with docetaxel or paclitaxel, possibly in combination with cetuximab (off label), can be carried out.*

- EC
- Strong consensus

**9.19 Consensus-based recommendation 2024**

*After failure of first-line therapy with pembrolizumab or nivolumab as monotherapy, second-line therapy with platinum/5-FU and cetuximab or with docetaxel or pac* *l* *i* *taxel and cetuximab can be carried out.*

- EC
- Strong consensus

**9.20 Consensus-based recommendation 2024**

*Patients who do not show tumour progression under second-line therapy including cetuximab or pembrolizumab or nivolumab should be offered continuation of therapy with the respective substance used until treatment failure (progression or intolerance).*

- EC
- Strong consensus

For patients with progression after platinum-containing chemotherapy, the PD1 checkpoint inhibitor nivolumab significantly extended survival to 7.5 versus 5.1 months compared to monotherapy with a taxane, methotrexate or cetuximab (HR 0.70 p=0.01) [604]. Nivolumab has been approved independently of PD-L1 status in progression after platinum-containing therapy since 2017.

In an analogous study design, similar results were achieved for the PD1 inhibitor pembrolizumab, with the highest significance being achieved for patients with a PD-L1 TPS≥50% [611]. For patients with a PD-L1 TPS≥50%, the median overall survival of 11.6 months with pembrolizumab versus 6.6 months with taxane, MTX or cetuximab was highly significantly better, so that a comparatively strict approval was only granted for patients with TPS≥50% with progression after platinum-containing prior therapy in 2018.

In this respect, nivolumab (regardless of PD-L1 expression) or pembrolizumab (TPS≥50%) should be used in second-line therapy after platinum-based therapy (EXTREME or TPEx) in the absence of contraindications to a checkpoint inhibitor. After first-line therapy with pembrolizumab mono or platinum-5-FU-pembrolizumab, there is no standard therapy established by studies.

There are several other therapeutic agents in the literature, some of which are not approved in the EU, but are being discussed for use in recurrent/metastatic oropharyngeal and hypopharyngeal carcinomas (NCCN Guidelines Version 2.2023).

**Combination therapies**

- Cisplatin/Cetuximab [612]
- Cisplatin or carboplatin/docetaxel [613] or paclitaxel [614]
- Cisplatin/5-FU [614], [598]
- Cisplatin or carboplatin/docetaxel/cetuximab [601]
- Cisplatin or carboplatin/paclitaxel/cetuximab [615]
- Pembrolizumab/platinum (cisplatin or carboplatin)/docetaxel [599], [613]
- Pembrolizumab/platinum (cisplatin or carboplatin)/paclitaxel [614]

**Monotherapy**

- Cisplatin [612], [616]
- Carboplatin [617]
- Paclitaxel [618]
- Docetaxel [619], [620]
- 5-FU [616]
- Methotrexate [598], [621]
- Cetuximab [622], [623]
- Capecitabine [624]
- Afatinib (subsequent line only for progression after platinum therapy) [625]

### 9.3 Supportive therapy

This chapter is closely based on the extensive explanations of the two S3 guidelines on laryngeal and oral cavity carcinoma [1], [2], which are largely compatible with the treatment of oropharyngeal and hypopharyngeal carcinoma.

In oropharyngeal and hypopharyngeal carcinoma, the various treatment options interact with each other and with many different normal tissues. Potential side effects (e.g. organ loss due to surgery in the case of a laryngopharyngectomy for advanced hypopharyngeal carcinoma, fibrosis after radiotherapy) have an influence on the therapy – in the primary decision, the combinability and the prognosis. Avoidance, treatment and support in the management of therapy consequences have a significant influence on the quality of life of patients with and after oropharyngeal and hypopharyngeal carcinoma. For this reason, side effects are also addressed in various ways in the other chapters of this guideline. This chapter deals with the prophylaxis and treatment of individual side effects of oropharyngeal and hypopharyngeal carcinoma therapy and general supportive measures, which are to be understood in a broader sense as supportive therapy.

A basic distinction must be made between side effects that occur during and immediately after treatment and those that either occur immediately and are long-lasting or occur over a longer period of time after treatment – the latter are particularly common after radiotherapy.

At this point, reference should be made to the S3 Guideline for Supportive Measures in Oncology, which is available in the current version from February 2020 [626]. Many specific side effects are described in detail and provided with recommendations. To prevent overlapping updates, the following side effects of radiotherapy and chemotherapy will not be commented on here, even if they are relevant:

- Anemia/neutropenia
- Nausea/emesis
- Diarrhea
- Oral mucositis due to chemotherapy
- Tumour therapy-induced skin toxicity
- Chemotherapy-induced neurotoxicity (CIPN)
- Supportive therapy in radiation oncology
  - Radiodermatitis
  - Radiogenic osteonecrosis
  - Radiogenic mucositis
  - Radiogenic xerostomia
  - Radiation effects on the brain and spinal cord

The diagnosis of oropharyngeal or hypopharyngeal carcinoma is shocking for most patients and can seriously change their life plans at a stroke. After the shock of the cancer diagnosis and the explanation of the expected therapy, including further postoperative treatments, the patient must come to terms with the information about the possible changes in swallowing function, voice production, breathing, facio-oral functions (smelling, blowing) and stigmatizing changes in the neck area (stoma). It is therefore important to show the patient rehabilitation and support options from the outset through social services, nursing staff, speech therapy, physiotherapy, physical therapy, psychosocial services and early contact with self-help groups. All these measures should be seen as an essential part of supportive therapy, which is not limited to drug treatment of normal tissue reactions that have occurred.

#### 9.3.1 Prevention, side effects and their treatment 

##### 9.3.1.1 Specific supportive measures after surgery

In contrast to complications, the consequences of treatment are typical phenomena that can always be expected after surgery, even if they occur in varying degrees. Surgical treatment of oropharyngeal or hypopharyngeal carcinoma usually leads to functional impairments of varying degrees.

The least functional impairment occurs after transoral surgical techniques (TLM, TORS) for T1 carcinomas of the oropharynx and hypopharynx. The dysphonia or dysphagia that occurs afterwards usually regresses significantly after a few weeks, supported by speech therapy. Larger resections in the oropharynx can lead to permanent functional disorders of the tongue, palate closure, swallowing, voice production and articulation.

As the larynx separates the airway from the alimentary canal, aspiration is one of the frequent side effects after more extensive partial resections of smaller hypopharyngeal carcinomas, as the protective mechanisms of the laryngeal inlet are restricted. This problem does not occur with complete removal of the larynx, as in this situation the airway is separated from the alimentary canal. If a voice prosthesis is inserted, there is a risk of a pharyngo-tracheal fistula forming. Widening of the tracheoesophageal fistula occurs in early and late stages after the insertion of voice prostheses.

In some extensive procedures, shortness of breath due to tissue swelling close to the glottis can be a typical side effect that requires a temporary or even a permanent tracheostomy.

After a laryngopharyngectomy, there is a loss of smell due to the lack of nasal breathing and the risk of recurrent inflammation of the trachea due to the loss of the nose as a filter and humidifier. This can lead to the development of obstructive tracheitis, which requires intensive inpatient treatment. After a laryngectomy, the abdominal press is also not possible, e.g. when lifting heavy loads. Loss of voice after laryngectomy must be regarded as one of the serious side effects, which naturally cannot be avoided primarily due to the removal of the vocal organ. However, various rehabilitation measures are available to develop a replacement voice again. The specific supportive measures following laryngectomy (air humidification, nursing handling of tracheostoma, tracheostomy tubes, resuscitation in patients with tracheostoma) are described in detail in Chapter 7.9.1.1 of the S3 Guideline for Laryngeal Carcinoma [2].

A less frequently observed functional disorder after laryngectomy is stenosis of the hypopharynx, which can occur particularly after extensive resection of the hypopharynx and adjuvant radiotherapy and can then lead to considerable dysphagia and even aphagia.

Many of these typical side effects can be eliminated or reduced by intensive swallowing or voice training. In many cases, it is not possible to treat side effects with medication or surgery. In individual cases, postoperative adhesions of the tongue can be surgically removed and relevant hypopharyngeal stenoses can be eliminated by bougienage or dilatation plasty.

After a neck dissection, swelling may occur in the head and neck area due to congestion and drainage disorders in the lymph vessels. Movement of the head, e.g. turning and tilting, may also be restricted. This can occur mainly after adjuvant radiotherapy and the onset of radiation fibrosis, but also after lesions of the accessorius nerve. Physical and physiotherapeutic treatments can lead to an improvement, although there are no studies that prove the effect of lymphatic drainage. Damage to other nerves (hypoglossal nerve, lingual nerve, vagus nerve, border cord and phrenic nerve) can also occur [2].

##### 9.3.1.2 Side effects and their treatment after radio(chemo)therapy 

During radio(chemo)therapy, the acute side effects listed above (skin reaction, mucositis, xerostomia, radiation effects on nerve tissue) are in the foreground. Other side effects include loss of taste, nausea/vomiting, fatigue and weakness. It is not uncommon for pronounced side effects to lead to the patient wishing to discontinue or pause the therapy, both of which significantly compromise the effect of the therapy. Optimal prophylaxis and therapy are essential components of successful tumour therapy. The supportive measures in radiation oncology are described in detail in Chapter 12 of [626].

There are also chronic side effects that can potentially occur in the long term, but whose probability can be reduced – at least in part – by adequate prophylaxis. This applies in particular to changes to the teeth and jaw, but also to chronic xerostomia and trismus. In addition, there are other chronic side effects of combined therapy such as lymphoedema, functional limitations of the neck and throat muscles and chronically delayed wound healing with a tendency to scarring and fistula formation. A variety of different changes can trigger chronic swallowing disorders, aspiration or hoarseness – examples include progressive fibrosis of the pharyngeal muscles; chronic mucosal oedema in the area of the laryngeal entrance, the ariepiglottic fold and the pocket folds; late oedema in the area of the entire larynx [627]; neuropathies and scarring of the mucosa; fibrosis of the entire oropharyngeal area and the masticatory muscles with consecutive difficulties in opening the mouth; cartilage necrosis in the laryngeal area.

All these side effects require close patient care, ideally by an interdisciplinary team. The sequence of measures is also important – dental restoration must take place before the start of radiotherapy, but the time window between surgery and adjuvant therapy should not be longer than 6 weeks without a compelling reason. If the preoperative presentation to the dentist takes place, tooth extractions can be performed during anaesthesia for tumour resection if necessary. It also helps with adherence if patients receive the same care instructions from all the doctors and other professional groups involved in their care. The benefits of early involvement of nutritional therapy and voice rehabilitation have been described elsewhere. Coordinated management is therefore necessary.

Once chronic changes have occurred, they tend to persist and increase in frequency over the long term, and there is little literature on incidence and treatment. Overall, it is recommended that patients with newly occurring symptoms after oncological therapy, including radiotherapy, should be presented to the radiotherapist in order to establish a connection if necessary and to provide information about treatment options [2].

##### 9.3.1.3 Side effects and their treatment after drug-based tumour therapy

Very often, chemotherapy is administered simultaneously with radiotherapy (primary or adjuvant) and also alone in palliative treatment situations. Substances from immuno-oncology and EGFR targeting are also used (Section 9.2). The most common potential side effects of the substances used (tumour therapy-induced nausea and vomiting, diarrhea, altered hematopoiesis, oral mucositis due to systemic tumour therapy, skin toxicities, nephrotoxicity, chemotherapy-induced peripheral neuropathy, osseous complications, extravasations) are discussed in detail, excluding the specific toxicities of the checkpoint inhibitors approved to date (pembrolizumab, nivolumab) in the above-mentioned S3-LL Supportive Therapy [626].

**Specific toxicities and their therapy of the PD-1 inhibitors pembrolizumab and nivulomab**

Immune-mediated adverse events, including severe cases with sometimes fatal outcomes, have occurred in patients receiving pembrolizumab or nivolumab. Most immune-mediated adverse events that occurred during treatment with PD-1 inhibitors were reversible and manageable by discontinuation of pembrolizumab therapy, administration of corticosteroids and/or supportive measures. Immune-mediated side effects also occurred after administration of the last dose of the PD-1 inhibitor. Immune-mediated side effects can occur simultaneously in more than one organ system. Overview in [628].

If immune-mediated side effects are suspected, appropriate clarification should be ensured to confirm the aetiology or to exclude other causes. Depending on the severity of the side effect, the administration of PD-1 inhibitors should be interrupted and corticosteroids given. If improvement to grade 1 or less is observed, the corticosteroid dose should be reduced and discontinued for at least one month. Based on limited data from clinical trials in patients in whom the immune-mediated side effects could not be controlled with corticosteroids, the administration of other systemic immunosuppressants may be considered. Treatment with PD1 inhibitors should not be continued while the patient is receiving immunosuppressive doses of corticosteroids or other immunosuppressants. Antibiotics should be given prophylactically to prevent opportunistic infections in patients receiving immunosuppressive treatment. Therapy with pembrolizumab/nivolumab can be resumed within 12 weeks of the last dose if the side effect improves to grade 1 or less and the corticosteroid dose has been reduced to ≤10 mg prednisone or equivalent per day. PD-1 inhibitors should be permanently discontinued if another episode of any Grade 3 immune-mediated adverse reaction recurs or if any Grade 4 immune-mediated toxicity occurs, except in the case of endocrinopathies that can be controlled with hormone replacement therapy.

Immune-mediated pneumonitis

Pneumonitis has been reported in patients receiving PD-1 inhibitors. Patients should be monitored for signs and symptoms of pneumonitis. Any suspected pneumonitis should be confirmed by radiologic examination and other causes should be excluded. Corticosteroids should be given for grade ≥2 events (initial dose 1–2 mg/kg/day prednisone or equivalent with subsequent tapering). Treatment with PD-1 inhibitors should be interrupted in grade 2 pneumonitis and permanently discontinued in grade 3, grade 4 or recurrent grade 2 pneumonitis.

Immune-mediated colitis

Colitis has been reported in patients receiving PD-1 inhibitors. Patients should be monitored for signs and symptoms of colitis and other causes should be excluded. Corticosteroids should be given for grade ≥2 events (initial dose 1–2 mg/kg/day prednisone or equivalent with subsequent tapering). Treatment with PD-1 inhibitors should be interrupted in grade 2 or grade 3 colitis and permanently discontinued in grade 4 or repeated grade 3 colitis. The potential risk of gastrointestinal perforation should be considered.

Immune-mediated hepatitis

Hepatitis has been reported in patients receiving PD-1 inhibitors. Patients should be monitored for abnormalities in liver function (at baseline, periodically during treatment and when indicated by clinical evaluation) and for signs of hepatitis, and other causes of hepatitis should be excluded. Corticosteroids should be given at an initial dose of 0.5–1 mg/kg/day prednisone or equivalent for grade 2 events and 1–2 mg/kg/day prednisone or equivalent for grade ≥3 events with subsequent tapering. Depending on the severity of the liver enzyme elevation, treatment with PD-1 inhibitors should be interrupted or permanently discontinued.

Immune-mediated nephritis

Nephritis has been reported in patients receiving PD-1 inhibitors (see Section 4.8). Patients should be monitored for abnormalities in renal function and other causes of renal dysfunction should be excluded. Corticosteroids should be given for grade ≥2 events (initial dose 1–2 mg/kg/day prednisone or equivalent with subsequent tapering). Depending on the severity of the serum creatinine elevation, treatment with PD-1 inhibitors should be interrupted in grade 2 nephritis and permanently discontinued in grade 3 or 4 nephritis.

Immune-mediated endocrinopathies

Severe endocrinopathies, including adrenal insufficiency, hypophysitis, type 1 diabetes mellitus, diabetic ketoacidosis, hypothyroidism and hyperthyroidism have been observed during treatment with PD-1 inhibitors. In cases of immune-mediated endocrinopathies, long-term hormone replacement therapy may be necessary. Adrenal insufficiency (primary and secondary) has been reported in patients treated with PD-1 inhibitors, as has hypophysitis. Patients should be monitored for signs and symptoms of adrenal insufficiency and hypophysitis (including hypofunction of the pituitary gland) and other causes should be excluded. If clinically indicated, corticosteroids should be given to treat adrenal insufficiency and other hormones for replacement. PD-1 inhibitor therapy should be discontinued if grade 2 adrenal insufficiency or hypophysitis occurs until it is controlled with hormone replacement therapy. If grade 3 or 4 adrenal insufficiency or symptomatic hypophysitis, PD-1 inhibitors should be interrupted or permanently discontinued. Resumption of therapy with PD-1 inhibitors can be considered after corticosteroid therapy has been discontinued, if necessary. Pituitary function and hormone levels should be monitored to ensure adequate hormone replacement.

Type 1 diabetes mellitus, including diabetic ketoacidosis, has been reported in patients receiving PD-1 inhibitors. Patients should be monitored for hyperglycemia and other signs and symptoms of diabetes. Insulin should be given for type 1 diabetes. In cases of type 1 diabetes associated with grade ≥3 hyperglycemia or ketoacidosis, therapy with pembrolizumab should be interrupted until the metabolism is under control.

Disorders of thyroid function, including hypothyroidism, hyperthyroidism and thyroiditis, have been reported in patients receiving PD-1 inhibitors and may occur at any time during treatment. Hypothyroidism is reported more frequently in patients with oro-, hypopharyngeal carcinoma and previous radiotherapy. Patients should be monitored for changes in thyroid function (at the start of treatment, periodically during treatment and when indicated by appropriate clinical evaluation) and for clinical signs and symptoms of thyroid disease. Hypothyroidism can be treated with hormone replacement therapy and does not require interruption of therapy or corticosteroid administration. Hyperthyroidism can be treated symptomatically. PD-1 inhibitors should be interrupted in grade ≥3 hyperthyroidism until improvement to grade ≤1. Thyroid function and hormone levels should be monitored to ensure appropriate hormone replacement.

In patients with grade 3 or 4 endocrinopathies that have improved to Grade 2 or less and are under control with hormone replacement therapy if indicated, resumption of PD-1 inhibitor therapy may be considered after tapering off corticosteroid therapy if necessary. Otherwise, treatment should be discontinued.

Immune-mediated side effects on the skin

Immune-mediated severe skin reactions have been reported in patients receiving PD-1 inhibitors (see section 4.8). Patients should be monitored for suspected severe skin reactions, and other causes of severe skin reactions should be excluded. Depending on the severity of the adverse reaction, the administration of PD-1 inhibitors should be interrupted in grade 3 skin reactions until improvement to grade ≤1 or permanently discontinued in grade 4 skin reactions and corticosteroids should be administered.

Cases of Stevens-Johnson syndrome (SJS) and toxic epidermal necrolysis (TEN) have been reported in patients receiving PD-1 inhibitors. If SJS or TEN is suspected, administration of PD-1 inhibitors should be discontinued and the patient should be referred to an appropriate medical specialist for evaluation and treatment. If SJS or TEN is confirmed, PD-1 inhibitors should be permanently discontinued. Caution should be exercised when considering the use of PD-1 inhibitors in a patient who has previously experienced a serious or life-threatening skin side effect with other immunostimulatory drugs used to treat cancer.

Other immune-mediated adverse reactions

The following other clinically relevant immune-mediated adverse reactions have been reported in clinical trials or after the market launch of PD-1 inhibitors: uveitis, arthritis, myositis, myocarditis, pancreatitis, Guillain-Barré syndrome, myasthenic syndrome, hemolytic anaemia, sarcoidosis, encephalitis, myelitis, vasculitis, sclerosing cholangitis, gastritis, non-infectious cystitis and hypoparathyroidism.

Depending on the severity and type of side effect, therapy with PD-1 inhibitors should be interrupted for grade 2 or 3 events and corticosteroids should be administered.

PD-1 inhibitor therapy may be resumed within 12 weeks of the last dose if the adverse event improves to Grade 1 or less and the corticosteroid dose has been reduced to ≤10 mg prednisone or its equivalent per day.

Treatment with PD-1 inhibitors should be permanently discontinued if any grade 3 immune-mediated adverse reaction recurs and for any grade 4 immune-mediated adverse reaction.

For grade 3 or 4 myocarditis, encephalitis or Guillain-Barré syndrome, PD-1 inhibitors should be permanently discontinued.

Detailed information on PD1 inhibitors pembrolizumab and nivolumab is available on the website of the European Medicines Agency (https://www.ema.europa.eu) [629], [630]. Further information can also be found in the specific ESMO guideline [631].

##### 9.3.1.4 Supportive therapy teeth and gnatological system

In the treatment of oropharyngeal or hypopharyngeal carcinoma, the preservation and restoration of a functional stomatognathic system (teeth, oral cavity, jaw) plays an important role in maintaining quality of life. When preparing a patient for surgery or radiotherapy, the oral cavity should be germ-reduced by professional dental removal of soft and hard dental plaque. In the case of planned radiotherapy of the oropharyngeal or hypopharyngeal region (depending on the extent of the tumour and the affected lymphatic drainage pathways), different radiation exposure to the lower jaw and teeth is to be expected. This results in the classification of these patients into the “risk groups” according to Studer et al. [632]. The recommended prophylactic measures for the stomatognathic system can be found at [626], [633], [634].

For further information on specific dental supportive measures, please refer to Section 7.9.1.4 of the S3 Guideline for Laryngeal Guideline [2].

**9.21 Evidence-based recommendation 2024**

*Patients who have undergone surgery and/or radiotherapy for oropharyngeal or hypopharyngeal carcinoma should have their chewing ability restored through masticatory rehabilitation with implants or conventional prosthetic restorations. Furthermore, these patients should be regularly monitored by a dentist. Dental surgery should be performed on these patients by specialists experienced in this clinical picture.*

- GoR: B
- LoE: 3
- [1], [635], [636], [637], [638], [639], [640]
- 3: S3 guideline adaptation – Oral Cavity Carcinoma, Version 3.0 202 (9.2)
- Strong consensus

**9.22 Consensus-based recommendation 2024**

*If radio- or radiochemotherapy is planned for oropharyngeal or hypopharyngeal carcinoma (primary or adjuvant), a dental check-up should be carried out before the start of treatment. The patient should be informed about prophylactic measures.*

- EC
- Strong consensus

## 10 Rehabilitation, psychosocial care and supportive therapy

**10.1 Consensus-based recommendation 2024**

*For the best possible functional outcome,* ***pre-, peri- and/or post-therapeutic*** *rehabilitative measures should be part of the treatment concept.*

- EC
- Strong consensus

**10.2 Consensus-based recommendation 2024**

*Before and/or during primary therapy, information should be provided about the possibility of contacting self-help groups.*

- EC
- Strong consensus

### 10.1 Swallowing rehabilitation

**10.3 Consensus-based recommendation 202**4

*In the case of a pre-operative swallowing disorder, dysphagia diagnostics and, if necessary, swallowing training should be carried out pre-operatively or pre-therapeutically.*

*Swallowing function should be examined as early as possible post-operatively or post-therapeutically. The aim is rapid oral food intake and, if necessary, swallowing training, depending on the healing process and the therapy methods used.*

- EC
- Strong consensus

In patients with oropharyngeal and hypopharyngeal carcinoma, swallowing function may already be impaired pre-therapeutically. Therapy often results in dysphagia or inability to swallow, which can be alleviated or overcome with timely and appropriate therapy. For this reason, appropriate diagnostics and advice should be provided by doctors and therapists on the various rehabilitation measures in accordance with the patient’s medical history and clinic, explaining the planned procedure and the resulting rehabilitation options (see also consideration of prehabilitation in Section 10.3.4). The therapists are available to answer patients’ questions about swallowing rehabilitation. The early involvement of self-help groups can be useful here.

In everyday clinical practice, FEES (fiberoptic endoscopic evaluation of swallowing) performed by doctors plays a central role in diagnosis and therapy monitoring. Swallowing therapy in the inpatient setting begins as early as possible after surgery. In consultation with the doctors, if there are no complications, treatment can begin to promote the elasticity of the throat, neck, face and speech muscles [641]. To optimize the therapy results, adequate pain and nutrition therapy should be provided at an early stage if necessary [642]. If dysphagia therapy is indicated, it should be carried out promptly, on an outpatient basis or as part of inpatient follow-up treatment [643]. Sensory disorders or (usually unilateral) tongue paralysis can occur in the oral cavity following damage to the hypoglossal nerve caused by neck dissection. Promoting the elasticity of the throat muscles, swallowing aids and dietary adaptation of consistencies can be helpful [642], [644]. Due to the separation of the airway and food pathway, the dietary reconstruction after laryngopharyngectomy is usually uncomplicated, provided no stenosis has occurred. Aspirations can occur due to inadequate control of swallowing in the event of sensory disturbances in the hypopharynx and laryngeal inlet, substance deficits in the laryngeal inlet area and a leaky shunt valve after laryngectomy. Swallowing disorders in the sense of passage disorders with retention (food residue) in the pharynx occur with mechanical (e.g. restriction of tongue movement) and motor deficits in the area of the oral cavity and pharynx or may only develop after a few weeks or after radiation due to stenosis. The most common causes are mucosal swelling, scarring or recurrences, which must be clarified diagnostically. Partial resections of the hypopharynx/larynx are generally function-preserving measures, but can lead to functional impairments depending on the surgical procedure (hemipharyngolaryngectomy or horizontal partial resection for supraglottic infiltrating tongue base carcinomas) [645], [646].

Breathing/swallowing coordination can be severely impaired after a partial hypopharyngeal resection. In contrast to a total laryngectomy, the airway and alimentary canal are not separated. Swallowing disorders, aspirations and the wearing of a blocked tracheostomy tube, which makes voiced speech impossible, are the result [647], [648]. In addition, the regular suctioning of aspirated saliva often has a major impact on quality of life for a long time. Swallowing rehabilitation and tracheal cannula management therefore often take priority over voice rehabilitation.

Swallowing should be trained as early as possible. The therapy includes elements of elasticity promotion to improve mobility and coordination of the processes in the respiratory-swallowing tract [647] as well as compensatory procedures (such as head posture changes during swallowing, swallowing maneuvers [649]. Various studies on head and neck tumours in general show a decrease in dysphagia when therapy is started early [650] and poorer therapy results when therapy is started later [651]. Kulbersh et al. [652] confirm clinical observations according to which the swallowing results after radiotherapy were significantly better if speech therapy was started 14 days before radiotherapy.

In order for the patient to withstand the stress of therapy, adequate pain and nutrition therapy is necessary at an early stage [642]. If possible, tube feeding should not be the patient’s sole form of nutrition. Gillespie et al. [650] showed that swallowing ability deteriorated after 14 days following tube feeding alone without oral nutrition. During regular oral intake, even of small amounts, all structures involved in the swallowing process are moved and kept elastic several times a day.

If removal of the tracheal cannula (decannulation) is not possible during the inpatient stay due to aspiration and the inability to swallow, the therapy should be continued in inpatient rehabilitation or on an outpatient basis. The staff of the outpatient nursing service should be experienced in handling cannulas. Any necessary functional voice rehabilitation should also be carried out as part of inpatient rehabilitation or on an outpatient basis.

### 10.2 Speech and voice rehabilitation

**10.4 Consensus-based recommendation 2024**

*Even before the start of tumour therapy, the later speech and voice function should be taken into account.*

*Patients should be informed about the various re* *h* *a* *b* *i* *l* *i* *t* *a* *tion options with the involvement of voice thera* *p* *i* *sts and patient advisors from self-help groups.*

- EC
- Strong consensus

**10.5 Consensus-based recommendation 2024**

*When deciding which procedure to use for speech and voice rehabilitation after treatment of oropharyngeal or hypopharyngeal cancer, the expected anatomical conditions after treatment, the findings of the voice and articulation diagnostics and the patient’s preference should be taken into account.*

- EC
- Strong consensus

Motor function restrictions of the soft palate and tongue, as well as sensitivity disorders, can lead to pronunciation disorders, which are often very stressful for the patient [653]. Targeted speech therapy (dysglossia therapy) should then be carried out. Patients who have not undergone direct laryngeal surgery but have been treated with radiotherapy or radiochemotherapy also often develop a voice disorder and can benefit from voice therapy [654]. Voice rehabilitation after laryngectomy and partial laryngectomy for hypopharyngeal carcinoma is discussed in detail in section 7.10.2 of the S3 guideline on laryngeal carcinoma, so please refer to this supplementary guideline for further information [2].

### 10.3 Psychosocial rehabilitation

Although cancer is often diagnosed in the older population and the average age of oncology in Germany is 70 years for men and 69 years for women, a large number of patients of working age also develop malignancies [655]. In Germany, around 45% of males and 57% of females out of a total of 476,120 oncology patients are younger than 65 [656]. Depending on the type and location of the tumour and the country of origin, between 24% and 94% of patients return to work after malignant tumour treatment [657], [658], [659], [660]. Returning to society and working life is important for one’s own identity, self-esteem, social integration and economic status. But it is also economically relevant, as around 60% of the costs incurred are mainly due to the absence of the patient and family carers from work [661], [662], [663]. The importance of reintegration into social and professional life will increase in the future, and with it the importance of psychosocial rehabilitation, due to the optimization of treatment procedures and the associated higher cure rates, younger patients with HPV-associated oropharyngeal carcinomas in particular (significantly fewer comorbidities), as well as the longer working life in Germany due to the postponement of the retirement age to 67.

For patients with squamous cell carcinoma of the head and neck, there is as yet little knowledge regarding the effects of the disease and therapy on professional reintegration [660], [664]. In contrast, the post-therapeutic occupational status and its influencing factors for other common malignant neoplasms, such as breast and colorectal cancer, are much better recorded. Studies show that the number of people who return to work after a head and neck tumour is lower than for other tumour entities [664], [665], [666], [667]. In 2013, tumour diseases of the mouth and throat in Germany led to 368,078 days of incapacity for work and 1,466 new pensioners due to reduced earning capacity; laryngeal carcinomas led to 110,446 days of incapacity for work and 333 new pensioners due to reduced earning capacity [655].

For patients of working age, it can be seen that returning to work is becoming increasingly important after treatment for oropharyngeal and hypopharyngeal cancer [657], [660], [668]. For this reason, it is an important goal of the social and socio-legal counselling services to secure material and economic existence as well as participation in working life and to enable access to social benefits. The psychosocial counselling offered by the social services should be low-threshold, i.e. patients in an inpatient context should receive initial oncological counselling from the social workers at acute hospitals and rehabilitation facilities. This can ensure that patients and their relatives receive basic psychosocial information, such as information on social law, advice on medical and occupational rehabilitation options, assistance with applications, psychosocial counselling in conflict situations or for coping with the disease and, if necessary, initial interventions are possible.

Outpatient cancer counselling centres also offer low-threshold psychosocial counselling on psychological, social and social law issues for the phase after inpatient treatment and provide information on self-help groups and other counselling services.

#### 10.3.1 Vocational rehabilitation

**10.6 Consensus-based recommendation 2024**

*Occupational rehabilitation following treatment for oropharyngeal or hypopharyngeal carcinoma is a particular challenge due to the functional restrictions and should be taken into account in the decision-making process before treatment begins.*

- EC
- Strong consensus

A systematic literature search on occupational rehabilitation after laryngectomy in laryngeal and hypopharyngeal cancer patients [668] revealed that re-employment rates after surgery vary greatly depending on the country in which the patient lives and when the study was conducted: Spain, 1990s, 11% [669], USA, 1970s, 26% [670], Soviet Union, 1980s, 27% [671], France, 1980s, 50% [672], Soviet Union, 1960s, 51% [673] and Norway, 1970s, 63% [674] of all patients. If only those who were employed before the operation are considered, the reintegration rates were 20% in Germany in the 1980s [675], 32% in Germany in the 1960s [676] and 41.5% in the USA during the 1970s [670].

There is little corresponding data available for Germany. In the Central German Laryngectomy Study [668], 38% of patients ≤60 years of age before laryngectomy were employed, one year after laryngectomy only 13%, two years 15% and 3 years 14%. Most patients (65%) received a disability pension. Of all patients who were employed before the laryngectomy, 27% still had a job after treatment.

In another register-based study in the Free State of Thuringia, three quarters of the head and neck tumour patients surveyed (at least 2 years after diagnosis, ≤60 years old) stated that they had been employed before the diagnosis; at the time of the survey, only one third were still employed [677]. A meaningful analysis of the few international publications by Zebralla et al. on the return to work of head and neck cancer patients [664] showed that, depending on the study, between 10% and 67% of pre-therapeutic working patients did not return to work after curative therapy, with younger patients returning to work more frequently than older patients. Between 4.1% and 40% of patients took early retirement or retired. The main reasons for not returning to work were physical symptoms such as fatigue, weakness, pain, dysphonia, oral dysfunction (trismus, xerostomia, difficulty eating) and loss of appetite. In addition, there were psychological limitations that manifested themselves in depression, memory and sleep disorders. Alcohol abuse, low level of education, lower income, few or no social contacts and single marital status were also associated with unemployment or early retirement. Up to 36% of patients changed jobs after therapy. The main reasons for this were functional complaints caused by the carcinoma or as a result of its treatment. In addition, some studies showed a significant reduction in weekly working hours compared to working hours before the tumour disease. Up to 52% of working patients worked significantly fewer hours after their tumour disease. These factors lead to a reduction in the household income of those affected, which was reported by up to 56% of patients (overview [664]).

The median time for patients to return to work after treatment ranged from 2 to 9 months in the various studies. The majority of patients who returned to work (up to 71%) did so within 6 months of the end of treatment. However, a relevant proportion (15% of patients) also returned to work more than 12 months after completing treatment. The self-employed returned to work more quickly than the non-self-employed. There was also a difference between blue collar workers and white collar workers. This was shown by Handschel et al. [678], among others, in a German collective of 755 employees. In this collective, 52% of patients did not return to work after diagnosis and treatment of head and neck cancer. Here, 63% of blue-collar workers did not return to work after tumour therapy, compared to only 41% of white-collar workers. Of those who returned to work after therapy, 63% of white-collar workers, but only 37% of blue-collar workers returned to the same job. Overall, white-collar workers returned to work earlier, with 32% of white-collar workers and 12% of blue-collar workers returning to work after 3 months. Blue-collar workers also reduced their working hours more frequently than white-collar workers (overview [664]).

With regard to gender, there were divergent and sometimes contradictory results in the individual studies, so that it cannot be conclusively determined whether men or women with head and neck cancer return to work earlier or more frequently. Depending on the respective study, different tumour localizations were indicated as prognostically favourable for a return to work. In the analysis by Vartanian et al. [679], patients with laryngeal carcinomas had the highest rate of return to work, followed by patients with oral cavity carcinomas. In contrast, in the study by Buckwalter et al. [680], patients with oropharyngeal carcinomas returned to work most frequently, while patients with oral cavity carcinomas also returned to work at a lower rate. In the study by Verdonck et al., patients who had undergone treatment for oral cavity and oropharyngeal cancers had a higher rate of return to work than those with nasopharyngeal cancers [681]. Most studies that investigated the influence of tumour stage on working life after the end of treatment found a negative association of these parameters with subsequent employment (overview [664]). Comorbidity (Charlson Comorbidity Score ≥3) was also a negative predictor of return to work.

While Pearce et al. [682] showed that patients who did not receive chemotherapy as part of treatment were more likely to take no time off at all and return to work more quickly than patients who had undergone chemotherapy, and Buckwalter et al. [680] showed a negative association between multimodal therapy and return to work, other studies investigating the relationship between treatment and return to work found no significant differences between treatment modality and post-therapy employment rate (overview [664]). Patients who did not return to work at all or who changed jobs were significantly more likely to be dissatisfied with their own appearance after treatment for their head and neck tumour than patients who returned to their original job after treatment [680]. The global quality of life of patients who returned to work after treatment was also better [677], [682], [683], [684]. In a recent meta-analysis in 2022, Yu et al. showed that head and neck cancer patients who returned to work had lower levels of anxiety and depressive symptoms on the Hospital Anxiety and Depression Scale [683].

According to Zebralla et al. [664], the following predictive factors for the professional reintegration of patients with head and neck tumours, including oropharyngeal and hypopharyngeal carcinomas, are summarized:

**Positive predictors**

- Employees/civil servants
- High professional qualification
- Satisfactory aesthetic result postoperatively
- Voice prosthesis for laryngectomy

**Unclear predictors**

- Gender
- Tumour location
- Treatment modality

**Negative predictors**

- Charlson Comorbidity Score ≥3
- Advanced tumour stage
- Alcohol abuse
- Low level of education
- “Single” marital status
- Lack of social contacts
- Depression
- Functional limitations and physical symptoms (including pain, dysphonia, dysphagia)

Overall, there has been growing interest in employment after curative treatment of patients with oropharyngeal carcinoma in particular over the last 20 years, with an increasing number of publications. However, the limited comparability of the available studies must be taken into account, as the analysis dates were different and not all patients were gainfully employed before the start of treatment. In addition, the studies showed heterogeneous patient groups with different tumour locations, stages and therapies, which most likely do not affect employment to the same extent. Compared to patients with tumours of other entities, head and neck tumour patients primarily have to struggle with treatment side effects that are visible to the social and professional environment directly or via social interaction or their dysfunctionality caused by reduced organ function. Mehnert et al. [659] were able to show that almost half of breast cancer patients returned to their original job immediately after rehabilitation, compared to only around a third of head and neck cancer patients. This may be due to the advanced stage of the tumour at the time of diagnosis and a reduced physical condition [659].

In summary, up to 50% of pre-therapeutic head and neck cancer patients do not return to work after tumour therapy, and job changes and reductions in working hours are also common. Positive effects have been shown for rehabilitation measures and reintegration programs. For this reason, it is an important goal of the social and socio-legal counselling services to secure material and economic existence as well as participation in working life and to enable access to social benefits. The psychosocial counselling offered by the social services should be low-threshold, i.e. patients with oropharyngeal and hypopharyngeal carcinoma should receive initial oncological counselling from the social workers at acute hospitals and rehabilitation facilities in an inpatient context. This ensures that those affected and their relatives receive basic psychosocial information, e.g. information on social law, advice on medical and occupational rehabilitation options, assistance with applications, psychosocial counselling in conflict situations or for coping with the disease and, if necessary, initial interventions are possible.

Outpatient cancer counselling centres also offer low-threshold psychosocial counselling on psychological, social and socio-legal issues for the phase after inpatient treatment and provide information on self-help groups and other counselling services. In recent literature, the importance of occupational therapy in the occupational rehabilitation of head and neck tumour patients is particularly emphasized. Occupational therapists play an important role in daily coping as part of reintegration through lifestyle management and the use of positive coping strategies for daily routine management. Occupational therapy can positively impact the debilitating stress and anxiety associated with head and neck cancer diagnosis, treatment and recovery, while facilitating a return to previous or adapted daily routines [684], [685].

#### 10.3.2 Psycho-oncological care

**10.7 Consensus-based recommendation 2024**

*Immediate and long-term needs-oriented psycho-oncological care should be ensured.*

- EC
- Strong consensus

Around 30% of all patients with head and neck tumours suffer from severe psychological stress [686], [687], [688], [689], [690], [691] and their relatives also frequently suffer from anxiety and depression as well [691].

Often these psychological stresses are not actively communicated by the patients, so that they are not noticed by the treating physician and therefore remain untreated [692], [693].

Head and neck cancer patients’ mental health deteriorates more frequently over time than other cancer patients [694], which is probably due to the fact that they are less likely to ask for social support and therefore receive less support. It is therefore particularly important here that the doctor and the treating team actively and repeatedly enquire about the patient’s psychological stress [695]. This can be done in personal discussions and/or with the help of computer-based routine screening during follow-up care [696].

Psycho-oncological care has been shown to help improve the psychological well-being of cancer patients and increase their quality of life [697]. All patients with increased psychological stress should therefore be offered such care, for example with direct referral to a psycho-oncologist. Psycho-oncological care should not only be offered during the patient’s inpatient treatment, but should also be considered during aftercare. It should be based on the S3 Guideline: “Psycho-oncological diagnostics, counselling and therapy” [685].

The treatment phase and the disease- and therapy-related effects on physical, psychological and social functioning often lead to dramatic changes in the social lives of people with cancer and their caregivers.

The stresses and strains include

- Family and social stresses (e.g. partnership conflicts, conflicts in dealing with the disease and the consequences of the disease, care needs of the affected person or even uncared for relatives, loss of social contacts in the circle of friends or leisure associations)
- Occupational changes (problems at work, limited and inadequate professional performance up to the loss of earning capacity and job, difficulties in finding a new job)
- Financial burdens (e.g. due to reduced income, additional payments for health services, travel costs)
- In connection with medical rehabilitation options, it is important to emphasize the offers of medical-occupational orientation in rehabilitation for the targeted, interdisciplinary promotion of occupational integration. It enables patients to test the skills they need for their profession and to train any deficits in a targeted manner [698].

#### 10.3.3 Social law support

**10.8 Consensus-based recommendation 2024**

*Patients and relatives should already be informed about possible psychosocial consequences and, if necessary, help during primary therapy.*

- EC
- Strong consensus

The treatment phase and the disease- and therapy-related effects on physical, psychological and social functioning often lead to dramatic changes in the social lives of people with cancer and their caregivers.

These non-medical aspects and stresses should also be taken into account during the initial process of providing information about the tumour disease, the treatment options and the course of the disease, as described above. In particular, this includes the doctor pointing out the additional contacts in the various areas and the wide range of support options.

#### 10.3.4 Medical rehabilitation

**10.9 Consensus-based recommendation 2024**

*Patients with oropharyngeal/hypopharyngeal carcinoma should be informed that they are entitled under social law to follow-up treatment (FUT) and subsequently to medical rehabilitation.*

*FUT and rehabilitation should be recommended by the doctor.*

- EC
- Consensus

**10.10 Consensus-based recommendation 2024**

*Patients with oropharyngeal and hypopharyngeal cancer should be rehabilitated in appropriately specialized facilities.*

- EC
- Strong consensus

**10.11 Consensus-based recommendation 2024**

*For the best possible functional outcome, pre-, peri- and post-therapeutic rehabilitative measures should be part of the treatment concept.*

- EC
- Strong consensus

Depending on the need for rehabilitation and the patient’s ability and willingness to undergo rehabilitation, primary therapy should be followed by inpatient rehabilitation in the form of follow-up treatment (FUT). The consequences of a mostly multimodal therapy consisting of surgery, radiation and chemotherapy and other drug therapy procedures are to be treated. The aim of medical rehabilitation is to promote the patient’s self-determined participation in the sense of an independent and responsible social life despite health and functional limitations and to enable reintegration into working life.

Follow-up treatment is prescribed by the attending physicians as part of the inpatient stay or as part of outpatient tumour aftercare. The social services of the acute hospitals provide advice and initiate rehabilitation.

Patients with oropharyngeal or hypopharyngeal cancer who have received radiotherapy/radiochemotherapy should start rehabilitation at the earliest six weeks after the end of radiotherapy. Although the acute skin changes diminish shortly after treatment, lymphoedema increasingly develops in the skin and mucous membrane of the head and neck area. It is fully developed after six weeks and must be treated with lymphatic drainage. As a result, the period for commencing FUT after radiotherapy/radiochemotherapy is extended to up to 10 weeks. In individual cases, FUT can be started earlier as a complex medical inpatient measure with swallowing tests and training), nutritional stabilization, lymph drainage, exercise therapy and adapted medication and pain therapy.

Depending on the extent of the symptoms and functional impairments, the patient has the option of repeating the inpatient rehabilitation measure.

##### Prehabilitation

In recent years, the term prehabilitation has found its way into the literature and is arising also in cancer therapy. Prehabilitation is the targeted preparation for an operation or a debilitating therapy. While classic rehabilitation supports patients in their recovery following a stay in hospital, prehabilitation is intended to have a positive influence on this in advance. Ideally, prehabilitation combines various components that are individually tailored to the patient: primarily physiotherapy, muscle and breathing training, but also weight loss and nutritional therapy. Initial studies have shown the positive effects in endoprosthetics, for example. In patients who received a new hip or knee joint, pain was halved after four weeks of training. Prehabilitation is still in its infancy in Germany and is only widespread for hip and knee replacements.

In the context of oncological therapy, current studies show clear evidence of the positive effects of more intensive preparation for surgery in patients with frailty and malnutrition. Both factors [699], [700], have a significant influence on peri- and post-therapeutic morbidity and oncological functional outcome. More than a third of hospitalized patients suffer from malnutrition, far more frequently than long assumed [701]. The aim of oncological prehabilitation is therefore to identify malnutrition, frailty and anaemia in particular and to improve them before the start of therapy [702].

The increased therapy-related morbidity risk of increasingly elderly and multimorbid head and neck tumour patients is well known [144] and can be recorded as a frailty score. The topic is currently also finding its way into the clinical consideration of head and neck cancer, so that the S3 guidelines on oral cavity and laryngeal carcinoma do not yet contain any clear recommendations on targeted pre-therapeutic preparation, apart from the advice to take early measures to ensure adequate nutrition [1], [2]. Recommendations largely focus on improving therapies (e.g. minimally invasive and reconstructive surgery, intensity-modulated radiotherapy, studies on therapy de-escalation) and on classic rehabilitation measures. In Germany, professional swallowing therapy is usually only carried out when symptoms occur, as part of a specific rehabilitation measure, usually postoperatively or following radio(chemo)therapy [643], [703], [704], [705].

Head and neck tumour patients are particularly susceptible to malnutrition due to the location of the tumours in the upper aerodigestive tract and the resulting dysphagia, as well as the risk factors of tobacco and alcohol. Possible serious consequences of dysphagia include malnutrition, the risk of aspiration pneumonia and the associated increased mortality, social isolation and loss of quality of life [642], [706]. The prevalence of dysphagia among head and neck tumour patients is up to 80%, depending on tumour location and extent [707].

Study results increasingly indicate that head and neck tumour patients benefit if dysphagia therapy begins before or during radiotherapy [708]. Preventive exercises can reduce the consequences of pre-existing dysphagia or dysphagia that occurs as a result of therapy [709]. In summary, the studies available to date show that active exercises to improve swallowing function immediately after radio(chemo)therapy lead to demonstrably better outcomes, even if evidence of long-term benefit is still lacking. Collectives with primarily surgically treated patients have hardly been studied to date with regard to the effectiveness of prehabilitative measures, nor have suitable criteria for patient selection been developed. The first clinical implications are shown by the systematic introduction of frailty scores [144] and the associated growing awareness of individualized therapy preparation. This subtext is intended to raise awareness of the topic. Specific recommendations cannot yet be made within the framework of this guideline.

### 10.4 Nutrition

The diet must be adapted to the treatment procedure and the resulting function of the swallowing act. It must also be considered that sensitivity disorders in the oral cavity following damage to the lingual nerve or mucositis following radiotherapy can impair food intake. Compensatory swallowing techniques and dietary adjustment of consistencies can be helpful [642].

Aspiration of saliva, liquids and food are typical consequences in the first few weeks after immediate larynx-preserving surgery/radiation of the hypopharynx or surgery near the larynx/radiation in the base of the tongue [646]. It takes time for compensatory replacement strategies to develop to create new closures. For example, after partial laryngectomy, unilateral hyperplasia of the base of the tongue and the contralateral arytenoid cartilage can develop after a few weeks and form the new closure. Initial postoperative swelling may also subside after a few weeks. During this time, an artificial feeding tube, nasogastric tube or PEG is indicated. If things go well, oral training can then be started, possibly with drops of water and small amounts of mashed potato.

#### 10.4.1 PEG

**10.12 Evidence-based recommendation 2024**

*Patients who are at risk of malnutrition due to tumours or treatment should receive professional nutritional advice and nutritional therapy at an early stage.*

- GoR: B
- LoE: 2+
- [1], [710], [711], [712], [713], [714], [715]
- 2+: S3 guideline adaptation – Oral Cavity Carcinoma, Version 3.0 2021 (9.7)
- Strong consensus

**10.13 Consensus-based statement 2024**

*In patients with oropharyngeal and hypopharyngeal carcinomas with significant dysphagia, additional enteral tube feeding should be carried out if oral food intake is insufficient.*

- EC
- Strong consensus

**10.14 Consensus-based recommendation 2024**

*The prophylactic insertion of a PEG before primary or adjuvant radiochemotherapy should not be carried out in the absence of swallowing difficulties or, in the case of swallowing difficulties, without primary swallowing diagnostics.*

- EC
- Strong consensus

**10.15 Consensus-based statement 2024**

*If tube feeding (transnasal or transcutaneous) is necessary, a PEG should be preferred to a nasogastric tube if dysphagia is present or expected to persist for a long time.*

- EC
- Strong consensus

Oro- and hypopharyngeal carcinomas can lead to swallowing difficulties with significant weight loss and aspiration with consecutive pneumonia even before the diagnosis is made. Surgical therapies and adjuvant radiotherapy or radiochemotherapy cause relevant swallowing problems with the risk of serious malnutrition and/or aspiration pneumonia, just like primary radiotherapy or radiochemotherapy. Early nutritional support must therefore be ensured for all patients with oropharyngeal and hypopharyngeal carcinomas. Repeated nutritional counselling with dietary modifications contribute to the success of treatment and quality of life in patients with head and neck cancer who have difficulty swallowing. Risk factors for prolonged postoperative dysphagia include pharyngotomy, resection at the base of the tongue, reconstruction with a pectoralis major flap, advanced tumour growth, alcohol abuse and radiation and radiation chemotherapy [711].

In uncomplicated cases, nutrition can be provided with soft or liquid high-calorie food, possibly supported by local and systemic analgesic therapies, provided the patient does not aspirate. If these measures are not sufficient, nutrition must be provided via a PEG or nasogastric tube or parenterally. If it is expected that oral feeding will no longer be possible for at least several weeks, the PEG tube has proven to be safe and effective [712], [713], [714]. A retrospective comparison of a PEG and a nasogastric feeding tube showed greater impairment of swallowing and speech and poorer wearing comfort with the nasogastric feeding tube [715]. In a small randomized study (n=57), early swallowing training led to a significant reduction in the duration of PEG tube placement [716].

The benefit of prophylactic placement of a PEG tube in patients at high risk of severe swallowing problems compared to placement on demand was investigated in 3 small randomized trials (n=70–134), most of which had received primary or adjuvant radiochemotherapy [717], [718], [719]. The studies consistently showed no significant benefit of prophylactic PEG placement in terms of weight development, quality of life and oncological outcomes. The complication rate of PEG placement was low in all studies and did not differ between the study arms. A need for tube feeding was found in 86–90% of patients in the control arms of the studies. The longer dependency on tube feeding reported in retrospective comparative studies [715] due to prophylactic PEG placement was not confirmed in the randomized studies.

### 10.5 Palliative care

Palliative care has now been described in detail in a current S3 guideline, backed up with the latest evidence and has also been comprehensively agreed to for patients with oropharyngeal and hypopharyngeal carcinomas [720]. With reference to this guideline, only the principles of palliative care are listed below.

The basic aim of palliative care is to improve or maintain the quality of life of patients and their families by alleviating and preventing suffering, despite incurable illness. Since the beginnings of the palliative and hospice movement, this has been associated with a basic attitude of all those involved in treatment, characterized by the holistic perception of patients and their relatives as persons in (family) systems as well as the acceptance of dying and death as part of life [721]. The living environment of those affected is perceived holistically in four dimensions – physical, psychological, social and spiritual. In practical implementation, this is based on the principles set out in the S3 Guideline for Palliative Medicine for patients, relatives and members of the multi-professional treatment team. The following rules should be applied in the palliative care of patients with incurable cancer:

- The consideration of and responsiveness to the patient’s needs in all four dimensions (physical, psychological, social, spiritual);
- The consideration of patient preferences;
- Recognizing the patient’s cultural, ideological and religious identity;
- Determining realistic treatment goals together with the patient and relatives;
- Knowledge of organizational forms of palliative and hospice care;
- Creating local conditions that respect the intimacy of the patient and their family;
- Carrying out an appropriate differential diagnosis of the causes of the symptom for targeted therapy and identifying potentially reversible causes;
- The use of preventive measures and the treatment of reversible causes, if possible and appropriate;
- The implementation of symptomatic therapy – alone or in parallel with causal therapy;
- The balancing of tumour-specific measures (e.g. radiotherapy, surgical procedures, drug-based tumour therapies) with the primary or sole therapeutic goal of symptom relief. Interdisciplinary cooperation between the respective specialist areas and palliative medicine is a prerequisite.

Patients with oropharyngeal or hypopharyngeal carcinoma require special attention, even at the end of life, to maintain their ability to communicate and overcome the social isolation that is often observed.

The patient’s wishes must be respected at every stage of treatment, including the dying phase. If the patient is unable to express themselves, the patient’s representative (person authorized by a written power of attorney or court-appointed guardian) must determine the patient’s wishes and discuss this with the doctor. A written living will and other expressions of the patient’s wishes (e.g. treatment wishes expressed verbally or in writing, other expressions of wishes) must be included in this process [720].

## 11 Aftercare

**11.1 Consensus-based recommendation 2024**

*The maximum follow-up intervals should be 3 months for the 1* *^st^* *and 2* *^nd^* *year and 6 months for the 3* *^rd^* *to 5* *^th^* *year, even if there are no symptoms. A risk-adapted specialist aftercare plan should be drawn up for each patient. Quality of life and pain should be assessed regularly. After the 5* *^th^* *year, regular specialist check-ups should be offered.*

*Adoption recommendation 9.1: S3 Guideline on Oral Cavity Carcinoma *[1]

- EC
- Consensus

### 11.1 Clinical anamnestic examination

Regular tumour follow-up care is an essential part of the overall treatment, which should be carried out on an interdisciplinary basis with the radio-oncologist in charge and in communication with the specialist colleague in charge. The importance of tumour follow-up can be seen from the fact that around a quarter of patients with oropharyngeal and hypopharyngeal carcinoma experience a local tumour recurrence, which usually occurs within the first two years; even in the third year after primary treatment has been completed, recurrences still develop in a few cases [722]. The main aim of tumour follow-up care is therefore the careful examination of the larynx and neck to rule out recurrent carcinomas, which, according to the results of a retrospective study, only lead to symptoms in 61% of cases and are therefore not noticed by 39% of patients [723]. A further benefit of tumour follow-up is the detection of metachronous secondary tumours in the upper aerodigestive tract and lungs, which are associated with a similar risk profile to oro- (excluding HPV) and hypopharyngeal carcinomas and occur in 4–33% of patients [722].

Furthermore, the assessment of the functional, aesthetic and psychological follow-up condition (speech and swallowing function), the pain status and the need for rehabilitative (speech therapy, swallowing training) or supportive measures (pain therapy, nutritional therapy, physiotherapy, psycho-oncological and social care, lymphatic drainage), as well as advice on possible operations to improve functioning is the task of tumour aftercare [626], [685], [724] (Ch. 10).

The maximum follow-up intervals are 3 months for the 1^st^ and 2^nd^ year and 6 months for the 3^rd^ to 5^th^ year (analogous to recommendations in the S3 Guideline for Laryngeal Carcinoma [2] and the S3 Guideline for Oral Cavity Carcinoma [1]). After the 5^th^ year, an individual decision can be made as to whether further follow-up care appears necessary. In the event of a particular risk constellation or acute symptoms, more frequent examinations may be necessary in cooperation with the specialist colleagues providing care, which may also extend beyond 5 years.

The examinations required at each follow-up appointment are a careful and systematic inspection of the mouth, throat and larynx. In addition, an examination of the throat by palpation and possibly ultrasound is necessary. Indications of the possible presence of a tumour recurrence can also be ascertained by taking a history of pain, blood admixtures, weight loss, listlessness, etc. (for more details, see Chapter 7.1).

### 11.2 Imaging in aftercare

**11.2 Consensus-based recommendation 2024**

*In routine follow-up care, sectional imaging (CT, MRI) and the time intervals between examinations should be* *indicated* *depending on the risk profile, the stage of the tumour and the form of therapy.*

- EC
- Strong consensus

Imaging plays an important role in the aftercare of oropharyngeal and hypopharyngeal cancer and is an important part of the aftercare examination alongside the ENT specialist’s mirror examination/endoscopy. High-resolution CT is very sensitive in the area of the oropharynx and hypopharynx and is the procedure of first choice. In the same session, the lymphatic drainage channels of the neck can be visualized to detect or rule out metastases. MRI can be advantageous when it comes to the question of cartilage infiltration in hypopharyngeal carcinoma and the question of soft tissue contrast. Depending on the initial tumour category, the time interval for follow-up care should be determined by imaging. For larger carcinomas, the first imaging is recommended 3 months after the end of treatment. If there is an increased risk of distant metastasis, a CT thorax and abdomen should also be performed during the follow-up examination, possibly PET-CT [725], [726].

After definitive radiochemotherapy or radiotherapy with a good clinical response, contrast-enhanced baseline cross-sectional imaging of the head and neck area can be performed 8–12 weeks after the end of treatment to assess the post-therapeutic local findings. The choice of imaging modality (CT, MRI) should depend on the initial tumour location and the initial tumour or N category in accordance with the recommendations in the chapter on primary diagnostics (Section 7.3).

If a locoregional recurrence or residual tumour, distant metastasis or a second tumour is suspected on the basis of these examinations, FDG-PET/CT may be considered. There is no evidence that regular chest X-rays or the determination of tumour markers in serum have any benefit in tumour aftercare, so this is not recommended.

In March 2017, the Joint Federal Committee decided in accordance with Section 91 SGB V that, to avoid invasive procedures such as neck dissection and laryngoscopic biopsy, PET/CT for head and neck tumours will be covered by health insurance. This applies in particular to patients with laryngeal cancer following primary radiochemotherapy (Section 7.3).

### 11.3 Value of panendoscopy in follow-up care

A panendoscopy under anaesthesia is only indicated as part of the follow-up care of primary oropharyngeal and hypopharyngeal carcinoma if a tumour recurrence in the upper aerodigestive tract is suspected during the clinical examination. As explained in Section 7.4 and the Evidence Table “PICO 5: Panendoscopy vs. other imaging techniques”, panendoscopy is an obligatory part of the diagnostic work-up for suspected recurrence or second carcinoma.

### 11.4 Molecular diagnostics, screening in aftercare

**11.3 Consensus-based recommendation 2024**

*There are no established tumour markers for molecular diagnostics in the follow-up care of patients with oropharyngeal and hypopharyngeal carcinomas.*

- EC
- Strong consensus

There are currently no established tumour markers for molecular diagnostics in the follow-up care of patients with oropharyngeal and hypopharyngeal carcinoma that have been validated by sufficiently large retrospective and prospective studies. Various scientific approaches to molecular diagnostics, liquid biopsy, etc. are currently being investigated, but have not yet reached validated clinical maturity. There are also no serological screening approaches available for HPV-positive oropharyngeal carcinoma that could currently be recommended in follow-up care (see also Section 5.3). The molecular tumour board can be a useful addition in special recurrence situations.

### 11.5 Social and psychosocial counselling

**11.4 Consensus-based recommendation 2024**

*Socio-legal and psychosocial counselling should be part of the long-term care of patients with oropharyngeal and hypopharyngeal carcinomas.*

- EC
- Strong consensus

In recent years, the risk of falling into a precarious economic situation due to cancer has increased significantly. On the one hand, expenses increase and on the other, income is often significantly reduced due to the receipt of sickness benefit or a reduced earning capacity pension. Illness as a cause of over-indebtedness is now a well-known problem.

For this reason, an important goal of the social and socio-legal counselling services is to secure material and economic existence as well as participation in working life and to enable access to social benefits. The following counselling services are therefore part of the social and socio-legal counselling:

- Comprehensive information on social law (health, pension, long-term care insurance, severely disabled person’s pass)
- Assistance in filling out applications and submitting applications
- Advice on options for maintaining employment, initiating professional reintegration, initiating professional rehabilitation measures
- Psychosocial counselling on coping with illness, in conflict situations, participation in social life
- Information about counselling centres (integration service, addiction and debt counselling, pension insurance service centres)

In order to ensure psychosocial and socio-legal counselling, it is important that patients with laryngeal carcinoma receive initial oncological counselling from the social services of acute hospitals and rehabilitation facilities in an inpatient context. This is the only way to ensure that those affected and their relatives receive basic psychosocial information and that initial rapid interventions are possible if necessary.

Outpatient cancer counselling centres offer a low-threshold psychosocial counselling service for the phase after inpatient treatment on psychological, social and socio-legal burdens and provide information on self-help groups and other counselling services (analogous to the S3 Guideline for Larynx Carcinoma [2]).

## 12 Supply structures

**12.1 Consensus-based recommendation 2024**

*The multidisciplinary care of patients with oropharyngeal and hypopharyngeal carcinomas should be provided by facilities that meet the certification requirements of the German Cancer Society (certified head and neck tumour centres).*

- EC
- Strong consensus

The patient should receive the best possible care in accordance with the latest medical knowledge. In order to achieve an optimal result in terms of functional preservation, quality of life and longevity, the cooperation of different experts is required. This presupposes that the patient is cared for at highly qualified centres (preferably certified head and neck tumour centres according to the German Cancer Society), in which he can be comprehensively informed about the treatment options of head and neck surgery, radiotherapy and internal oncology and in which or under whose coordination the chosen therapy (including psycho-oncological and phoniatric care) and rehabilitation can be implemented.

Since its inception 20 years ago, the DKG’s certification concept in Germany has been supported by all specialist oncology societies and patient representatives and has been continuously developed on the basis of the guidelines. As of March 31, 2022, there were 1,778 DKG-certified centres, 148 of which were located abroad. The centres are represented at around 430 hospitals in Germany and in 2019, 56% of newly diagnosed patients were treated in a certified centre. According to the annual report of the Head and Neck Cancer Centres 2023, 11,299/year of primary head and neck cases in Germany (i.e. approx. 60% of the approx. 19,000 cases in total; HNSCC alone 18,000) are currently treated in 76 certified centres (DKG).

The WiZen results (effectiveness of care in oncology centres, Dresden Study 2022 based on approx. 1 million data sets from AOK routine data + data from clinical cancer registries [727], [728], [729]) show for the first time a clear survival advantage for almost all major cancer entities after treatment in certified centres. This study also included 15,287 patients with head and neck tumours from 2009–2017, 11,325 of whom were treated outside certified centres and only 3,962 within certified centres. **A significant survival benefit after treatment in a certified centre was demonstrated.** This effect remained stable, regardless of the overall size of the hospital. The advantage of centre treatment was clearer for patients with stages I–III than with IV. Across all stages, the advantage of having been treated in a certified centre was significantly different for a certification period of >2 years (HR 0.9; CI 0.83–0.97) [728]. The differences in the subgroup of R0-resected patients with stage I–III head and neck tumours with regard to recurrence-free survival were impressive (HR 0.81; CI 7.2–9.2). The number 341, which is the calculated sum of avoidable cancer deaths 5 years after initial diagnosis through treatment in certified centres in Germany compared to treatment in non-certified facilities, states this drastically [729].

In cost-effectiveness analyses, other publications also point to the correlation between lower treatment costs for treatment in certified cancer centres compared to non-certified treatment structures in Germany (e.g. colon cancer [730], [731]).

**The current findings indisputably speak in favour of concentrating the multidisciplinary care of patients with oropharyngeal and hypopharyngeal carcinomas in future on treatment facilities that meet the certification requirements of the German Cancer Society.**

Primary care ENT physicians/specialists and general practitioners must also be involved in the care according to the respective phase of the disease in order to ensure optimal care. The patient’s wish for optimal care can only be fulfilled by networking all the structures involved in the patient’s care. The additional counselling offered by self-help groups is of great importance. All professional/self-help groups involved in the patient’s care should therefore work together within the framework of network structures, through which the patient is guided in order to receive the appropriate care according to the respective phase of the disease. The coordination of patient care by the primary treating clinic (preferably a certified centre) but also the early involvement of patient self-help groups is of great importance.

## 13 Quality indicators

Quality indicators are measures that are used to assess the quality of the underlying structures, processes and results. Quality indicators are an important instrument of quality management. The aim of their use is the continuous improvement of care by presenting, critically reflecting on and, if necessary, improving the results of care. This selection of quality indicators was created in accordance with the methodology of the Oncology Guidelines Program [732]. A “Quality Indicators Working Group” (AG QI) was formed for the derivation process. This group created the final set of quality indicators based on the strong recommendations (“should”) of the newly developed guideline on oropharyngeal and hypopharyngeal carcinoma, as well as the results of the search for existing international quality indicators. The exact procedure and composition of the QI working group are described in the guideline report.

After two online meetings of this working group, the final set of 13 quality indicators (QI) was defined and adopted (Table 7 (Tab. 7)).

Please note in Table 7 (Tab. 7): The numerator is always a subset of the denominator. 

The quality indicators 6, 7, 8, 9, 12 and 13 are to be documented with the oncological basic data set of the cancer registries (as of 12/2023).

## Note

This guideline was first published by the Leitlinienprogramm Onkologie (Guideline Program in Oncology) at https://www.leitlinienprogramm-onkologie.de/leitlinien/oro-und-hypopharynxkarzinom [733].

## Funding

This guideline was funded by the German Cancer Aid as part of the German Guideline Program in Oncology.

## Competing interests

See Attachment 1.

## Supplementary Material

Competing interests

## Figures and Tables

**Table 1:**
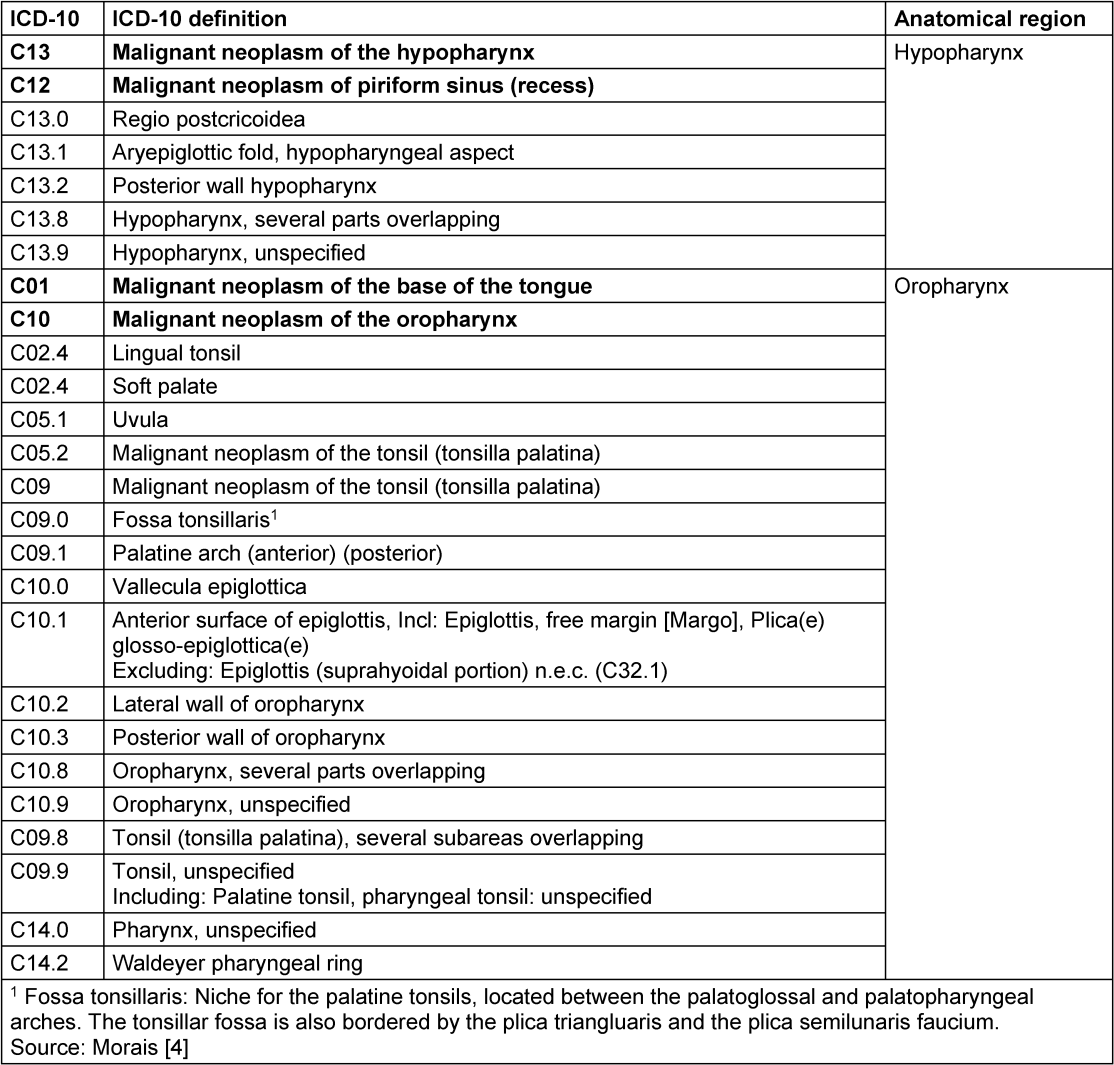
According to the ICD-10-GM-2022 code, the oropharynx and hypopharynx are subdivided as follows

**Table 2:**
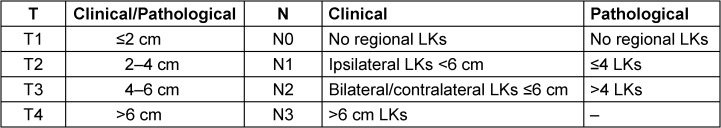
Oropharyngeal carcinoma TNM p16-positive: definition of the clinical and pathological T and N category

**Table 3:**
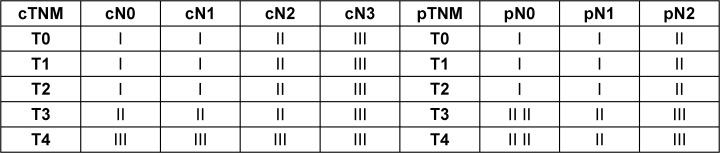
Oropharyngeal carcinoma TNM p16-positive: definition of clinical and pathological TNM staging, including unknown primary tumour (CUP)

**Table 4:**
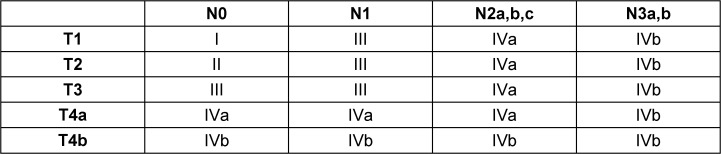
Oropharyngeal carcinoma TNM p16-negative: definition of TNM staging (clinically and pathologically identical; M1 always stage IVc)

**Table 5:**
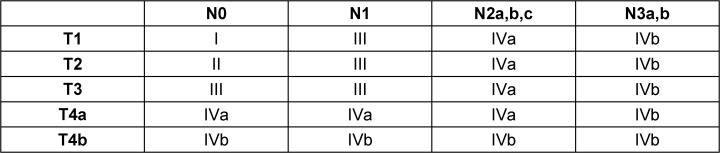
Hypopharyngeal carcinoma: definition of TNM staging (clinically and pathologically identical; M1 always stage IVc)

**Table 6:**
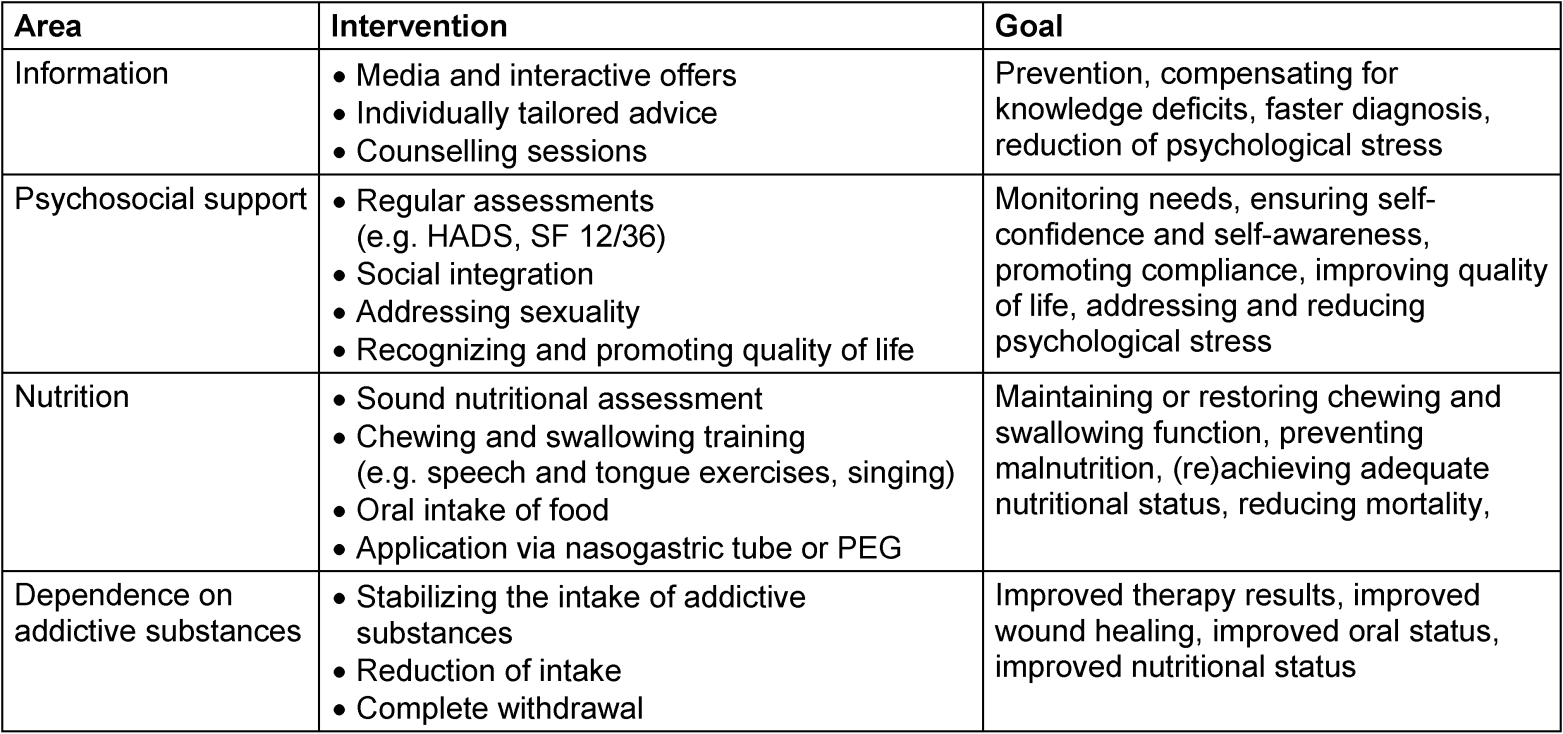
Special nursing care for OPC patients

**Table 7:**
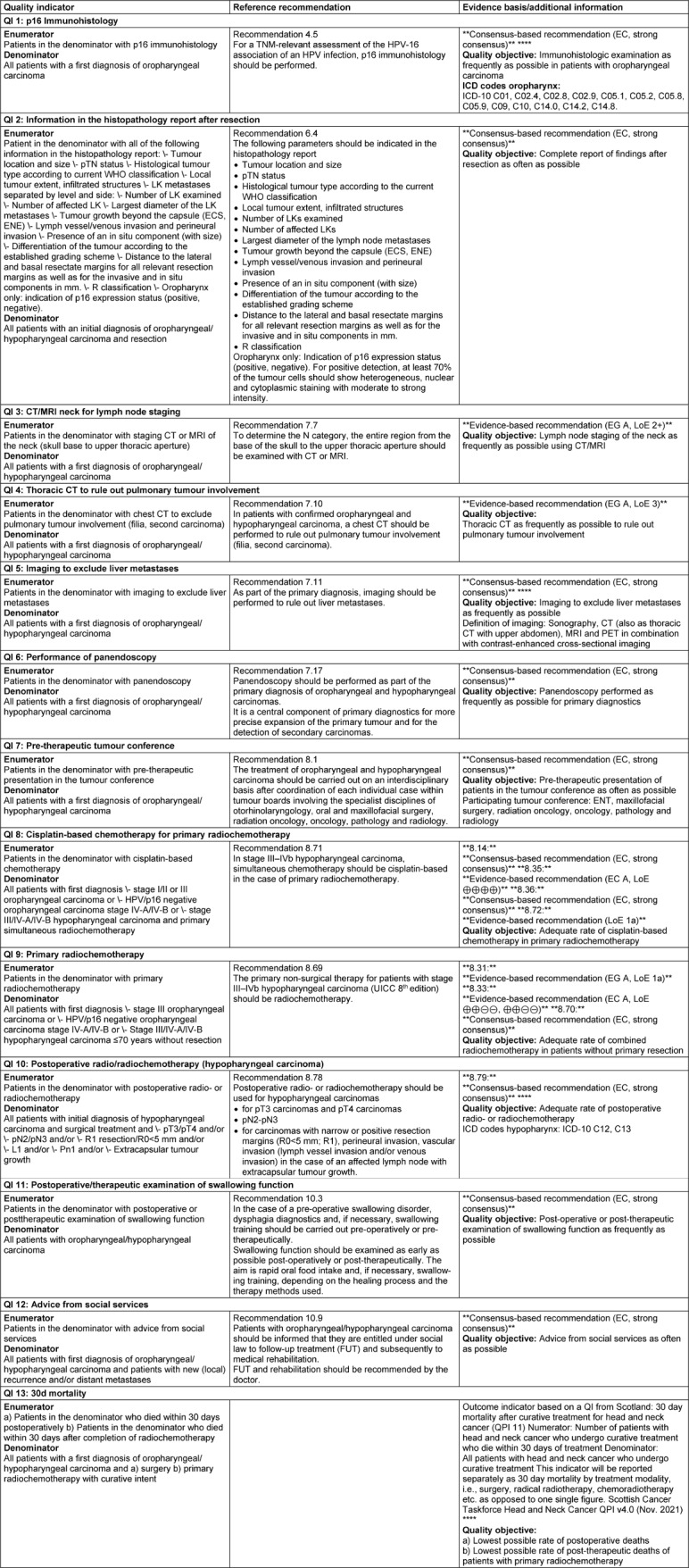
Quality indicators
