# Supplementary material for: Evidence-based guideline diagnosis, treatment, prevention and aftercare of oropharyngeal and hypopharyngeal carcinoma
Source: Ger Med Sci. 2025 Jun 24;23:Doc03. doi: 10.3205/000339 (PMC12247573; doi:10.3205/000339)
Supplement: Competing interests [file GMS-23-03-s-001.pdf]

Attachment 1: Competing interests

	Work as a consultant and/or assessor	Participation in a scientific advisory board	Paid lectures or training activities	Paid author or co-authorship	Research project/ conducting clinical studies	Owner-interests (patent, copyright share ownership)	Indirect interests	Guideline topics affected by COI, classification regarding relevance, consequence
Prof. Dr. Balempas, Panigiotis	No	No	No	No	No	No	Member: DEGRO<br>ARO<br>SRO<br>SASRO<br>ESTRO<br>EORTC<br>DKG, Scientific activity: Head and neck tumors<br>Stereotactic radiotherapy<br>Dermatological tumors, Clinical activity: Head and neck tumors<br>Stereotactic radiotherapy<br>Dermatological tumors	No topics (none), none
Bayer, Oliver	No	No	No	No	No	No	Scientific activity: cancer epidemiology, systematic reviews, qualitative research with cancer counseling centers, participation in further education/training: free workshops on systematic reviews at the University Medical Center Mainz (without remuneration,<br>- teaching in cross-sectional subject 1 (medicine): epidemiology, medical biometrics and computer science,<br>- Master’s degree in epidemiology,<br>- evidence-based medicine (EbM)	No topics (none), none
Prof. Dr. Brossart, Peter	BMS	BMS, Amgen	BMS, MSD, AstraZeneca	No	BMS	No	Scientific activity: Tumor immunology, Immuntherapien, Clinical activity: Hematology, Oncology, Participation in further education/training: Post-ASCO, Post-ASH, Expert Forum, Oncology Forum, Immuno-Oncology Update	No topics (moderat), Abstention

Attachment 1 to: Dietz A, Taylor K, Bayer O, Singer S, Follmann M, Nothacker M, Langer T, Klussmann P, Lang S, Hoffmann T, Maschmeyer G, Wiegand S, Fuchs M, Weichert W, Heß J, Guntinas-Lichius O, Waterboer T, Lell M, Büntzel J, Balermpas P, Schmidt K, Steingräber M, Klautke G, Hellmund H, Kissinger G, Brossart P, Maatouk I, Lethaus B, Raguse J, Zöphel K, Lippach K, Sterr F, Christiansen H, Duncker C, Keilmann A, Cici H, Yzer J, Relic A, Paradies K, Budach W. Evidence-based guideline diagnosis, treatment, prevention and aftercare of oropharyngeal and hypopharyngeal carcinoma. GMS Ger Med Sci. 2025;23:Doc03. DOI: 10.3205/000339, URN: urn:nbn:de:0183-0003395

	Work as a consultant and/or assessor	Participation in a scientific advisory board	Paid lectures or training activities	Paid author or co-authorship	Research project/ conducting clinical studies	Owner-interests (patent, copyright share ownership)	Indirect interests	Guideline topics affected by COI, classification regarding relevance, consequence
Prof. Dr. Budach, Wilfried	No	Merck, MSD, BMS	med publico GmbH, MSD, Merck, German Academy of Senology, Jörg Eickeler, consulting, organization, event, Düsseldorf	No	Pfizer and Merck	No	Member: DEGRO member 2015–2021 President Elect, President, Past President, Member: Professional Association of German Radiation Therapists e.V. Member 2017–2019 Member of the Board, Member: German Society for Senology Member, Member: ESTRO – Member, Member: ASTRO – Member, Member: ASCO – Member, Member: German University Association, Scientific activity: head and neck tumors, Scientific activity: breast cancer, Scientific activity: soft tissue sarcomas, Clinical activity: breast cancer, Clinical activity: head and neck tumors, Clinical activity: brain tumors, Clinical activity: prostate cancer.	No topics (moderat), Abstention
Prof. Dr. Büntzel, Jens	No	BMS	Merck Serono	No	BMS	No	Member: PRIO working group of the DKG, Member: DGHNOKC, Member: APM of the DKG, Scientific activity: Head and neck tumors, palliative medicine, supportive medicine, spiritual care, Clinical activity: Head and neck tumors, palliative medicine, supportive medicine, spiritual care	No topics (moderat), Abstention

	Work as a consultant and/or assessor	Participation in a scientific advisory board	Paid lectures or training activities	Paid author or co-authorship	Research project/ conducting clinical studies	Owner-interests (patent, copyright share ownership)	Indirect interests	Guideline topics affected by COI, classification regarding relevance, consequence
Prof. Dr. Christiansen, Hans	Medical office Niedersachsen/ Bremen, Ärztekammer Niedersachsen	Novocure, Nanobiotix, Bristol-Myers	Astra Zeneca, Onkologischer Arbeitskreis Hannover	Lehmanns – co-editor of a textbook on radiotherapy	Wolfgang-Dieckmann-Stiftung, Bahlsen-Stiftung	No	Member: Memberships: German Society for Radiooncology (DEGRO), Medical Association of Lower Saxony (ÄKN), Society for Pediatric Hematology and Oncology (GPOH), Society for Biological Radiation Research (GBS) German University Association (DHV), German Cancer Society (DKG), Lower Saxony Cancer Society (NKG), European Society for Radiotherapy and Oncology (ESTRO), Oncology Working Group Hannover (OAK), Medical Office of Lower Saxony/Bremen, Marburger Bund Lower Saxony, Scientific activity: clinical, experimental and translational radiooncology, Clinical activity: radiotherapy and radio-oncology, Participation in further education/training: Speaker of the DEGRO Academy for "Further education and training in radio-oncology"	No topics (moderat), Abstention
Cici, Havva	No	No	No	No	No	No	No	No topics (none), none
Prof. Dr. Combs, Stephanie	No	No	No	No	No	No	No	None
Prof. Dr. Dettmers, Stephan	No	No	No	No	No	No	No	None
Prof. Dr. Dietz, Andreas	Sanofi	MSD	MSD	BMS	Nanobiotix	GSK, Astra Zeneca, Novartis	Member: Spokesperson IAG-KHT of the DKG, Member: President/Past President DGHNO-KHT, Member: Member of the Board of Directors EHNS (European head and neck society), Scientific activity: Head and neck oncology, Clinical activity: All ENT medicine, focus on head and neck surgery and oncology, Participation in further education/ training: none,<br>Personal relationship: none	Medikamentöse Tumortherapie,<br>Moderat<br>Abstention

	Work as a consultant and/or assessor	Participation in a scientific advisory board	Paid lectures or training activities	Paid author or co-authorship	Research project/ conducting clinical studies	Owner-interests (patent, copyright share ownership)	Indirect interests	Guideline topics affected by COI, classification regarding relevance, consequence
Dr. Duncker, Christian	No	No	No	No	IRF Reha-wissenschaftliches Institut Universität Ulm	No	Member: Working group spokesman of the ATO Baden Württ. for oncological rehabilitation, Scientific activity: Rehabilitation for head and neck patients, Clinical activity: oncological rehabilitation	No topics (none), none
Dr. Follmann, Markus	Reviewer of various journals<br><br>Expert for the G-BA	No	Lectures on guideline methodology AWMF	No	No	No	Member: German Network for Evidence-Based Medicine, GRADE, Guidelines International Network, Scientific activity: EBM methodology and guidelines, LL-based quality indicators; QS cycle in oncology, Clinical activity: non-clinical activity, Participation in further education/ training: Speaker at guideline methodology WS of the OL and AWMF Guideline Advisory Seminars, Personal Relationship: No	No topics (none), none
Prof. Dr. Fuchs, Michael	Hochschule für Musik und Theater Leipzig, Oper Leipzig	Expert Advisory Board of the Federal Association of Throat Surgery Patients	Sächsische Landesärzte-kammer	No	No	No	Member: Board member of the German Society for Phoniatrics and Paedaudiology, Member: Member of the expert advisory board of the Federal Association of Throat Surgery Patients, Member: Contact person/advisor of the Saxony State Association of Throat Surgery Patients, Scientific activity: Scientific publications on head and neck tumors, focus on functionality (voice, speech, swallowing), Clinical activity: Clinical care of patients with head and neck tumors, focus on functionality (voice, speech, swallowing), Participation in further education/training: Co-organizer of oncological training courses on head and neck tumors, focus on functionality (voice, speech, swallowing)	No topics (none), none

	Work as a consultant and/or assessor	Participation in a scientific advisory board	Paid lectures or training activities	Paid author or co-authorship	Research project/ conducting clinical studies	Owner-interests (patent, copyright share ownership)	Indirect interests	Guideline topics affected by COI, classification regarding relevance, consequence
Prof. Dr. Guntinas-Lichius, Orlando	GBA	Merz	No	No	MEDEL, Innsbruck	Patent: System and Method for Facial Nerve Stimulation	Member: German Society for Otorhinolaryngology (DGHNO), Scientific activity: Facial paralysis, head and neck tumors, salivary gland tumors, postoperative ENT pain, Clinical activity: Facial paralysis, head and neck tumors, salivary gland tumors, Participation in further education/training: No	No topics (none), none
Dr. med. Hackenberg, Ulrich	None	None	No	No	No	No	Member: No, Scientific activity: non-scientific activity, Clinical activity: non-clinical activity, Participation in further education/training: No, Personal relationship: No	No topics (none), none
Hellmund, Herbert	None	No	No	No	No	No	Member: Federal Association of Laryngeal Surgery Patients e. V. President, Scientific Activity: No, Clinical Activity: None, Participation in Further Education/Training: No, Personal Relationship: No	No topics (none), none
Prof. Dr. Heß, Jochen	MSD SHARP DOHME GMBH, Bristol-Myers Squibb, Wilhelm Sander-Stiftung, Swiss Cancer League, European Science Foundation	No	MedKom Akademie GmbH, Merck Healthcare Germany GmbH	No	PROGEN Biotechnik GmbH, CureVac AG	No	Member: German Society for Otorhinolaryngology, Head and Neck Surgery (DGHNO), Member: German Society for Cell Biology (DGZ), Member: Society for Biochemistry and Molecular Biology (GBM), Member: European Association for Cancer Research (EACR), Member: European Head and Neck Society (EHNS), Member: Interdisciplinary Working Group for Head and Neck Tumors from the German Cancer Society (IAG-KHT), Member: Collegium Oto–Rhino–Laryngologicum Amicitiae Sacrum (CORLAS), Scientific activity: Experimental and Translational Head and Neck Oncology	No topics (not yet evaluated), none

	Work as a consultant and/or assessor	Participation in a scientific advisory board	Paid lectures or training activities	Paid author or co-authorship	Research project/ conducting clinical studies	Owner-interests (patent, copyright share ownership)	Indirect interests	Guideline topics affected by COI, classification regarding relevance, consequence
Prof. Dr. Hoffmann, Thomas	No	MSD, BMS, Merck	No	No	MSD, BMS, AZ, Merck, Sanofi, Pfizer	No	Member: DGHNO, Society for Skull Base Surgery, Professional Association, Scientific Activity: Oncology, Immunotherapy, Oncosurgery, Clinical Activity: Oncology, Immunotherapy, Oncosurgery, Participation in Further Education/Training: Multiple Advanced Training Courses in Oncology	No topics (moderat), Abstention
Karrasch, Dagmar	No	No	No	No	No	No	No	None
Prof. Dr. Keilmann, Annerose	No	Member of the scientific advisory board of the German Association of Speech Therapists, member of the scientific board of the KIND Foundation	No	Book chapter together with S. Lübbe: Rehabilitation of voice, speech and swallowing disorders	No	No	Member: President, then past president of the DGPP, Scientific activity: Therapy of voice and swallowing disorders of various origins, Clinical activity: Therapy of voice and swallowing disorders of various origins	No topics (none), none
Kissinger, Gunthard	No	No	No	No	No	No	No	No topics (none), none
Prof. Dr. Klaus, Zöphel	No	No	Bayer Healthcare	No	No	No	Member: Society of Nuclear Medicine and Molecular Imaging, German Society for Nuclear Medicine, Society for Nuclear Medicine of Central Germany, Scientific activity: PET in oncology, especially in radiation planning, in vitro diagnostics in thyroidology (see Pubmed), Clinical activity: PET and nuclear medicine therapy in oncology	No topics (none), none
PD Dr. Klautke, Gunther	No	Astra Zeneca, Merck Serono	Astra Zeneca, Lilly, Meck Serono, Roche, Bristol Myers Squibb, Boehringer Ingelheim, Sennewald, Brainlap	Bristol Myers Squibb	Astra Zeneca	No	Member: DEGRO, Member: Professional Association of German Radiotherapists	No topics (moderat), Abstention

	Work as a consultant and/or assessor	Participation in a scientific advisory board	Paid lectures or training activities	Paid author or co-authorship	Research project/ conducting clinical studies	Owner-interests (patent, copyright share ownership)	Indirect interests	Guideline topics affected by COI, classification regarding relevance, consequence
Prof. Dr. Klussmann, Peter	MSD	MSD	BMS	Merck	No	No	No	No topics (moderat), Abstention
Kristina, Lippach	No	No	No	No	Sturm-Stiftung München	No	Member: German Society for Nursing Science (DGP) – Mandate holder for this guideline, Member: Conference of Oncological Nursing and Pediatric Nursing (KOK) – KOK mandate holder in the certification commission of the DKG, Scientific activity: Publications on oncological nursing topics (oncological nursing visits, nursing-oncological initial interview, assessments), Participation in further education/ training: Staff unit for human resources development and nursing science – Participation in the implementation of specialist training	No topics (none), none
Prof. Dr. Lang, Stephan	No	No	MedKom Akademie	No	No	No	No	No topics (none), none
Prof. Dr. Dr. Lethaus, Bernd	Ärztekammer Nordrhein	No	Strasbourg Osteosynthesis Group (S.O.R.G)	No	Frauenhofer Institut	No	Member: DGMKG, Member: Scientific Working Group (AKWi) of the DGMKG, Member: European Association of Cranio-Maxillofacial Surgery, Member: LKG Working Group, Scientific activity: Malformation surgery, oncology, reconstruction, Clinical activity: Malformation surgery, oncology, reconstruction, Participation in further education/training: Strasbourg Osteosynthesis Group (S.O.R.G), Personal relationship: none	No topics
Prof. Dr. Maatouk, Imad	No	AG Psycho-onkologie in der Deutschen Krebsgesell-schaft	Vivantes AG Further training in psychosocial oncology	No	DFG, BMBF	No	Member: German Cancer Society German College for Psychosomatic Medicine, German Society for Medical Psychology, Scientific Activity: Psycho-oncology, Quality of Life Research, Clinical Activity: Psycho-oncology	No topics (none), none

	Work as a consultant and/or assessor	Participation in a scientific advisory board	Paid lectures or training activities	Paid author or co-authorship	Research project/ conducting clinical studies	Owner-interests (patent, copyright share ownership)	Indirect interests	Guideline topics affected by COI, classification regarding relevance, consequence
Prof. Dr. Maschmeyer, Georg	No	No	Merck-Serono, Gilead Sciences, AstraZeneca, RG GmbH Gräfeling, Forum für medizinische Fortbildung, MedUpdate GmbH, OSHO Services GmbH, Uniklinik Kiel, Uniklinik Leipzig, Landesapothekerkammer Baden-Württemberg, Yesnssen-Cilag, GlaxoSmithKline	No	No	No	Member: German Society for Hematology and Medical Oncology (DGHO); collaboration in the coordination of Onkopedia guidelines, Member: German Society for Hematology and Medical Oncology (DGHO), Member: European Conference on Infections in Leukemia (ECIL), Member: Working Group on Infections in Hematology and Oncology of the DGHO, Member: Interdisciplinary Working Group on Head and Neck Tumors of the German Cancer Society, Member: German Medical Association, Member: Drug Commission of the German Medical Association, Scientific activity: Infections in immunocompromised individuals; multiple myeloma; head and neck tumors; palliative medicine, Clinical activity: Chief physician of the Clinic for Hematology, Oncology and Palliative Medicine, Ernst bon Bergmann Hospital in Potsdam, Participation in further education/training: see above; and annual course on drug-based tumor therapy for head and neck tumors at the University Hospital UKE Hamburg	No topics
Prof. Dr. med. Michael, Lell	No	No	Siemens Healthcare, Siemens Healthcare	No	Siemens Healthcare, Medical Communications	No	Member: German X-ray Society, Head and Neck Working Group, Member: German Medical Association, Member: Bavarian State Medical Association, Scientific activity: Oncological imaging Cardiovascular imaging Dose reduction and AI, Clinical activity: Oncological imaging Cardiovascular imaging, Participation in further education/ training: German X-ray Society, Bavarian X-ray Society	No topics (none), none

	Work as a consultant and/or assessor	Participation in a scientific advisory board	Paid lectures or training activities	Paid author or co-authorship	Research project/ conducting clinical studies	Owner-interests (patent, copyright share ownership)	Indirect interests	Guideline topics affected by COI, classification regarding relevance, consequence
Prof. Dr. Mönig, Stefan	No	No	No	No	No	No	No	None
Nothacker, Monika	No	1. Health services research project "ZWEIT" (relevance of second opinions) without remuneration 2. Health services research project INDiQ (measurement of indication quality from routine data – remuneration as stated 3. National Cancer Plan steering group without remuneration, IQTIG	Berlin School of Public Health	No	German Cancer Society (DKG), University Medicine Network, BMG, University Medicine 2.0 Network, G-BA Innovation Fund	No	Member: - German Network for Evidence-Based Medicine (member) - German Cancer Society (member until 12/2020) - Guidelines International Network/GRADE Working Group (member), Scientific activity: Guidelines and guideline methodology. Prioritization of guideline recommendations (Making Smart Decisions Together), quality indicators, topic-related reviews, Clinical activity: non-clinical activity, Participation in further education/ training: Guideline seminars for guideline developers/consultants as part of the curriculum for guideline consultants of the AWMF 1-3/Yeshr, Personal relationship: No	No topics (none), none
Paradies, Kerstin	No	No	No	No	No	No	No	None
PD Dr. Dr. Raguse, Yesn-Dirk	No	BMS, Sanofi Aventis	MDHNO	No	KLS Martin	No	Member: DGMKG, Member: DÖSAK, Member: DKG, Member: EACMFS, Scientific activity: HNSCCC, Clinical activity: HNSCC	No topics (moderat), Abstention
Dr. Relic, Alessandro	No	No	No	No	No	No	Member: Federal Association of Attending Physicians	No topics (none), none
Prof. Dr. Rhode, Stefan	No	No	No	No	No	No	No	None
Schmidt, Kerstin	No	No	No	No	No	No	No	No topics (none), none
Prof. Dr. Dr. Schultze-Mosgau, Stefan	No	No	No	No	No	No	No	None

	Work as a consultant and/or assessor	Participation in a scientific advisory board	Paid lectures or training activities	Paid author or co-authorship	Research project/ conducting clinical studies	Owner-interests (patent, copyright share ownership)	Indirect interests	Guideline topics affected by COI, classification regarding relevance, consequence
Prof. Dr. Singer, Susanne	No	Content Ed Net, Lilly	No	Pfizer	No	No	Member: EORTC Quality of Life Group, Member: EORTC Head and Neck Cancer Group, Member: AG PSO of the DKG, Scientific activity: Quality of life research, oncology, mental health and psychosocial care of cancer patients, psychotherapy, EbM, social epidemiology, Clinical activity: currently not clinically active, Participation in further education/training: Board member of the Mainz Psychoanalytic Institute	No topics (none), none
Prof. Dr. Smola, Sigrun	No	No	No	No	Helmholtz-Institut für Pharmazeuti-sche Forschung Saarland (HIPS) Helmholtz-Zentrum für Infektions-forschung (HZI)	No	Member: Society for Virology, Member: Professional Association of Doctors for Microbiology, Virology and Infectious Epidemiology, Member: Deputy Spokesperson of the Department of Virology, Dept. of Cancer Research (AEK) of the DKG, Member: Member of the guideline group for the S3 guideline “Vaccination prevention of HPV-associated neoplasia”, Member: (Deputy) Chair of the Central Commission for Biological Safety (ZKBS), Member: Scientific Advisory Board of the Paul Ehrlich Institute, Langen, Scientific activity: Infections caused by human papilloma viruses, Clinical activity: Virological diag-nostics, Personal relationship: Hus-band works for Paul Hartmann AG.	No topics (none), none
Dr. Steingräber, Maria	No	No	No	No	No	No	No	No topics (none), none
Sterr, Fritz	No	No	No	No	No	No	Member: German Society for Nursing Science e.V. (DGP), Deputy Representative, Member, Member: German Society for Specialist Nursing and Functional Services e.V. (DGF), Member, Member: German Professional Association for Nursing Professions (DBfK), Member, Member: State Nursing Chamber of Rhineland-Palatinate, Member	No topics (not yet evaluated), none

Attachment 1 to: Dietz A, Taylor K, Bayer O, Singer S, Follmann M, Nothacker M, Langer T, Klussmann P, Lang S, Hoffmann T, Maschmeyer G, Wiegand S, Fuchs M, Weichert W, Heß J, Guntinas-Lichius O, Waterboer T, Lell M, Büntzel J, Balermpas P, Schmidt K, Steingräber M, Klautke G, Hellmund H, Kissinger G, Brossart P, Maatouk I, Lethaus B, Raguse J, Zöphel K, Lippach K, Sterr F, Christiansen H, Duncker C, Keilmann A, Cici H, Yzer J, Relic A, Paradies K, Budach W. Evidence-based guideline diagnosis, treatment, prevention and aftercare of oropharyngeal and hypopharyngeal carcinoma. GMS Ger Med Sci. 2025;23:Doc03. DOI: 10.3205/000339, URN: urn:nbn:de:0183-0003395

	Work as a consultant and/or assessor	Participation in a scientific advisory board	Paid lectures or training activities	Paid author or co-authorship	Research project/ conducting clinical studies	Owner-interests (patent, copyright share ownership)	Indirect interests	Guideline topics affected by COI, classification regarding relevance, consequence
Taylor, Katy	No	No	No	No	No	No	Member: EORTC Quality of Life Group	No topics (none), none
Dr. Waterboer, Tim	No	MSD (Merck) Sharp Dohme	No	No	No	No	No	No topics (moderat), Abstention
Prof. Dr. Weichert, Wilko (✞10 July 2023)	See below	Advisory boards and invited speaker for Roche, MSD, BMS, AstraZeneca, Pfizer, Merck, Lilly, Boehringer, Novartis, Takeda, Bayer, Amgen, Astellas, Illumina, Eisai, Siemens, Agilent, ADC, GSK and Molecular Health	See above	No	Research support from Roche, MSD, BMS, and AstraZeneca	No	Member: DGP, BV Deutscher Pathologen, AACR, ESMO, ESP, Hochschulverband, Scientific activity: Morphological and molecular diagnostics, biomarker analysis, including in head/neck cancer, Clinical activity: Pathological diagnostics, including head/neck area	No topics (moderat), Abstention
Wenzel, Gregor	No	No	No	No	No	No	No	No topics
Prof. Dr. med. Wiegand, Susanne	No	BMS, MSD, Merck Serono, Nanobiotix, Sanofi Genzyme	BMS, Astra Zeneca, MSD, Roche, Merck Serono	No, MSD	No	No	Member: Member of DGHNO, Scientific activity: Head and neck carcinoma, Clinical activity: Head and neck carcinoma	No topics (moderat), Abstention
Yzer, Jutta	No	No	No	No	No	No	Member: DVSG, German Association for Social Work in Health Care<br><br>Spokesperson for the LAG-Bremen	No topics (none), none
